# Ugi 5-center-4-component reaction of α-amino aldehydes and its application in synthesis of 2-oxopiperazines

**DOI:** 10.1007/s11030-023-10760-1

**Published:** 2023-12-17

**Authors:** Marta Splandesci, Martyna Z. Wróbel, Izabela D. Madura, Maciej Dawidowski

**Affiliations:** 1 Department of Drug Technology and Pharmaceutical Biotechnology, Faculty of Pharmacy, Medical University of Warsaw, Banacha 1, 02-097 Warsaw, Poland; 2 Faculty of Chemistry, Warsaw University of Technology, Noakowskiego 3, 00-664 Warsaw, Poland

**Keywords:** Multicomponent reactions, Ugi reaction, Ugi 5-center-4-component reaction, 2-Oxopiperazines, Molecular diversity

## Abstract

A synthetic route leading to densely functionalized 2-oxopiperazines is presented. The strategy employs a 5-center-4-component variant of Ugi multicomponent reaction followed by a deprotection/cyclization sequence. *N*-Boc-α-amino aldehydes were used for the first time as carbonyl components in a key Ugi 5-center-4-component reaction (U-5C-4CR). It is shown that the presented synthetic route can lead to rigid, heterocyclic scaffolds, as demonstrated by the synthesis of tetrahydro-2*H*-pyrazino[1,2-*a*]pyrazine-3,6,9(4*H*)-trione β-turn mimetic and derivatives of 1,6-dioxooctahydropyrrolo[1,2-*a*]pyrazine and 3,8-dioxohexahydro-3*H*-oxazolo[3,4-*a*]pyrazine.

**Graphical abstract:**

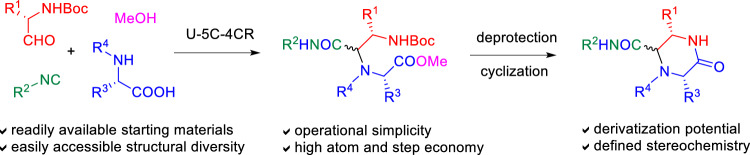

**Supplementary Information:**

The online version contains supplementary material available at 10.1007/s11030-023-10760-1.

## Introduction

Multicomponent reactions (MCRs) are reactions where more than two starting materials form a single product. Importantly, they proceed with high atom economy and they have propensity to generate molecular diversity by using simple, one-pot procedures, starting from a wide variety of readily available building blocks. These example features make the MCRs particularly attractive for the rapid synthesis of libraries of compounds for drug-discovery purposes [1].

**Scheme 1:**
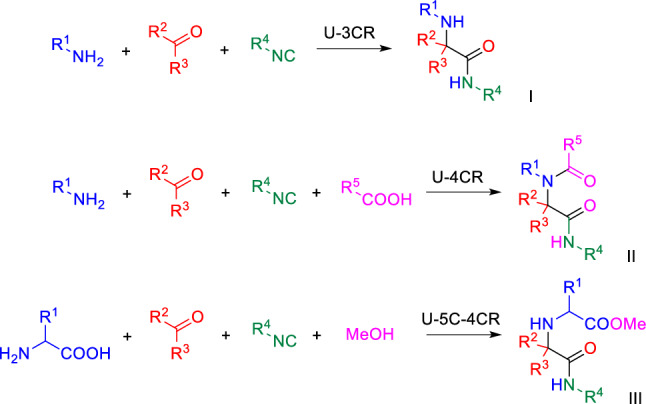
A general reaction course of U-3CR, U-4CR and U-5C-4CR

Among the known MCRs, the isocyanide-based Ugi reaction [2] has received much attention in medicinal [3, 4] and macrocyclic chemistry [5–7], chemical biology and bioconjugation [8–10], as well as in natural product synthesis [11, 12]. Several variants of this condensation have been developed, the Ugi three- (U-3CR) [13, 14] and four-component reactions (U-4CR) [15] being most extensively studied (Scheme 1). The first is a reaction of an amine with a carbonyl and an isocyanide, which results in a formation of an α-aminocarboxamide (**I**), while the latter employs an additional carboxylate component to produce an α-acylaminocarboxamide (**II**). These two reactions are often followed by the subsequent post-condensation modifications and both have been used as powerful tools for generating diverse scaffolds for drug discovery purposes [16]. Another interesting but less studied variant is the Ugi 5-center-4-component reaction (U-5C-4CR) [17], which employs carbonyls, isocyanides, alcohols and α- or β-amino acids as bifunctional reagents to provide α, α′-imino dicarboxylic acids (**III**). Similarly to U-3CR and U-4CR, U-5C-4CR has a proven potential to generate libraries of small-molecular scaffolds. Importantly, depending on the combination of condensation components, all variants of Ugi MCR can deliver C(sp^3^)-rich amino acid derivatives and peptidomimetics [18, 19].

**Fig. 1:**
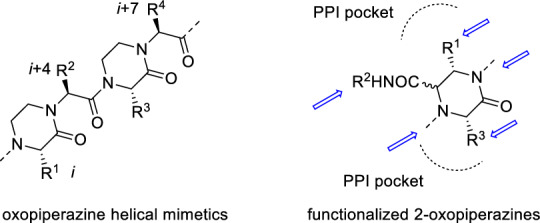
Structure of Arora’s oligooxopiperazine template that adopts stable conformations to reproduce the arrangement of *i*, *i* + *4*, and *i* + *7* residues on an α-helix (left) and designed, functionalized 2-oxopiperazines (right). Potential sites for generating structural diversity are marked with arrows

The 2-oxopiperazine framework is found in multiple biologically active compounds, such as arenavirus cell entry [20], factor Xa [21] and membrane-associated hepatitis C virus (HCV) Protein NS4B inhibitors [22], as well as in natural products [23, 24]. Recently, Arora and co-workers have shown that 2-oxopiperazine helix mimetics (OHMs, Fig. 1) can be useful templates for design of protein–protein interaction (PPI) inhibitors [25–27]. One of our projects utilized a variation of this approach and required access to highly functionalized 2-oxopiperazines (Fig. 1) projecting aromatic amino acid side chains towards their respective hydrophobic pockets localized in the PPI interface.

**Fig. 2:**
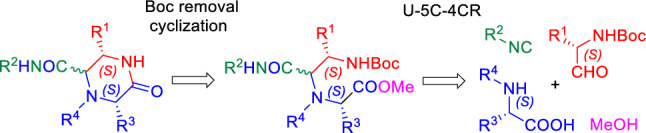
U-5C-4CR/deprotection/cyclization strategy to target, functionalized 2-oxopiperazines

Similar compounds have previously been synthesized by various strategies [23, 28–33], including those based on the U-4CR [30, 31], the ‘disrupted’ Ugi [30] and the Castagnoli–Cushman [31] MCRs. A retrosynthetic analysis showed that the target, functionalized 2-oxopiperazines can be assembled by a short Ugi/deprotection/cyclization sequence based on the U-5C-4CR variant (Fig. 2). The potential advantages of this approach are: low number of reaction steps; high atom economy; operational simplicity of the respective reactions; capability of functionalization of the 2-oxopiperazine scaffold with amino acid side chains; and potential of generating structural diversity at five atoms of the 2-oxopiperazine framework by varying the respective components of the U-5C-4CR, or by post-condensation modifications of secondary amine nitrogen in products for which N-unsubstituted amino acids are used as inputs. Moreover, a proper combination of accessible, enantiopure starting materials may lead to final products with a desired stereochemistry.

**Fig. 3:**
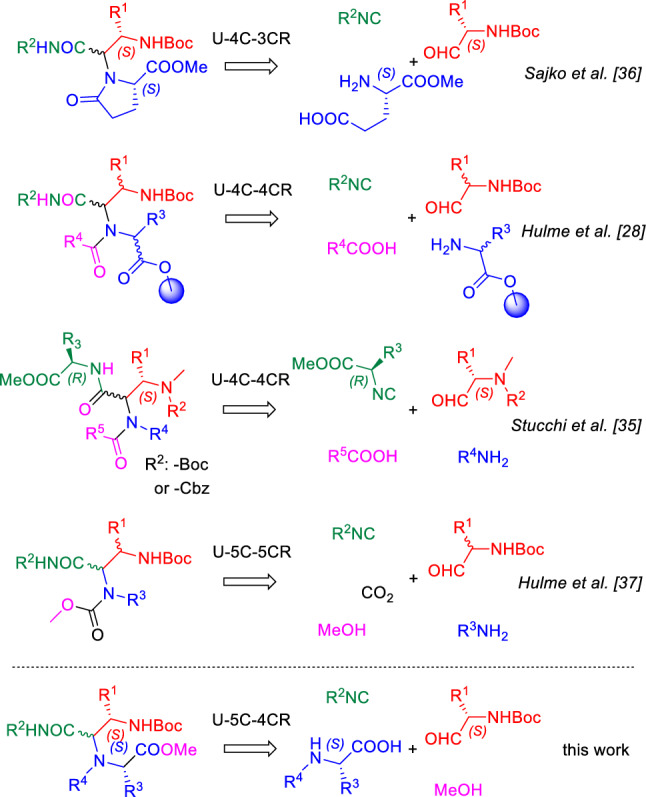
The outcomes of the reported Ugi condensations of *N*-protected α-amino aldehydes

The U-5C-4CR step of the proposed synthetic pathway employs the condensation of *N*-protected α-amino aldehydes with α-amino acids, isocyanides and MeOH. Although various carbonyl compounds have been used in the Ugi MCR, only a few literature reports exist on the condensations of *N*-protected α-amino aldehydes. Such components were coupled with the respective α-amino esters or amines in the ‘classical’ 4-center-4-component reaction (U-4C-4CR) [30, 34, 35] and the similar 4-center-3-component (U-4C-3CR) [36] and 5-center-5-component (U-5C-5CR) [37] variants, whereas no reports exist on their use as condensation partners for α-amino acids in the U-5C-4CR. It is worth noting, that the reported outcomes of the mentioned Ugi MCRs differed significantly, and each time the unique sets of products were formed (Fig. 3). Driven by the need for access to chiral 2-oxopiperazines functionalized with amino acid [28, 29] side chains and by the fact that their precursors, the unprecedented U-5C-4CR products, may open access to novel peptidomimetic chemical space, in this paper we investigate the usefulness of the *N*-protected α-amino aldehydes as carbonyl components in U-5C-4CR. Further, we show that such adducts can be efficiently used in cyclization reactions to the substituted 2-oxopiperazines having five potential diversity points and a defined stereochemistry. Finally, we demonstrate, that the proposed method can deliver useful intermediates for the assembly of rigid, C(sp^3^)-rich, bicyclic scaffolds.

## Results and discussion

Several factors influence the outcomes of U-5C-4CR, among them the nature and the degree of bulkiness of the condensation components, the temperature and the presence of the Lewis acid catalyst, which is postulated to facilitate the imine formation/activation during the early steps in reaction course [38–41]. We performed U-5C-4CR using optimized conditions (Table 1), with equimolar amounts of the *N*-Boc-protected α-amino aldehydes, α-amino acids and isocyanides in MeOH (0.5 M solution), in the presence of Sc(OTf)_3_, at 60 °C.

**Table 1:** Optimization of a model U-5C-4CR

Entry	Catalyst^a^	Temperature (°C)	Time (h)	Yield (%)^b^	dr^c^
1	–	23	12	34	–
2	–	60	12	63	73:27
3	Sc(OTf)_3_	60	12	72	72:28 (70:30)^d^
4	TiCl_4_	60	12	74	73:27
5	Sc(OTf)_3_	60	24	69	74:26

Reactions were performed on a 0.602 mmol scale in MeOH

^a^10 mol%

^b^Isolated yields

^c^dr values based on HPLC/MS analyses of the crude reaction mixtures

^d^dr value based on ^1^H NMR analysis of the sample purified by column chromatography on silica

**Scheme 2:**
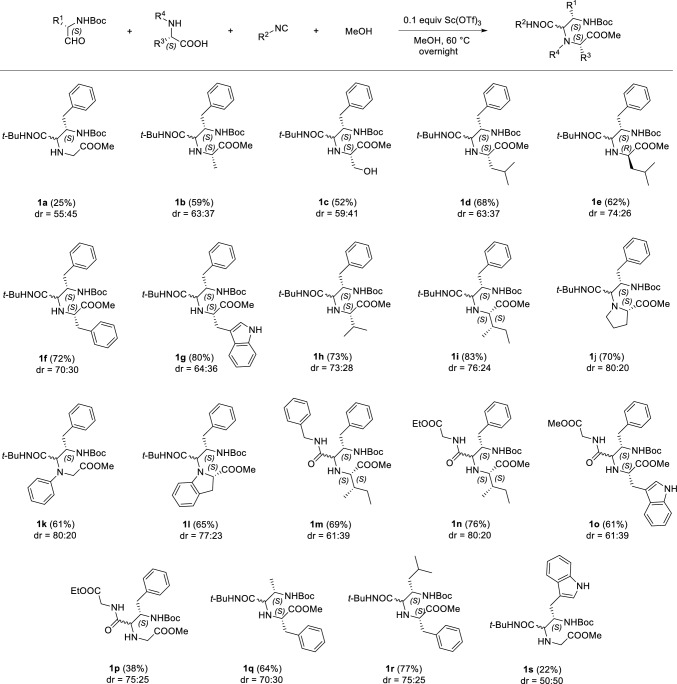
The substrate scope of U-5C-4CR of *N*-Boc-protected α-amino aldehydes. 1 Equiv of each U-5C-4CR component was used. Isolated yields refer to the sum of diastereoisomers. The dr values were estimated by ^1^H NMR and refer to the purified products

Using these conditions, we investigated the substrate scope of U-5C-4CR of *N*-Boc-protected α-amino aldehydes (Scheme 2). First, we tested various amino acids as coupling partners for *N*-Boc-*L*-phenylalaninal, *tert*-butyl isocyanide and MeOH. The products **1a–l** were formed with fair to very good yields, glycine adduct **1a** being the only notable exception (25%). In most cases, the yields only slightly depended on the steric bulk of the starting amino acids. The steric hindrance introduced by the branched or aromatic amino acid side chains did not affect the yields. Thus, the adducts **1f–i** were generally formed with similar yields (52–83%) to those obtained by U-5C-4CRs of less branched or less bulky amino acids **1a–e** (25–68%), the aforementioned glycinate adduct **1a** being the most notable example. Secondary amino acids have also proven suitable coupling components of U-5C-4CR, as illustrated by the products **1j–l** that were obtained with yields comparable to those observed for reactions of the primary amino acids (61–70%). Next, we tested the reactivity of the example, readily available isocyanides. In general, we observed similar or slightly lower yields of condensation products **1m–o** of linear isocyanides, as compared with the yields of their respective analogues **1i** and **1g** derived from *tert*-butyl isocyanide (**1m**: 69% vs. **1i**: 83%; **1n**: 76% vs. **1i**: 83%; **1o**: 61% vs. **1g**: 80%). Only the glycine derivative **1p** of ethyl isocyanoacetate was formed in a higher yield than its analogue **1a** obtained from *tert*-butyl isocyanide (38% vs. 25%, respectively). Finally, the molecular diversity of U-5C-4CR can be expanded by employing various *N*-protected α-amino aldehydes, as illustrated by the synthesis of compounds **1q-s** from *N*-Boc-*L*-alaninal, *N*-Boc-*L*-leucinal and *N*-Boc-*L*-tryptophanal, respectively.

A new stereocenter is formed in the course of U-5C-4CR and diastereoselectivity is observed when enantiopure chiral substrates are employed as the condensation components. In the examples **1a–s** shown (Scheme 2), at least one chiral starting material was used, and the observed diastereoselectivities were generally low to good (dr values up to 80:20). In general, we observed highest diastereoinduction for the U-5C-4CRs of branched and secondary *L*-amino acids. Interestingly, slightly higher dr values were obtained when linear ethyl isocyanoacetate was used instead of bulky *tert*-butyl isocyanide in the synthesis of glycine adducts **1a** and **1p**.

**Scheme 3:**
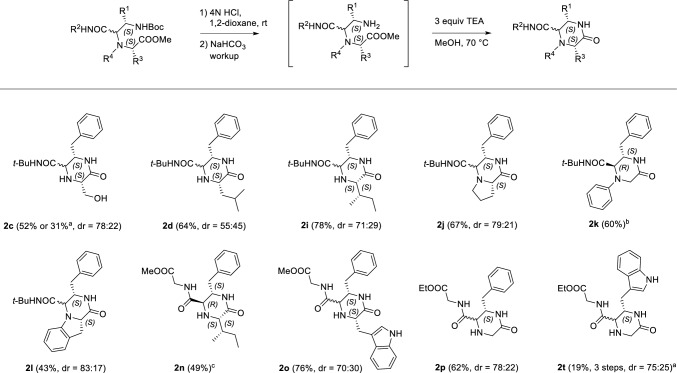
Deprotection/cyclization of the selected U-5C-4CR adducts **1** to substituted 2-oxopiperazines **2**. If not stated otherwise, the diastereoisomeric mixtures of **1** were used as starting materials. Isolated yields refer to the sum of diastereoisomers. dr values were measured by ^1^H analyses of purified products. The colors indicate the origin of atoms from U-5C-4CR: green, isocyanide; red, α-amino aldehyde; blue, α-amino acid; pink, alcohol. ^a^Synthesized without purification of the respective U-5C-4CR adducts, yield over 3 steps. ^b^A single diastereoisomer of **2k** was isolated. ^c^Compound **2n** was synthesized from the single **(2R, 3S)-1n** diastereoisomer

Next, we investigated the transformation of the U-5C-4CR products **1** to the target substituted 2-oxopiperazines **2** (Scheme 3). The selected U-5C-4CR adducts, **1d, i–l, n–p**, were subjected to Boc cleavage in acidic media, followed by the base-promoted cyclocondensation reaction. This operationally simple procedure gave the respective 2-oxopiperazines, **2d, i–l, n–p**, with moderate to good yields (43–78%). In the cyclocondensation step, the reaction times varied from several hours to 9 days. Apart from the fact that the fastest reactions took place in the case of the secondary aromatic amino acids (4 h for **2k** and **2l**), there was no clear trend between the cyclization times and the structures of the U-5C-4CR adducts **1** used as starting materials. We observed a transesterification reaction of ethyl to methyl ester in the synthesis of **2n**. This side reaction can be avoided by replacing MeOH with toluene, as demonstrated by the conversion of **1p** to 2-oxopiperazine **2p**. We next sought to simplify the U-5C-4CR-based synthesis of 2-oxopiperazines, omitting the chromatographic purification of the U-5C-4CR adducts. These attempts were successful, as shown in the synthesis of compounds **2c** and **2t**. These 2-oxopiperazines were formed in 31% and 19% yields, respectively, over 3 steps, which was acceptable bearing in mind the ease of the protocol and the structural complexity of the final products. The yield of **2c** obtained using this procedure was comparable to the one from the three-step synthesis employing purified Ugi adduct **1c** (31% *vs* 29%, overall).

**Scheme 4:**
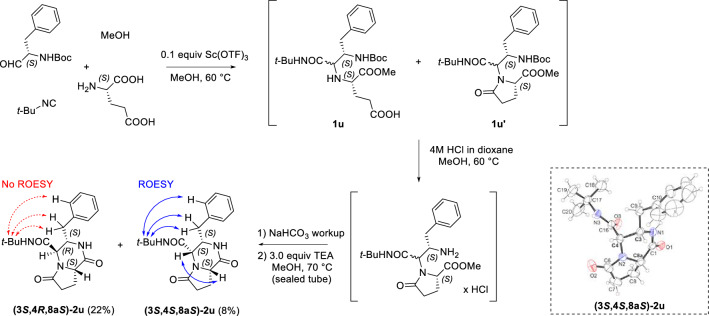
Synthesis and assignment of absolute configurations of diastereoisomers of **2u**. The crystal structure of the minor diastereoisomer **(3S, 4S, 8aS)-2u** is shown. The protons attached to the stereocenters C-3, C-4 and C-8a are in the axial, equatorial and axial positions, respectively, with regard to the 2-oxopiperazine ring of the fused bicyclic system

We obtained the bicyclic *bis*-lactam **2u** using a simplified protocol, which was similar to the one previously applied in the synthesis of **2c** and **2t** (Scheme 4). The HPLC–MS analysis of the U-5C-4CR of *L*-glutamic acid showed the formation of a complex mixture of byproducts and intermediates **1u** and **1u′**, separation of which would be impractical. Therefore, we performed a one-pot esterification of **1u**/Boc cleavage by simply heating the post-Ugi reaction mixture with HCl (addition of a 4N solution of HCl in 1,4-dioxane). The subsequent NaHCO_3_ workup followed by the cyclocondensation reaction gave **2u** in a 30% yield (dr = 73:27), over 3 steps. The obtained *bis*-lactam **2u** is a pyroglutamic acid analogue of the bicyclic proline derivative **2j**. Both compounds share a cyclo (Prol-Phe)-like 3-benzyl-perhydropyrrolo[1,2-*a*]pyrazin-1-one scaffold, which is present in potent thyroliberin antagonists [42]. Gratifyingly, no epimerization took place during the cyclocondensation step upon prolonged heating of Boc-deprotected U-5C-4CR adducts in the presence of excess of TEA. In most cases, the diastereoisomers of the 2-oxopiperazines **2** were separable by recrystallization and/or by column chromatography on silica. The obtained compounds are cyclic and rigid, which allowed assignment of the stereochemistry of the particular isomers by NMR. The ROESY experiments performed for the respective separated isomers of **2u** indicated that the diastereo induction favored the (*R*)-configuration on the stereo center created in the course of the U-5C-4CR (Scheme 4). To further support this, we solved and refined a crystal structure of the minor isomer of *bis*-lactam **2u**, which revealed a (3*S*, 4*S*, 8a*S*)-absolute configuration.

**Scheme 5:**
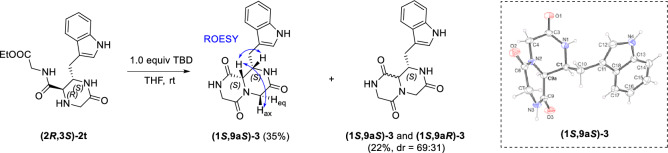
Synthesis and assignment of relative configurations of diastereoisomers of **3**. The crystal structure of the major diastereoisomer **(1S, 9aS)-3** is shown. The protons attached to the stereocenters C-1 and C-9a are in the equatorial and axial positions, respectively, with regard to the 2-oxopiperazine ring of the fused bicyclic system

In addition to the synthesis of 2-oxopiperazines **2** with five potential diversity points accessible by manipulation of the U-5C-4CR components, we investigated the simple transformations of these compounds that would potentially lead to other rigid, C(sp^3^)-rich heterocycles. First, we performed the 1, 5, 7-triazabicyclo[4.4.0]dec-5-ene (TBD)-triggered intramolecular cyclization of **(2R, 3S)-2t** to obtain **3**, a derivative of a tetrahydro-2*H*-pyrazino[1,2-*a*]pyrazine-3, 6, 9(4*H*)-trione scaffold, which had previously been shown to be a valuable scaffold for β-helical mimetics (Scheme 5) [31]. We isolated the major isomer of **3** by column chromatography. In a ROESY experiment, we observed a pronounced nOe between the proton attached to the bridgehead C-9a carbon atom and the protons in the C-1 and C-4 (axial) positions, which clearly suggested the (1*S*, 9a*S*)- configuration of this diastereoisomer (Scheme 5). The X-ray crystallography confirmed this assignment. This was in line with a literature report on high levels of epimerization at the C-9a stereocenter triggered upon exposure of compounds similar to **3** to a strong base, which favors a *cis*- configuration of the protons in C-9a and C-1 positions. Gratifyingly, it was also reported that the tetrahydro-2*H*-pyrazino[1,2-*a*]pyrazine-3,6,9(4*H*)-triones of this stereochemistry are capable of effectively mimicking the peptide β-turn [31].

**Scheme 6:**
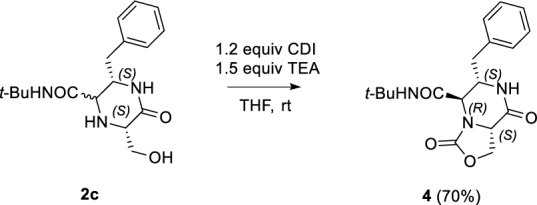
Synthesis of 3, 8-dioxohexahydro-3*H*-oxazolo[3, 4-*a*]pyrazine **4**

As demonstrated by the synthesis of **2u**, the derivatization potential of the 2-oxopiperazines obtained via U-5C-4CR/deprotection/cyclization sequence depends not only on the presence of a reactive group introduced by the isocyanide in the Ugi step (as in the synthesis of **3**), but can also result from the reactivity of a side chain of the α-amino acid involved in the condensation. In this context, we sought to explore the usefulness of a hydroxyl and secondary amino groups present in the *L*-serine derivative **2c**. A mild reaction of **2c** with 1, 1′-carbonyldiimidazole (CDI) in the presence of TEA afforded compound **4**, having a novel, drug-like 3, 8-dioxohexahydro-3*H*-oxazolo[3,4-*a*]pyrazine heterobicyclic scaffold (Scheme 6).

## Conclusions

In this study, we have shown that *N*-Boc-α-amino aldehydes efficiently couple with various α-amino acids, isocyanides and MeOH in the course of the U-5C-4CR. The condensations usually gave moderate to high yields, which is satisfactory bearing in mind the operational simplicity of the reaction and the levels of structural complexity of the respective products. We observed moderate diastereoselectivities, with the diastereo induction favoring the (*R*)-configuration of the newly created stereocenter. We have shown, that the obtained U-5C-4CR products may be useful for the construction of libraries of heterocycles that fulfill the drug-likeness criteria. First, we have designed a U-5C-4CR/deprotection/cyclization sequence leading to a library mono-, bi- and tricyclic 2-oxopiperazines functionalized with amino acid side chains. Further, we have shown, that our method may be a complementary approach to the MCR-based synthesis of the tetrahydro-2*H*-pyrazino[1,2-*a*]pyrazine-3, 6, 9(4*H*)-trione β-turn mimetics developed recently [31], which potentially leads to analogs with unique, amino acid-derived substitution patterns. Finally, by synthesizing the tetrahydropyrrolo[1,2-*a*]pyrazine-1,6(2*H*, 7*H*)-dione and the unprecedented 3,8-dioxohexahydro-3*H*-oxazolo[3, 4-*a*]pyrazine heterobicyclic, C(sp^3^)-rich scaffolds, we have shown that the convertible amino acid side chains may be useful for further derivatization of 2-oxopiperazines. In conclusion, our results show that the presented method can generate principal components of structural diversity (appendage, functional group, stereochemical and skeletal diversity) [43] within the 2-oxopiperazine framework, in an operationally simple manner, using commonly available reagents.

## Experimental section

### General methods

Reagents and solvents were purchased from commercial suppliers and used without further purification. *N*-Boc-*L*-tryptophanal was synthesized by a literature protocol [44]. Thin layer chromatography (TLC) was carried out on Merck TLC silica gel 60 glass plates. Manual preparative flash column chromatography (CC) was performed using Merck silica gel 60 (particle size 0.040–0.063 mm, 230–400 mesh ASTM). Automated preparative CC was performed on a Buchi Reveleris Prep purification system using linear gradient elution and Buchi Reveleris silica 40 µm cartridges. Melting points of diastereoisomerically pure crystalline solids were determined on a Cole-Parmer Electrothermal IA9100 apparatus with open capillary tubes and were uncorrected. HPLC–MS analyses were performed on a Dionex UltiMate 3000 HPLC system coupled with a Thermo Scientific ISQ EC-LC (column: Thermo Scientific Accucore RP-MS, 50 × 2.1 mm, particle size 2.6 µm; gradient: water/MeCN containing 0.1% (v/v) formic acid each, 5% MeCN for 0.5 min, 5–95% MeCN over the course of 2 min, 95% MeCN for 4 min, flow rate 0.6 mL/min; UV detection at 254 nm; temperature 20 °C). NMR data were recorded on a Varian 300 MHz VNMRS, Varian 500 MHz Inova, or an Agilent 400 MHz 400-MR DD2 instruments. ^1^H NMR peaks are reported as follows: chemical shift (*δ*) in parts per million (ppm) relative to residual non-deuterated solvent and tetramethylsilane (TMS) as the internal standards, multiplicity (s = singlet, d = doublet, t = triplet, q = quartet, dd = doublet of doublets, ddd = doublet of doublets of doublets, dt = doublet of triplets, m = multiplet and bs = broad signal), coupling constant (in Hz), number of nuclei and proton assignment (if applicable; ax = axial, eq = equatorial). The dr values refer to the purified reaction products. Optical rotation analysis was performed with a Perkin Elmer 241 polarimeter using a sodium lamp (λ = 589 nm, D-line), at 20 °C. The [*α*]_D_^20^ values are reported in 10^−1^ deg cm^2^ g^−1^, the concentrations (*c*) are in g/100 mL. High resolution mass spectrometry (HRMS) analyses were carried out using a Thermo Scientific Q-Exactive apparatus using an electrospray ionization (ESI). X-ray diffraction data for **(3S, 4S, 8aS)-2u** and **(1S, 9aS)-3** were collected on the Rigaku Oxford Diffraction Gemini A Ultra diffractometer using mirror monochromated Cu Kα (λ = 1.54184 Å) radiation at room temperature.

### General procedure for U-5C-4CR

Isocyanide (1 equiv) was added to the mixture of α-amino acid (1 equiv), *N-*Boc-α-amino aldehyde (1 equiv), Sc(OTf)_3_ (0.1 equiv) in MeOH (2 mL per 1 mmol of isocyanide, degassed by passage of Ar gas for 20 min). The mixture was stirred at 60 °C overnight and the solvent was evaporated in vacuo. The residue was purified by CC to give the corresponding iminocarboxylic acids as diastereomeric mixtures (**1a–s**) that were not separated. The samples of pure diastereoisomers of **1n** and **1q** were obtained by repeated CC. Due to the dynamic processes (rotamers), line broadening in the ^13^C NMR spectra of the U-5C-4CR products **1** is observed.

#### Methyl ((3*S*)-3-((*tert*-butoxycarbonyl)amino)-1-(*tert*-butylamino)-1-oxo-4-phenylbutan-2-yl)glycinate (1a)

From glycine (45 mg, 0.602 mmol), *N*-Boc-*L*-phenylalaninal (150 mg, 0.602 mmol), *tert*-butyl isocyanide (70 μL, 0.602 mmol) and Sc(OTf)_3_ (30 mg, 0.060 mmol) in MeOH (1.2 mL). Purification by automated CC (4 g silica cartridge, gradient: cyclohexane to AcOEt, the desired products were eluted at approximately 60:40 solvent ratio), yield 64 mg (25%). White solid; ^1^H NMR (400 MHz, CDCl_3_, diastereoisomers, dr = 55:45) *δ* 7.32–7.66 (m, 3H), 7.32–7.66 (m, 7H), 7.14 (bs, 1H), 6.94 (bs, 1H), 5.26 (d, *J* = 9.7 Hz, 1H), 4.97 (d, *J* = 9.1 Hz, 1H), 4.13–3.96 (m, 3H), 3.70 (s, 3H), 3.65 (s, 3H), 3.40 (d, *J* = 17.5 Hz, 1H), 3.36–3.23 (m, 3H), 3.06–3.00 (m, 2H), 3.00–2.87 (m, 3H), 2.85–2.73 (m, 1H), 2.26 (bs, 2H), 1.37 (s, 27H), 1.33 (s, 9H); ^13^C NMR (101 MHz, CDCl_3_, diastereoisomers) *δ* 172.2, 138.2, 129.4, 129.3, 128.44, 128.42, 126.46, 126.42, 65.9, 64.6 (bs), 54.9 (bs), 53.9 (bs), 52.0, 51.8, 51.0, 50.8, 49.4, 28.74, 28.69, 28.3; HRMS (ESI +) m/z: [M + H]^+^ calcd. for C_22_H_36_N_3_O_5_ 422.2650, found 422.2654.

#### Methyl ((3S)-3-((*tert*-butoxycarbonyl)amino)-1-(*tert*-butylamino)-1-oxo-4-phenylbutan-2-yl)-*L*-alaninate (1b)

From *L*-alanine (54 mg, 0.602 mmol), *N*-Boc-*L*-phenylalaninal (150 mg, 0.602 mmol), *tert*-butyl isocyanide (70 μL, 0.602 mmol) and Sc(OTf)_3_ (30 mg, 0.060 mmol) in MeOH (1.2 mL). Purification by automated CC (12 g silica cartridge, gradient: cyclohexane to AcOEt, the desired products were eluted at approximately 40:60 solvent ratio), yield 154 mg (59%). Beige solid; ^1^H NMR (400 MHz, CDCl_3_, diastereoisomers, dr = 63:37) *δ* 7.33 (bs, 1H_major_), 7.30–7.14 (m, 5H_major_ and 5H_minor_), 7.05 (bs, 1H_minor_), 5.58 (d, *J* = 7.2 Hz, 1H_major_), 4.81 (d, *J* = 8.6 Hz, 1H_minor_), 4.10–3.93 (m, 1H_major_ and 1H_minor_), 3.66 (s, 3H_major_), 3.58 (s, 3H_minor_), 3.33 (q, *J* = 6.9 Hz, 1H_major_), 3.16 (q, *J* = 7.0 Hz, 1H_minor_), 3.07 (d, *J* = 4.1 Hz, 1H_major_), 3.04–2.93 (m, 2H_minor_), 2.91–2.80 (m, 2H_major_ and 1H_minor_), 1.43–1.29 (m, 18H_major_ and 18H_minor_), 1.23 (d, *J* = 7.8 Hz, 3H_major_), 1.13 (d, *J* = 6.7 Hz, 3H_minor_); ^13^C NMR (101 MHz, CDCl_3_, diastereoisomers) *δ* 175.2, 174.8, 171.6, 170.8, 156.0, 155.4, 138.16, 138.11, 129.38, 129.31, 128.6, 128.4, 126.6, 126.4, 79.4, 79.2, 64.1, 63.9, 55.8, 55.7, 55.4, 54.2, 51.94, 51.89, 50.8, 38.2, 37.8, 29.7, 28.7, 28.6, 28.3, 19.3, 18.3; HRMS (ESI +) m/z: [M + H]^+^ calcd. for C_23_H_38_N_3_O_5_ 436.2806, found 436.2815.

#### Methyl ((3*S*)-3-((*tert*-butoxycarbonyl)amino)-1-(*tert*-butylamino)-1-oxo-4-phenylbutan-2-yl)-*L*-serinate (1c)

From *L*-serine (64 mg, 0.602 mmol), *N*-Boc-*L*-phenylalaninal (150 mg, 0.602 mmol), *tert*-butyl isocyanide (70 μL, 0.602 mmol) and Sc(OTf)_3_ (30 mg, 0.060 mmol) in MeOH (1.2 mL). Purification by automated CC (12 g silica cartridge, gradient: cyclohexane to AcOEt, the desired products were eluted at approximately 30:70 solvent ratio), yield 142 mg (52%). White solid; ^1^H NMR (400 MHz, CDCl_3_, diastereoisomers, dr = 59:41) *δ* 7.32–7.15 (m, 5H_major_ and 5H_minor_), 7.00 (bs, 1H_major_), 6.84 (bs, 1H_minor_), 5.33 (d, *J* = 9.3 Hz, 1H_major_), 5.01 (d, *J* = 9.0 Hz, 1H_minor_), 4.07–3.97 (m, 1H_major_ and 1H_minor_), 3.82–3.72 (m, 4H_major_ and 1H_minor_), 3.71–3.68 (m, 1H_major_ and 1H_minor_), 3.71 (s, 3H_minor_), 3.66 (bs, 3H_major_), 3.38 (t, *J* = 4.7 Hz, 1H_major_), 3.28 (t, *J* = 4.8 Hz, 1H_minor_), 3.21–3.16 (m, 1H_major_ and 1H_minor_), 2.99–2.47 (m, 4H_major_ and 4H_minor_), 1.37 (s, 18H_minor_), 1.36 (s, 9H_major_), 1.33 (s, 9H_major_); ^13^C NMR (101 MHz, CDCl_3_, diastereoisomers) *δ* 172.9, 172.8, 171.4 (bs), 171.3 (bs), 156.0, 155.6, 138.1, 138.0, 129.30, 129.29, 128.49, 128.44, 126.51, 126.45, 79.54 (bs), 79.46 (bs), 63.8 (bs), 63.6 (bs), 63.0, 62.3, 62.02 (bs), 61.95 (bs), 55.3 (bs), 54.7 (bs), 52.25, 52.19 (bs), 51.2, 51.1, 37.7 (bs), 37.5, 30.8, 28.9, 28.7, 28.6, 28.31, 28.26; HRMS (ESI +) m/z: [M + H]^+^ calcd. for C_23_H_38_N_3_O_6_ 452.2755, found 452.2751.

#### Methyl ((3*S*)-3-((*tert*-butoxycarbonyl)amino)-1-(*tert*-butylamino)-1-oxo-4-phenylbutan-2-yl)-L-leucinate (1d)

From *L*-leucine (79 mg, 0.602 mmol), *N*-Boc-*L*-phenylalaninal (150 mg, 0.602 mmol), *tert*-butyl isocyanide (70 μL, 0.602 mmol) and Sc(OTf)_3_ (30 mg, 0.060 mmol) in MeOH (1.2 mL). Purification by automated CC (12 g silica cartridge, gradient: cyclohexane to AcOEt, the desired products were eluted at approximately 60:40 solvent ratio), yield 195 mg (68%). White solid; ^1^H NMR (400 MHz, CDCl_3_, diastereoisomers, dr = 63:37) *δ* 7.35–7.15 (m, 6H_major_ and 6H_minor_), 5.64 (d, *J* = 8.5 Hz, 1H_major_), 4.69 (d, *J* = 9.2 Hz, 1H_minor_), 4.15–4.06 (m, 1H_minor_), 4.04–3.93 (m, 1H_major_), 3.67 (s, 3H_major_), 3.53 (s, 3H_minor_), 3.30 (dd, *J* = 7.4, 6.1 Hz, 1H_major_), 3.14–2.99 (m, 1H_major_ and 2H_minor_), 2.96 (d, *J* = 3.1 Hz, 1H_minor_), 2.90–2.80 (m, 2H_major_ and 1H_minor_), 1.80–1.70 (m, 1H_minor_), 1.63–1.17 (m, 21H_major_ and 20H_minor_), 0.94–0.88 (m, 6H_minor_), 0.88–0.83 (m, 6H_major_); ^13^C NMR (101 MHz, CDCl_3_, diastereoisomers) *δ* 175.5, 175.0, 171.6 (bs), 170.7 (bs), 156.0 (bs), 155.3 (bs), 138.2, 138.1, 129.5, 129.4, 128.6, 128.4, 126.5, 126.4, 79.4, 79.1, 64.5, 64.4, 59.5, 59.2, 55.7 (bs), 54.4 (bs), 51.81, 51.75 (bs), 50.8, 50.7 (bs), 42.8 (bs), 42.1, 38.4 (bs), 37.9 (bs), 29.7 (bs), 28.7, 28.6, 28.3, 24.9, 24.7, 23.1, 22.7, 22.3, 21.9; HRMS (ESI +) m/z: [M + H]^+^ calcd. for C_26_H_44_N_3_O_5_ 478.3276, found 478.3273.

#### Methyl ((3*S*)-3-((*tert*-butoxycarbonyl)amino)-1-(*tert*-butylamino)-1-oxo-4-phenylbutan-2-yl)-D-leucinate (1e)

From *D*-leucine (79 mg, 0.602 mmol), *N*-Boc-*L*-phenylalaninal (150 mg, 0.602 mmol), *tert*-butyl isocyanide (70 μL, 0.602 mmol) and Sc(OTf)_3_ (30 mg, 0.060 mmol) in MeOH (1.2 mL). Purification by automated CC (12 g silica cartridge, gradient: cyclohexane to AcOEt, the desired products were eluted at approximately 60:40 solvent ratio), yield 177 mg (62%). White solid; ^1^H NMR (400 MHz, CDCl_3_, diastereoisomers, dr = 74:26) *δ* 7.35–7.14 (m, 5H_major_ and 5H_minor_), 7.06 (bs, 1H_minor_), 6.93 (bs, 1H_major_), 5.12 (d, *J* = 9.6 Hz, 1H_minor_), 5.02 (d, *J* = 9.0 Hz, 1H_major_), 4.02–3.89 (m, 1H_major_ and 1H_minor_), 3.68 (s, 3H_minor_), 3.63 (s, 3H_major_), 3.40–3.26 (m, 1H_minor_), 3.24–3.12 (m, 1H_major_ and 1H_minor_), 3.05 (d, *J* = 3.2 Hz, 1H_major_), 2.99–2.84 (m, 2H_major_ and 1H_minor_), 2.76 (dd, *J* = 14.0, 8.2 Hz, 1H_minor_), 2.18 (bs, 1H_major_), 1.92 (bs, 1H_minor_), 1.82–1.69 (m, 2H_minor_), 1.68–1.57 (m, 1H_major_), 1.54–1.28 (m, 19H_major_ and 18H_minor_), 0.98–0.90 (m, 6H_minor_), 0.90–0.81 (m, 6H_major_); ^13^C NMR (101 MHz, CDCl_3_, diastereoisomers) *δ* 175.7, 175.2, 171.5 (bs), 171.3 (bs), 156.1 (bs), 155.6 (bs), 138.47, 137.68, 129.65, 129.48 (bs), 129.35, 129.26, 128.57, 128.46, 128.35, 128.26, 126.53, 126.38, 126.35, 79.4 (bs), 79.3 (bs), 65.4 (bs), 64.5 (bs), 63.3, 59.3, 58.7, 55.4 (bs), 54.2 (bs), 51.9, 51.80, 51.71, 50.93, 50.65, 43.09, 42.21, 42.06, 38.36 (bs), 37.74 (bs), 29.70, 28.69, 28.66, 28.31 (bs), 24.9, 24.8, 23.12, 23.09, 22.8, 22.7, 22.1 (bs), 22.0; HRMS (ESI +) m/z: [M + H]^+^ calcd. for C_26_H_44_N_3_O_5_ 478.3276, found 478.3271.

#### Methyl ((3*S*)-3-((*tert*-butoxycarbonyl)amino)-1-(*tert*-butylamino)-1-oxo-4-phenylbutan-2-yl)-*L*-phenylalaninate (1f)

From *L*-phenylalanine (99 mg, 0.602 mmol), *N*-Boc-*L*-phenylalaninal (150 mg, 0.602 mmol), *tert*-butyl isocyanide (70 μL, 0.602 mmol) and Sc(OTf)_3_ (30 mg, 0.060 mmol) in MeOH (1.2 mL). Purification by automated CC (12 g silica cartridge, gradient: cyclohexane to AcOEt, the desired products were eluted at approximately 60:40 solvent ratio), yield 221 mg (72%). White solid; ^1^H NMR (400 MHz, CDCl_3_, diastereoisomers, dr = 70:30) *δ* 7.37–7.13 (m, 9H_major_ and 10H_minor_), 7.07–6.96 (m, 2H_major_), 6.72 (s, 1H_minor_), 5.41 (d, *J* = 8.1 Hz, 1H_major_), 4.59 (d, *J* = 9.3 Hz, 1H_minor_), 4.21–4.06 (m, 1H_minor_), 3.84 (bs, 1H_major_), 3.67 (s, 3H_major_), 3.61–3.48 (m, 1H_major_ and 3H_minor_), 3.23 (bs, 1H_minor_), 3.11–2.90 (m, 2H_major_ and 3H_minor_), 2.85 (dd, *J* = 13.8, 7.4 Hz, 1H_minor_), 2.78 (dd, *J* = 13.7, 8.2 Hz, 1H_major_), 2.66–2.57 (m, 1H_major_ and 1H_minor_), 2.53 (dd, *J* = 13.7, 6.0 Hz, 1H_major_), 1.75 (bs, 1H_major_ and 1H_minor_), 1.44 (s, 9H_minor_), 1.35 (s, 9H_major_), 1.32 (bs, 9H_major_), 1.07 (s, 9H_minor_); ^13^C NMR (101 MHz, CDCl_3_, diastereoisomers) *δ* 174.6, 1739, 171.4 (bs), 170.3, 156.0, 155.2 (bs), 138.2, 137.92, 137.86, 136.7, 129.6, 129.44, 129.37, 129.2, 128.8, 128.5, 128.37, 128.41, 127.1, 126.8, 126.4, 79.4 (bs), 79.0 (bs), 64.1, 64.0, 62.6, 62.2, 55.8, 53.8 (bs), 52.0, 51.9, 50.8, 50.2, 40.0, 39.0, 38.6, 37.2 (bs), 28.6, 28.41, 28.38, 28.3 (bs); HRMS (ESI +) m/z: [M + H]^+^ calcd. for C_29_H_42_N_3_O_5_ 512.3119, found 512.3120.

#### Methyl ((3*S*)-3-((*L*-butoxycarbonyl)amino)-1-(*L*-butylamino)-1-oxo-4-phenylbutan-2-yl)-L-tryptophanate (1g)

From *L*-tryptophan (123 mg, 0.602 mmol), *N*-Boc-*L*-phenylalaninal (150 mg, 0.602 mmol), *tert*-butyl isocyanide (70 μL, 0.602 mmol) and Sc(OTf)_3_ (30 mg, 0.060 mmol) in MeOH (1.2 mL). Purification by automated CC (12 g silica cartridge, gradient: cyclohexane to AcOEt, the desired products were eluted at approximately 60:40 solvent ratio), yield 265 mg (80%). Yellow solid; ^1^H NMR (400 MHz, CDCl_3_, diastereoisomers, dr = 64:36) *δ* 8.16 (bs, 1H_major_), 8.09 (bs, 1H_minor_), 7.62–7.54 (m, 1H_major_ and 1H_minor_), 7.44–7.07 (m, 8H_major_ and 8H_minor_), 7.06–7.01 (m, 1H_major_ and 1H_minor_), 6.81 (bs, 1H_major_), 6.74 (bs, 1H_minor_), 5.46 (d, *J* = 7.6 Hz, 1H_major_), 4.64 (d, *J* = 9.1 Hz, 1H_minor_), 4.16–4.04 (m, 1H_minor_), 3.79 (bs, 1H_major_), 3.73–3.60 (m, 4H_major_), 3.55 (s, 3H_minor_), 3.39 (bs, 1H_minor_), 3.26–3.07 (m, 1H_major_ and 1H_minor_), 3.06–2.93 (m, 2H_major_ and 1H_minor_), 2.93–2.80 (m, 2H_minor_), 2.61 (bs, 1H_minor_), 2.55–2.32 (m, 2H_major_), 1.82 (bs, 1H_major_ and 1H_minor_), 1.41 (s, 9H_minor_), 1.34 (s, 9H_major_), 1.30 (s, 9H_major_), 0.91 (s, 9H_minor_); ^13^C NMR (101 MHz, CDCl_3_, diastereoisomers) *δ* 175.0 (bs), 174.4 (bs), 171.6 (bs), 170.5 (bs), 156.0 (bs), 155.2 (bs), 138.2, 137.9, 136.4, 129.5, 129.3, 129.2, 129.0, 128.5, 128.4, 128.2, 127.3, 126.4, 126.2, 123.0 (bs), 122.9, 122.4, 122.1, 119.8, 119.6, 118.8, 118.5, 111.7 (bs), 111.5, 111.3, 110.6, 79.3 (bs), 79.0 (bs), 64.31 (bs), 64.27 (bs), 61.7 (bs), 55.7, 53.9, 52.0, 51.9, 50.8, 50.2, 38.5, 37.1 (bs), 29.8 (bs), 29.7 (bs), 28.7 (bs), 28.6, 28.39 (bs), 28.37 (bs), 28.3 (bs), 28.1; HRMS (ESI +) m/z: [M + H]^+^ calcd. for C_31_H_43_N_4_O_5_ 551.3228, found 551.3230.

#### Methyl ((3*S*)-3-((*tert*-butoxycarbonyl)amino)-1-(*tert*-butylamino)-1-oxo-4-phenylbutan-2-yl)-L-valinate (1 h)

From *L*-valine (75 mg, 0.602 mmol), *N*-Boc-*L*-phenylalaninal (150 mg, 0.602 mmol), *tert*-butyl isocyanide (70 μL, 0.602 mmol) and Sc(OTf)_3_ (30 mg, 0.060 mmol) in MeOH (1.2 mL). Crude dr = 78:22 (LC/MS). Purification by automated CC (12 g silica cartridge, gradient: cyclohexane to AcOEt, the desired products were eluted at approximately 60:40 solvent ratio), yield 204 mg (73%). White solid; ^1^H NMR (400 MHz, CDCl_3_, diastereoisomers, dr = 73:28) *δ* 7.35 (bs, 1H_minor_), 7.32–7.14 (m, 6H_major_ and 5H_minor_), 5.61 (bs, 1H_major_), 4.61 (d, *J* = 9.3 Hz, 1H_minor_), 4.19–4.08 (m, 1H_minor_), 4.03–3.92 (m, 1H_major_), 3.65 (s, 3H_major_), 3.51 (s, 3H_minor_), 3.12–2.98 (m, 2H_major_ and 1H_minor_), 2.92 (d, *J* = 3.0 Hz, 1H_minor_), 2.90–2.79 (m, 2H_major_ and 2H_minor_), 1.97–1.80 (m, 1H_major_ and 1H_minor_), 1.44–1.28 (m, 18H_major_ and 18H_minor_), 0.94 (d, *J* = 6.8 Hz, 3H_minor_), 0.87 (d, *J* = 6.7 Hz, 3H_minor_), 0.85–0.77 (m, *J* = 7.4 Hz, 6H_major_); ^13^C NMR (101 MHz, CDCl_3_, diastereoisomers) *δ* 174.7 (bs), 174.4, 171.8 (bs), 170.5 (bs), 156.0 (bs), 155.3 (bs), 138.3, 138.2, 129.5, 129.4, 128.6, 128.3, 126.5, 126.3, 79.4 (bs), 79.1 (bs), 67.3, 66.5, 65.1, 64.8, 55.6, 54.6 (bs), 51.61, 51.57, 50.8, 50.6, 38.3, 37.9 (bs), 31.34, 31.31, 29.6 (bs), 28.8, 28.6, 28.33, 28.30, 20.0, 19.0, 18.2, 17.7 (bs); HRMS (ESI +) m/z: [M + H]^+^ calcd. for C_25_H_42_N_3_O_5_ 464.3119, found 464.3111.

#### Methyl ((3*S*)-3-((*tert*-butoxycarbonyl)amino)-1-(*tert*-butylamino)-1-oxo-4-phenylbutan-2-yl)-L-isoleucinate (1i)

From *L*-isoleucine (79 mg, 0.602 mmol), *N*-Boc-*L*-phenylalaninal (150 mg, 0.602 mmol), *tert*-butyl isocyanide (70 μL, 0.602 mmol) and Sc(OTf)_3_ (30 mg, 0.060 mmol) in MeOH (1.2 mL). Crude dr = 74:26 (LC/MS). Purification by automated CC (12 g silica cartridge, gradient: cyclohexane to AcOEt, the desired products were eluted at approximately 60:40 solvent ratio), yield 238 mg (83%). Yellow oil; ^1^H NMR (400 MHz, CDCl_3_, diastereoisomers, dr = 76:24) *δ* 7.35–7.15 (m, 6H_major_ and 6H_minor_), 5.74–5.58 (d, *J* = 6.8 Hz, 1H_major_), 4.63 (d, *J* = 9.2 Hz, 1H_minor_), 4.16–4.07 (m, 1H_minor_), 4.02–3.92 (m, 1H_major_), 3.64 (s, 3H_major_), 3.51 (s, 3H_minor_), 3.16 (d, *J* = 5.1 Hz, 1H_major_), 3.08–2.97 (m, 1H_major_ and 1H_minor_), 2.94–2.79 (m, 2H_major_ and 3H_minor_), 1.75–1.48 (m, 2H_major_ and 2H_minor_), 1.43–1.28 (m, 18H_major_ and 18H_minor_), 1.21–0.96 (1H_major_ and 1H_minor_), 0.96–0.74 (m, 6H_major_ and 6H_minor_); ^13^C NMR (101 MHz, CDCl_3_, diastereoisomers) *δ* 174.6 (bs), 174.3, 171.8 (bs), 170.6 (bs), 156.1 (bs), 155.3 (bs), 138.3, 138.2, 129.5, 129.4, 128.6, 128.3, 126.5, 126.3, 79.4 (bs), 79.1 (bs), 66.0 (bs), 65.6, 64.9, 64.8 (bs), 55.6 (bs), 54.5 (bs), 51.6, 50.8, 50.6 (bs), 38.3 (bs), 38.2 (bs), 38.0, 37.9 (bs), 29.7 (bs), 28.8, 28.6, 28.3, 25.2, 24.8 (bs), 16.4, 15.5, 11.6, 11.5; HRMS (ESI +) m/z: [M + H]^+^ calcd. for C_26_H_44_N_3_O_5_ 478.3276, found 478.3280.

#### Methyl ((3*S*)-3-((*tert*-butoxycarbonyl)amino)-1-(*tert*-butylamino)-1-oxo-4-phenylbutan-2-yl)-L-prolinate (1j)

From *L*-proline (69 mg, 0.602 mmol), *N*-Boc-*L*-phenylalaninal (150 mg, 0.602 mmol), *tert*-butyl isocyanide (70 μL, 0.602 mmol) and Sc(OTf)_3_ (30 mg, 0.060 mmol) in MeOH (1.2 mL). Crude dr = 81:19 (LC/MS). Purification by automated CC (12 g silica cartridge, gradient: cyclohexane to AcOEt, the desired products were eluted at approximately 50:50 solvent ratio), yield 194 mg (70%). Pale-yellow solid; ^1^H NMR (400 MHz, CDCl_3_, diastereoisomers, dr = 80:20) *δ* 7.32–7.12 (m, 5H_major_ and 6H_minor_), 6.84 (bs, 1H_major_), 5.55 (d, *J* = 9.8 Hz, 1H_major_), 5.22 (bs, 1H_minor_), 4.22–4.03 (m, 1H_major_ and 1H_minor_), 3.89–3.79 (m, 1H_major_ and 1H_minor_), 3.71 (s, 3H_minor_), 3.64 (s, 3H_major_), 3.37–3.19 (m, 1H_major_ and 1H_minor_), 3.05–2.86 (m, 3H_major_ and 3H_minor_), 2.78–2.60 (m, 1H_major_ and 1H_minor_), 2.19–2.01 (m, 1H_major_ and 1H_minor_), 1.98–1.67 (m, 3H_major_ and 3H_minor_), 1.37 (s, 18H_minor_), 1.36–1.33 (m, 18H_major_); ^13^C NMR (101 MHz, CDCl_3_, diastereoisomers) *δ* 176.4, 175.8 (bs), 170.7 (from HMBC), 170.2 (bs), 155.5, 155.3, 138.6 (bs), 129.4, 129.2, 128.4, 128.2, 126.3, 126.2, 79.1 (bs), 79.0 (bs), 64.9, 60.4 (bs), 52.0, 51.7, 51.2, 50.7 (bs), 50.4 (from HMBC), 50.2 (from HMBC), 41.1, 37.8, 30.7, 30.4, 29.69, 28.64, 28.60, 28.33, 28.26, 24.4, 23.7; HRMS (ESI +) m/z: [M + H]^+^ calcd. for C_25_H_40_N_3_O_5_ 462.2962, found 462.2958.

#### Methyl N-((3*S*)-3-((*tert*-butoxycarbonyl)amino)-1-(*tert*-butylamino)-1-oxo-4-phenylbutan-2-yl)-N-phenylglycinate (1k)

From *N*-phenylglycine (91 mg, 0.602 mmol), *N*-Boc-*L*-phenylalaninal (150 mg, 0.602 mmol), *tert*-butyl isocyanide (70 μL, 0.602 mmol) and Sc(OTf)_3_ (30 mg, 0.060 mmol) in MeOH (1.2 mL). Purification by automated CC (12 g silica cartridge, gradient: cyclohexane to AcOEt, the desired products were eluted at approximately 90:10 solvent ratio), yield 183 mg (61%). Beige solid; ^1^H NMR (400 MHz, CDCl_3_, diastereoisomers, dr = 80:20) *δ* 7.40 (bs, 1H_major_), 7.33–7.05 (m, 7H_major_ and 8H_minor_), 6.85–6.71 (m, 1H_major_ and 1H_minor_), 6.45–6.30 (m, 2H_major_ and 2H_minor_), 5.55 (d, *J* = 9.7 Hz, 1H_major_), 5.33 (d, *J* = 10.3 Hz, 1H_minor_), 4.33–4.27 (m, 1H_major_ and 1H_minor_), 4.23–3.98 (m, 3H_major_ and 2H_minor_), 3.97–3.87 (m, 1H_minor_), 3.78–3.74 (m, 3H_major_ and 3H_minor_), 3.18–2.93 (m, 2H_major_ and 2H_minor_), 1.43 (s, 9H_minor_), 1.38 (s, 9H_major_), 1.34–1.28 (m, 9H_major_ and 9H_minor_); ^13^C NMR (101 MHz, CDCl_3_) *δ* 172.6, 169.6, 155.3, 146.2, 138.5, 129.5, 129.3, 128.5, 126.5, 119.1, 113.4, 79.3, 63.4, 53.6, 52.4, 51.3, 50.3, 40.2, 28.5, 28.4. The signals of minor isomer are not observed in ^13^C NMR; HRMS (ESI+) m/z: [M + H]^+^ calcd. for C_28_H_40_N_3_O_5_ 498.2962, found 498.2965.

#### Methyl (2S)-1-((3S)-3-((tert-butoxycarbonyl)amino)-1-(tert-butylamino)-1-oxo-4-phenylbutan-2-yl)indoline-2-carboxylate (1l)

From (2*S*)-indoline-2-carboxylic acid (98 mg, 0.602 mmol), *N*-Boc-*L*-phenylalaninal (150 mg, 0.602 mmol), *tert*-butyl isocyanide (70 μL, 0.602 mmol) and Sc(OTf)_3_ (30 mg, 0.060 mmol) in MeOH (1.2 mL). Purification by automated CC (12 g silica cartridge, gradient: cyclohexane to AcOEt, the desired products were eluted at approximately 75:25 solvent ratio), yield 198 mg (65%). Yellow oil; ^1^H NMR (400 MHz, CDCl_3_, diastereoisomers, dr = 77:23) *δ* 7.98 (bs, 1H_minor_), 7.68 (bs, 1 H_major_), 7.31–7.15 (m, 5H_major_ and 5H_minor_), 7.07–6.91 (m, 2H_major_ and 2H_minor_), 6.75–6.65 (m, 1H_major_ and 1H_minor_), 6.24 (d, *J* = 7.9 Hz, 1H_minor_), 6.08 (d, *J* = 7.9 Hz, 1H_major_), 5.21 (d, *J* = 10.2 Hz, 1H_major_), 4.76 (d, *J* = 10.2 Hz, 1H_minor_), 4.72–4.60 (m, 1H_major_ and 1H_minor_), 4.14–4.06 (m, 1H_major_), 4.04–3.98 (m, 1H_minor_), 3.90 (d, *J* = 2.8 Hz, 1H_major_), 3.83 (bs, 1H_minor_), 3.73 (s, 3H_major_ and 3H_minor_), 3.62–3.50 (m, 1H_major_ and 1H_minor_), 3.14–2.91 (m, 3H_major_ and 3H_minor_), 1.39 (s, 9H_major_ and 9H_minor_), 1.38 (s, 9H_major_), 1.30 (s, 9H_minor_); ^13^C NMR (101 MHz, CDCl_3_, diastereoisomers) *δ* 175.5, 169.6, 155.3, 150.0, 138.0, 129.7, 129.6, 129.3, 128.5, 128.4, 128.2, 127.8, 127.1, 126.54, 126.46, 126.3, 124.2, 124.1, 119.4, 119.2, 108.1, 108.0, 79.5, 63.6 (bs), 62.8, 60.3, 59.9 (bs), 54.2 (bs), 53.1, 52.4, 52.0 (bs), 51.6 (bs), 51.3, 41.1, 35.0, 34.8 (bs), 28.5, 28.3, 28.0 (bs), 27.8 (bs); HRMS (ESI +) m/z: [M + H]^+^ calcd. for C_29_H_40_N_3_O_5_ 510.2962, found 510.2971.

#### Methyl ((3S)-1-(benzylamino)-3-((tert-butoxycarbonyl)amino)-1-oxo-4-phenylbutan-2-yl)-L-isoleucinate (1m)

From *L*-isoleucine (79 mg, 0.602 mmol), Boc-*L*-phenylalaninal (150 mg, 0.602 mmol), benzyl isocyanide (69 μL, 0.602 mmol) and Sc(OTf)_3_ (30 mg, 0.060 mmol) in MeOH (1.2 mL). Crude dr = 78:22 (LC/MS). Purification by automated CC (12 g silica cartridge, gradient: cyclohexane to AcOEt, the desired products were eluted at approximately 60:40 solvent ratio), yield 214 mg (69%). White solid; ^1^H NMR (400 MHz, CDCl_3_, diastereoisomers, dr = 61:39) *δ* 7.86 (bs, 1H_major_), 7.71 (t, *J* = 6.3 Hz, 1H_minor_), 7.40–7.11 (m, 10H_major_ and 10H_minor_), 5.66 (bs, 1H_major_), 4.68 (d, *J* = 9.2 Hz, 1H_minor_), 4.58–4.39 (m, 2H_major_ and 2H_minor_), 4.29–4.18 (m, 1H_minor_), 4.10–3.99 (m, 1H_major_), 3.58 (s, 3H_major_), 3.55 (s, 3H_minor_), 3.24 (d, *J* = 5.2 Hz, 1H_major_), 3.18 (d, *J* = 3.8 Hz, 1 H_major_), 3.13–3.04 (m, 2H_minor_), 2.96–2.81 (m, 2H_major_ and 2H_minor_), 1.86–1.48 (m, 2H_major_ and 2H_minor_), 1.39 (s, 9H_minor_), 1.37 (s, 9H_major_), 1.11–0.97 (m, 1H_major_ and 1H_minor_), 0.87–0.75 (m, 6H_major_ and 6H_minor_); ^13^C NMR (101 MHz, CDCl_3_, diastereoisomers) *δ* 174.52, 174.46, 172.3 (bs), 171.3 (bs), 156.1 (bs), 155.4 (bs), 138.4, 138.3 (bs), 138.2, 138.1, 129.5, 129.3, 128.8, 128,7, 128.6, 128.4, 127.73, 127.67, 127.6, 127.4, 126.6, 126.4, 79.6, 79.4 (bs), 66.0, 65.6, 64.6, 64.1, 55.5, 54.3 (bs), 51.6, 43.3, 43.2, 38.2 (bs), 38.0, 37.7 (bs), 29.7, 28.31, 28.28, 25.3, 25.0, 15.9, 15.6, 11.6, 11.4; HRMS (ESI +) m/z: [M + H]^+^ calcd. for C_29_H_42_N_3_O_5_ 512.3119, found 512.3121.

#### Methyl (3S)-3-((tert-butoxycarbonyl)amino)-1-((2-ethoxy-2-oxoethyl)amino)-1-oxo-4-phenylbutan-2-yl)-L-isoleucinate (1n)

From *L*-isoleucine (158 mg, 1.20 mmol), *N*-Boc-*L*-phenylalaninal (300 mg, 1.20 mmol), ethyl isocyanoacetate (141 μL, 1.20 mmol) and Sc(OTf)_3_ (60 mg, 0.12 mmol) in MeOH (2.4 mL). Purification by automated CC (12 g silica cartridge, gradient: cyclohexane to AcOEt, the desired products were eluted at approximately 50:50 solvent ratio), yield 93 mg (15%) of **(2S, 3S)-1n** (faster eluting) and 369 mg (61%) of **(2R, 3S)-1n** (slower eluting). Overall yield 462 mg (76%, dr = 80:20). **(2R,3S)-1n**: White solid; m.p.: 150–152 °C; [*α*]_D_^20^ =  − 25.2 (*c* = 0.83, CHCl_3_); ^1^H NMR (400 MHz, CDCl_3_) *δ* 7.80 (bs, 1H), 7.33–7.25 (m, 4H), 7.24–7.18 (m, 1H), 5.64 (bs, 1H), 4.24 (q, *J* = 7.1 Hz, 2H), 4.17–3.93 (m, 3H), 3.67 (s, 3H), 3.27 (t, *J* = 4.2 Hz, 1H), 3.21 (t, *J* = 4.0 Hz, 1H), 2.96 (dd, *J* = 13.9, 6.7 Hz, 1H), 2.91–2.77 (m, 1H), 1.76–1.68 (m, 1H), 1.64–1.54 (m, 1H), 1.36 (s, 9H), 1.30 (t, *J* = 7.1 Hz, 3H), 1.13–1.00 (m, 1H), 0.90–0.77 (m, 6H); ^13^C NMR (101 MHz, CDCl_3_) *δ* 174.6, 169.7, 155.4 (bs), 138.4, 129.3, 128.6, 126.5, 79.3 (bs), 65.7, 64.2, 61.4, 54.2 (bs), 51.7, 41.2, 38.0, 37.3, 28.3, 25.2, 15.7, 14.2, 11.7; HRMS (ESI+) m/z: [M + H]^+^ calcd. for C_26_H_42_N_3_O_7_ 508.3017, found 508.3013. **(2S, 3S)-1n**: White gum; [*α*]_D_^20^ =  − 37.2 (*c* = 0.83, CHCl_3_); ^1^H NMR (400 MHz, CDCl_3_) *δ* 7.71 (t, *J* = 6.2 Hz, 1H), 7.35–7.22 (m, 4H), 7.22–7.14 (m, 1H), 5.49–5.27 (m, 1H), 4.36–4.18 (m, 4H), 3.86 (dd, *J* = 18.0, 5.3 Hz, 1H), 3.53 (s, 3H), 3.14–3.09 (m, 1H), 3.06–2.90 (m, 2H), 2.80 (dd, *J* = 14.0, 7.7 Hz, 1H), 2.48 (bs, 1H), 1.75–1.61 (m, 1H), 1.51–1.34 (m, 10H), 1.30 (t, *J* = 7.1 Hz, 3H), 1.20–1.07 (m, 1H), 0.92–0.81 (m, 6H); ^13^C NMR (101 MHz, CDCl_3_) *δ* 174.5, 172.0, 170.1 (bs), 156.4, 138.2, 129.4, 128.3, 126.3, 79.4 (bs), 65.2, 63.8, 61.6, 55.2, 51.5, 41.0, 38.2, 38.1, 28.3, 24.9, 15.9, 14.2, 11.5; HRMS (ESI +) m/z: [M + H]^+^ calcd. for C_26_H_42_N_3_O_7_ 508.3017, found 508.3015.

#### Methyl ((3S)-3-((tert-butoxycarbonyl)amino)-1-((2-methoxy-2-oxoethyl)amino)-1-oxo-4-phenylbutan-2-yl)-L-tryptophanate (1o)

From *L*-tryptophan (204 mg, 1.00 mmol), *N*-Boc-*L*-phenylalaninal (249 mg, 1.00 mmol), methyl isocyanoacetate (91 μL, 1.00 mmol) and Sc(OTf)_3_ (49 mg, 0.100 mmol) in MeOH (2 mL). Purification by automated CC (12 g silica cartridge, gradient: cyclohexane to AcOEt, the desired products were eluted at approximately 65:35 solvent ratio), yield 345 mg (61%). Yellow solid; ^1^H NMR (400 MHz, CDCl_3_, diastereoisomers, dr = 61:39) *δ* 8.32 (bs, 1H_major_ and 1H_minor_), 7.99 (bs, 1H_major_), 7.65–7.57 (m, 1H_major_ and 1H_minor_), 7.38–7.30 (m, 1H_major_ and 1H_minor_), 7.26–7.06 (m, 6H_major_ and 7H_minor_), 7.04 (s, 1H_major_), 7.01–6.94 (m, 1H_minor_), 6.74 (bs, 1H_major_ and 1H_minor_), 5.46 (d, *J* = 7.8 Hz, 1H_major_), 5.20 (d, *J* = 9.6 Hz, 1H_minor_), 4.30–4.20 (m, 1H_minor_), 4.09–3.91 (m, 1H_major_ and 2H_minor_), 3.80–3.74 (m, 4H_major_, 3.71–3.68 (bs, 3H_major_), 3.65 (s, 3H_minor_), 3.58 (s, 3H_minor_), 3.34 (dd, *J* = 9.9, 3.0 Hz, 1H_minor_), 3.28–3.15 (m, 2H_major_ and 2H_minor_), 3.02–2.91 (m, 1H_major_ and 1H_minor_), 2.86 (dd, *J* = 14.3, 10.0 Hz, 1H_major_), 2.75 (dd, *J* = 14.0, 8.1 Hz, 1H_minor_), 2.61 (dd, *J* = 17.8, 5.1 Hz, 1H_major_), 2.52–2.31 (m, 2H_major_ and 1H_minor_), 1.43 (s, 9H_minor_), 1.28 (s, 9H_minor_); ^13^C NMR (101 MHz, CDCl_3_, diastereoisomers) *δ* 174.7, 174.5, 173.1 (bs), 171.7, 170.2, 170.1, 156.0 (bs), 155.2 (bs), 138.2 (bs), 137.8, 136.4 (bs), 136.2, 129.4, 129.2, 128.3, 128.2, 127.2 (bs), 127.0 (bs), 126.3, 126.2, 123.4 (bs), 123.1 (bs), 122.4 (bs), 122.2 (bs), 119.9 (bs), 119.7 (bs), 119.0 (bs), 118.5, 112.1 (bs), 111.6 (bs), 111.2, 110.6, 79.3 (bs), 79.1 (bs), 63.5 (bs), 63.3, 61.5 (bs), 61.0 (bs), 55.2, 53.2 (bs), 52.3, 52.20, 52.18, 52.0 (bs), 40.9, 40.0, 38.0 (bs), 36.5 (bs), 29.8 (bs), 28.7 (bs), 28.4 (bs), 28.2 (bs); HRMS (ESI +) m/z: [M + H]^+^ calcd. for C_30_H_39_N_4_O_7_ 567.2813, found 567.2814.

#### Ethyl ((3S)-3-((tert-butoxycarbonyl)amino)-2-((2-methoxy-2-oxoethyl)amino)-4-phenylbutanoyl)glycinate (1p)

From glycine (45 mg, 0.602 mmol), *N*-Boc-*L*-phenylalaninal (150 mg, 0.602 mmol) and ethyl isocyanoacetate (71 μL, 0.602 mmol) and Sc(OTf)_3_ (30 mg, 0.060 mmol) in MeOH (1.2 mL). Crude dr = 75:25 (LC/MS). Purification by automated CC (12 g silica cartridge, gradient: cyclohexane to AcOEt, the desired products were eluted at approximately 60:40 solvent ratio), yield 103 mg (38%). White solid; ^1^H NMR (400 MHz, CDCl_3_, diastereoisomers, dr = 75:25) *δ* 7.78–7.65 (m, 1H_major_ and 1H_minor_), 7.34–7.11 (m, 5H_major_ and 5H_minor_), 5.35 (d, *J* = 9.7 Hz, 1H_major_), 5.22 (d, *J* = 9.3 Hz, 1H_minor_), 4.30–3.85 (m, 5H_major_ and 5H_minor_), 3.69 (s, 3H_minor_), 3.63 (s, 3H_major_), 3.49 (d, *J* = 17.5 Hz, 1H_minor_), 3.42–3.34 (m, 1H_major_ and 1H_minor_), 3.31 (d, *J* = 17.6 Hz, 1H_major_), 3.26–3.21 (m, 1H_major_ and 1H_minor_), 3.06–2.92 (m, 1H_major_ and 1H_minor_), 2.91–2.75 (m, 1H_major_ and 1H_minor_), 1.36 (s, 9H_major_), 1.32 (s, 9H_minor_), 1.31–1.22 (m, 3H_major_ and 3H_minor_); ^13^C NMR (101 MHz, CDCl_3_, diastereoisomers) *δ* 172.4, 172.2, 172.1 (bs), 171.6 (bs), 170.0 (bs), 169.6 (bs), 156.4 (bs), 138.2 (bs), 137.7 (bs), 129.41, 129.37, 128.42, 128.37, 126.5, 126.4, 79.5 (bs), 65.2, 64.0 (bs), 61.6 (bs), 61.4, 57.9, 54.7 (bs), 53.6 (bs), 52.0 (bs), 51.8 (bs), 49.3, 49.2, 40.99, 40.95, 38.4 (bs), 37.5 (bs), 28.3 (bs), 14.1; HRMS (ESI +) m/z: [M + H]^+^ calcd. for C_22_H_34_N_3_O_7_ 452.2391, found 452.2391.

#### Methyl (3S)-3-((tert-butoxycarbonyl)amino)-1-(tert-butylamino)-1-oxobutan-2-yl)-L-phenylalaninate (1q)

From *L*-phenylalanine (99 mg, 0.602 mmol), *N*-Boc-*L*-alaninal (104 mg, 0.602 mmol), *tert*-butyl isocyanide (70 μL, 0.602 mmol) and Sc(OTf)_3_ (30 mg, 0.060 mmol) in MeOH (1.2 mL). Crude dr = 78:22 (LC/MS). Purification by automated CC (gradient: cyclohexane to AcOEt, the desired products were eluted at approximately 75:25 solvent ratio), yield 56 mg (21%) of **(2R, 3S)-1q** (faster eluting) and 113 mg (43%) mixture of **(2R, 3S)-1q** and **(2S, 3S)-1q**. Overall yield 169 mg (64%, dr = 70:30) **(2R, 3S)-1q**: White solid; [*α*]_D_^20^ =  − 9.6 (*c* = 0.83, CHCl_3_); ^1^H NMR (400 MHz, CDCl_3_) *δ* 7.36–7.27 (m, 3H), 7.26–7.21 (m, 1H), 7.20–7.16 (m, 2H), 5.63 (d, *J* = 7.1 Hz, 1H), 3.68 (s, 3H), 3.65–3.50 (m, 2H), 3.10 (dd, *J* = 13.7, 5.9 Hz, 1H), 2.93 (d, *J* = 3.9 Hz, 1H), 2.85 (dd, *J* = 13.7, 8.2 Hz, 1H), 1.63 (bs, 1H), 1.41 (s, 9H), 1.31 (s, 9H), 0.84 (d, *J* = 6.7 Hz, 3H); ^13^C NMR (101 MHz, CDCl_3_) *δ* 173.8, 171.2 (bs), 155.2 (bs), 136.4, 129.0, 128.8, 127.2, 79.1 (bs), 64.5 (bs), 61.8, 52.0, 50.8, 47.9 (bs), 38.8, 28.6, 28.4, 15.8 (bs); HRMS (ESI+) m/z: [M + H]^+^ calcd. for C_23_H_37_N_3_O_5_ 436.2806, found 436.2808. **(2S, 3S)-1q** (from a mixture of diastereoisomers): ^1^H NMR [400 MHz, CDCl_3_, diastereoisomers, dr_**(2R,3S)-1q**/**(2S,3S)-1q**_ = 55:45] *δ* 7.37–7.26 (m, 2H, overlapped with 2H_**(2R,3S)-1q**_), 7.25–7.14 (m, 2H, overlapped with 3H_**(2R,3S)-1q**_), 6.51 (bs, 1H), 4.71 (d, *J* = 8.3 Hz, 1H), 4.02–3.92 (m, 1H), 3.73 (s, 3H), 3.30 (dd, *J* = 10.1, 3.9 Hz, 1H), 3.02 [dd, *J* = 13.7, 3.9 Hz, 1H), 2.97–2.89 (m, 1H, overlapped with 1H_**(2R,3S)-1q**_], 2.65 (dd, *J* = 13.7, 10.1 Hz, 1H), 2.47 (bs, 1H), 1.46 (s, 9H), 1.18 (d, *J* = 6.9 Hz, 3H), 1.03 (s, 9H); ^13^C NMR (101 MHz, CDCl_3_, diastereoisomers) *δ* 175.0, 170.0, 137.8 (bs), 129.4 (bs), 128.6, 126.9, 79.3 (bs), 66.5 (bs), 62.4 (bs), 52.1 (bs), 49.5 (bs), 47.9 (bs), 40.1 (bs), 28.42, 28.41, 28.3, 18.0 (bs).

#### Methyl ((3S)-3-((tert-butoxycarbonyl)amino)-1-(tert-butylamino)-5-methyl-1-oxohexan-2-yl)-L-phenylalaninate (1r)

From *L*-phenylalanine (99 mg, 0.602 mmol), *N*-Boc-*L*-leucinal (129 mg, 0.602 mmol), *tert*-butyl isocyanide (70 μL, 0.602 mmol) and Sc(OTf)_3_ (30 mg, 0.060 mmol) in MeOH (1.2 mL). Purification by automated CC (12 g silica cartridge, gradient: cyclohexane to AcOEt, the desired products were eluted at approximately 80:20 solvent ratio), yield 221 mg (77%). Pale-yellow oil; ^1^H NMR (400 MHz, CDCl_3_, diastereoisomers, dr = 75:25) *δ* 7.33–7.16 (m, 6H_major_ and 5H_minor_), 6.58 (bs, 1H_minor_), 5.33 (d, *J* = 9.6 Hz, 1H_major_), 4.36 (d, *J* = 9.5 Hz, 1H_minor_), 3.99–3.87 (m, 1H_minor_), 3.71 (s, 3H_minor_), 3.68 (s, 3H_major_), 3.62–3.52 (m, 2H_major_), 3.28 (d, *J* = 10.0 Hz, 1H_minor_), 3.08 (dd, *J* = 13.7, 5.5 Hz, 1H_major_), 3.00 (dd, *J* = 13.7, 3.9 Hz, 1H_minor_), 2.95–2.88 (m, 1H_major_ and 1H_minor_), 2.83 (dd, *J* = 13.7, 8.5 Hz, 1H_major_), 2.64 (dd, *J* = 13.6, 10.1 Hz, 1H_minor_), 1.73–1.57 (m, 1H_major_ and 1H_minor_), 1.51–1.42 (m, 1H_major_ and 10H_minor_), 1.40 (s, 9H_major_), 1.31 (s, 9H_major_), 1.21–1.08 (m, 1H_major_ and 1H_minor_), 1.02 (s, 9H_minor_), 0.91–0.86 (m, 6H_minor_), 0.78 (d, *J* = 6.7 Hz, 3H_major_), 0.68 (d, *J* = 6.5 Hz, 3H_major_); ^13^C NMR (101 MHz, CDCl_3_, diastereoisomers) *δ* 174.7, 173.9, 171.5 (bs), 170.1, 156.1, 155.5 (bs), 137.9, 136.5, 129.5, 129.1, 128.8, 128.5, 127.2, 126.8, 79.2 (bs), 78.9 (bs), 66.1, 64.7 (bs), 62.6, 62.2, 52.4, 52.00, 51.97, 50.7, 50.5 (bs), 50.2, 41.7, 40.0, 39.6 (bs), 38.8, 28.6, 28.42, 28.37, 24.9, 24.4, 23.5, 22.9, 21.9, 21.5; HRMS (ESI +) m/z: [M + H]^+^ calcd. for C_26_H_44_N_3_O_5_ 478.3276, found 478.3270.

#### Methyl ((3S)-3-((tert-butoxycarbonyl)amino)-1-(tert-butylamino)-4-(1*H*-indol-3-yl)-1-oxobutan-2-yl)glycinate (1s)

From glycine (263 mg, 3.507 mmol), *N*-Boc-*L*-tryptophanal (1.01 g, 3.507 mmol), *tert*-butyl isocyanide (397 μL, 3.507 mmol) and Sc(OTf)_3_ (173 mg, 0.351 mmol) in MeOH (7 mL). Purification by automated CC (gradient hexane: AcOEt, the desired products were eluted at approximately 75:25 solvent ratio), yield 357 mg (22%). **1s**: White solid; ^1^H NMR (400 MHz, CDCl_3_, diastereoisomers, dr = 50:50) *δ* 8.32 (bs, 1H), 8.28 (bs, 1H), 7.67 (d, *J* = 7.8 Hz, 1H), 7.63 (d, *J* = 7.8 Hz, 1H), 7.37–7.34 (m, 1H), 7.34–7.31 (m, 1H), 7.20–7.14 (m, 3H), 7.14–7.06 (m, 4H), 6.99 (bs, 1H), 5.30 (d, *J* = 9.5 Hz, 1H), 5.09 (d, *J* = 6.6 Hz, 1H), 4.26–4.13 (m, 2H), 3.65 (bs, 3H), 3.56 (s, 3H), 3.35 (d, *J* = 17.4 Hz, 1H), 3.29–3.18 (m, 3H), 3.15–3.09 (m, 3H), 3.09–2.94 (m, 3H), 2.12 (bs, 2H), 1.45–1.29 (m, 36H); ^13^C NMR (101 MHz, CDCl_3_, diastereoisomers) *δ* 172.3, 172.2, 160.5, 136.3 (bs), 136.2 (bs), 127.8 (bs), 127.6 (bs), 123.2, 122.9 (bs), 122.0, 119.44, 119.42, 119.0 (bs), 118.9 (bs), 111.8 (bs), 111.4, 111.13, 111.09, 79.4 (bs), 65.7 (bs), 64.6 (bs), 54.0 (bs), 53.0 (bs), 51.9, 51.7 (bs), 51.6, 51.0 (bs), 50.8, 49.4, 30.9, 28.9, 28.73, 28.71, 28.5 (bs), 28.3 (bs); HRMS (ESI+) m/z: [M + H]^+^ calcd. for C_24_H_37_N_4_O_5_ 461.2758, found 461.2758.

### General procedure for *N*-Boc-deprotection/cyclocondensation of the U-5C-4CR adducts

Except of the synthesis of **2n**, diastereoisomeric mixtures of **1** were used as starting materials. The U-5C-4CR adduct **1** (1.0 equiv) was dissolved in 4N solution of HCl in 1,4-dioxane (2 mL/1 mmol of the substrate). The mixture was stirred at rt until HPLC–MS analysis indicated a complete removal of the Boc group (usually between 2 and 8 h). The mixture was degassed by passage of Ar gas for 20 min and concentrated in vacuo. The residue was partitioned between CHCl_3_ (3 mL) and saturated aqueous solution of NaHCO_3_ (1 mL). The layers were separated and the aqueous phase was extracted with CHCl_3_ (1 mL). The combined organic extracts were washed with saturated aqueous solution of NaCl (1 mL), dried over anhydrous Na_2_SO_4_, filtered and concentrated in vacuo. The residue was dissolved in MeOH (3 mL/1 mmol of the starting U-5C-4CR adduct) or toluene (for the cyclization reaction of **1p**), followed by the addition of TEA (3.0 equiv). The mixture was heated in a sealed tube at 70 °C until HPLC–MS analysis showed full conversion of the deprotected U-5C-4CR adduct to its cyclic derivative (usually between 4 h and 7 days). The mixture was concentrated in vacuo and the residue was purified by automated CC to give the corresponding 2-oxopiperazines **2**. The products were mainly obtained as diastereomeric mixtures. In some instances, the samples of pure diastereoisomers were obtained by repeated CC (**(2R, 3S, 6S)-2d**, **(2R, 3S, 6S)-2i**, **(2S, 3S, 6S)-2i**, **(2R, 3S)-2k**, **(2R, 3S, 6S)-2o**, **(2S, 3S, 6S)-2o**) or recrystallization (**(2R, 3S)-2p**).

#### (3S, 6S)-3-benzyl-*N*-(tert-butyl)-6-(hydroxymethyl)-5-oxopiperazine-2-carboxamide (2c)

From **1c** (135 mg, 0.299 mmol), TEA (125 μL, 0.897 mmol) and MeOH (0.9 mL). Reaction time 2 days. Purified by automated CC (12 g silica cartridge, gradient: cyclohexane to AcOEt, the desired products were eluted at approximately 20:80 solvent ratio), yield 50 mg (52%) of **2c**. For compound characterization refer to protocol for synthesis of **2c** without purification of intermediate **1c**.

#### (3S, 6S)-3-benzyl-N-(tert-butyl)-6-isobutyl-5-oxopiperazine-2-carboxamide (2d)

From **1d** (248 mg, 0.519 mmol), TEA (217 μL, 1.557 mmol) and MeOH (1.6 mL). Reaction time 16 h. Purified by automated CC (12 g silica cartridge, gradient: cyclohexane to AcOEt, the desired products were eluted at approximately 50:50 solvent ratio), yield 57 mg (21%) of **(2R, 3S, 6S)-2d** (faster eluting) and 58 mg (21%) of diastereomeric mixture of **(2R, 3S, 6S)-2d** and **(2S, 3S, 6S)-2d**, overall yield 115 mg (64%, dr = 55:45). **(2R, 3S, 6S)-2d**: Pale-yellow solid; m.p.: 46–50 °C; [*α*]_D_^20^ =  − 64.5 (*c* = 0.67, CHCl_3_); ^1^H NMR (400 MHz, CDCl_3_) *δ* 7.36–7.26 (m, 3H), 7.24–7.14 (m, 3H), 5.67 (bs, 1H), 4.23 (dtd, *J* = 8.8, 4.4, 2.4 Hz, 1H), 3.34 (d, *J* = 4.6 Hz, 1H), 3.23 (dd, *J* = 13.7, 4.2 Hz, 1H), 3.19–3.12 (m, 1H), 2.70 (dd, *J* = 13.7, 8.7 Hz, 1H), 1.89–1.71 (m, 3H), 1.36 (s, 9H), 1.24–1.17 (m, 1H), 0.94 (d, *J* = 6.3 Hz, 3H), 0.90 (d, *J* = 6.2 Hz, 3H); ^13^C NMR (101 MHz, CDCl_3_) *δ* 172.5, 168.4, 137.0, 129.7, 128.9, 127.1, 58.7, 56.9, 54.2, 51.1, 40.7, 37.3, 28.6, 24.6, 23.4, 21.3; HRMS (ESI+) m/z: [M + H]^+^ calcd. for C_20_H_32_N_3_O_2_ 346.2489, found 346.2486. **(2S, 3S, 6S)-2d** (from a mixture of diastereoisomers): ^1^H NMR [400 MHz, diastereoisomers, dr_**(2R,3S,6S)-2p**/**(2S,3S,6S)-2p**_ = 10:90, CDCl_3_] *δ* 7.33–7.28 (m, 2H), 7.27–7.22 (m, 1H), 7.21–7.17 (m, 2H), 6.79 (bs, 1H), 5.91 (d, *J* = 4.2 Hz, 1H), 4.11–4.04 (m, 1H), 3.68 (d, *J* = 4.4 Hz, 1H), 3.34 (dd, *J* = 10.2, 3.6 Hz, 1H), 2.91 (dd, *J* = 13.1, 3.1 Hz, 1H), 2.72 (dd, *J* = 13.2, 9.0 Hz, 1H), 1.90 (ddd, *J* = 14.0, 9.6, 3.6 Hz, 1H), 1.83–1.67 (m, 2H), 1.40 (s, 9H), 1.16 (ddd, *J* = 14.0, 10.2, 4.7 Hz, 2H), 0.99–0.87 (m, 6H); ^13^C NMR (101 MHz, CDCl_3_) *δ* 173.1, 169.3, 136.4, 129.3, 129.0, 127.2, 57.0, 52.3, 52.1, 50.9, 42.3, 39.7, 28.7, 24.5, 23.6, 21.0.

#### (3S, 6S)-3-benzyl-6-((S)-*sec*-butyl)-N-(tert-butyl)-5-oxopiperazine-2-carboxamide (2i)

From **1i** (238 mg, 0.499 mmol), TEA (209 μL, 1.497 mmol) and MeOH (1.5 mL). Reaction time 9 days. Purified by automated CC (12 g silica cartridge gradient: cyclohexane to AcOEt, the desired products were eluted at approximately 75:25 solvent ratio), yield 83 mg (48%) of **(2R, 3S, 6S)-2i** (faster eluting), 29 mg (17%) of **(2S, 3S, 6S)-2i** (slower eluting) and 23 mg (13%) of diastereomeric mixture, overall yield 135 mg (78%, dr = 71:29). **(2R, 3S, 6S)-2i**: Colorless oil; [*α*]_D_^20^ =  − 70.5 (*c* = 0.53, CHCl_3_); ^1^H NMR (400 MHz, CDCl_3_) *δ* 7.35–7.29 (m, 2H), 7.28–7.23 (m, 1H), 7.23–7.16 (m, 3H), 5.65 (d, *J* = 1.5 Hz, 1H), 4.27 (dddd, *J* = 9.0, 4.5, 3.5, 2.7 Hz, 1H), 3.39 (d, *J* = 3.6 Hz, 1H), 3.12 (dd, *J* = 13.5, 4.6 Hz, 1H), 3.04 (d, *J* = 3.4 Hz, 1H), 2.72 (dd, *J* = 13.6, 9.0 Hz, 1H), 2.30–2.15 (m, 1H), 1.46–1.37 (m, 1H), 1.35 (s, 9H), 1.03 (d, *J* = 7.0 Hz, 3H), 1.02–0.95 (m, 1H), 0.93 (t, *J* = 7.7, 6.2 Hz, 3H); ^13^C NMR (101 MHz, CDCl_3_) *δ* 172.1, 169.1, 136.4, 129.3, 129.0, 127.2, 58.8, 57.1, 51.8, 50.8, 42.7, 34.5, 28.7, 24.0, 16.4, 12.3; HRMS (ESI +) m/z: [M + H]^+^ calcd. for C_20_H_32_N_3_O_2_ 346.2489, found 346.2491. **(2S, 3S, 6S)-2i**: Colorless oil; [*α*]_D_^20^ =  − 141.0 (*c* = 0.63, CHCl_3_); ^1^H NMR (400 MHz, CDCl_3_) *δ* 7.35–7.27 (m, 2H), 7.27–7.21 (m, 1H), 7.20–7.14 (m, 2H), 6.74 (bs, 1H), 5.77 (d, *J* = 4.3 Hz, 1H), 4.05–3.94 (m, 1H), 3.68 (bs, 1H), 3.38 (d, *J* = 4.4 Hz, 1H), 2.90 (dd, *J* = 12.9, 2.9 Hz, 1H), 2.58 (dd, *J* = 12.9, 10.6 Hz, 1H), 2.31–2.17 (m, 1H), 1.53–1.43 (m, 1H), 1.40 (s, 9H), 1.23–1.14 (m, 1H), 1.06 (d, *J* = 7.0 Hz, 3H), 0.97 (t, *J* = 7.3 Hz, 3H); ^13^C NMR (101 MHz, CDCl_3_) *δ* 171.3, 168.4, 136.9, 129.6, 128.9, 127.0, 63.9, 58.8, 54.6, 51.1, 37.2, 36.0, 28.6, 24.4, 16.0, 12.5; HRMS (ESI +) m/z: [M + H]^+^ calcd. for C_20_H_32_N_3_O_2_ 346.2489, found 346.2490.

#### (3S, 8aS)-3-benzyl-N-(tert-butyl)-1-oxooctahydropyrrolo[1, 2-a]pyrazine-4-carboxamide (2j)

From **1j** (390 mg, 0.842 mmol), TEA (345 μL, 2.472 mmol) and MeOH (3 mL). Reaction time 16 h. Purification by automated CC (4 g silica cartridge, gradient hexane: AcOEt, the desired products were eluted at approximately 40:60 solvent ratio), yield 186 mg (67%, dr = 79:21). Colorless oil; ^1^H NMR (400 MHz, CDCl_3_, diastereoisomers, dr = 79:21) *δ* 7.36–7.08 (m, 5H_major_ and 6H_minor_), 6.77 (bs, 1H_major_), 5.63 (bs, m, 1H_minor_), 5.33 (bs, 1H_major_), 3.97 (dddd, *J* = 11.8, 6.9, 3.3, 1.7 Hz, m, 1H_major_), 3.78 (dddd, *J* = 10.4, 8.3, 3.8, 1.7 Hz, 1H_minor_), 3.69 (t, *J* = 7.8 Hz,1H_minor_), 3.34–3.27 (m, 2H_major_), 3.23 (t, *J* = 8.2 Hz, 1H_major_), 3.15 (ddd, *J* = 9.0, 7.7, 3.5 Hz, 1H_major_), 3.10 (ddd, *J* = 9.8, 7.5, 3.8 Hz, 1H_minor_), 3.00 (d, *J* = 8.2 Hz, 1H_minor_), 2.22–2.12 (m, 2H_minor_), 2.11–2.01 (m, 2H_major_), 2.00–1.73 (m, 2H_major_ and 3H_minor_), 1.39 (s, 9H_minor_), 1.39 (s, 9H_major_); ^13^C NMR (101 MHz, CDCl_3_, diastereoisomers) *δ* 173.4 (major), 172.4 (minor), 169.6 (minor), 168.8 (major), 136.9 (major), 136.0 (minor), 129.4, 129.14 (major), 129.12 (minor), 128.9 (minor), 127.3 (major), 67.6 (minor), 66.2 (major), 61.4 (minor), 59.6 (major), 54.8 (minor), 54.4 (minor), 53.5 (major), 51.9 (major), 51.0 (major), 50.8 (minor), 39.2 (minor), 37.1 (major), 28.8 (major), 28.7 (minor), 28.2 (minor), 24.3 (major), 23.7 (minor), 22.2 (major); HRMS (ESI+) m/z: [M + H]^+^ calcd. for C_19_H_28_N_3_O_2_ 330.2176, found 330.2171.

#### (2R, 3S)-3-benzyl-N-(tert-butyl)-5-oxo-1-phenylpiperazine-2-carboxamide (2R, 3S)-2k

From **1k** (206 mg, 0.414 mmol) and TEA (173 μL, 1.242 mmol) in MeOH (1.2 mL). Reaction time 4 h. Purification by automated CC (12 g silica cartridge gradient: cyclohexane to AcOEt, the desired products were eluted at approximately 90:10 solvent ratio), yield 90 mg (60%). Beige solid; m.p.: 216–220 °C; [*α*]_D_^20^ =  − 24.0 (*c* = 0.75, CHCl_3_); ^1^H NMR (400 MHz, CDCl_3_) *δ* 7.39–7.24 (m, 6H), 6.92 (tt, *J* = 7.3, 1.0 Hz, 1H), 6.80–6.72 (m, 2H), 6.00 (bs, 1H), 5.97 (bs, 1H), 4.12–4.05 (m, 2H), 4.02 (d, *J* = 4.0 Hz, 1H), 3.95 (d, *J* = 15.8 Hz, 1H), 3.33 (dd, *J* = 14.1, 5.3 Hz, 1H), 2.91 (dd, *J* = 14.2, 9.7 Hz, 1H), 1.30 (s, 9H); ^13^C NMR (101 MHz, CDCl_3_) *δ* 168.9, 168.2, 147.2, 136.3, 129.7, 129.2, 129.1, 127.4, 120.1, 113.9, 63.3, 54.1, 51.6, 49.5, 37.1, 28.5; HRMS (ESI +) m/z: [M + H]^+^ calcd. for C_22_H_28_N_3_O_2_ 366.2176, found 366.2172. The **(2S, 3S)-2k** epimer could not be isolated in a pure form.

#### (3S, 10aS)-3-benzyl-N-(tert-butyl)-1-oxo-1, 2, 3, 4, 10, 10a-hexahydropyrazino[1,2-a]indole-4-carboxamide (2l)

From **1l** (174 mg, 0.342 mmol) and TEA (143 μL, 1.026 mmol) in MeOH (1 mL). Reaction time 4 h. Purification by automated CC (12 g silica cartridge, gradient: cyclohexane to AcOEt, the desired products were eluted at approximately 90:10 solvent ratio), yield 56 mg (43%, dr = 83:17). Pale-yellow solid; ^1^H NMR (400 MHz, diastereoisomers, dr = 83:17, CDCl_3_) *δ* 7.41–7.31 (m, 2H_major_ and 3H_minor_), 7.30–7.25 (m, 1H_major_ and 1H_minor_), 7.23–7.16 (m, 3H_major_ and 2H_minor_), 7.14–7.12 (m, 1H_minor_), 7.12–7.07 (m, 1H_major_), 7.05 (dd, *J* = 8.3, 2.1 Hz, 1H_minor_), 6.81 (td, *J* = 7.5, 1.0 Hz, 1H_major_), 6.48 (d, *J* = 7.8 Hz, 1H_major_), 6.36 (d, *J* = 8.3 Hz, 1H_minor_), 6.31 (bs, 1H_major_), 6.19 (s, 1H_minor_), 5.65 (bs, 1H_minor_), 5.63 (s, 1H_major_), 4.36–4.25 (m, 1H_major_ and 1H_minor_), 4.17–4.07 (m, 1H_major_ and 1H_minor_), 4.00–3.94 (m, 1H_major_ and 1H_minor_), 3.47–3.40 (m, 1H_major_ and 1H_minor_), 3.39–3.30 (m, 1H_major_ and 1H_minor_), 3.20–3.08 (m, 1H_major_ and 1H_minor_), 2.54–2.43 (m, 1H_major_ and 1H_minor_), 1.34 (s, 9H_major_), 1.34 (s, 9H_minor_); ^13^C NMR (101 MHz, diastereoisomers, CDCl_3_) *δ* 172.2, 171.8, 167.6, 167.2, 147.7, 146.3, 136.5, 136.3, 130.4, 129.22, 129.18, 128.9, 128.6, 127.8, 127.5, 127.4, 125.4, 125.0, 124.8, 120.0, 108.7, 108.2, 63.0, 62.3, 62.2, 53.8, 51.5, 51.4, 36.7, 36.6, 30.3, 29.8, 29.73, 29.68, 28.5; HRMS (ESI+) m/z: [M + H]^+^ calcd. for C_23_H_28_N_3_O_2_ 378.2176, found 378.2175.

#### Methyl ((2R, 3S, 6S)-3-benzyl-6-((S)-sec-butyl)-5-oxopiperazine-2-carbonyl)glycinate (2n)

From **(2R, 3S)-1n** (205 mg, 0.404 mmol) and TEA (169 μL, 1.212 mmol) in MeOH (1 mL). Reaction time 3 days. Purification by automated CC (gradient: cyclohexane to AcOEt, the desired products were eluted at approximately 50:50 solvent ratio), yield 72 mg (49%). Yellow oil; [*α*]_D_^20^ =  − 67.2 (*c* = 0.83, CHCl_3_); ^1^H NMR (400 MHz, CDCl_3_) *δ* 7.69 [t, *J* = 5.4 Hz, 1H, –CON(*H*)–CH_2_–], 7.36–7.29 (m, 2H, H-2′, H-6′), 7.28–7.23 (m, 1H, H-4′), 7.22–7.18 (m, 2H, H-3′, H-5′), 5.74 [d, *J* = 2.3 Hz, 1H, –CON(*H*)–], 4.25 (dddd, *J* = 8.8, 7.0, 5.1, 2.7 Hz, 1H, *H*-3), 4.16 (dd, *J* = 18.3, 6.1 Hz, 1H, –C*H*′_2_–COOCH_3_), 4.00 (dd, *J* = 18.3, 5.0 Hz, 1H, –C*H*_2_–COOCH_3_), 3.76 (s, 3H, –COO*CH*_*3*_), 3.53 (d, *J* = 3.7 Hz, 1H, *H*-2), 3.26 (d, *J* = 3.6 Hz, 1H, *H*-6), 3.12 (dd, *J* = 13.6, 5.0 Hz, 1H, –C*H*_2_-Ph), 2.78 (dd, *J* = 13.6, 8.9 Hz, 1H, –C*H*′_2_-Ph), 2.25–2.13 [m, 1H, –C*H*(CH_3_)–CH_2_–], 1.94 (bs, 1H, –N*H*–), 1.52–1.38 [m, 1H, –CH(CH_3_)–C*H*′_2_–], 1.17–1.10 [m, 1H, –CH(CH_3_)–C*H*_2_–], 1.07 [d, *J* = 7.0 Hz, 3H, –CH(C*H*_3_)–CH_2_–], 0.94 (t, *J* = 7.3 Hz, 3H, –CH_2_–C*H*_3_); ^13^C NMR (101 MHz, CDCl_3_) *δ* 171.9 [–*C*ON(H)–], 170.7 [–*C*ON(H)–CH_2_–], 170.1 (–*C*OOCH_3_), 136.3 (C-1′), 129.3 (C-2′, C-6′), 129.0 (C-3′, C-4′), 127.2 (C-4′), 59.0 (C-6), 56.6 (C-2), 52.43 (–*C*OOCH_3_), 52.35 (C-3), 42.3 (–*C*H_2_-Ph), 41.1 (–*C*H_2_–COOCH_3_), 35.1 [–CH(CH_3_)–*C*H_2_–], 24.3 [–*C*H(CH_3_)–CH_2_–], 16.1 [–CH(*C*H_3_)–CH_2_–], 12.2 (–*C*H_3_); HRMS (ESI+) m/z: [M + H]^+^ calcd. for C_19_H_28_N_3_O_4_ 362.2074, found 362.2070.

#### Methyl ((3S, 6S)-6-((1H-indol-3-yl)methyl)-3-benzyl-5-oxopiperazine-2-carbonyl)glycinate (2o)

From **1o** (256 mg, 0.451 mmol) and TEA (189 μL, 1.353 mmol) in MeOH (1.4 mL). Reaction time 5 days. Purified by automated CC (12 g silica column, gradient: cyclohexane to AcOEt, the desired products were eluted at approximately 30:70 solvent ratio), yield 98 mg (50%) of **(2R, 3S, 6S)-2o** (faster eluting), 38 mg (19%) of **(2S, 3S, 6S)-2o** (slower eluting) and 14 mg (7%) of diastereomeric mixture, overall yield 150 mg (76%, dr = 70:30). **(2R, 3S, 6S)-2o**: Yellow solid; [*α*]_D_^20^ =  − 71.6 (*c* = 1.03, CHCl_3_); ^1^H NMR (400 MHz, CDCl_3_) *δ* 8.38 (bs, 1H), 7.77–7.73 (m, 1H), 7.58 (t, *J* = 5.6 Hz, 1H), 7.39 (dt, *J* = 8.0, 0.9 Hz, 1H), 7.25 (ddd, *J* = 8.1, 7.0, 1.2 Hz, 1H), 7.22–7.18 (m, 1H), 7.18–7.14 (m, 4H), 6.66–6.58 (m, 2H), 5.79 (d, *J* = 3.2 Hz, 1H), 4.17–4.08 (m, 1H), 4.03–3.90 (m, 2H), 3.82 (t, *J* = 5.0 Hz, 1H), 3.74 (s, 3H), 3.50 (dd, *J* = 14.7, 5.4 Hz, 1H), 3.29–3.20 (m, 2H), 2.29 (bs, 1H), 2.24 (dd, *J* = 13.4, 5.9 Hz, 1H), 2.10 (dd, *J* = 13.4, 9.0 Hz, 1H); ^13^C NMR (101 MHz, CDCl_3_) *δ* 171.6, 170.6, 170.2, 136.4, 136.3, 129.1, 128.9, 128.0, 127.1, 123.9, 122.6, 120.2, 119.2, 111.6, 110.9, 56.2, 55.2, 52.7, 52.5, 41.7, 41.1, 26.4; HRMS (ESI+) m/z: [M + H]^+^ calcd. for C_24_H_27_N_4_O_4_ 435.2027, found 435.2023. **(2S, 3S, 6S)-2o**: Yellow solid; m.p.: 88–91 °C; [*α*]_D_^20^ =  − 120.6 (*c* = 0.96, CHCl_3_); ^1^H NMR (400 MHz, CDCl_3_) *δ* 8.42 (bs, 1H), 7.85–7.78 (m, 1H), 7.48 (t, *J* = 5.5 Hz, 1H), 7.38 (dt, *J* = 8.1, 1.1 Hz, 1H), 7.23 (ddd, *J* = 8.1, 7.0, 1.3 Hz, 1H), 7.21–7.16 (m, 2H), 7.15–7.10 (m, 3H), 6.56–6.47 (m, 2H), 5.71 (d, *J* = 3.8 Hz, 1H), 4.05–4.01 (m, 2H), 3.87–3.78 (m, 3H), 3.77 (s, 3H), 3.62 (dd, *J* = 14.4, 4.5 Hz, 1H), 3.23 (dd, *J* = 14.5, 4.7 Hz, 1H), 2.47 (dd, *J* = 12.6, 2.1 Hz, 1H), 2.12–2.04 (m, 1H), 1.38 (dd, *J* = 12.5, 10.9 Hz, 1H); ^13^C NMR (101 MHz, CDCl_3_) *δ* 171.5, 169.9, 169.5, 136.6, 136.3, 129.3, 128.6, 127.7, 126.7, 123.8, 122.5, 120.1, 119.6, 111.4, 110.5, 58.9, 58.1, 54.8, 52.5, 40.8, 37.0, 26.7; HRMS (ESI+) m/z: [M + H]^+^ calcd. for C_24_H_27_N_4_O_4_ 435.2027, found 435.2025.

#### Ethyl ((3S)-3-benzyl-5-oxopiperazine-2-carbonyl)glycinate (2p)

From **1p** (295 mg, 0.653 mmol) and TEA (273 μL, 1.959 mmol) in MeOH (2 mL). Reaction time 2 days. Purified by repeated recrystallization from toluene, followed by manual CC of the concentrated filtrates (AcOEt/MeOH, from 99:1 to 95:5), yield 67 mg (32%) of **(2R, 3S)-2p** (crystallizes from toluene) and 62 mg (30%) of a mixture of **(2R, 3S)-2p** and **(2S, 3S)-2p**. Overall yield 129 mg (62%, dr = 78:22). **(2R, 3S)-2p**: White solid; m.p.: 150–153 °C; [*α*]_D_^20^ =  − 130.4 (*c* = 1.53, CHCl_3_); ^1^H NMR (400 MHz, CDCl_3_) *δ* 7.71 (bs, 1H), 7.32–7.26 (m, 2H), 7.25–7.18 (m, 3H), 6.24 (bs, 1H), 4.23–3.91 (m, 6H), 3.67–3.49 (m, 2H), 2.97 (dd, *J* = 13.2, 2.6 Hz, 1H), 2.93 (bs, 1H), 2.73 (dd, *J* = 13.1, 10.0 Hz, 1H), 1.27 (t, *J* = 7.1 Hz, 3H); ^13^C NMR (101 MHz, CDCl_3_) *δ* 169.6, 169.51, 169.46, 136.9, 129.6, 129.1, 127.2, 61.8, 58.6, 54.2, 49.1, 41.1, 37.2, 14.3; HRMS (ESI+) m/z: [M + H]^+^ calcd. for C_16_H_24_N_3_O_4_ 320.1605, found 320.1608. **(2S, 3S)-2p** (from a mixture of diastereoisomers): ^1^H NMR [400 MHz, diastereoisomers, dr_**(2R,3S)-2p**/**(2S,3S)-2p**_ = 55:45, CDCl_3_] *δ* 7.61 (t, *J* = 5.8 Hz, 1H), 7.35–7.17 [m, 5H, overlapped with 5H_**(2R,3S)-2p**_], 6.17 (bs, 1H), 4.27–3.92 [m, 5H, overlapped with 5H_**(2R,3S)-2p**_], 3.54–3.25 [m, 3H, overlapped with 2H_**(2R,3S)-2p**_], 3.14 (dd, *J* = 13.6, 4.7 Hz, 1H), 2.78–2.65 [m, 1H, overlapped with 1H_**(2R,3S)-2p**_], 2.34 [bs, 1H overlapped with 1H_**(2R,3S)-2p**_], 1.37–1.19 [m, 3H, overlapped with 3H_**(2R,3S)-2p**_]; ^13^C NMR (101 MHz, CDCl_3_, diastereoisomers) *δ* 170.3, 169.54, 169.49, 136.1, 129.3, 129.0, 127.2, 61.6, 57.8, 53.5, 46.2, 41.2, 41.0, 14.1.

### Synthesis of (3*S*, 6*S*)-3-benzyl-*N*-(*tert*-butyl)-6-(hydroxymethyl)-5-oxopiperazine-2-carboxamide (2c) without purification of intermediate 1c

*Tert*-butyl isocyanide (116 μL, 1.00 mmol, 1.0 equiv) was added to the mixture of *N*-Boc-*L*-phenylalaninal (249 mg, 1.00 mmol, 1.0 equiv), *L*-serine (105 mg, 1.00 mmol, 1.0 equiv) and Sc(OTf)_3_ (49 mg, 0.100 mmol, 0.1 equiv) in MeOH (2 mL, degassed by passage of Ar gas for 20 min). The mixture was stirred at 60 °C overnight. The volatiles were evaporated in vacuo and the residue was partitioned between CHCl_3_ (3 mL) and saturated aqueous solution of NaHCO_3_ (1 mL). The layers were separated and the aqueous phase was extracted with CHCl_3_ (1 mL). The combined organic extracts were washed with saturated aqueous solution of NaCl (1 mL), dried over anhydrous Na_2_SO_4_, filtered and concentrated in vacuo. The residue was dissolved in 4 N solution of HCl in dioxane (2 mL) and the resulting mixture was stirred at rt for 6 h. Ar gas was passed through for 20 min and the solvents were evaporated in vacuo. The residue was partitioned between CHCl_3_ (3 mL) and saturated aqueous solution of NaHCO_3_ (1 mL). The layers were separated and the aqueous phase was extracted with CHCl_3_ (1 mL). The combined organic extracts were washed with saturated aqueous solution of NaCl (1 mL), dried over anhydrous Na_2_SO_4_, filtered and concentrated in vacuo. The residue was dissolved in MeOH (1 mL) and TEA (417 μL, 3.00 mmol, 3.0 equiv) was added. The mixture was stirred at 70 °C for 2 days. The solvent was evaporated and the residue was purified by automated CC (12 g silica cartridge, gradient: cyclohexane to AcOEt, the desired products were eluted at approximately 20:80 solvent ratio), yield 98 mg (31%, 3 steps). White solid; ^1^H NMR (400 MHz, diastereoisomers, dr = 78:22, CDCl_3_) *δ* 7.35–7.13 (m, 5H_major_ and 5H_minor_), 7.11 (bs, 1H_major_), 6.88 (bs, 6H_minor_), 6.31 (d, *J* = 2.6 Hz, 1H_major_), 6.19 (d, *J* = 4.1 Hz, 1H_minor_), 4.26 (ddt, *J* = 8.8, 6.0, 3.4 Hz, 1H_major_), 4.17 (dd, *J* = 11.2, 2.8 Hz, 1H_minor_), 4.08 (dd, *J* = 11.2, 3.8 Hz, 1H_major_), 4.05–4.01 (m, 1H_minor_), 3.75–3.69 (m, 2H_minor_), 3.65 (dd, *J* = 11.1, 4.1 Hz, 1H_major_), 3.42 (bs, 1H_minor_), 3.36 (d, *J* = 3.5 Hz, 1H_major_), 3.29 (t, *J* = 3.5 Hz, 1H_major_), 2.27 (bs, 2H_major_ and 2H_minor_), 1.39 (s, 9H_minor_), 1.34 (s, 9H_major_); ^13^C NMR (101 MHz, CDCl_3_) *δ* 171.3 (minor), 171.1 (major), 169.0 (major), 168.2 (minor), 137.0 (minor), 136.5 (major), 129.7 (minor), 129.3 (major), 129.0 (major), 128.8 (minor), 127.2 (major), 126.9 (minor), 62.1 (minor), 61.9 (major), 60.3 (minor), 58.4 (minor), 56.7 (major), 55.7 (major), 54.7 (minor), 52.4 (major), 51.2 (minor), 51.1 (major), 41.5 (major), 37.0 (minor), 28.7 (major), 28.6 (minor); HRMS (ESI+) m/z: [M + H]^+^ calcd. for C_17_H_26_N_3_O_3_ 320.1969, found 320.1970.

### Synthesis of ((3*S*)-3-((1*H*-indol-3-yl)methyl)-5-oxopiperazine-2-carbonyl)glycinate (2t)

Ethyl isocyanoacetate (453 μL, 4.173 mmol, 1.0 equiv) was added to the mixture of *N*-Boc-*L*-tryptophanal (1.202 g, 4.173 mmol, 1.0 equiv), glycine (313 mg, 4.173 mmol, 1.0 equiv) and Sc(OTf)_3_ (205 mg, 0.417 mmol, 0.1 equiv) in MeOH (15 mL, degassed by passage of Ar gas for 20 min). The reaction mixture was stirred at 60° C overnight. The HPLC–MS analysis indicated formation of two stereoisomers in a 70:30 ratio. The volatiles were evaporated in vacuo and the residue was partitioned between CHCl_3_ (15 mL) and saturated aqueous solution of NaHCO_3_ (2 mL). The layers were separated and the aqueous phase was extracted with CHCl_3_ (2 mL). The combined organic extracts were washed with saturated aqueous solution of NaCl (2 mL), dried over anhydrous Na_2_SO_4_, filtered and concentrated in vacuo. The resulting material was dissolved in 4 N solution of HCl in 1, 4-dioxane (7.5 mL) and the mixture was stirred at rt for 2 h. The mixture was degassed by passage of Ar gas for 20 min and concentrated in vacuo. The residue was partitioned between CHCl_3_ (10 mL) and saturated aqueous solution of NaHCO_3_ (2 mL). The layers were separated and the aqueous phase was extracted with CHCl_3_ (3 mL). The combined organic extracts were washed with saturated aqueous solution of NaCl (3 mL), dried over anhydrous Na_2_SO_4_, filtered and concentrated in vacuo. The residue was dissolved in toluene (5 mL), followed by addition of TEA (462 μL, 3.318 mmol). The mixture was heated in a sealed tube at 70 °C overnight. The solvent was evaporated and the residue was stirred in AcOEt. The precipitated crystals were collected and recrystallized from AcOEt/MeOH to give 152 mg (10%) of **(2R, 3S)-2t**. The combined filtrates were concentrated in vacuo and the residue was purified by automated CC (gradient: AcOEt to AcOEt/MeOH 90:10) to give 128 mg (9%) of a mixture of **(2R, 3S)-2t** and **(2S, 3S)-2t**. Overall yield 280 mg (19%, 3 steps, dr = 75:25). **(2R, 3S)-2t**: White solid; m.p.: 138–142; [*α*]_D_^20^ =  − 79.5 (*c* = 0.67, MeOH); ^1^H NMR (500 MHz, CD_3_OD) *δ* 7.60 (dt, *J* = 7.9, 1.0 Hz, 1H, H-8′), 7.35 (dt, *J* = 8.2, 1.0 Hz, 1H, H-5′), 7.14 (s, 1H, H-2′), 7.09 (ddd, *J* = 8.2, 7.0, 1.2 Hz, 1H, H-6′), 7.01 (ddd, *J* = 7.9, 7.0, 1.0 Hz, 1H, H-7′), 4.20 (q, *J* = 7.1 Hz, 2H, –C*H*_2_CH_3_), 4.05 (dd, *J* = 8.5, 4.1 Hz, 1H, H-3), 3.99 (d, *J* = 6.0 Hz, 2H, –C*H*_2_–COOEt), 3.85 (d, *J* = 4.2 Hz, 1H, H-2), 3.42 (s, 2H, H-6), 3.09 (ddd, *J* = 14.2, 4.2, 1.0 Hz, 1H, –C*H*_2_–), 3.04 (dd, *J* = 14.3, 8.8 Hz, 1H, –C*H*′_2_–), 1.26 (t, *J* = 7.1 Hz, 3H, –CH_2_C*H*_3_); ^13^C NMR (126 MHz, CD_3_OD) *δ* 172.6 (–*C*OOEt), 172.5 (C-5), 171.1 [–*C*ON(H)–], 138.3 (C-9′), 128.7 (C-4′), 124.9 (C-2′), 122.5 (C-6′), 119.9 (C-7′), 119.7 (C-8′), 112.3 (C-5′), 111.1 (C-3′), 62.4 (–*C*H_2_CH_3_), 59.1 (C-2), 54.5 (C-3), 48.9 (C-6), 41.9 (–*C*H_2_–COOEt), 27.9 (–*C*H_2_–), 14.5 (–CH_2_*C*H_3_); HRMS (ESI+) m/z: [M + H]^+^ calcd. for C_18_H_23_N_4_O_4_ 359.1714, found 359.1710. **(2S, 3S)-2t** (from a mixture of diastereoisomers): ^1^H NMR [500 MHz, diastereoisomers, dr_**(2S,3S)-2t**/**(2R,3S)-2t**_ = 55:45, CD_3_OD] *δ* 7.64–7.57 [m, 1H, overlapped with 1H_**(2R,3S)-2t**_], 7.38–7.32 [m, 1H, overlapped with 1H_**(2R,3S)-2t**_], 7.17–7.06 [m, 2H, overlapped with 2H_**(2R,3S)-2t**_], 7.05–6.97 [m, 1H, overlapped with 1H_**(2R,3S)-2t**_], 4.23–4.12 [m, 3H, overlapped with 2H_**(2R,3S)-2t**_], 3.93 (d, *J* = 7.6 Hz, 2H), 3.52–3.39 [m, 2H, overlapped with 2H_**(2R,3S)-2t**_], 3.28 (d, *J* = 18.0 Hz, 1H), 3.21 (dd, *J* = 14.4, 6.5 Hz, 1H), 3.12–2.96 [m, 1H, overlapped with 2H_**(2R,3S)-2t**_], 1.31–1.18 [m, 3H, overlapped with 3H_**(2R,3S)-2t**_]; ^13^C NMR (126 MHz, diastereoisomers, CD_3_OD) *δ* 173.5, {172.48, 172.45, 171.17, 171.15, 138.2, 128.7, 128.7 (undistinguishable)}, 124.8, 122.7, 120.1, 119.5, 112.4, 110.7, {62.42, 62.37 (undistinguishable)}, 58.3, 54.1, 46.6, 42.1, 31.2, {14.5, 14.4 (undistinguishable)}.

### Synthesis of (3*S*, 8a*S*)-3-benzyl-*N*-(*tert*-butyl)-1, 6-dioxooctahydropyrrolo[1, 2-*a*]pyrazine-4-carboxamide (2u)

*Tert*-butyl isocyanide (70 μL, 0.602 mmol, 1.0 equiv) was added to the mixture of *N*-Boc-*L*-phenylalaninal (150 mg, 0.602 mmol, 1.0 equiv), *L*-glutamic acid (89 mg, 0.602 mmol, 1.0 equiv) and Sc(OTf)_3_ (30 mg, 0.060 mmol, 0.1 equiv) in MeOH (1.2 mL). The mixture was stirred at 60 °C overnight. The HPLC–MS analysis showed formation of a complex mixture, with both U-5C-4CR product **1u** and its lactam analog **1u′** present. The mixture was cooled (NaCl—ice bath) and 4 N solution of HCl in dioxane (0.9 mL) was added. The resulting solution was stirred at 60 °C for 4 h. After cooling the mixture to rt, Ar gas was passed through for 20 min and the solvents were evaporated in vacuo. The residue was partitioned between CHCl_3_ (3 mL) and saturated aqueous solution of NaHCO_3_ (1 mL). The layers were separated and the aqueous phase was extracted with CHCl_3_ (1 mL). The combined organic extracts were washed with saturated aqueous solution of NaCl (1 mL), dried over anhydrous Na_2_SO_4_, filtered and concentrated in vacuo. The residue was dissolved in MeOH (1.5 mL) and TEA (252 μL, 1.806 mmol, 3.0 equiv) was added. The mixture was stirred at 70 °C overnight. The solvent was evaporated and the residue was purified by manual CC (DCM/MeOH, from 99:1 to 94:6), yield 45 mg (22%) of **(3S, 4R, 8aS)-2u** (faster eluting) and 16 mg (8%) **(3S, 4S, 8aS)-2u** (slower eluting). Overall yield 61 mg (30%, 3 steps, dr = 73:27). **(3S, 4R, 8aS)-2u**: White solid; m.p.: 205–210 °C; [*α*]_D_^20^ =  − 15.0 (*c* = 0.63, CHCl_3_); ^1^H NMR (300 MHz, CDCl_3_) *δ* 7.31–7.08 (m, 5H, H-Ar), 6.31 (bs, 1H, –CON*Ht*Bu), 5.99 [d, *J* = 2.4 Hz, 1H, –CON(*H*)–], 4.28 (dddd, *J* = 8.3, 5.9, 5.1, 2.4 Hz, 1H, H-3), 4.22 (d, *J* = 5.9 Hz, 1H, H-4), 4.14 (dd, *J* = 8.3, 6.1 Hz, 1H, H-8a), 2.94 (dd, *J* = 13.8, 5.1 Hz, 1H, –C*H*_2_–), 2.57 (dd, *J* = 13.8, 8.4 Hz, 1H, –C*H*′_2_–), 2.50–2.11 (m, 4H, H-7, H′-7, H-8, H′-8), 1.23 (s, 9H, –*t*Bu); ^13^C NMR (101 MHz, CDCl_3_) *δ* 175.4 (–*C*ONR–), 170.2 [–*C*ON(H)–], 166.8 (–*C*ONH*t*Bu), 135.5 (C-Ar), 129.5 (C-Ar), 129.2 (C-Ar), 127.6 (C-Ar), 56.0 (C-8a), 55.6 (C-4), 52.3 (C-3), 51.9 [–*C*(CH_3_)], 40.6 (–*C*H_2_–), 30.0 (C-7), 28.8 [–C(*C*H_3_)], 20.3 (C-8); HRMS (ESI+) m/z: [M + H]^+^ calcd. for C_19_H_26_N_3_O_3_ 344.1969, found 344.1968. **(3S, 4S, 8aS)-2u**: White solid; m.p.: 282–283 °C; [*α*]_D_^20^ =  − 2.6 (*c* = 1.13, MeOH); ^1^H NMR (300 MHz, CD_3_OD) *δ* 7.42–7.22 (m, 5H, H–Ar), 4.43–4.35 (m, 1H, H-8a), 4.34–4.27 (m, 1H, H-3), 4.25 (d, *J* = 4.3 Hz, 1H, H-4), 3.15 (dd, *J* = 14.5, 4.5 Hz, 1H, –C*H*_2_–), 2.81 (dd, *J* = 14.5, 9.5 Hz, 1H, –C*H*′_2_–), 2.65–2.48 (m, 1H, H-8), 2.48–2.33 (m, 3H, H-7, H′-8), 2.33–2.19 (m, 1H, H′-7), 1.40 (s, 9H, –*t*Bu); ^13^C NMR (101 MHz, CD_3_OD) *δ* 175.0 (–*C*ONR–), 172.1 [–*C*ON(H)–], 167.3 (–*C*ONH*t*Bu), 136.7 (C–Ar), 128.5 (C-Ar), 128.4 (C-Ar), 126.7 (C-Ar), 57.0 (C-4), 56.6 (C-8a), 53.2 (C-3), 51.3 [–*C*(CH_3_)], 35.2 (–*C*H_2_–), 30.2 (C-7), 27.5 (-C(*C*H_3_)), 20.7 (C-8); HRMS (ESI+) m/z: [M + H]^+^ calcd. for C_19_H_26_N_3_O_3_ 344.1969, found 344.1969.

### Synthesis of (1*S*)-1-((1*H*-indol-3-yl)methyl)tetrahydro-2*H*-pyrazino[1, 2-*a*]pyrazine-3, 6, 9(4*H*)-trione (3)

Compound **(2R, 3S)-2t** (41 mg, 0.115 mmol, 1.0 equiv) was dissolved in anhydrous THF (1 mL). TBD (16 mg, 0.115 mmol, 1.0 equiv) was added and the resulting solution was stirred at rt overnight. The mixture was concentrated in vacuo and the residue was purified by automated CC (gradient DCM: MeOH from 99:1 to 90:10) to give 12 mg (34%) of **(1S, 9aS)-3** and 8 mg (22%) of a mixture of **(1S, 9aS)-3** and **(1S, 9aR)-3**. Overall yield 20 mg (56%). **(1S, 9aS)-3**: White solid; [*α*]_D_^20^ =  − 142.1 (*c* = 0.82, CDCl_3_/MeOH 1:1); ^1^H NMR (500 MHz, CD_3_OD) *δ* 7.53 (d, *J* = 7.9 Hz, 1H, H-8′), 7.33 (d, *J* = 8.1 Hz, 1H, H-5′), 7.13–7.07 (m, 1H, H-6′), 7.06 (s, 1H, H-2′), 7.01 (t, *J* = 7.5 Hz, 1H, H-7′), 4.51 (dt, *J* = 3.5, 1.7 Hz, 1H, H-9a), 4.37 (d, *J* = 18.8 Hz, 1H, H-4_ eq_), 4.16 (dt, *J* = 8.3, 3.4 Hz, 1H, H-1), 3.90 (dd, *J* = 18.0, 1.9 Hz, 1H, H-7), 3.75 (dd, *J* = 18.2, 1.6 Hz, 1H, H′-7), 3.65 (d, *J* = 18.8 Hz, 1H, H-4_ax_), 3.04 (dd, *J* = 14.4, 3.4 Hz, 1H, –C*H*_2_–), 2.95 (dd, *J* = 14.4, 8.3 Hz, 1H, –C*H*′_2_–); ^13^C NMR (126 MHz, CD_3_OD) *δ* 168.4 (C-3), 166.0 (C-9), 164.4 (C-6), 138.1 (C-9′), 128.6 (C-4′), 124.8 (C-2′), 122.6 (C-6′), 120.0 (C-7′), 119.2 (C-8′), 112.4 (H-5′), 110.0 (C-3′), 58.4 (C-9a), 54.5 (C-1), 45.8 (C-4), 45.2 (C-7), 28.0 (–*C*H_2_–); HRMS (ESI+) m/z: [M + H]^+^ calcd. for C_16_H_17_N_4_O_3_ 313.1295, found 313.1296. **(1S, 9aR)-3** (from a mixture of diastereoisomers): ^1^H NMR [400 MHz, diastereoisomers, dr_**(1S,9aS)-3/(1S,9aR)-3**_ = 61:39, CD_3_OD] *δ* 7.71–7.66 (m, 1H), 7.38–7.31 [m, 1H, overlapped with 1H_**(1S,9aS)-3**_], 7.24 (s, 1H), 7.13–7.07 [m, 1H, overlapped with 1H_**(1S,9aS)-3**_], 7.05–6.99 [m, 1H, overlapped with 1H_**(1S,9aS)-3**_], 4.73 (d, *J* = 17.6 Hz, 1H), 4.24–4.08 [m, 3H, overlapped with 1H_**(1S,9aS)-3**_], 3.90 (dd, *J* = 1.8, 0.7 Hz, 1H), 3.43 (ddd, *J* = 14.9, 3.3, 1.0 Hz, 1H), 3.37–3.32 (m, 1H), 3.28–3.21 (m, 1H); ^13^C NMR (101 MHz, diastereoisomers, CD_3_OD) *δ* 169.1, 164.5, 164.3, 129.2, 125.8, 122.6, 120.2, 119.7, 112.4, 109.0, 58.3, 55.8, 45.2, 30.0.

### Synthesis of (5*R*, 6*S*, 8a*S*)-6-benzyl-*N*-(*tert*-butyl)-8-oxohexahydro-3*H*-oxazolo[3, 4-*a*]pyrazine-5-carboxamide (4)

TEA (26 μL, 0.188 mmol, 1.5 equiv) was added to a stirred solution of **2c** (40 mg, 0.125 mmol, 1.0 equiv) and CDI (24 mg, 0.150 mmol, 1.2 equiv) in anhydrous THF (0.5 mL). The mixture was stirred at rt overnight and concentrated in vacuo. The residue was dissolved in CHCl_3_ (3 mL) and washed sequentially with 1 M aqueous solution of citric acid (1 mL), water (1 mL) and saturated aqueous solution of NaHCO_3_, dried over anhydrous Na_2_SO_4_, filtered and concentrated in vacuo. The residue was purified by automated CC (4 g silica cartridge, gradient: DCM to DCM/MeOH 95:5), yield 30 mg (70%). White solid; m.p.: 170–172 °C; [*α*]_D_^20^ = –38.0 (*c* = 1.00, CDCl_3_); ^1^H NMR (400 MHz, CDCl_3_) *δ* 7.38–7.32 (m, 2H), 7.32–7.26 (m, 1H), 7.25–7.20 (m, 2H), 6.45 (bs, 1H), 6.04 (bs, 1H), 4.75 (dd, *J* = 9.0, 4.2 Hz, 1H), 4.52 (t, *J* = 9.2 Hz, 1H), 4.39–4.31 (m, 2H), 4.05 (d, *J* = 6.9 Hz, 1H), 3.10 (dd, *J* = 13.9, 4.9 Hz, 1H), 2.67 (dd, *J* = 13.9, 9.0 Hz, 1H), 1.35 (s, 9H); ^13^C NMR (101 MHz, CDCl_3_) *δ* 168.4, 166.4, 158.2, 135.0, 129.2, 127.7, 64.1, 57.3, 53.2, 52.5, 52.0, 39.8, 28.6; HRMS (ESI+) m/z: [M + H]^+^ calcd. for C_18_H_24_N_3_O_4_ 346.1761, found 346.1762. The **(5S, 6S, 8aS)-4** epimer could not be isolated in a pure form.

## Supplementary material

The copies of ^1^H, ^13^C spectra of all compounds, the copies of 2D-NMR spectra of selected compounds. X-ray crystal data and refinement details for compounds **(3S, 4S, 8aS)-2u** and **(1S, 9aS)-3**.

## Accession codes

CCDC 2217077-2217078 contain the supplementary crystallographic data for this paper. These data can be obtained free of charge via www.ccdc.cam.ac.uk/data_request/cif, or by emailing data_request@ccdc.cam.ac.uk, or by contacting The Cambridge Crystallographic Data Centre, 12 Union Road, Cambridge CB2 1EZ, UK; fax: +44 1223 336033.

### Supplementary Information

Below is the link to the electronic supplementary material.

Supplementary file1 (PDF 9417 kb)
