# Supplementary material for: Ugi 5-center-4-component reaction of α-amino aldehydes and its application in synthesis of 2-oxopiperazines
Source: Mol Divers. 2023 Dec 17;28(1):229–48. doi: 10.1007/s11030-023-10760-1 (PMC10876754; doi:10.1007/s11030-023-10760-1)

Supplementary information

Ugi 5-center-4-component reaction of α-amino aldehydes and its application in synthesis of 2-oxopiperazines

Marta Splandesci[1], Martyna Z. Wróbel[1], Izabela D. Madura[2], Maciej Dawidowski[1,*]

[1]*Department of Drug Technology, Faculty of Pharmacy, Medical University of Warsaw, Banacha 1, 02-097 Warszawa, Poland*

[2]*Faculty of Chemistry, Warsaw University of Technology, Noakowskiego 3, 00-664 Warszawa, Poland*

[*]Corresponding Author: maciej.dawidowski@wum.edu.pl

Table of Contents

I. Single crystal X-ray diffraction data for compounds **(3*S*,4*S*,8a*S*)-2u** and **(1*S*,9a*S*)-3** **S2–S5**

II. Copies of ^{1}H and ^{13}C NMR spectra **S6–S105**

I. Single crystal X-ray diffraction data for compounds (3*S*,4*S*,8a*S*)-2u and (1*S*,9a*S*)-3

Single crystals suitable for X-ray diffraction studies were selected under a polarizing microscope, mounted on the capillary, and transferred to the diffractometer. Diffraction data for (3*S*,4*S*,8a*S*)-3-benzyl-*N*-(*tert*-butyl)-1,6-dioxooctahydropyrrolo[1,2-*a*]pyrazine-4-carboxamide **(3*S*,4*S*,8a*S*)-2u** and (1*S*,9a*S*)-1-((1*H*-indol-3-yl)methyl)tetrahydro-2*H*-pyrazino[1,2-*a*]pyrazine-3,6,9(4*H*)-trione **(1*S*,9a*S*)-3** were collected on the Rigaku Oxford Diffraction Gemini A Ultra diffractometer using mirror monochromated CuKα (λ = 1.54184 Å) radiation at room temperature. Cell refinement, data collection, data reduction and analysis were performed with the CrysAlisPro 1.171.42.51a software.[1] Empirical absorption correction using spherical harmonics implemented in SCALE3 ABSPACK scaling algorithm was used in both cases. Using Olex2,[2] the structures were solved with the SHELXT[3] structure solution program using Intrinsic Phasing and refined with the SHELXL[4] refinement package using Least Squares minimization. All non-hydrogen atoms were refined with anisotropic temperature factors. The H-atoms were placed in calculated positions riding on their parent atom with fixed isotropic thermal parameters. In the case of **(3*S*,4*S*,8a*S*)-2u** the disordered phenyl ring was modeled in two positions with freely refined occupancy factors, which converged to 0.74(4) and 0.26(4) for major and minor positions, respectively. In the case of **(1*S*,9a*S*)-3**, the solvent molecule was identified to reside on the symmetry element (two-fold axis) and was refined with the appropriate fixed coordinates. At the final refinement stage, Flack's[5] and Hooft's[6] parameters converged to 0.2(2) and 0.19(15) in the case of **(3*S*,4*S*,8a*S*)-2u**, and to -0.07(13) and-0.06(13) in the case of **(1*S*,9a*S*)-3**. Nevertheless, the absolute structure was established based on 3*S* and 1*S* configurations in **(3*S*,4*S*,8a*S*)-2u** and **(1*S*,9a*S*)-3**, respectively. Programs used for graphics preparation: Ortep-3 for Windows v. 2014/1[7] and Mercury 2022.2.0.[8]

Table S2. Crystal data and structure refinement

Identification code	**(3*S*,4*S*,8a*S*)-2u**	**(1*S*,9a*S*)-3**
Empirical formula	$C_{19}H_{25}N_3O_3$	$C_{16}H_{16}N_4O_3 \cdot 0.5\ CH_3OH$
Formula weight	343.42	328.35
Temperature/K	293.15	
Crystal system	monoclinic	orthorhombic
Space group	$P2_1$	$C222_1$
a/Å	9.5243(4)	11.1900(2)
b/Å	10.4289(4)	13.6907(3)
c/Å	9.9120(4)	20.2836(4)
α/°	90	90
β/°	103.710(4)	90
γ/°	90	90
Volume/Å3	956.49(7)	3107.43(11)
Z	2	8
ρcalcg/cm3	1.192	1.404
μ/mm-1	0.660	0.837
F(000)	368.0	1384.0
Crystal size/mm3	0.42 × 0.32 × 0.14	0.35 × 0.30 × 0.22
Radiation	Cu Kα (λ = 1.54184 Å)	
2θ range for data collection/°	9.184 to 134.322	8.718 to 134.366
Index ranges	-11 ≤ h ≤ 11, -11 ≤ k ≤ 12, -11 ≤ l ≤ 11	-8 ≤ h ≤ 13, -16 ≤ k ≤ 16, -21 ≤ l ≤ 24
Reflections collected	9630	10080
Independent reflections	3360 [R_{int} = 0.0479]	2796 [R_{int} = 0.0417]
Data/restraints/parameters	3360/94/285	2796/0/220
Goodness-of-fit on F^2	1.093	1.066
Final *R* indexes [*I*>=2σ (I)]	R_1 = 0.0449, wR_2 = 0.0965	$R_1$1 = 0.0365, wR_2 = 0.0911
Final *R* indexes [all data]	R_1 = 0.0661, wR_2 = 0.1053	R_1 = 0.0404, wR_2 = 0.0938
Largest diff. peak/hole / e $Å^{-3}$	0.10/–0.09	0.18/–0.23
Flack parameter	0.2(2)	–0.07(13)
Hooft	0.19(15)	–0.06(13)
CCDC No	2217077	2217078

The molecules of **(3*S*,4*S*,8a*S*)-2u** crystallize in the $P2_1$ space symmetry group of the monoclinic system. The fused ring system is composed of five- and six- membered rings. The five-membered ring shows envelope conformation at the C8 atom with the ring puckering parameters[9] Q and φ equal to 0.307(6) Å and 288.2(8)°, respectively. The 6-membered ring with puckering amplitude (Q) of 0.596(4) Å, θ and φ angles of 98.9(4)° and 98.9(3)°, respectively, shows twisted boat conformation.

In the crystal, the molecules interact via two N-H…O hydrogen bonds forming a 1D tape propagating along [010] direction. These supramolecular entities are further joined by numerous C-H…O and C-H…p interactions forming a 3D structure with a rectangular crosssection (Figure S1).

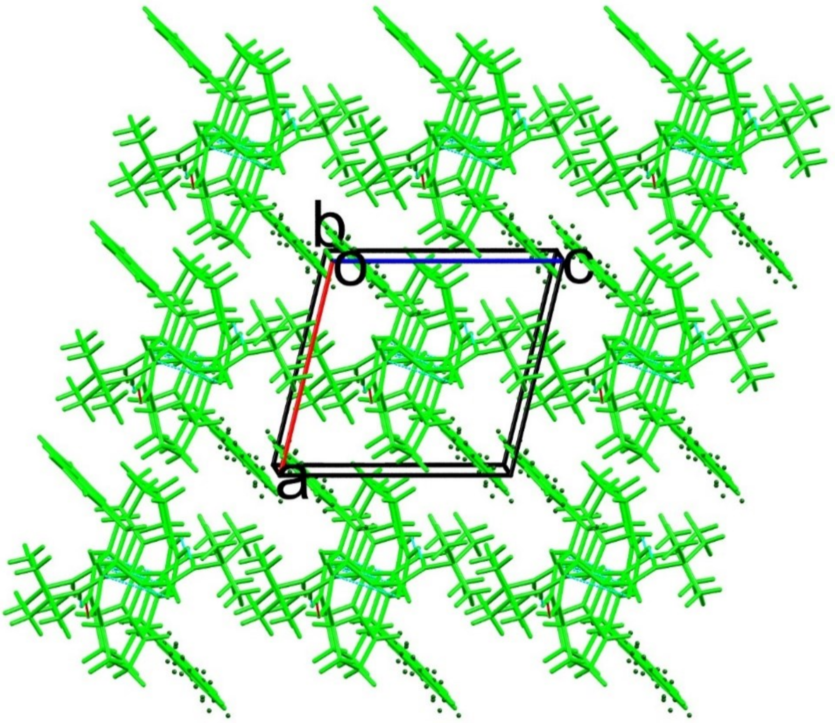

Figure S1. The packing diagram of **(3*S*,4*S*,8a*S*)-2u**. The molecules are shown in green and the dark dots represent the position of the disordered phenyl ring. View along [010] direction.

The molecules of **(1*S*,9a*S*)-3** crystallize in the $C222_1$ space symmetry group of the orthorhombic system with one methanol molecule per two molecules of **3**. The solvent resides on a 2-fold symmetry axis and is weakly bounded to the molecule of **3**. The main molecule possesses a fused ring system where both 6-membered rings are puckered. The 2-oxopiperazine ring shows an envelope conformation at the C9A atom with the puckering parameters equal to Q = 0.503(3) Å, θ = 52.5(3)°, φ = 316.5(4)°. The second ring is an almost flat boat, with the puckering amplitude being only 0.187(3) Å and angles θ and φ of 82.1(9)° and 307.1(8)°, respectively. The molecules interact *via* relatively short N-H…O hydrogen bonds forming a double layer structure oriented perpendicularly to [001] direction. Each layer contains a void accessible for the solvent molecule (Figure S2).

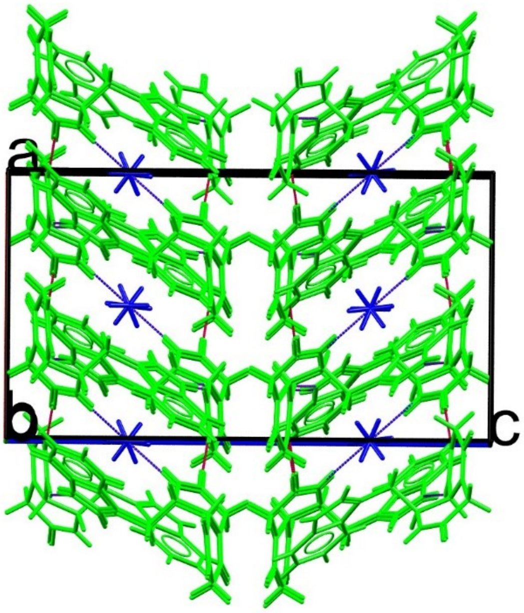

Figure S2. The packing diagram of **(1*S*,9a*S*)-3**. The molecules are shown in green while the disordered solvent molecule in blue. View along [010] direction.

References

(1) *CrysAlisPro 1.171.42.51a program (Rigaku Oxford Diffraction, 2022)*.

(2) Dolomanov, O. V.; Bourhis, L. J.; Gildea, R. J.; Howard, J. A. K.; Puschmann, H. OLEX2: A Complete Structure Solution, Refinement and Analysis Program. *J. Appl. Crystallogr.* **2009**, *42* (2), 339–341. https://doi.org/10.1107/S0021889808042726.

(3) Sheldrick, G. M. SHELXT - Integrated Space-Group and Crystal-Structure Determination. *Acta Crystallogr. Sect. A Found. Crystallogr.* **2015**, *71* (1), 3–8. https://doi.org/10.1107/S2053273314026370.

(4) Sheldrick, G. M. Crystal Structure Refinement with SHELXL. *Acta Crystallogr. Sect. C Struct. Chem.* **2015**, *71* (Md), 3–8. https://doi.org/10.1107/S2053229614024218.

(5) Parsons, S.; Flack, H. D.; Wagner, T. Use of Intensity Quotients and Differences in Absolute Structure Refinement. *Acta Crystallogr. Sect. B Struct. Sci. Cryst. Eng. Mater.* **2013**, *69* (3), 249–259. https://doi.org/10.1107/S2052519213010014.

(6) Hooft, R. W. W.; Straver, L. H.; Spek, A. L. Determination of Absolute Structure Using Bayesian Statistics on Bijvoet Differences. *J. Appl. Crystallogr.* **2008**, *41* (1), 96–103. https://doi.org/10.1107/S0021889807059870.

(7) Farrugia, L. J. WinGX and ORTEP for Windows: An Update. *J. Appl. Crystallogr.* **2012**, *45* (4), 849–854. https://doi.org/10.1107/S0021889812029111.

(8) MacRae, C. F.; Sovago, I.; Cottrell, S. J.; Galek, P. T. A.; McCabe, P.; Pidcock, E.; Platings, M.; Shields, G. P.; Stevens, J. S.; Towler, M.; Wood, P. A. Mercury 4.0: From Visualization to Analysis, Design and Prediction. *J. Appl. Crystallogr.* **2020**, *53*, 226–235. https://doi.org/10.1107/S1600576719014092.

(9) Cremer, D.; Pople, J. A. A General Definition of Ring Puckering Coordinates. *J. Am. Chem. Soc.* **1975**, *97* (6), 1354–1358. https://doi.org/10.1021/ja00839a011

II. Copies of ^{1}H and ^{13}C NMR spectra*

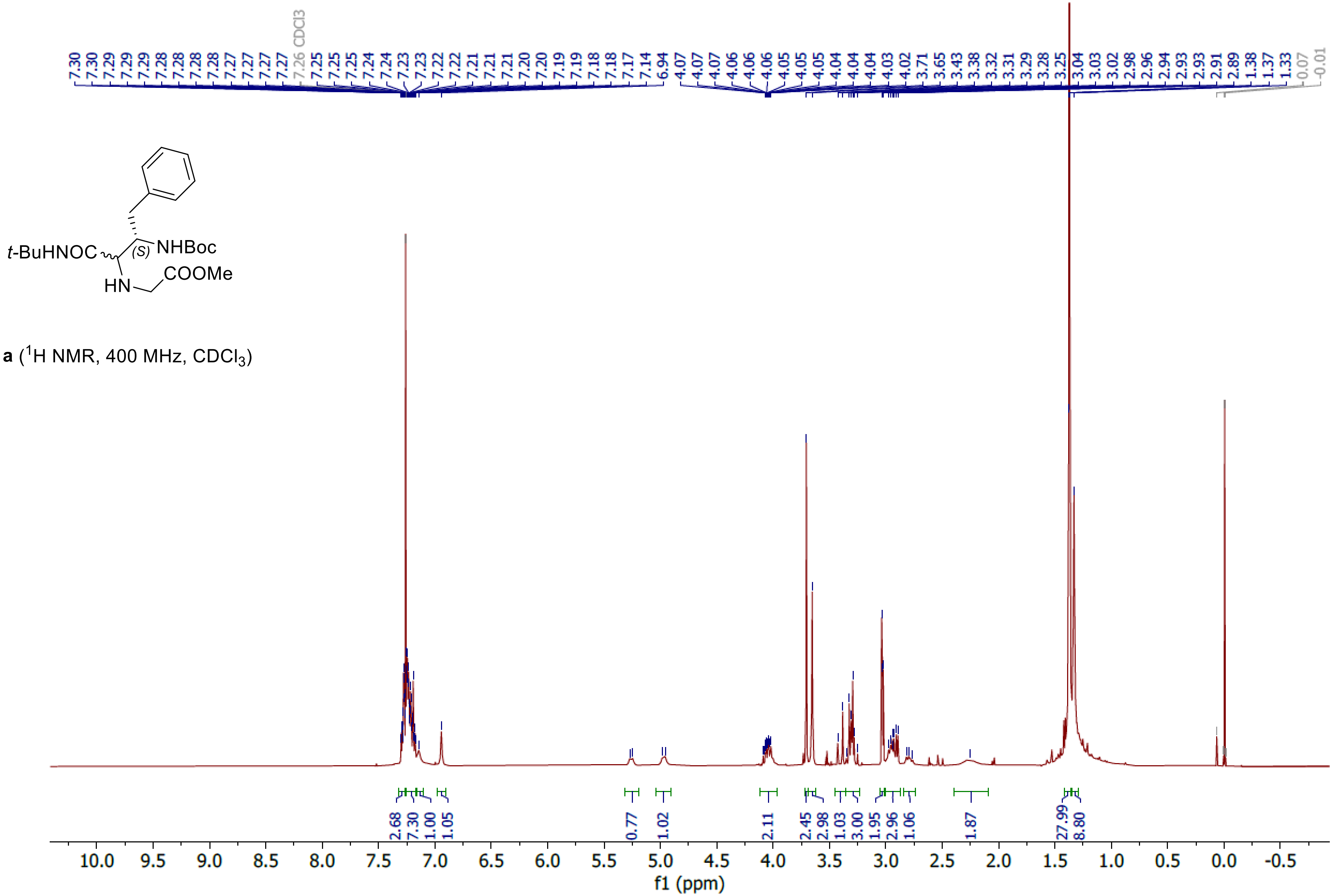

1a (^{1}H NMR, 400 MHz, $CDCl_3$)

* Line broadening in the ^{13}C NMR spectra is observed due to the dynamic processes.

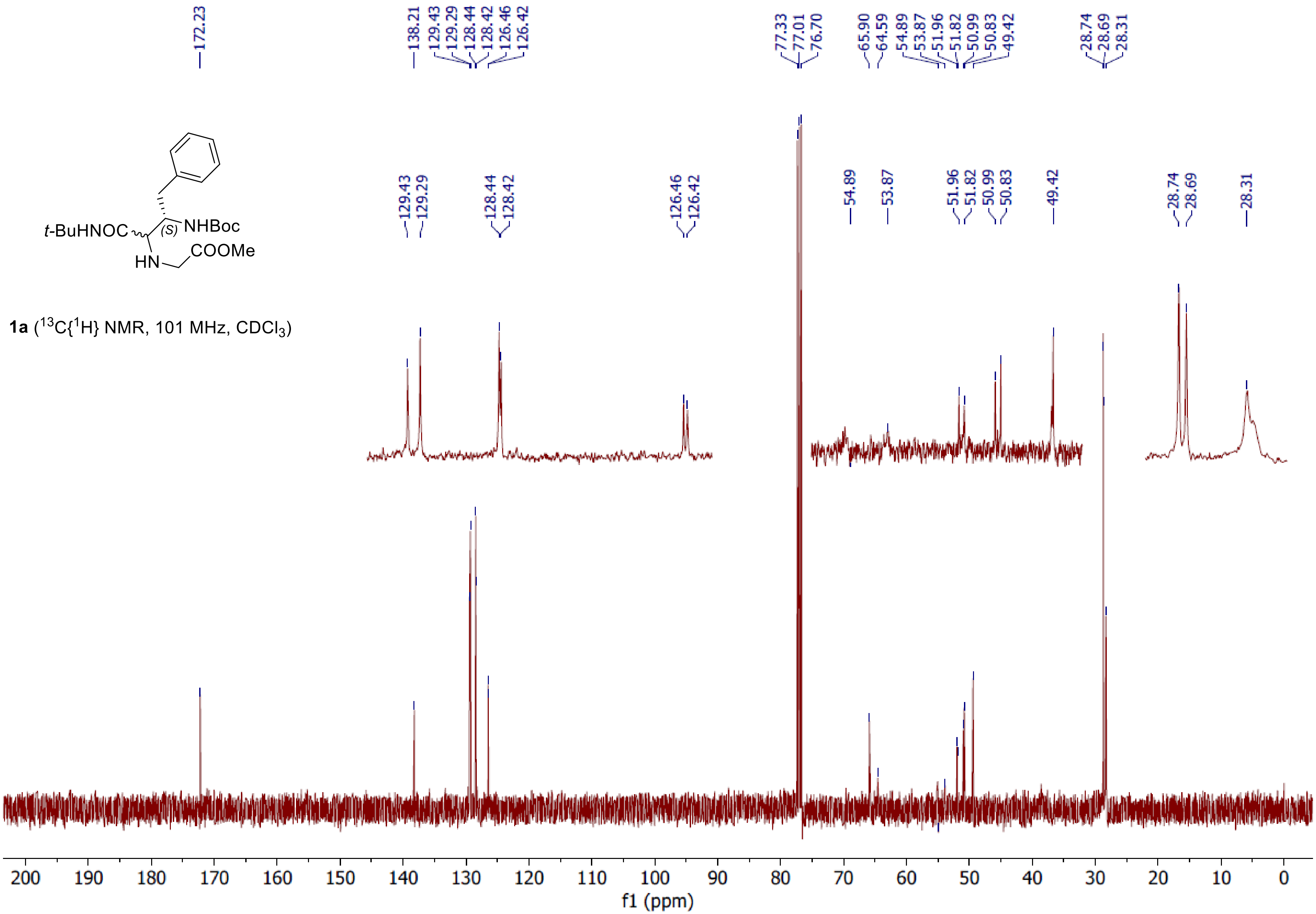

1a ($^{13}C\{^{1}H\}$ NMR, 101 MHz, $CDCl_3$)

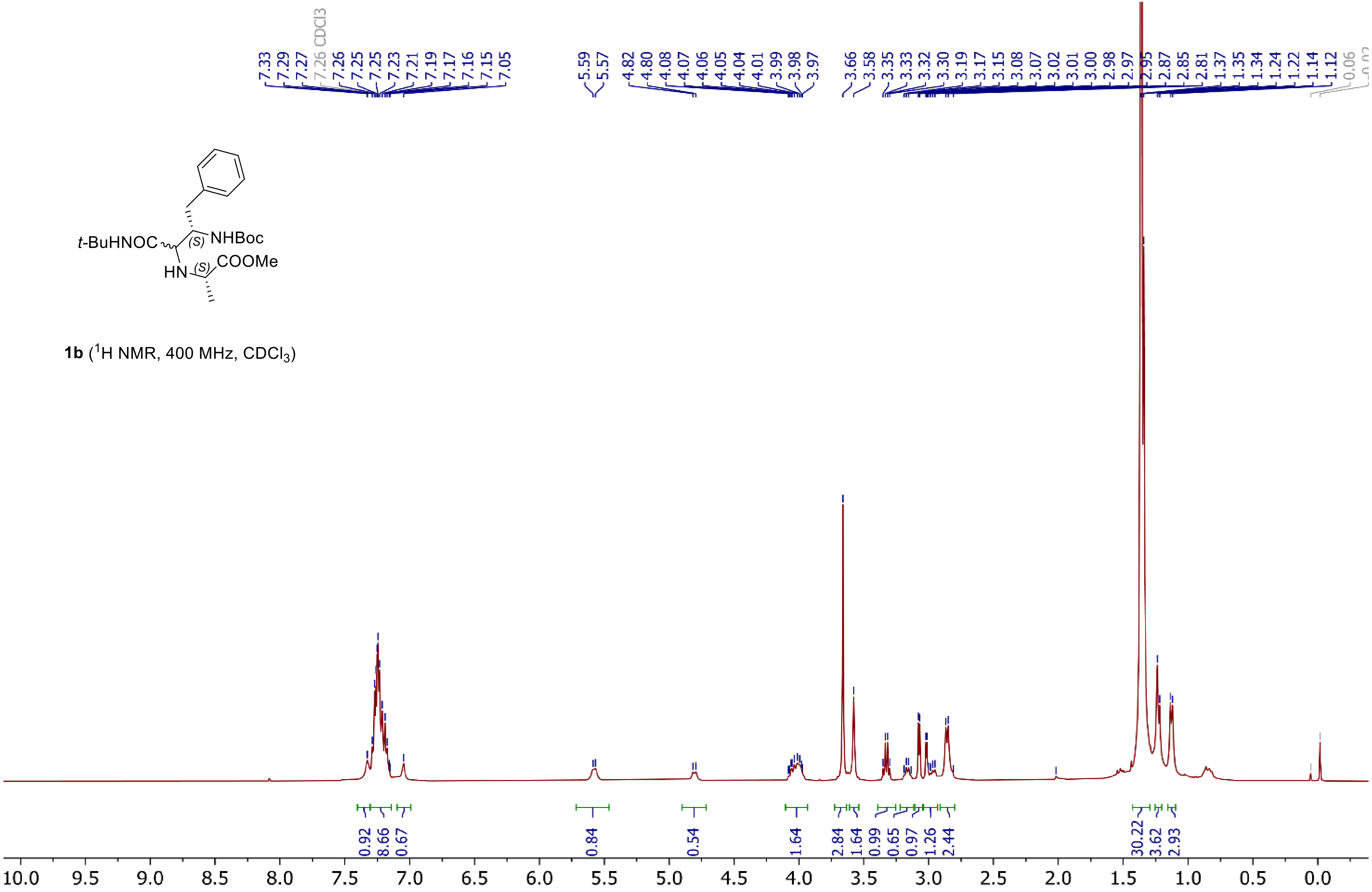

1b (^{1}H NMR, 400 MHz, $CDCl_3$)

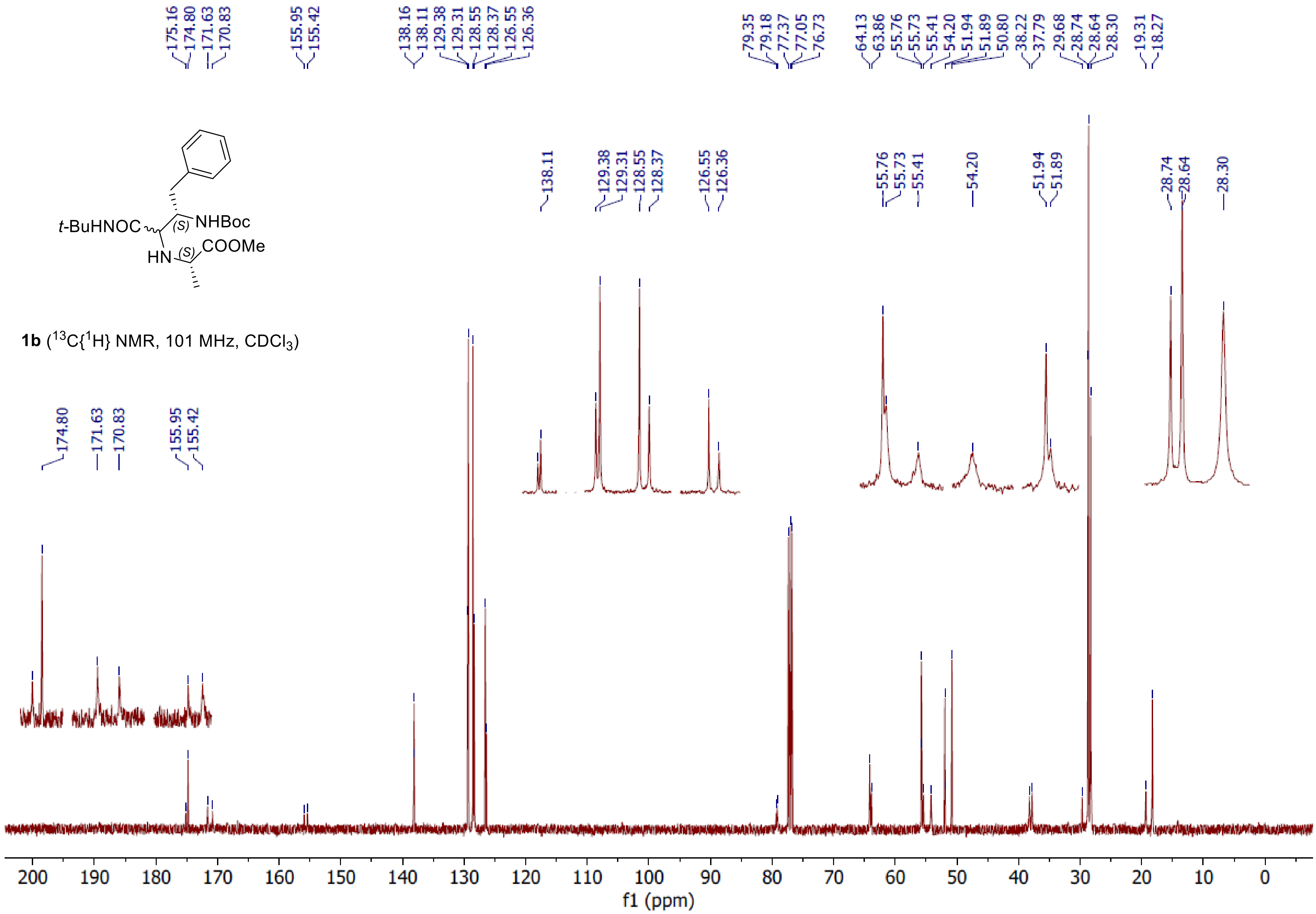

1b ($^{13}C\{^{1}H\}$ NMR, 101 MHz, $CDCl_3$)

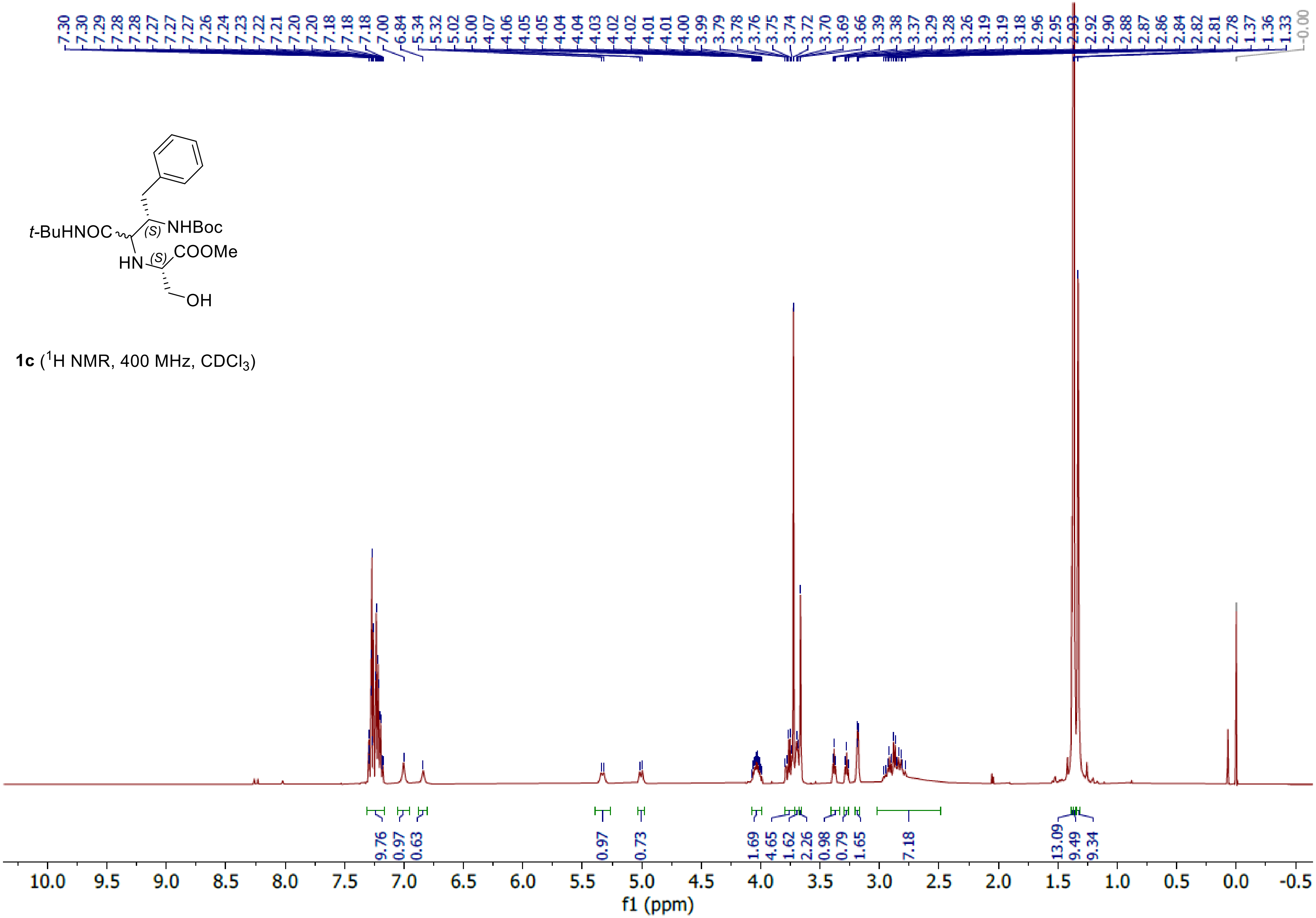

1c (^{1}H NMR, 400 MHz, $CDCl_3$)

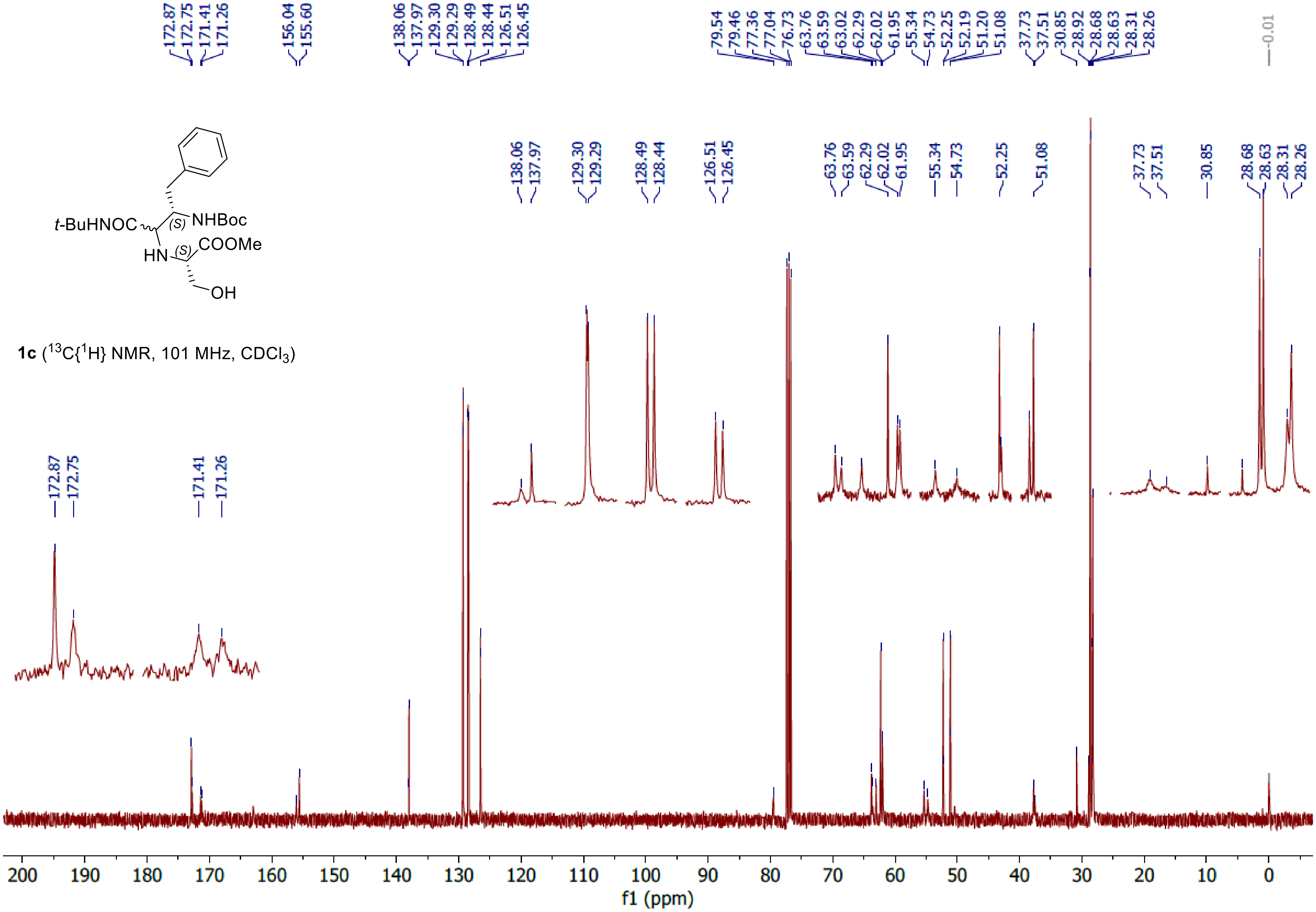

1c ($^{13}C\{^{1}H\}$ NMR, 101 MHz, $CDCl_3$)

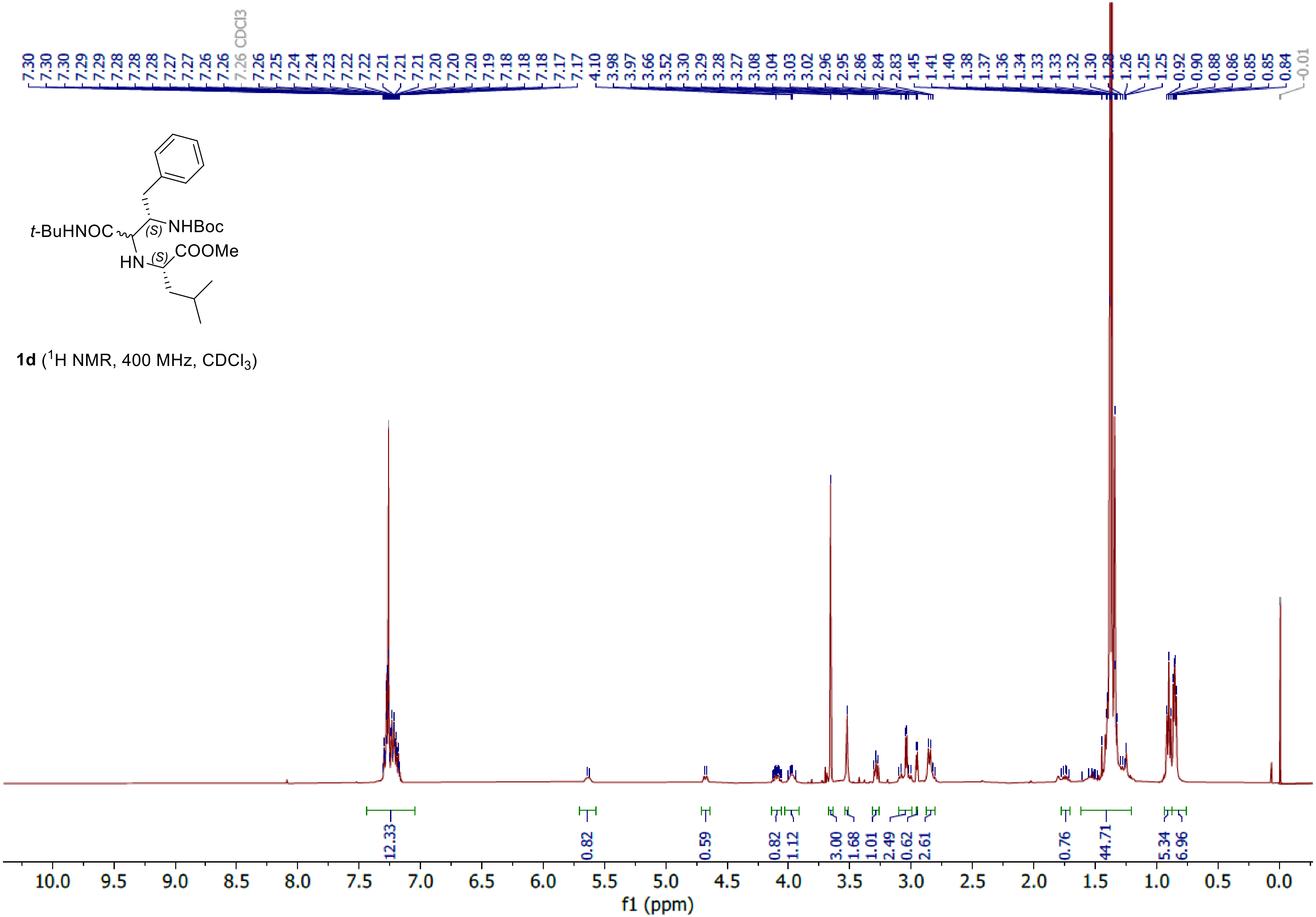

1d (^{1}H NMR, 400 MHz, $CDCl_3$)

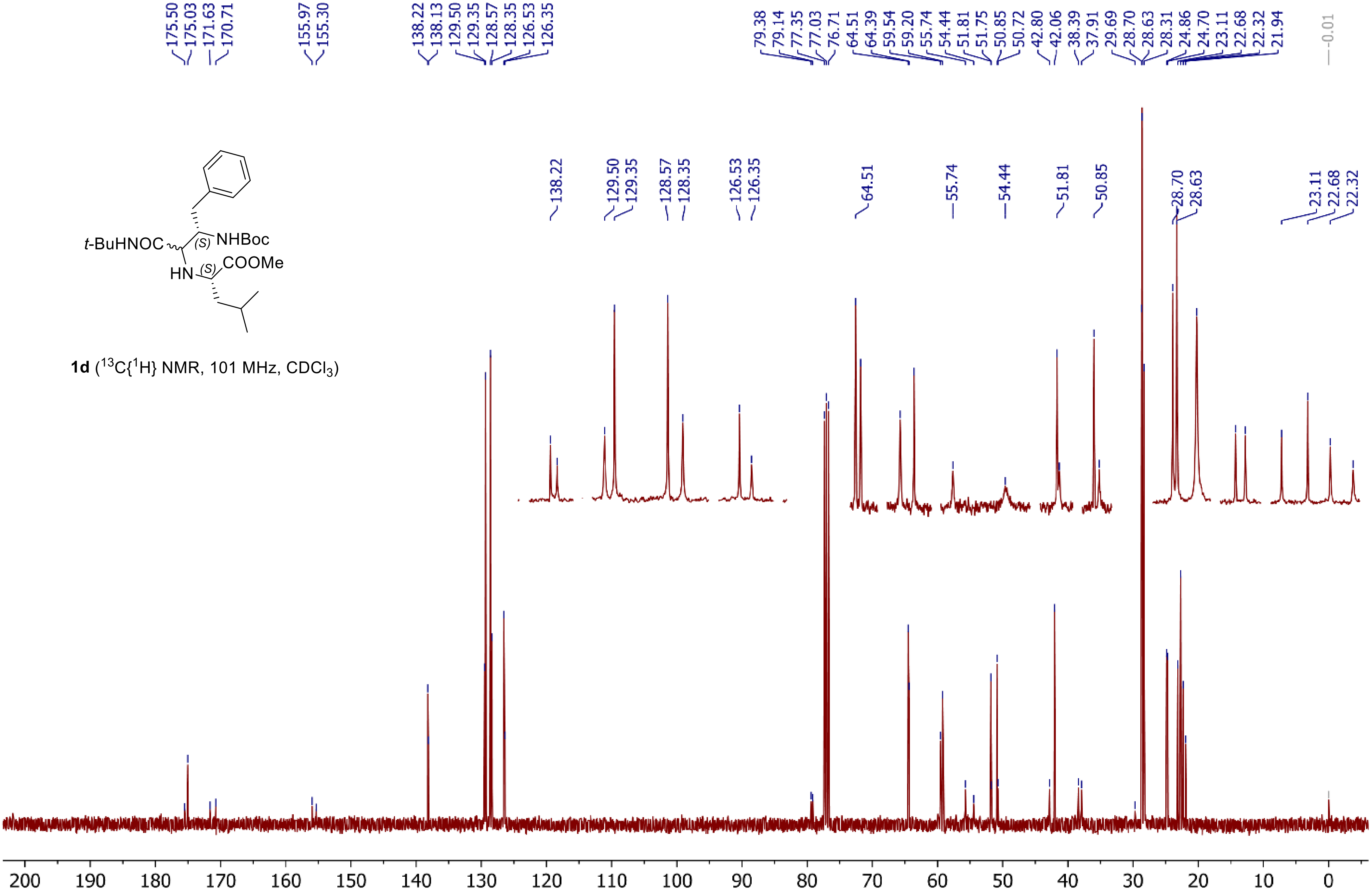

1d ($^{13}C\{^{1}H\}$ NMR, 101 MHz, $CDCl_3$)

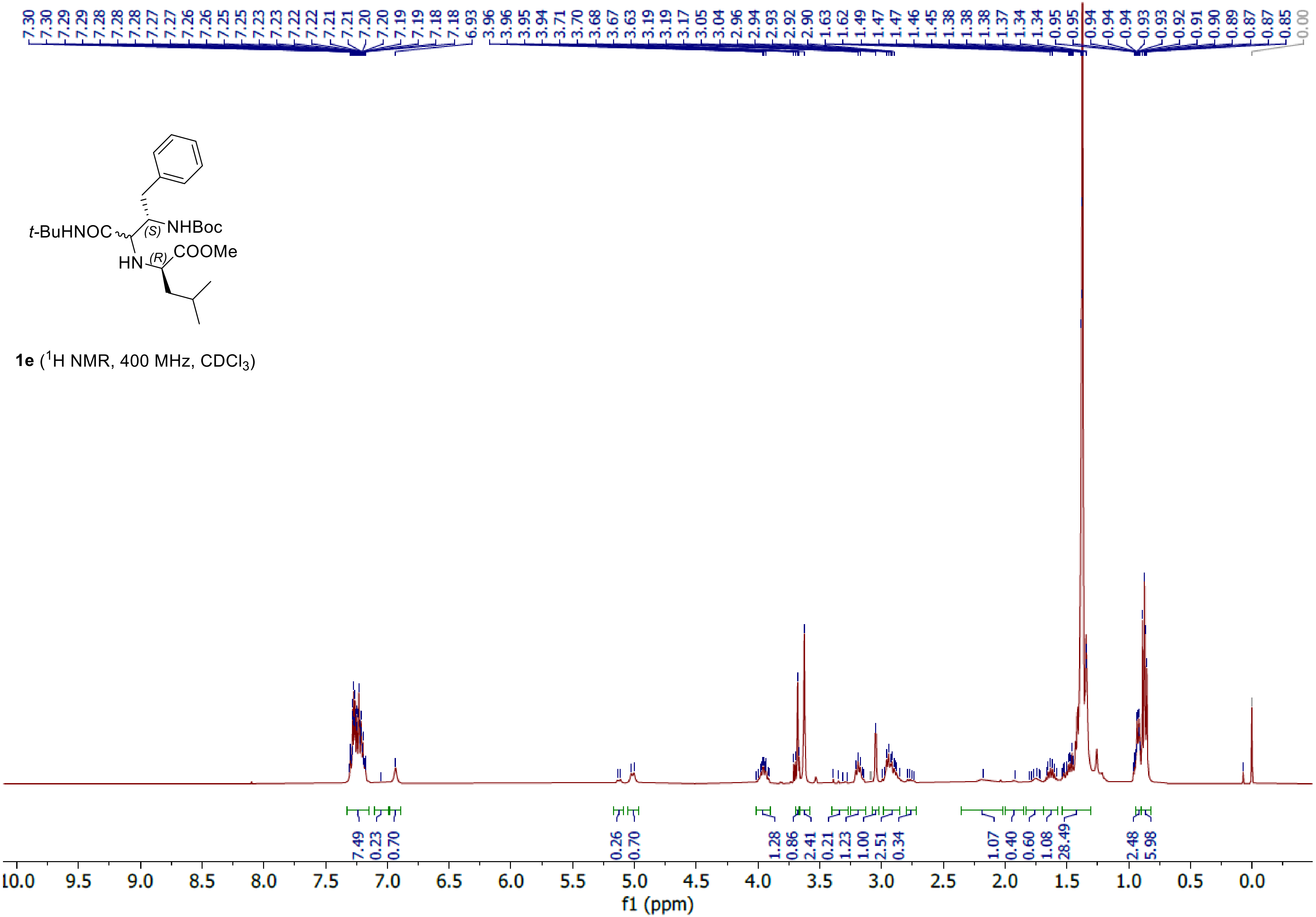

1e (^{1}H NMR, 400 MHz, $CDCl_3$)

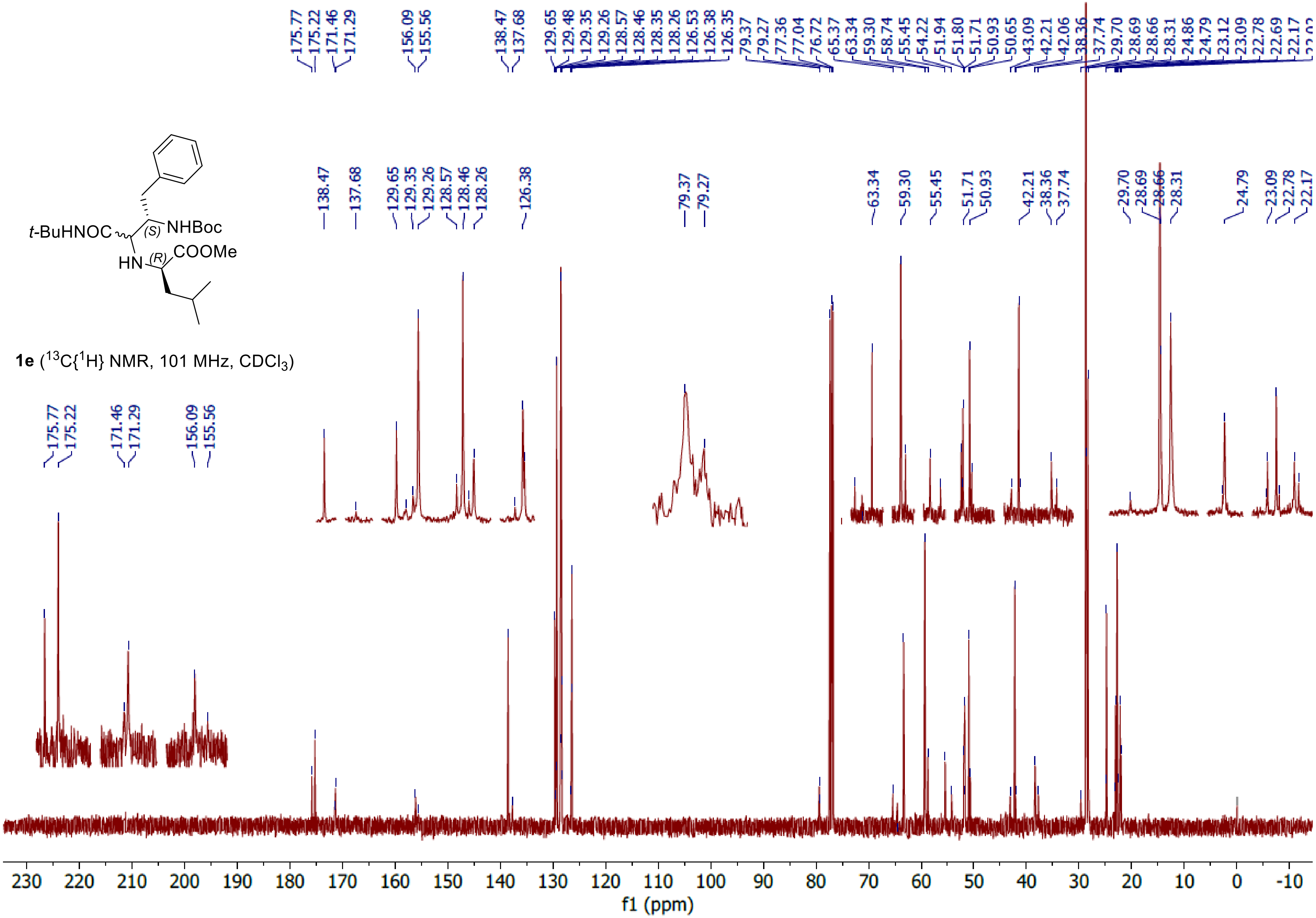

1e ($^{13}C\{^1H\}$ NMR, 101 MHz, $CDCl_3$)

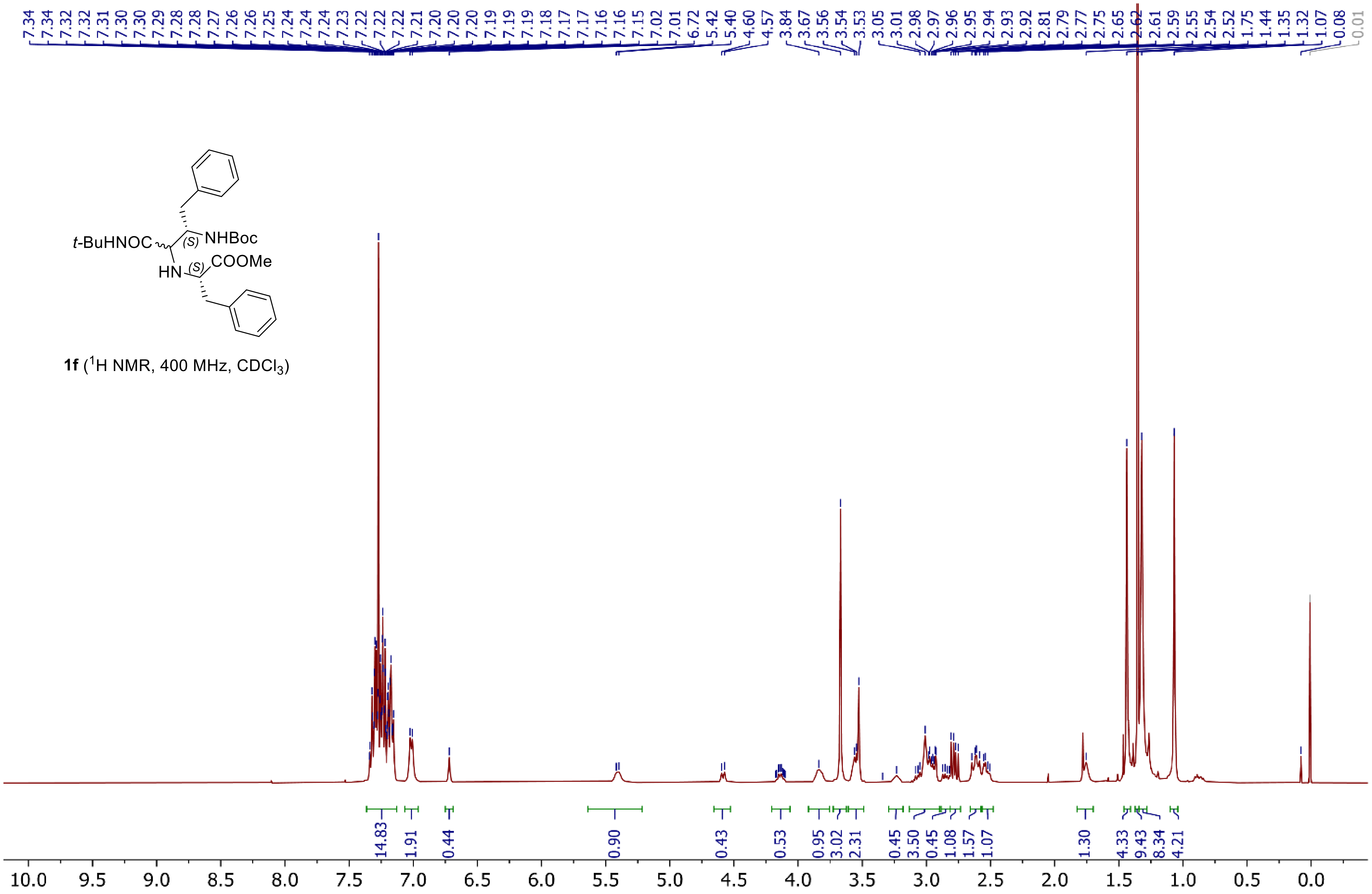
t-BuHNOC
(S)
NHBoc
HN
(S)
COOMe
1f (¹H NMR, 400 MHz, CDCl₃)
10.0 9.5 9.0 8.5 8.0 7.5 7.0 6.5 6.0 5.5 5.0 4.5 4.0 3.5 3.0 2.5 2.0 1.5 1.0 0.5 0.0
14.83 1.91 0.44 0.90 0.43 0.53 0.95 3.02 2.31 0.45 3.50 0.45 1.08 1.57 1.07 1.30 4.33 9.43 8.34 4.21

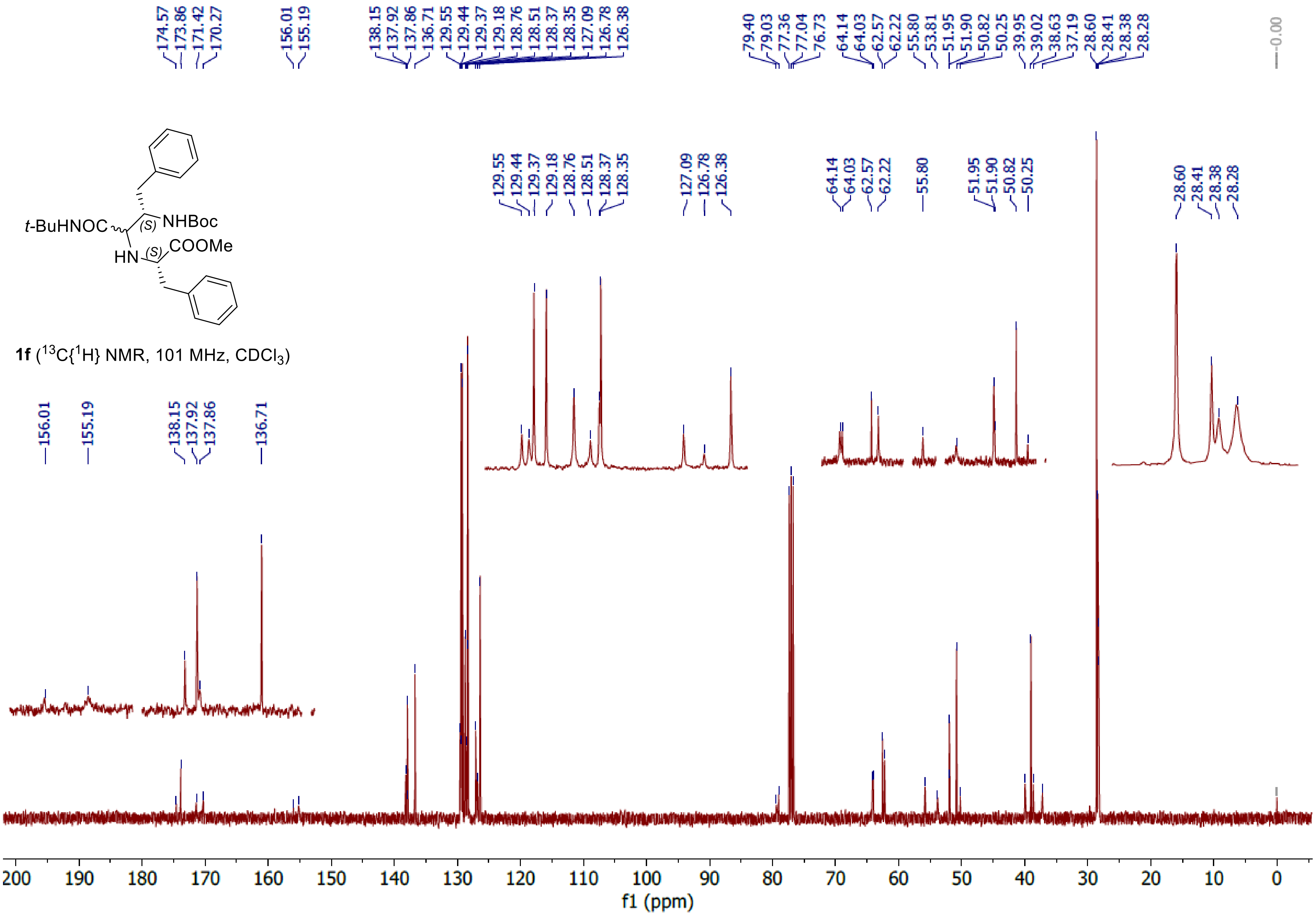
t-BuHNOC
NHBoc
HN
COOMe
(S)
1f (13C{1H} NMR, 101 MHz, CDCl3)
f1 (ppm)

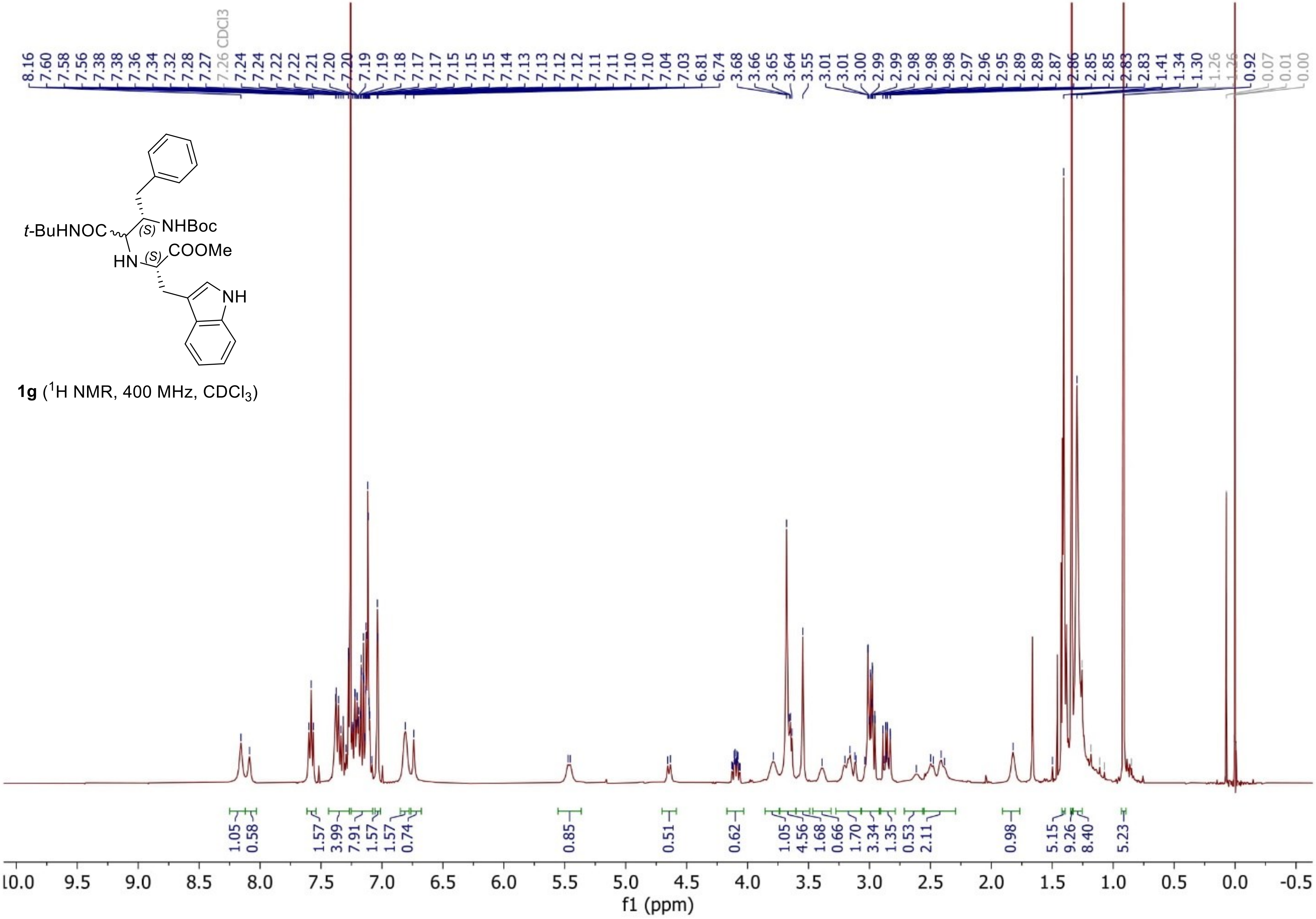

1g (^{1}H NMR, 400 MHz, $CDCl_3$)

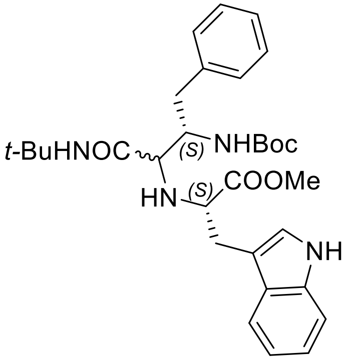

1g ($^{13}C\{^{1}H\}$ NMR, 101 MHz, $CDCl_3$)

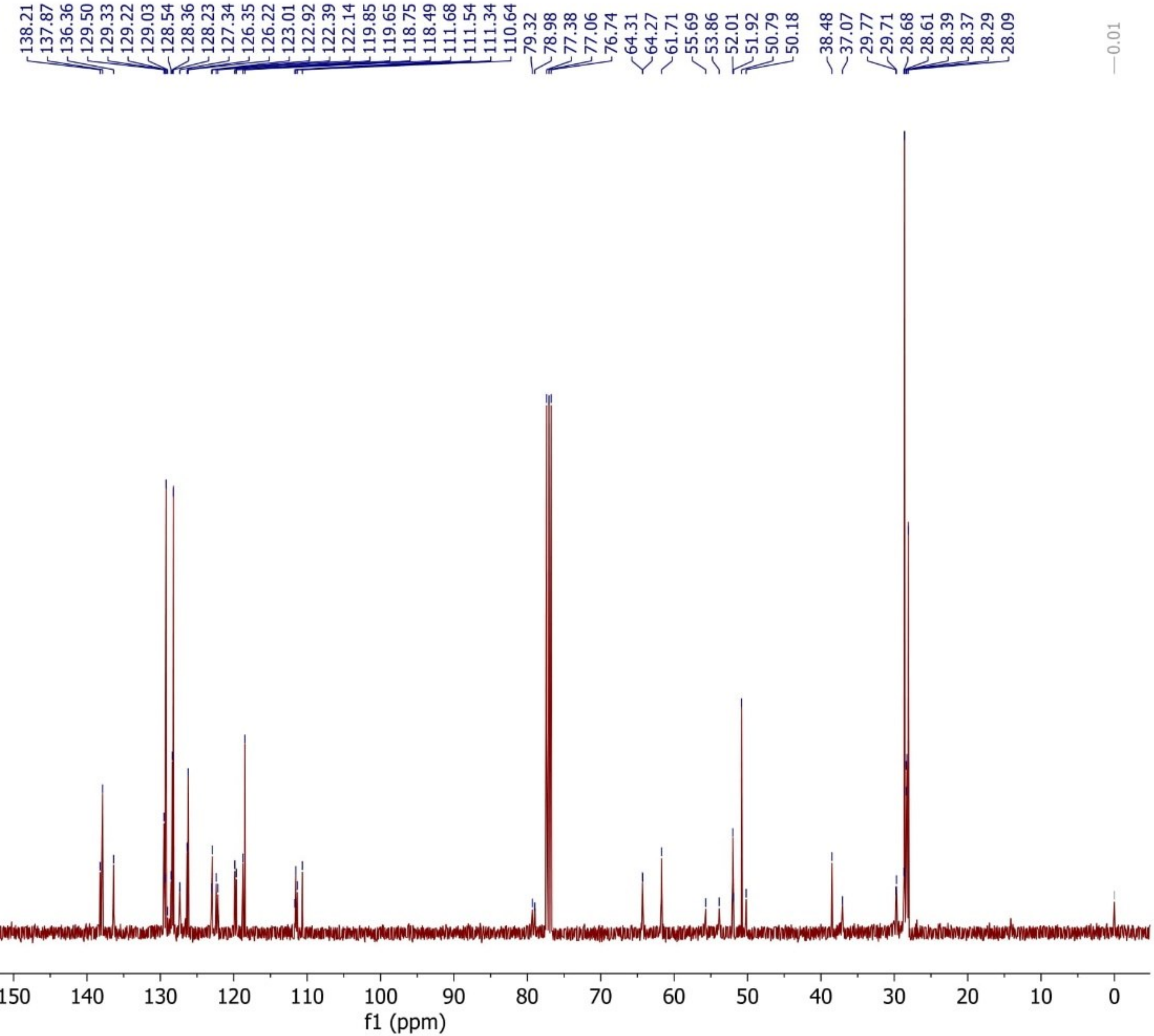

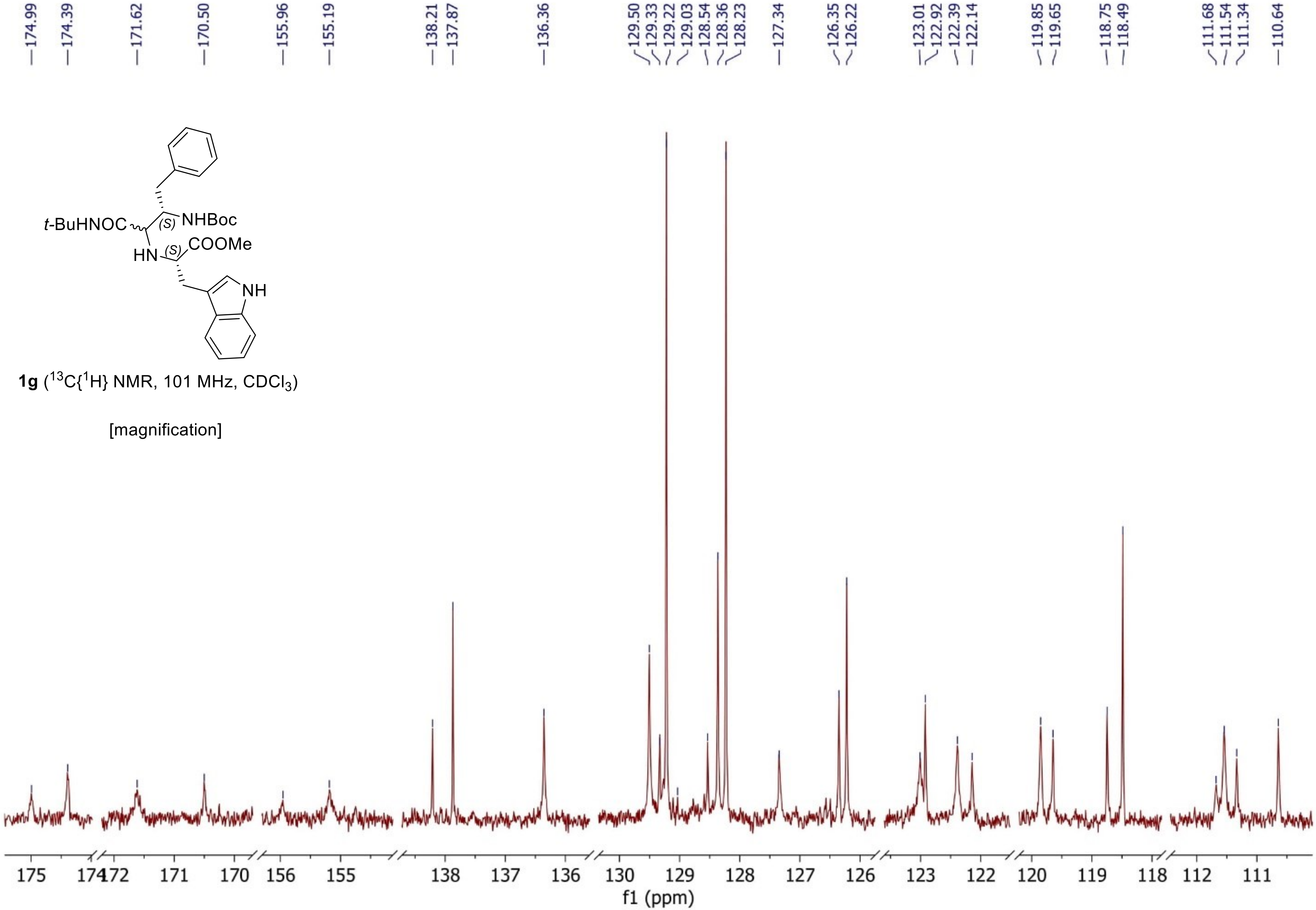

1g ($^{13}C\{^{1}H\}$ NMR, 101 MHz, $CDCl_3$)

[magnification]

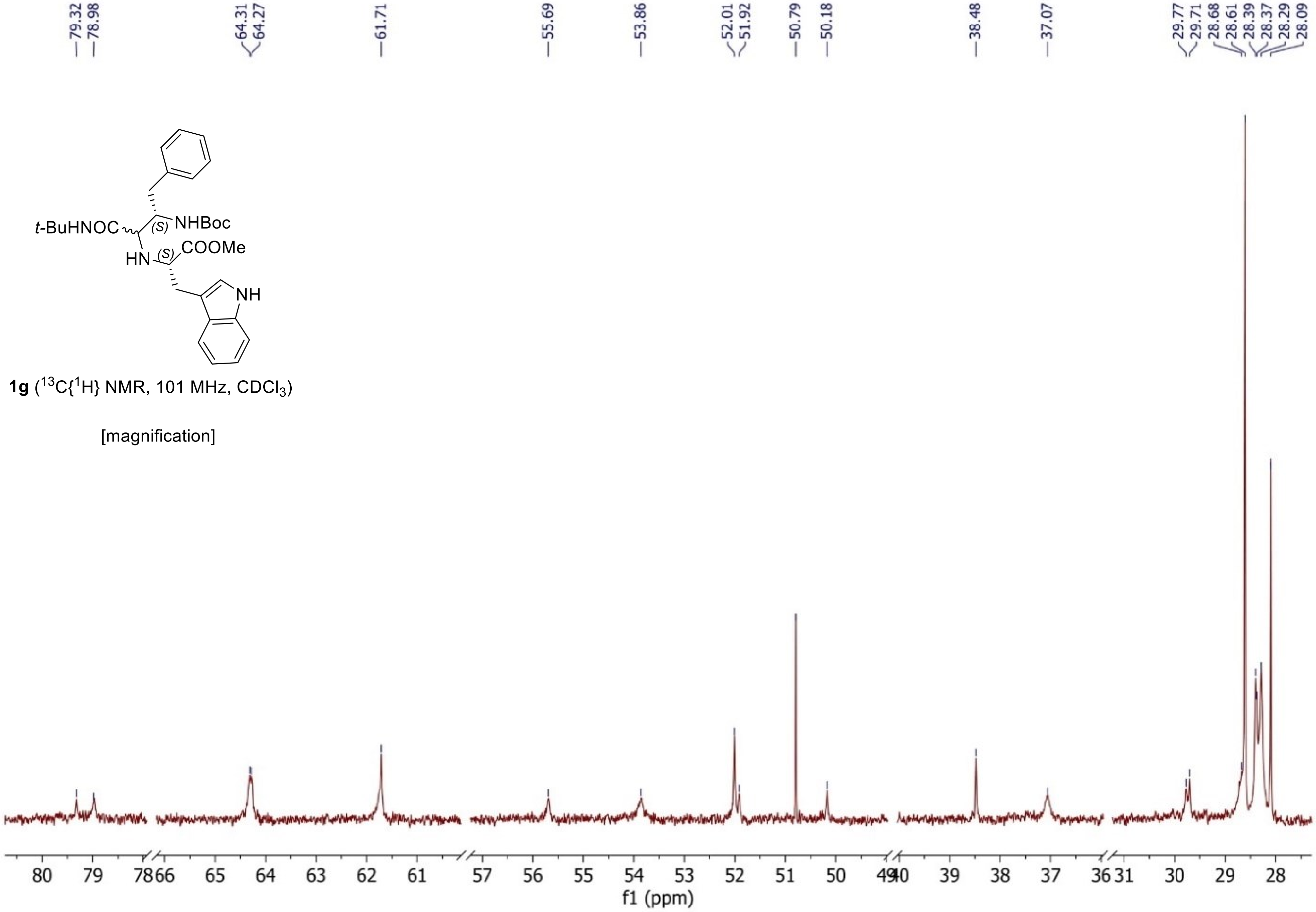

1g ($^{13}C\{^{1}H\}$ NMR, 101 MHz, $CDCl_3$)

[magnification]

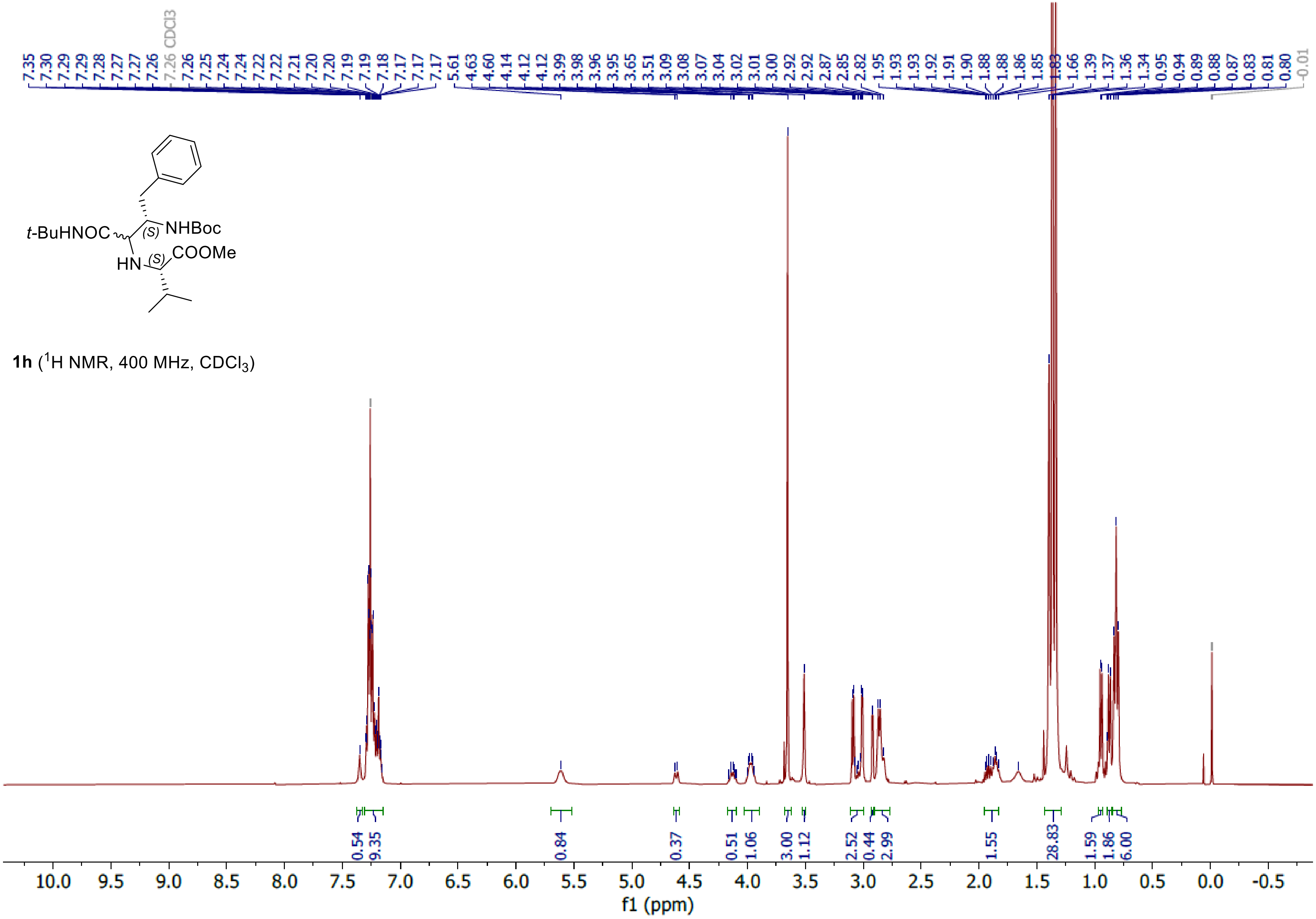

1h (^{1}H NMR, 400 MHz, $CDCl_3$)

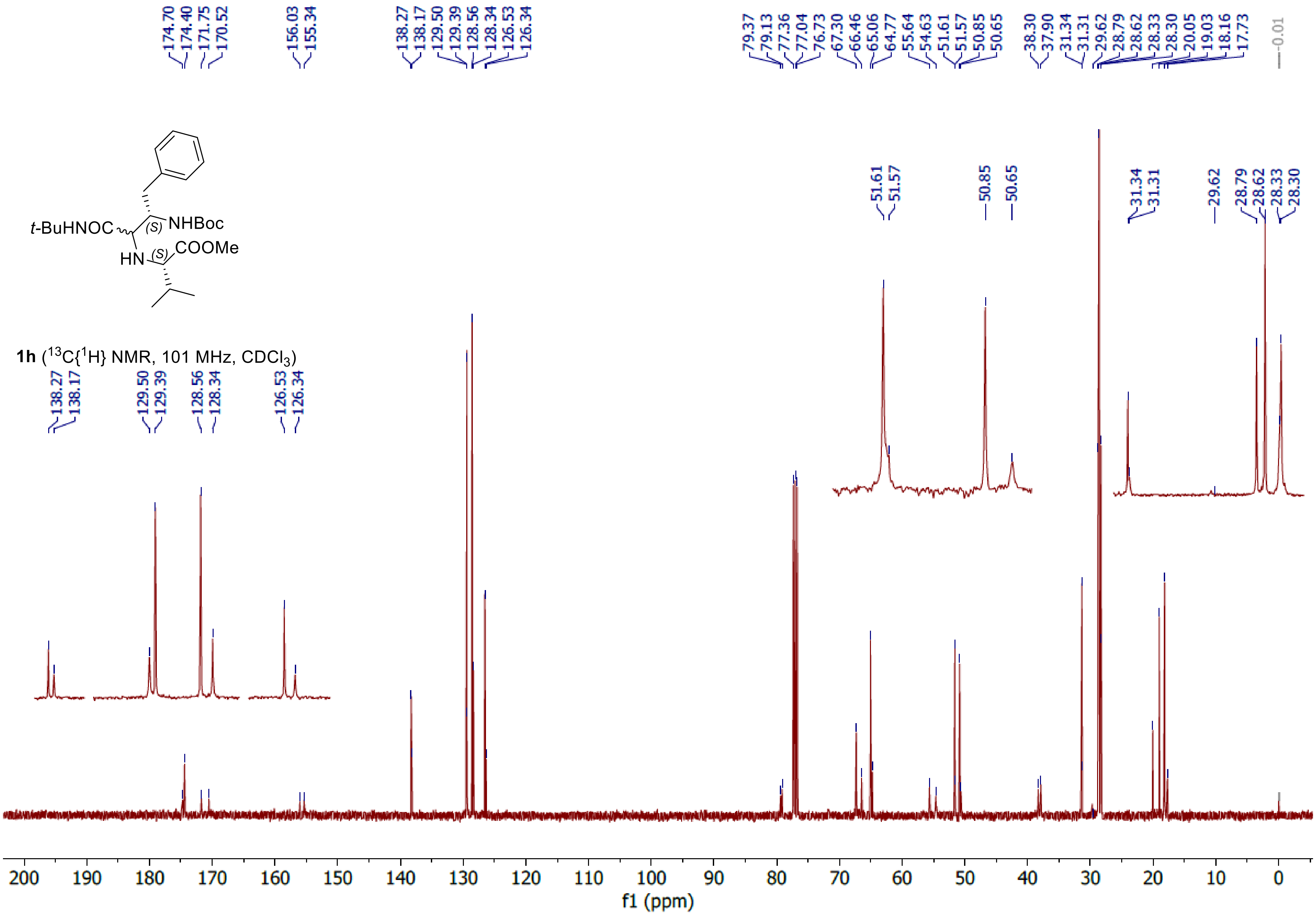

1h ($^{13}C\{^{1}H\}$ NMR, 101 MHz, $CDCl_3$)

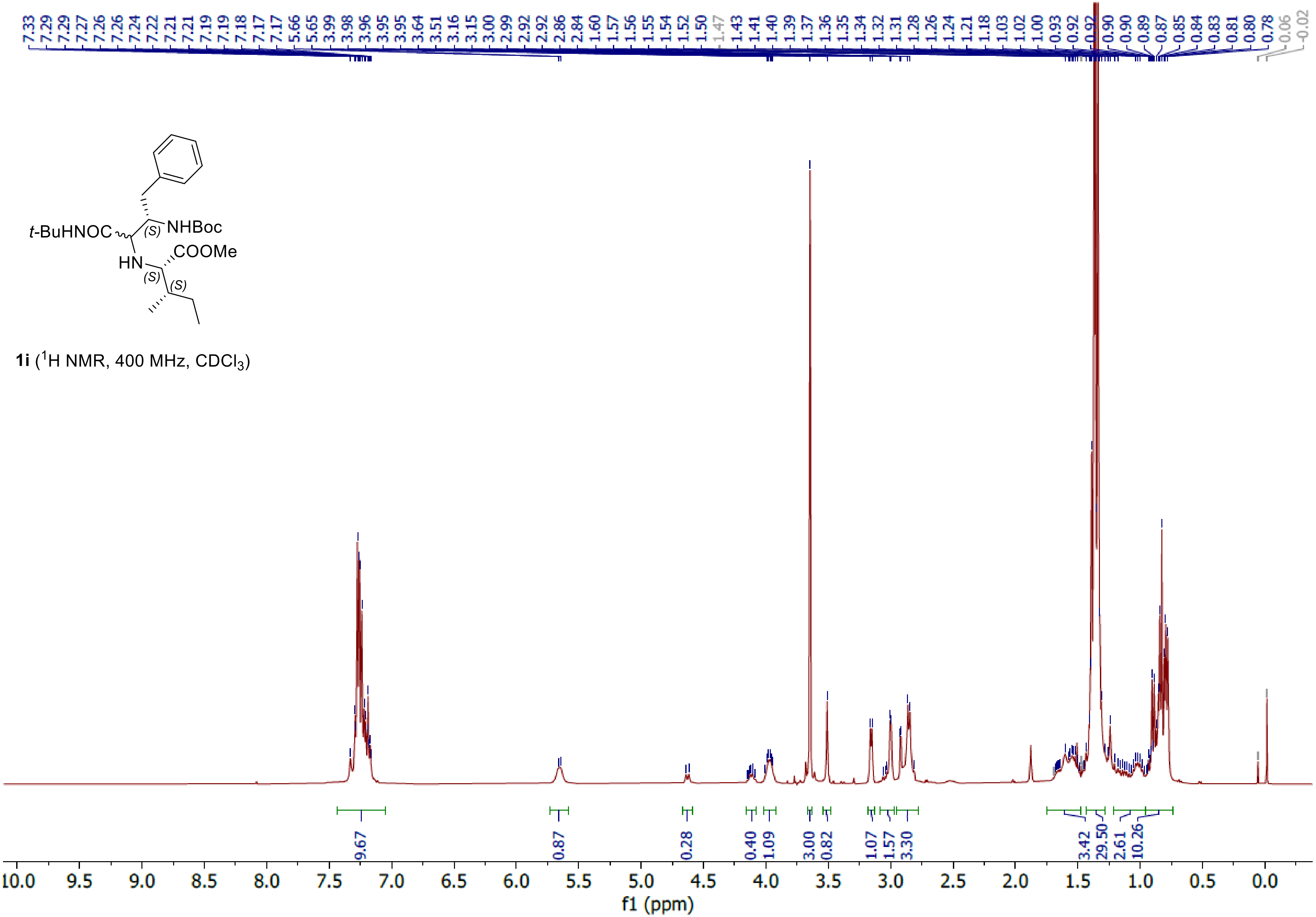

1i (^{1}H NMR, 400 MHz, $CDCl_3$)

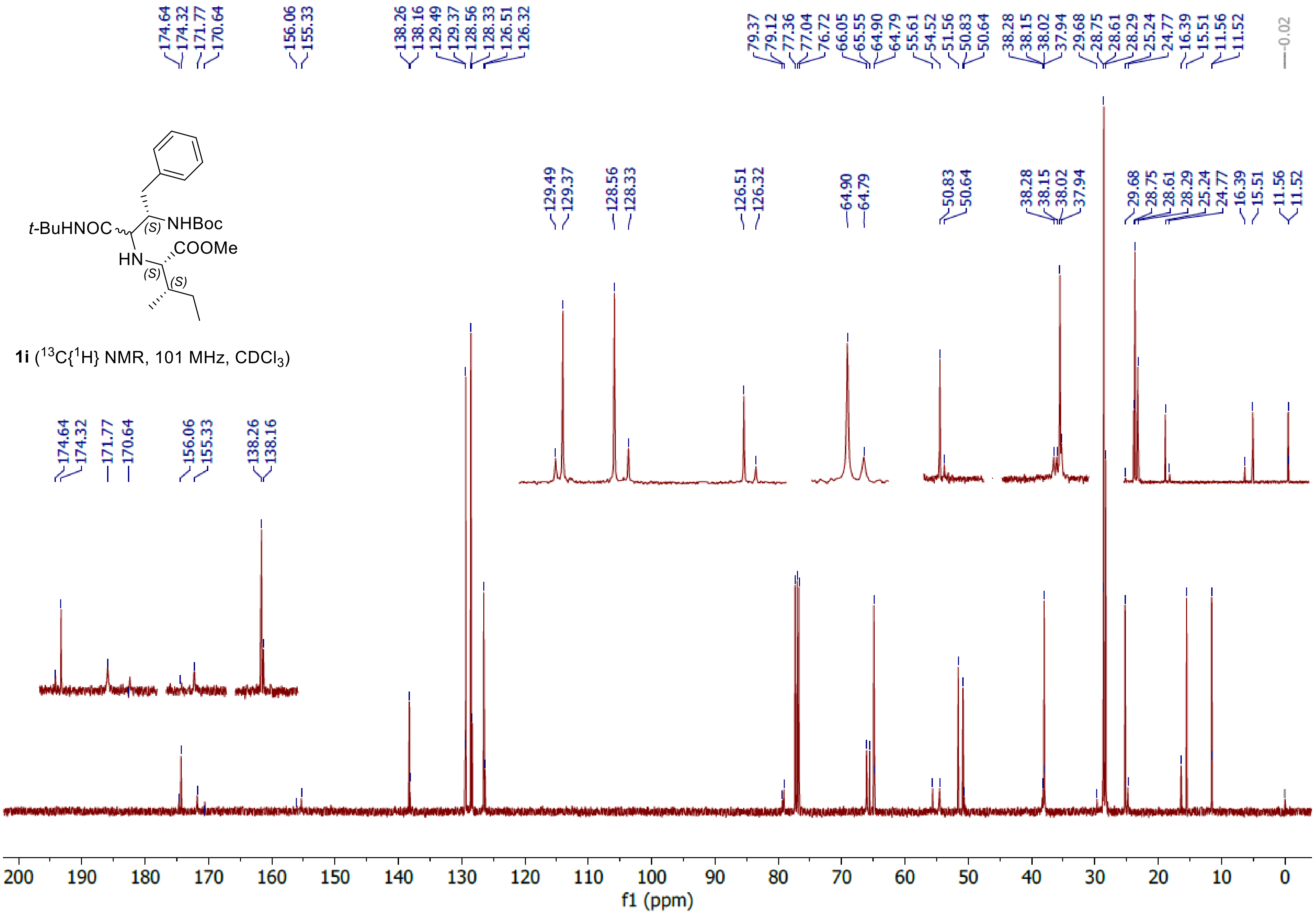
t-BuHNOC
NHBoc
COOMe
HN
(S)
1i ($^{13}C\{^{1}H\}$ NMR, 101 MHz, $CDCl_3$)
174.64
174.32
171.77
170.64
156.06
155.33
138.26
138.16
129.49
129.37
128.56
128.33
126.51
126.32
79.37
79.12
77.36
77.04
76.72
66.05
65.55
64.90
64.79
55.61
54.52
51.56
50.83
50.64
38.28
38.15
38.02
37.94
29.68
28.75
28.61
28.29
25.24
24.77
16.39
15.51
11.56
11.52
-0.02
f1 (ppm)

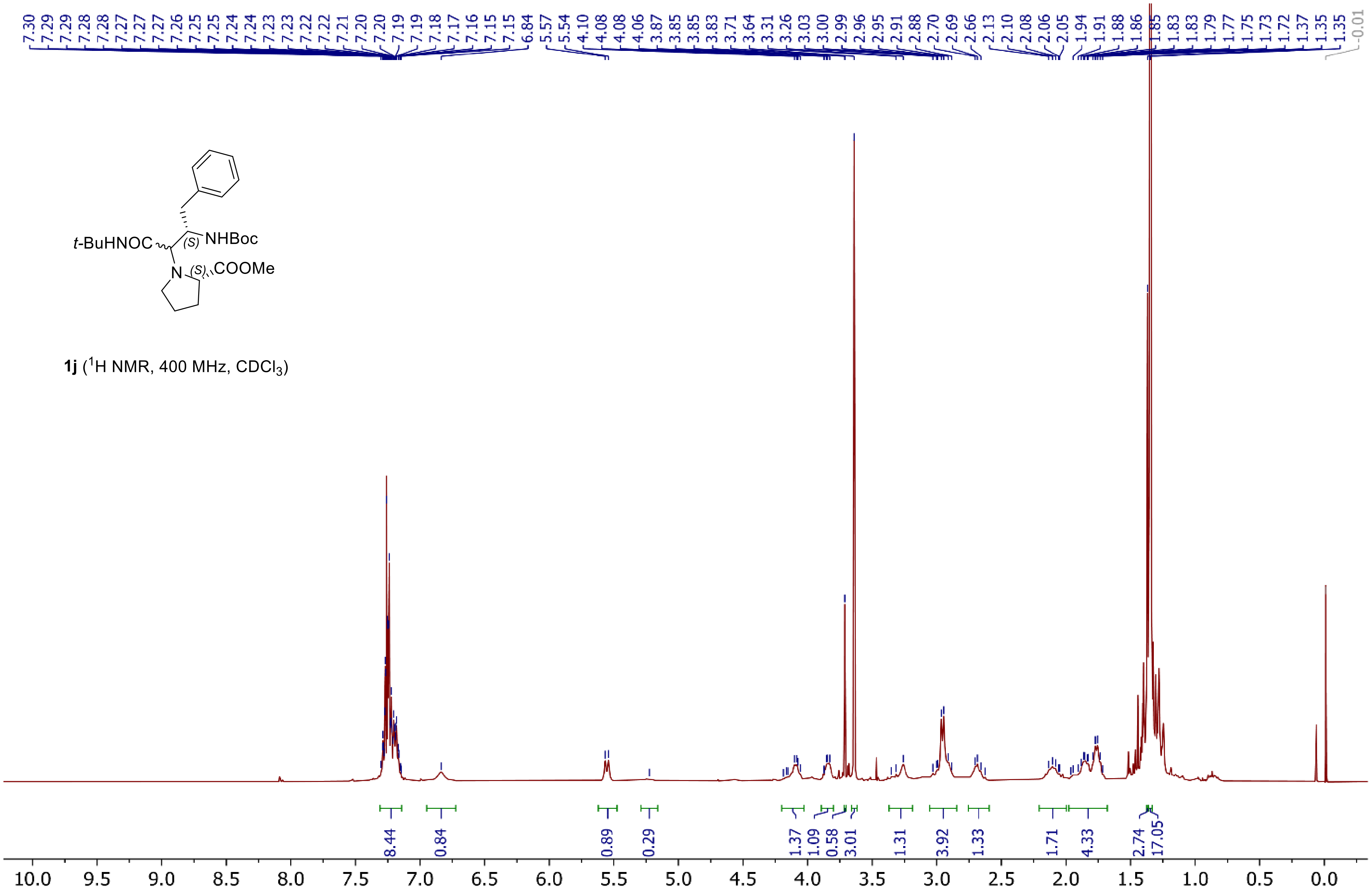

1j (^{1}H NMR, 400 MHz, $CDCl_3$)

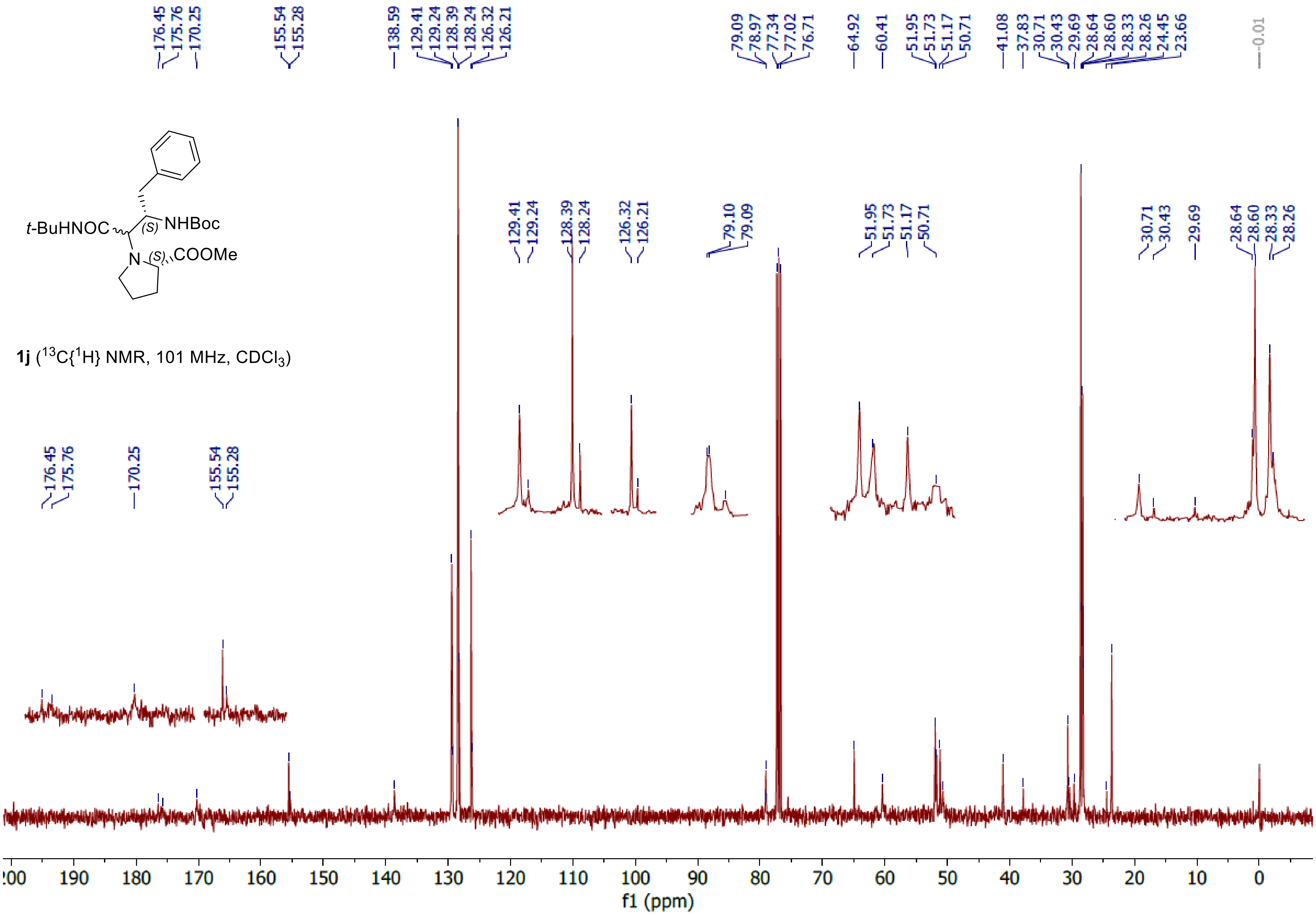

1j ($^{13}C\{^{1}H\}$ NMR, 101 MHz, $CDCl_3$)

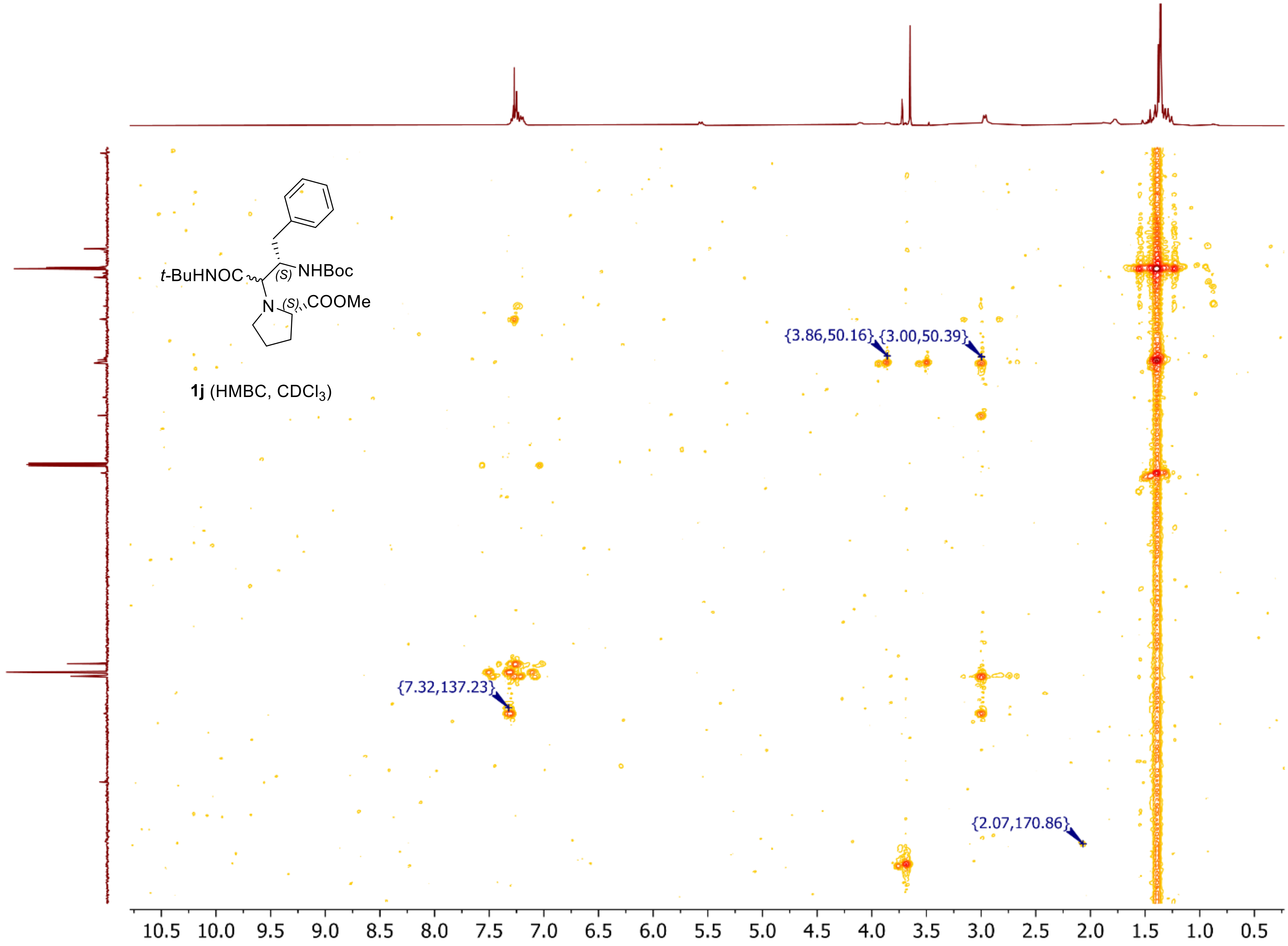

1j (HMBC, $CDCl_3$)

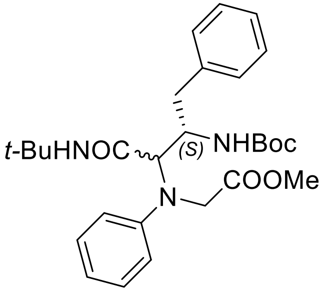

1k (^{1}H NMR, 400 MHz, $CDCl_3$)

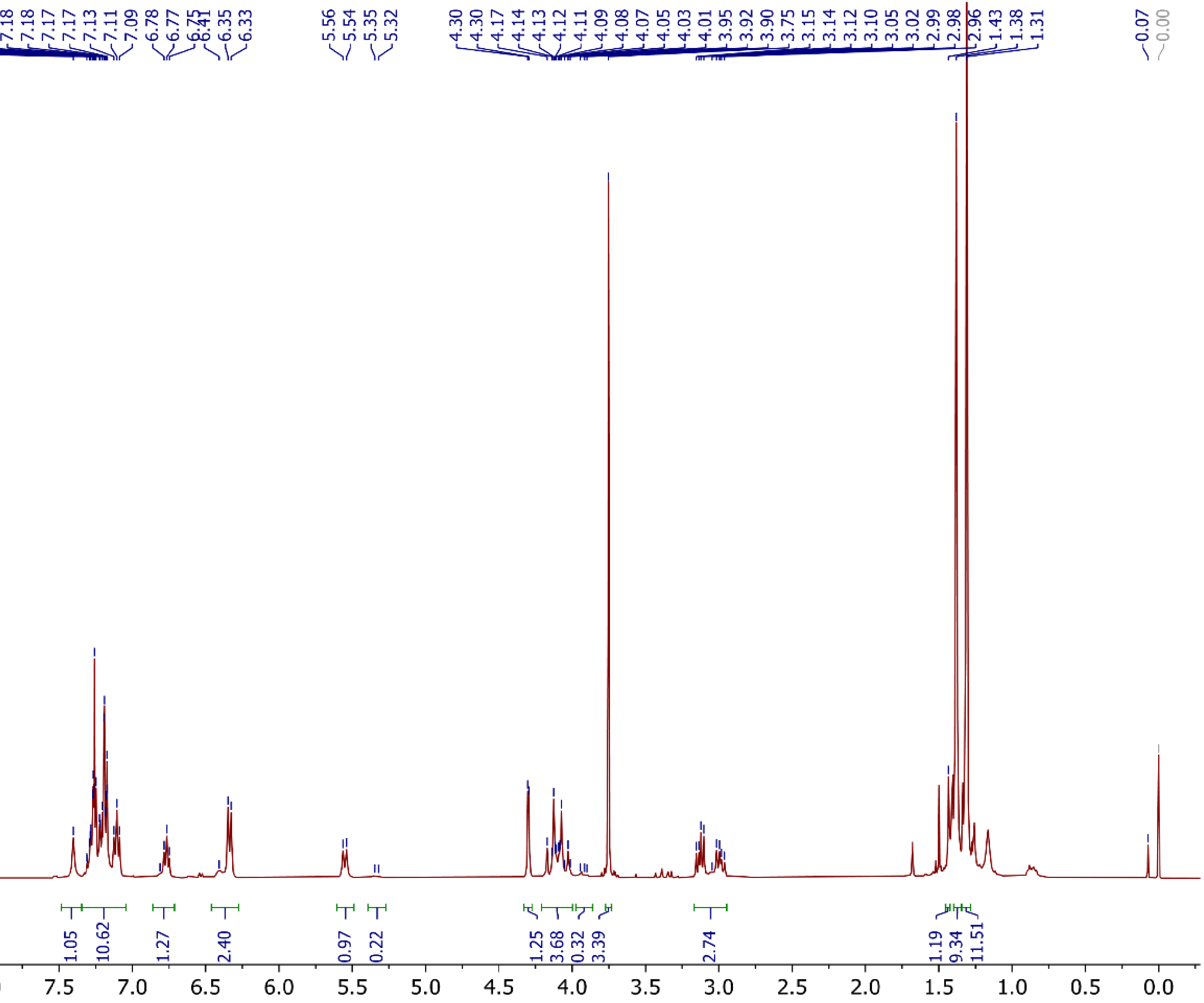

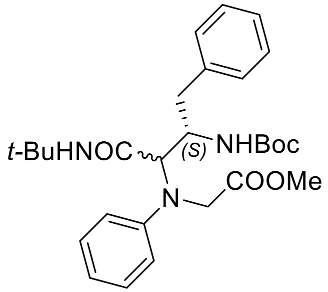

1k ($^{13}C\{^{1}H\}$ NMR, 101 MHz, $CDCl_3$)

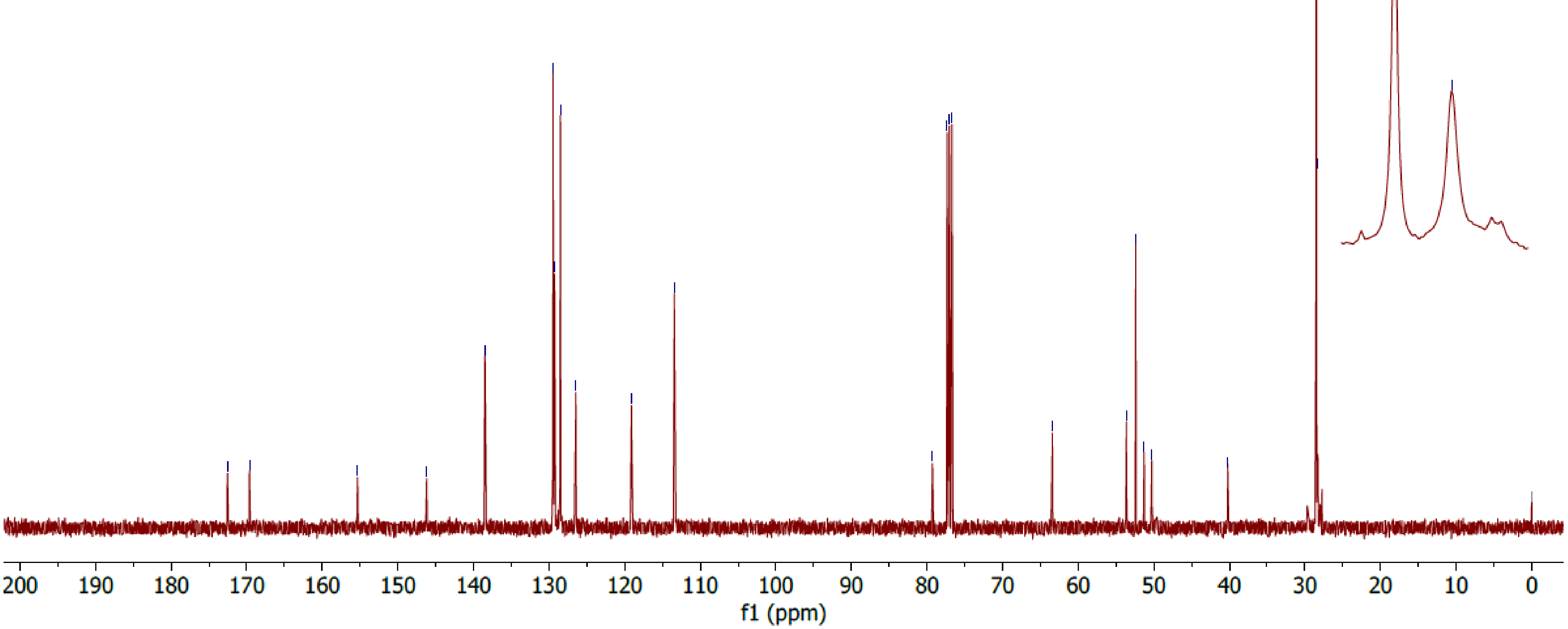

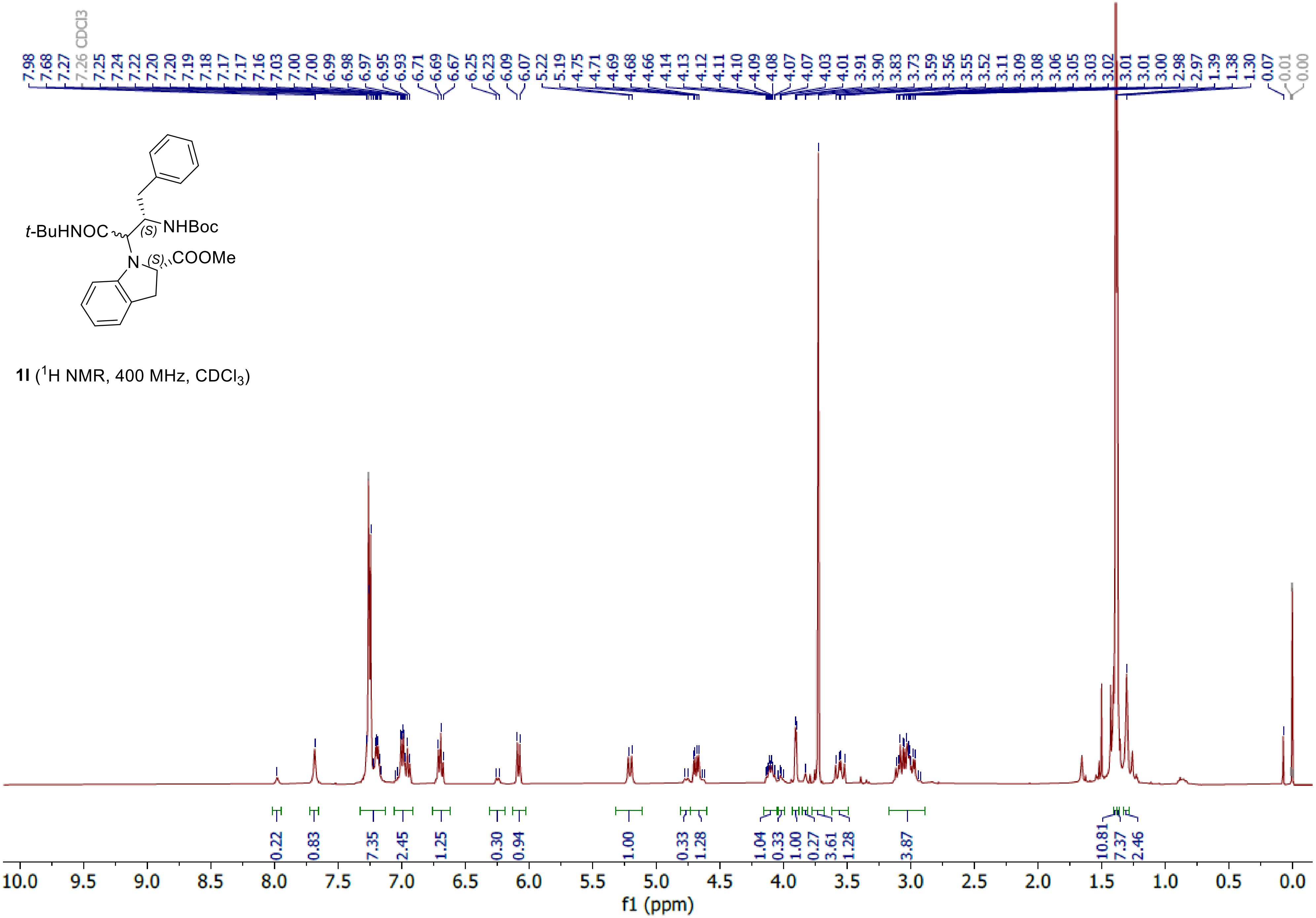

1l (^1H NMR, 400 MHz, $CDCl_3$)

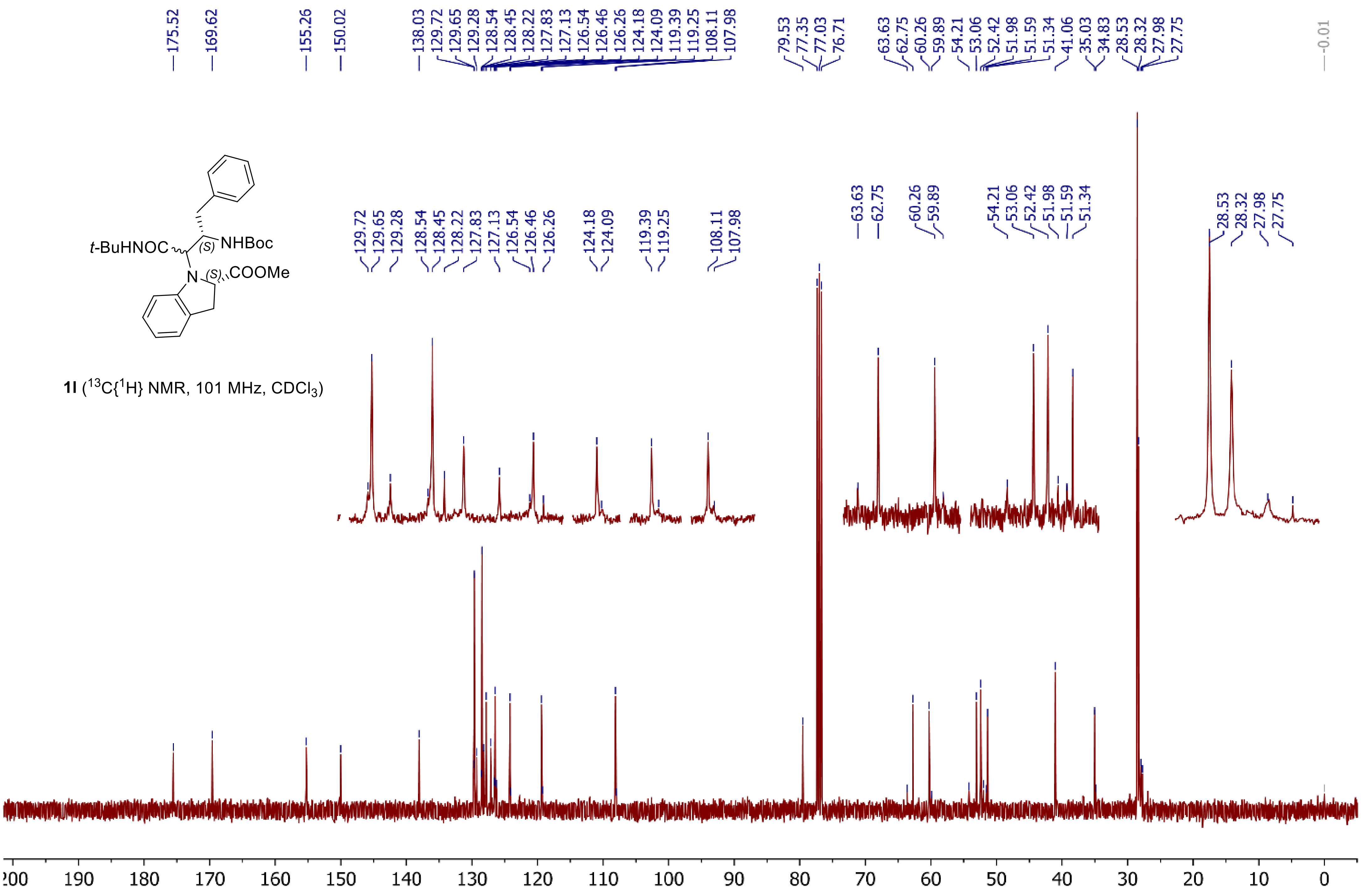

1l ($^{13}C\{^{1}H\}$ NMR, 101 MHz, $CDCl_3$)

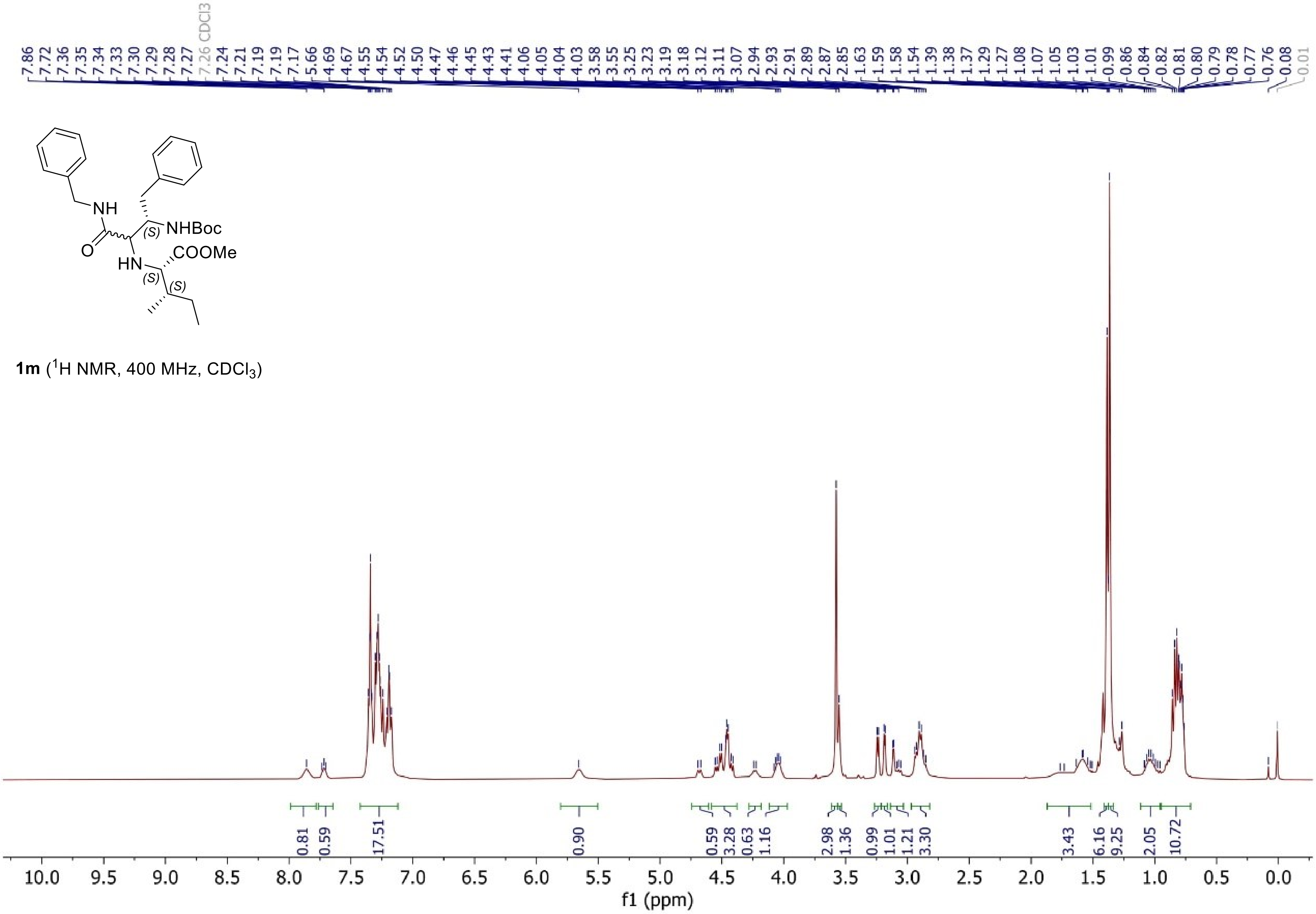

1m (^{1}H NMR, 400 MHz, $CDCl_3$)

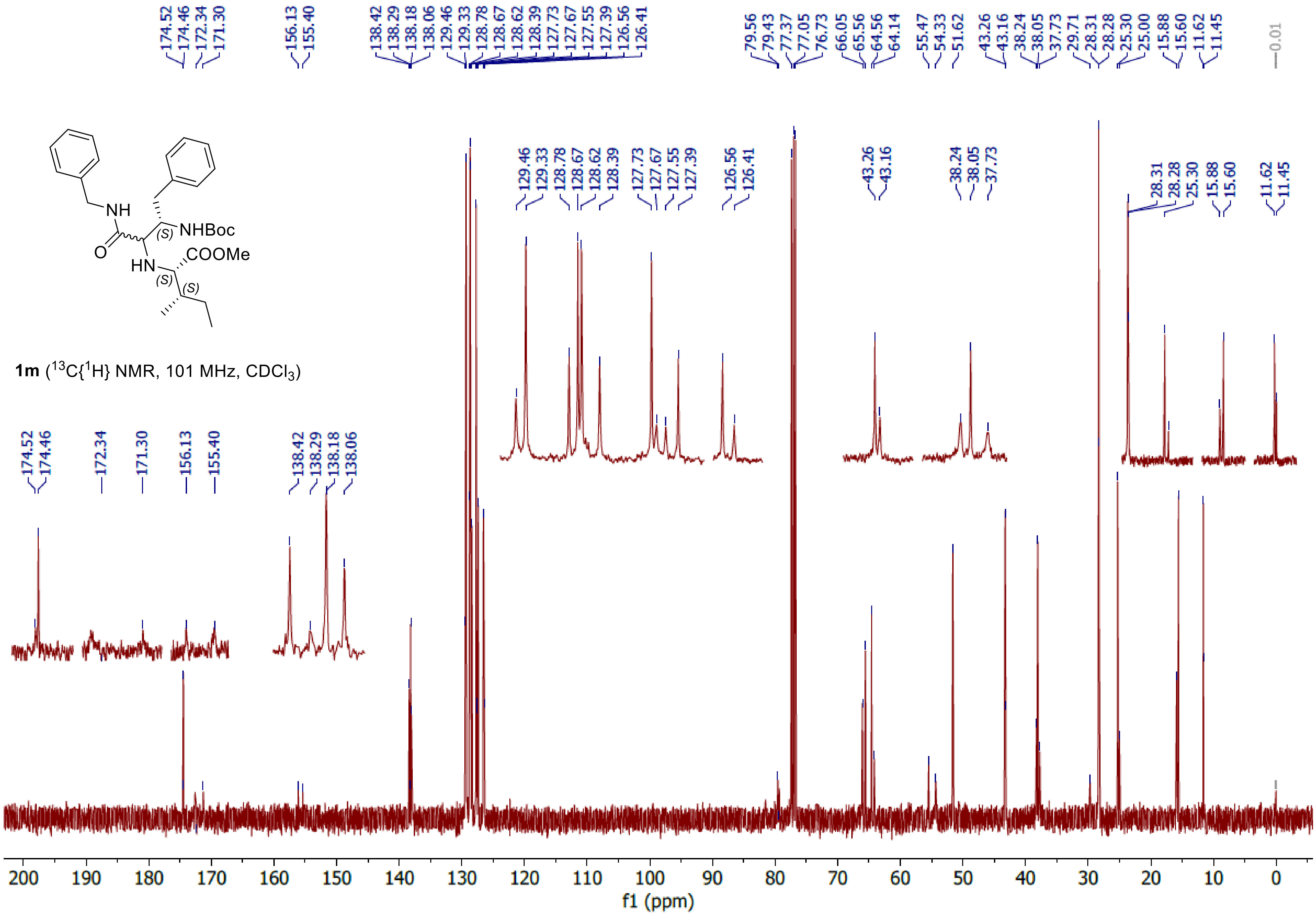

1m ($^{13}C\{^{1}H\}$ NMR, 101 MHz, $CDCl_3$)

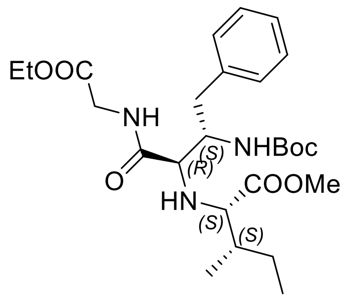

(2*R*,3*S*)-1n (^{1}H NMR, 400 MHz, $CDCl_3$)

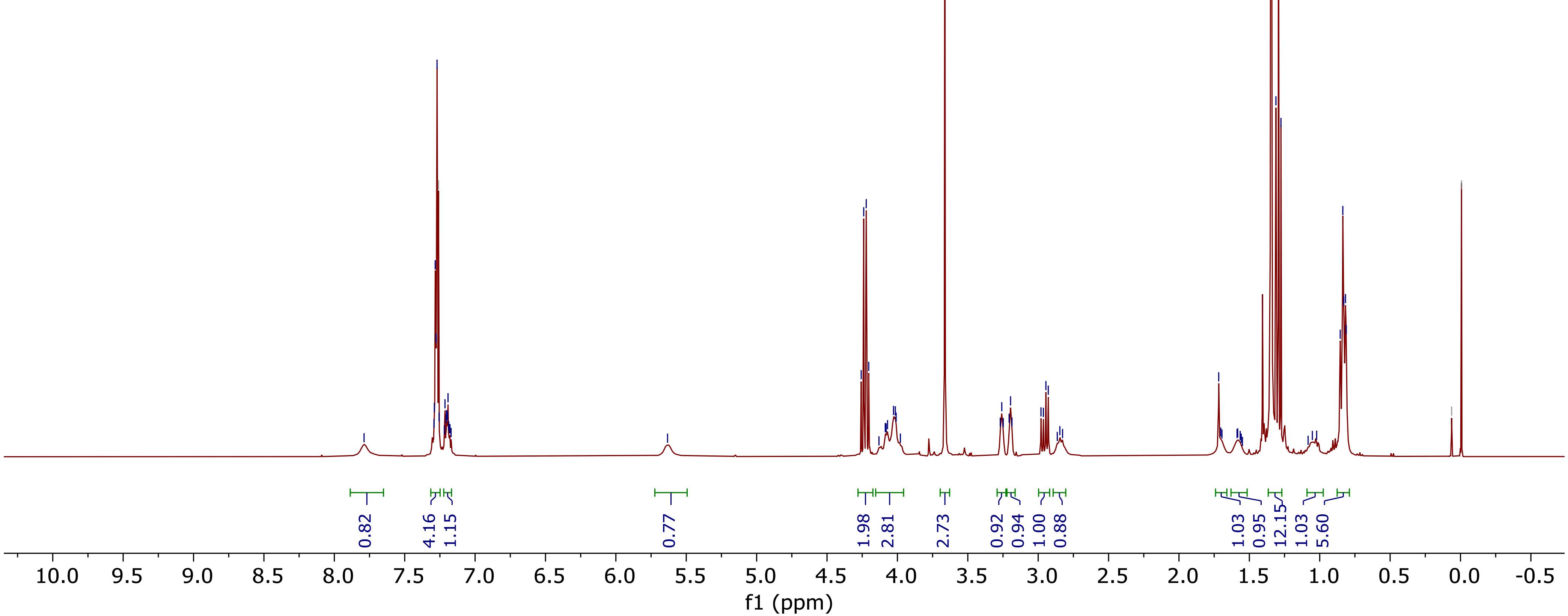

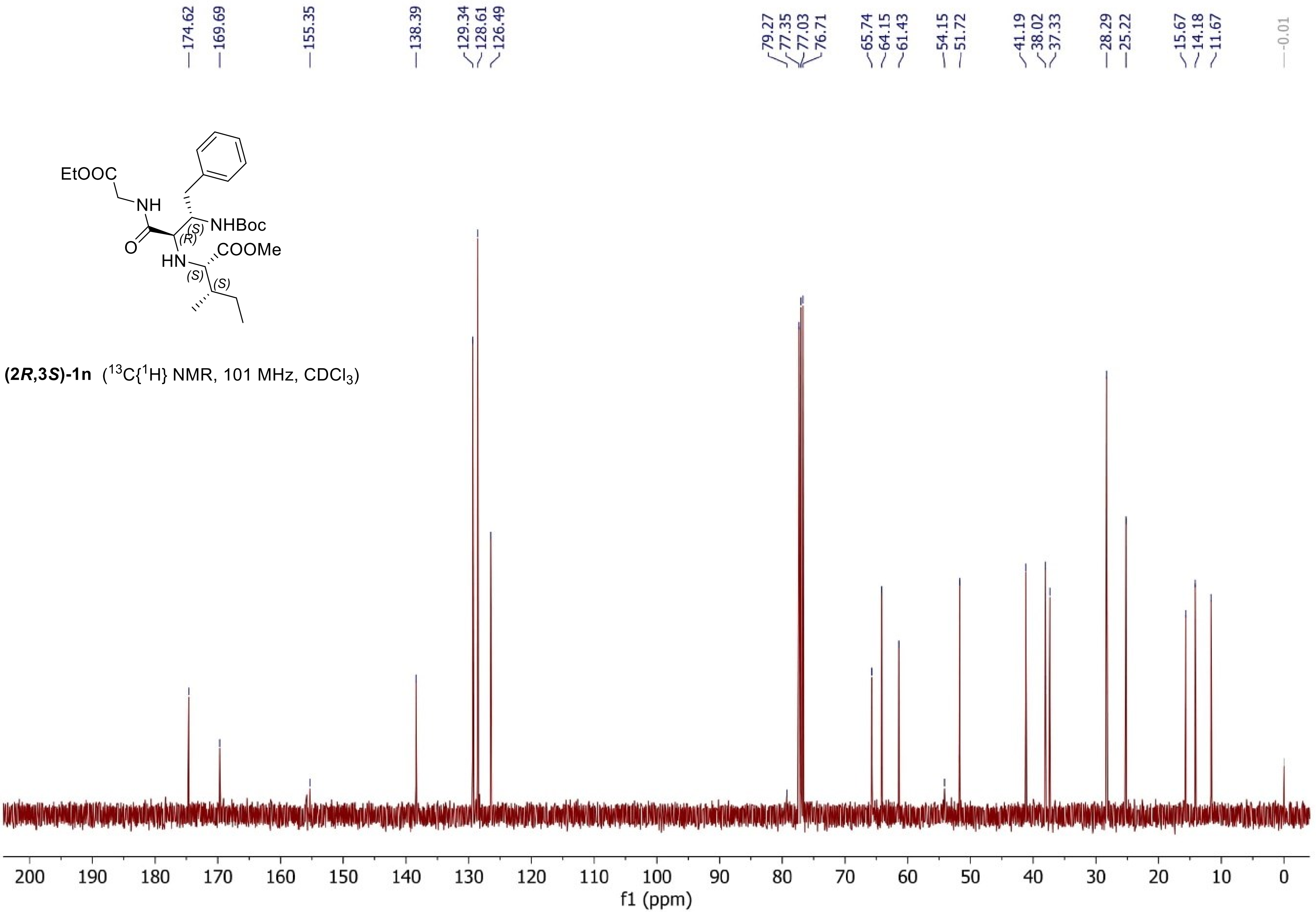

(2*R*,3*S*)-1n ($^{13}C\{^{1}H\}$ NMR, 101 MHz, $CDCl_3$)

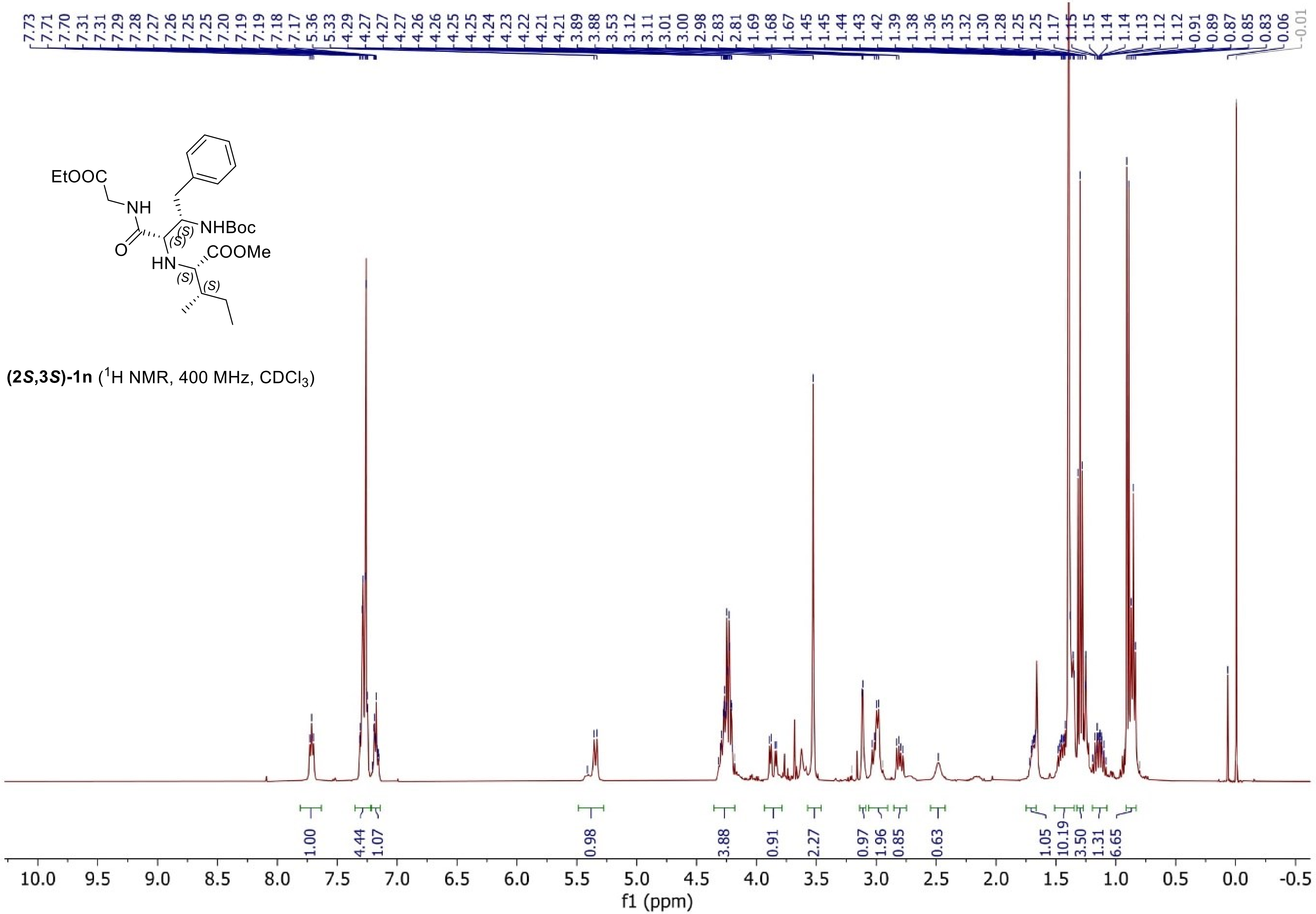

(2*S*,3*S*)-1n (^{1}H NMR, 400 MHz, $CDCl_3$)

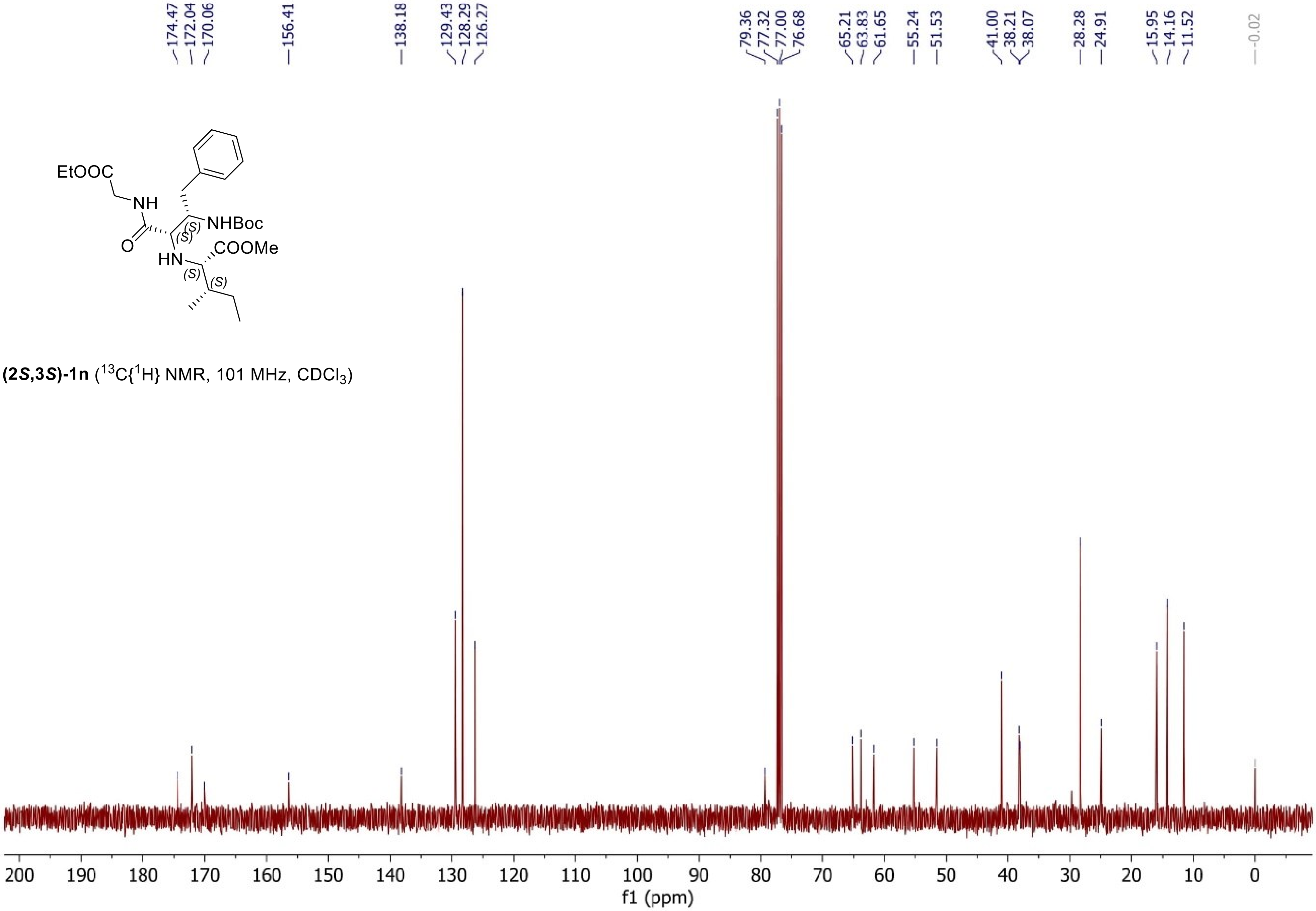

(2*S*,3*S*)-1n ($^{13}C\{^1H\}$ NMR, 101 MHz, $CDCl_3$)

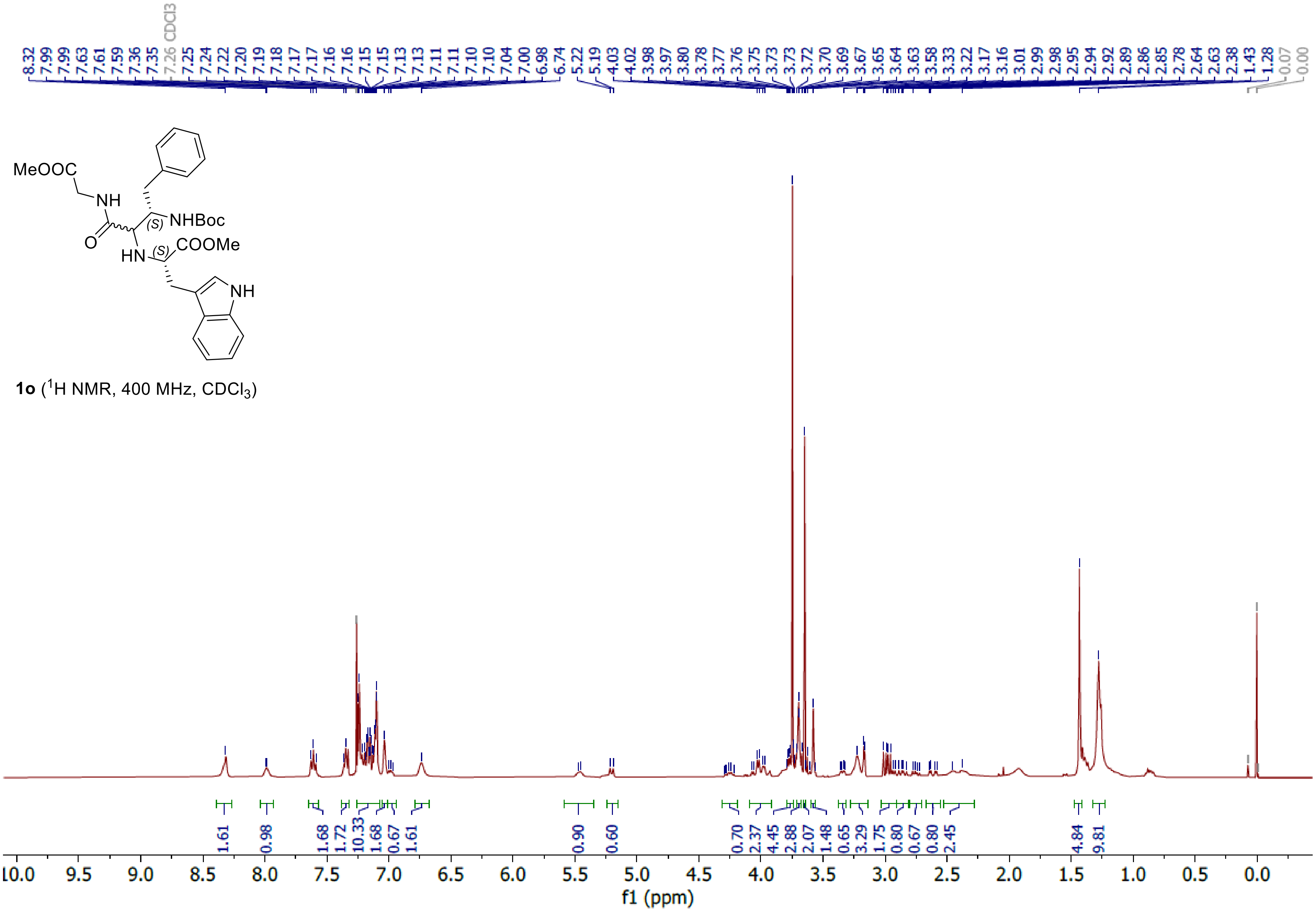

1o (^1H NMR, 400 MHz, $CDCl_3$)

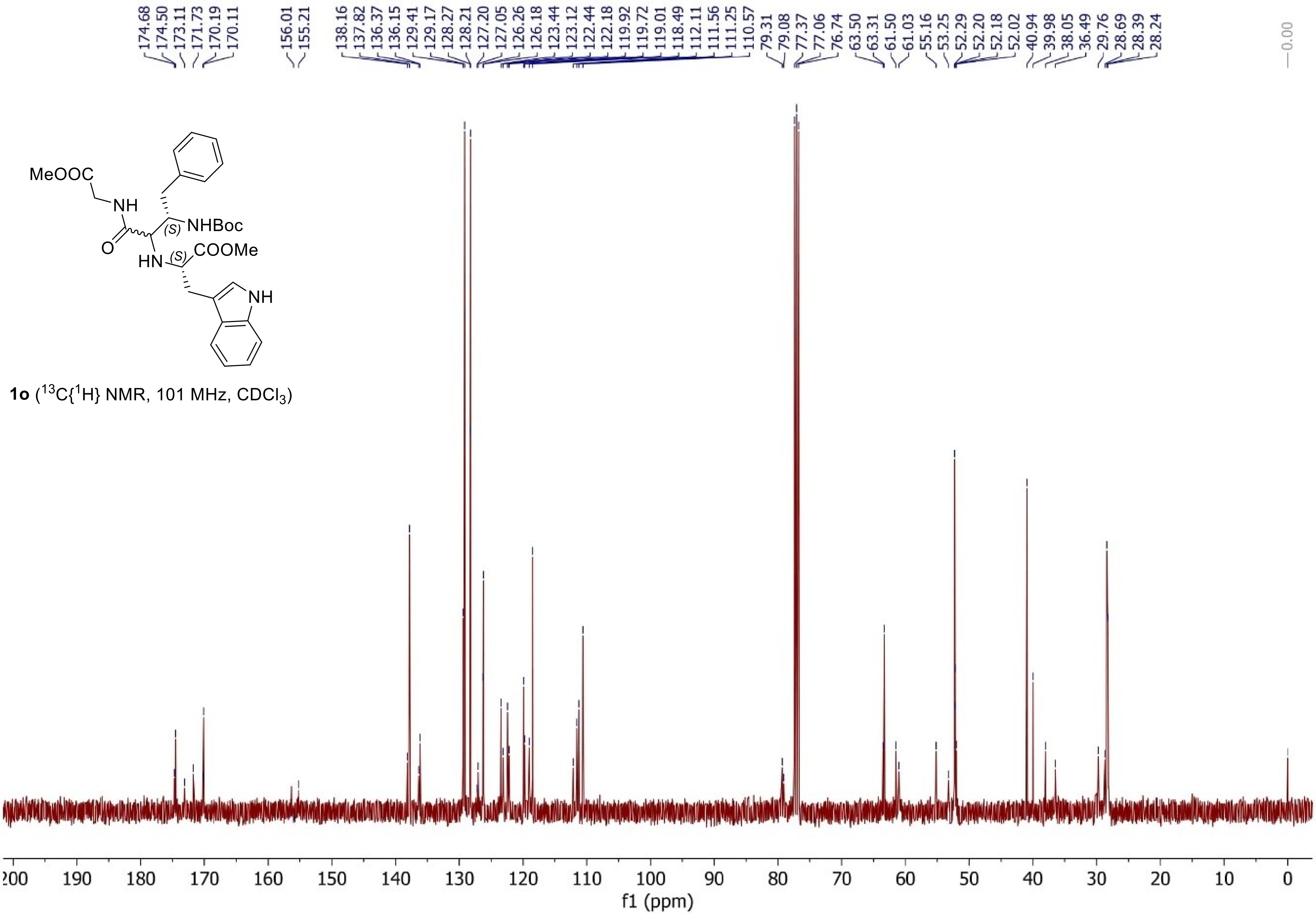

1o ($^{13}C\{^1H\}$ NMR, 101 MHz, $CDCl_3$)

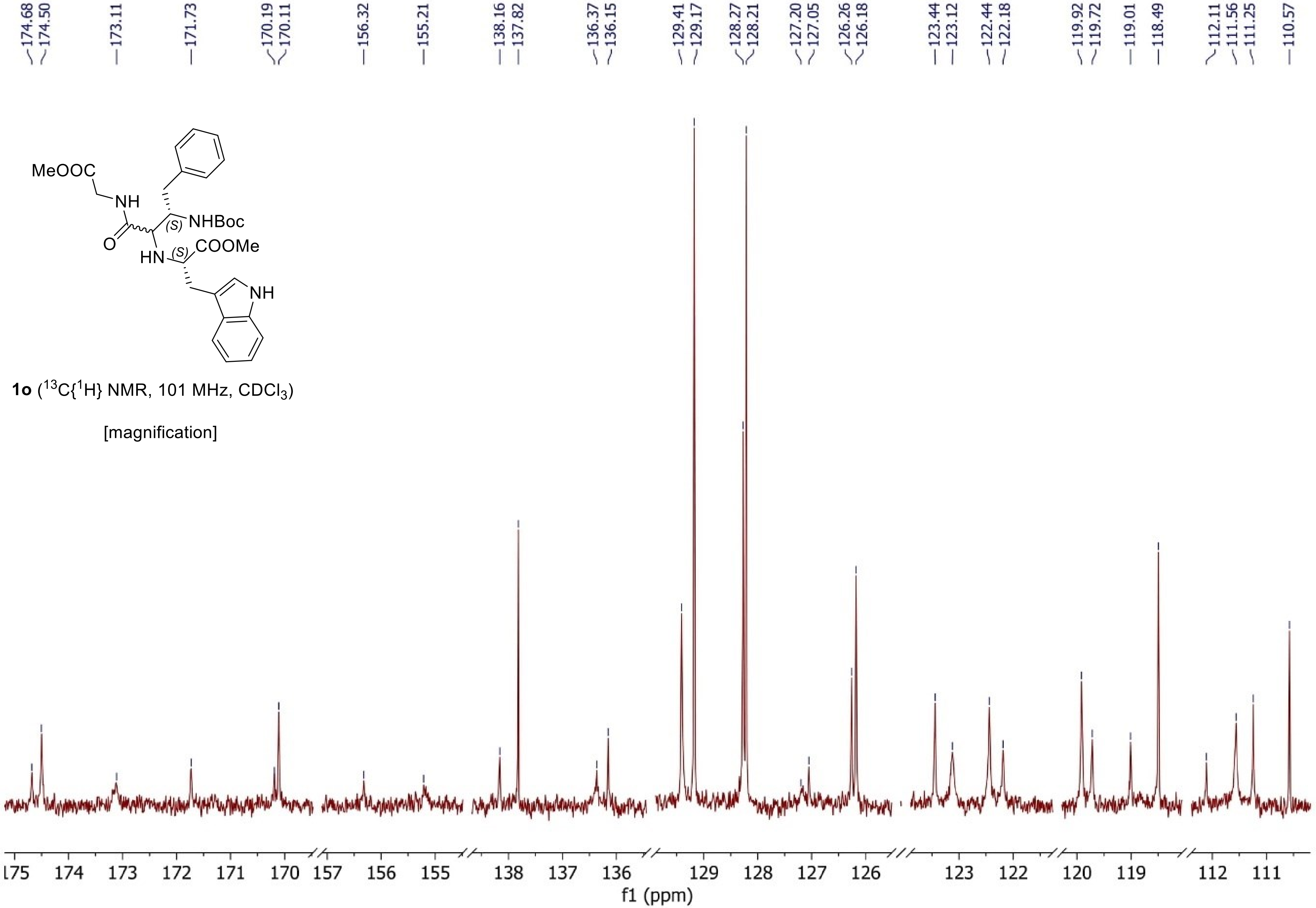

1o ($^{13}C\{^{1}H\}$ NMR, 101 MHz, $CDCl_3$)

[magnification]

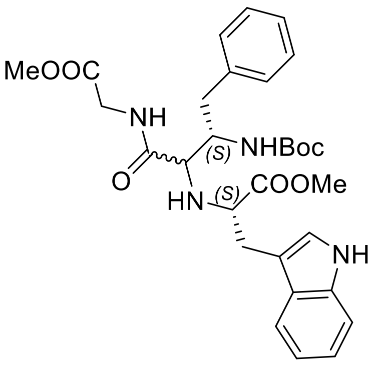

1o ($^{13}C\{^{1}H\}$ NMR, 101 MHz, $CDCl_3$)

[magnification]

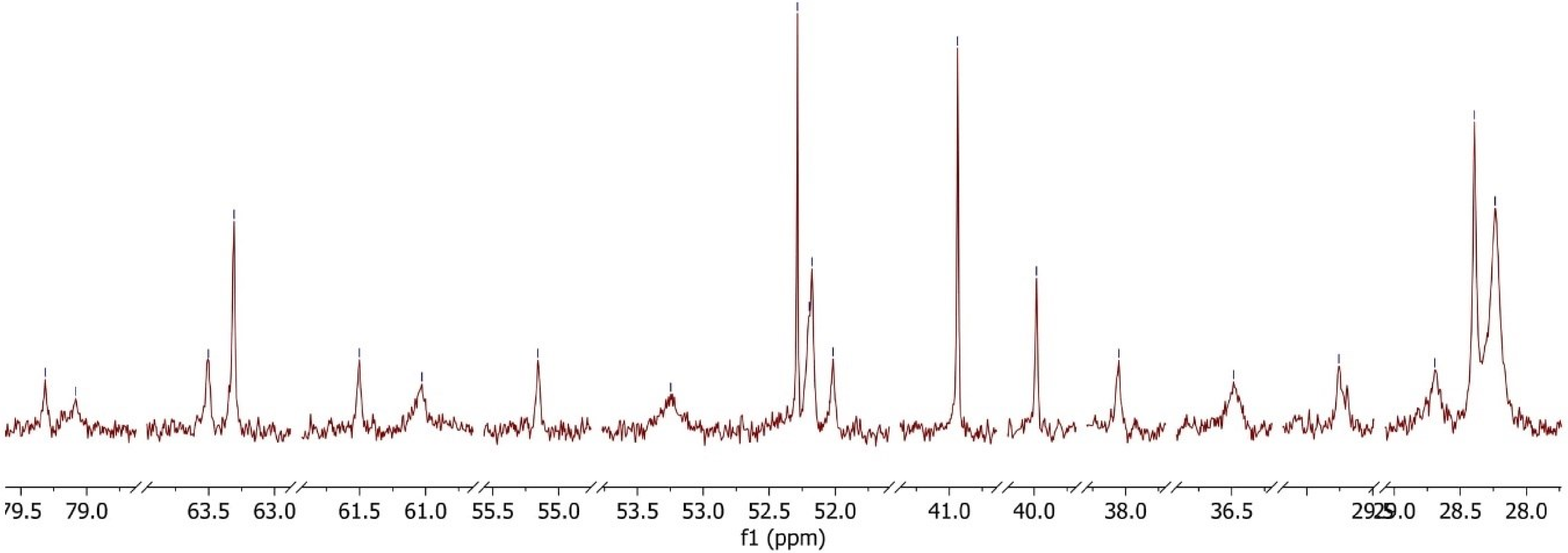

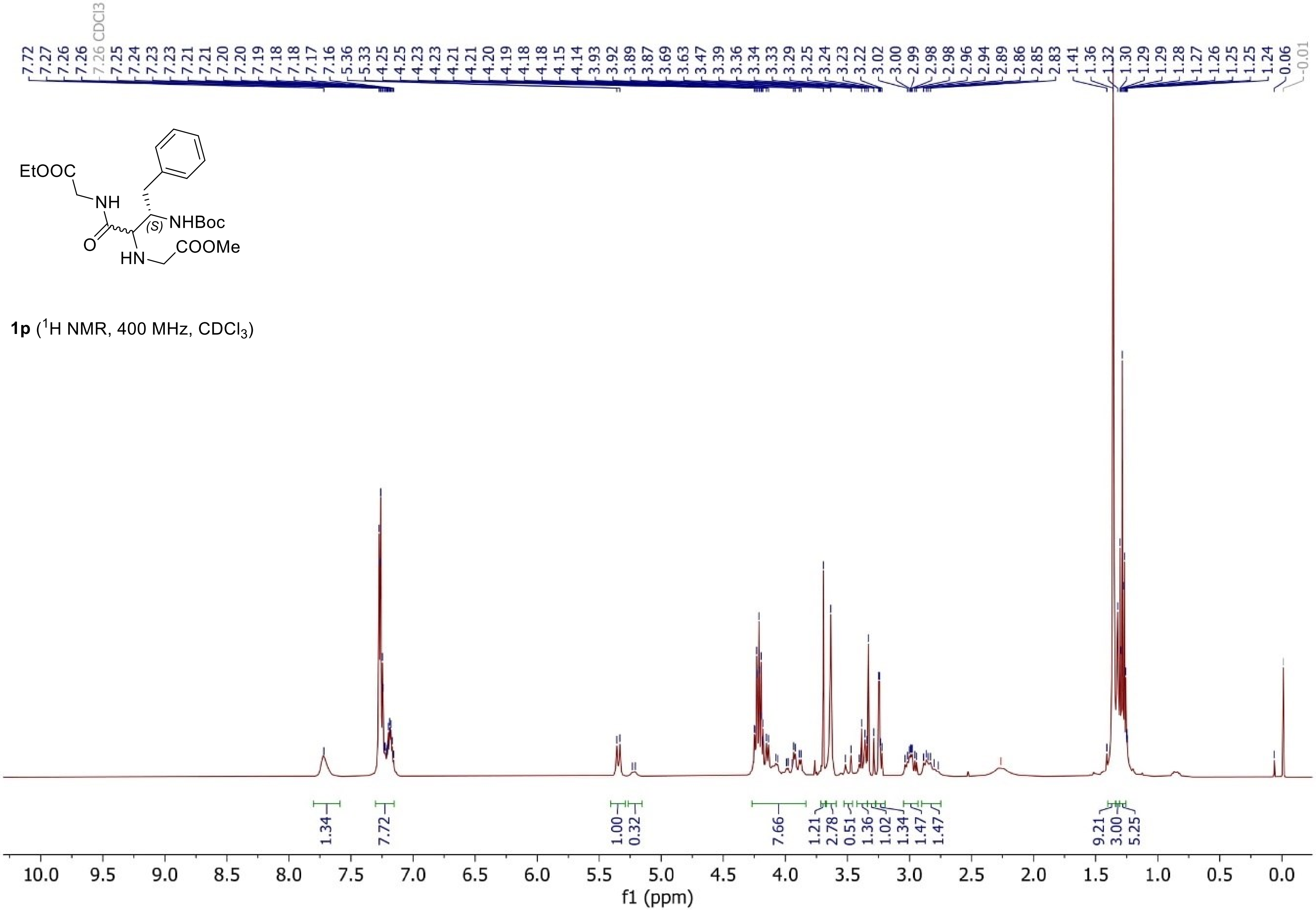

1p (^{1}H NMR, 400 MHz, $CDCl_3$)

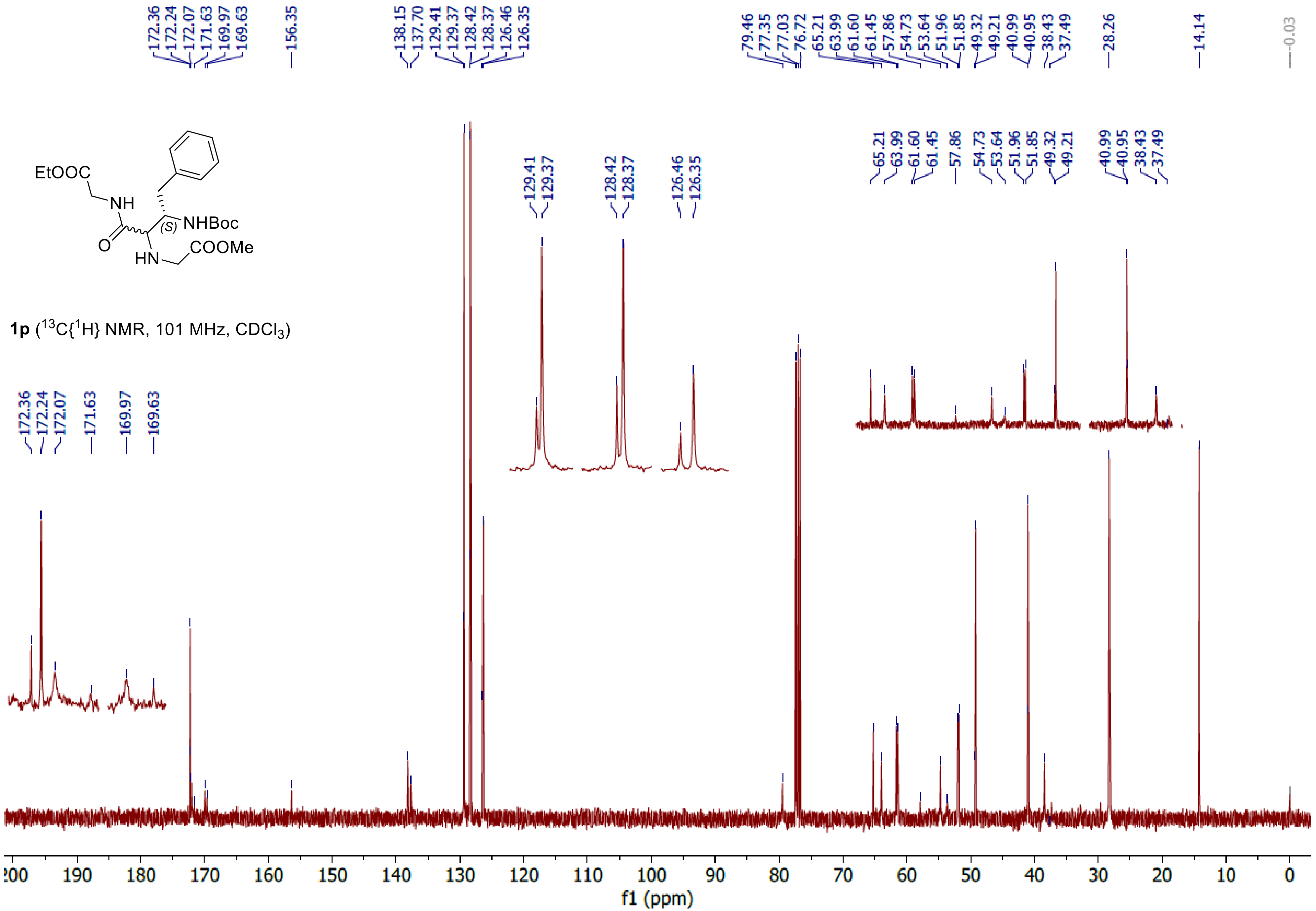
172.36
172.24
172.07
171.63
169.97
169.63
156.35
138.15
137.70
129.41
129.37
128.42
128.37
126.46
126.35
79.46
77.35
77.03
76.72
65.21
63.99
61.60
61.45
57.86
54.73
53.64
51.96
51.85
49.32
49.21
40.99
40.95
38.43
37.49
28.26
14.14
-0.03
EtOOC
NH
O
(S)
NHBoc
HN
COOMe
1p ($^{13}C\{^{1}H\}$ NMR, 101 MHz, $CDCl_3$)
f1 (ppm)

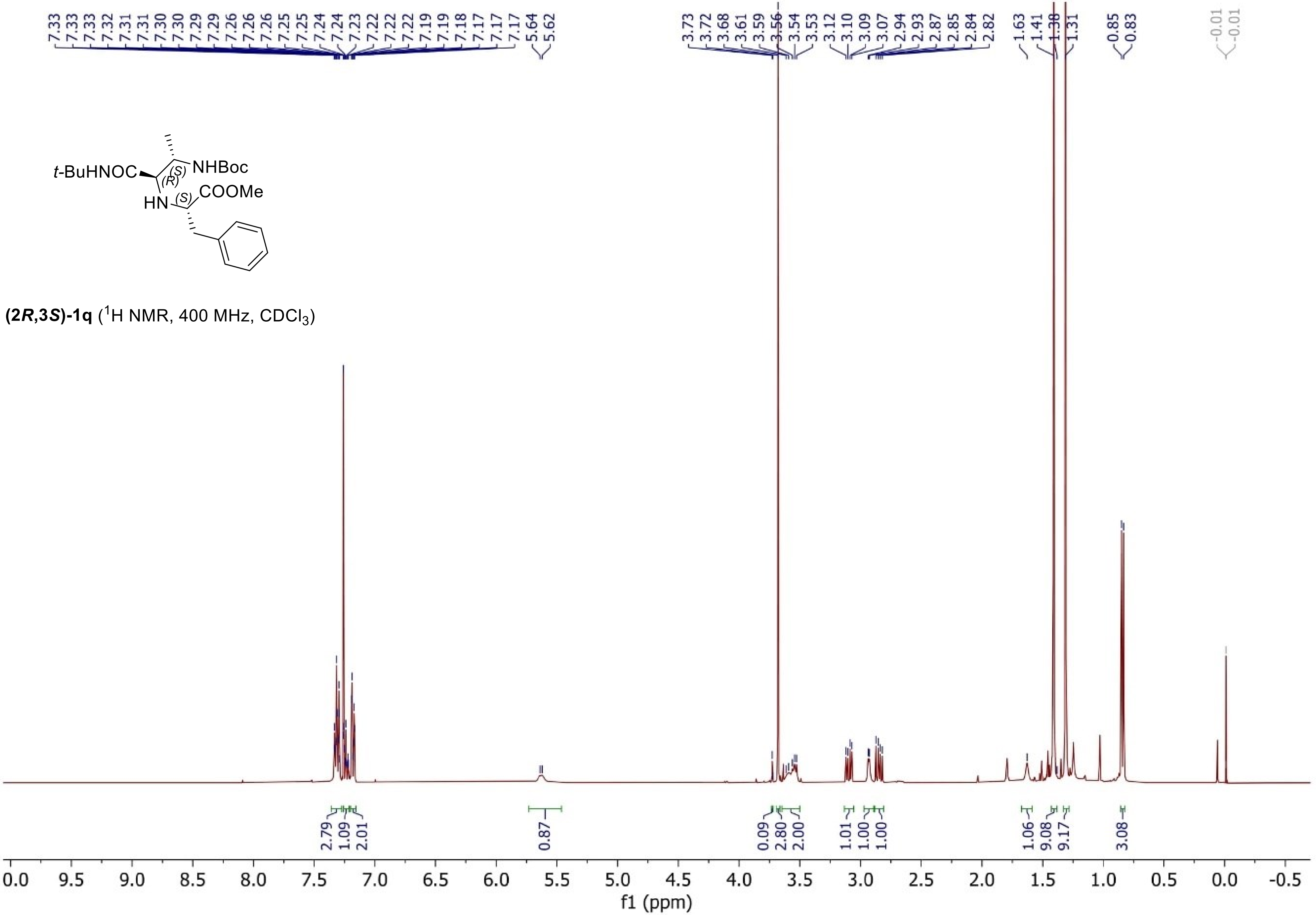

t-BuHNOC
NHBoc
(S)
(R)
COOMe
HN
(S)
(2R,3S)-1q (1H NMR, 400 MHz, CDCl3)
7.33
7.33
7.33
7.32
7.31
7.31
7.30
7.29
7.29
7.26
7.26
7.26
7.25
7.25
7.24
7.24
7.23
7.22
7.22
7.22
7.19
7.19
7.18
7.17
7.17
7.17
5.64
5.62
3.73
3.72
3.68
3.61
3.59
3.56
3.54
3.53
3.12
3.10
3.09
3.07
2.94
2.93
2.87
2.85
2.84
2.82
1.63
1.41
1.38
1.31
0.85
0.83
-0.01
-0.01
2.79
1.09
2.01
0.87
0.09
2.80
2.00
1.01
1.00
1.00
1.06
9.08
9.17
3.08
0.0
9.5
9.0
8.5
8.0
7.5
7.0
6.5
6.0
5.5
5.0
4.5
4.0
3.5
3.0
2.5
2.0
1.5
1.0
0.5
0.0
-0.5
f1 (ppm)

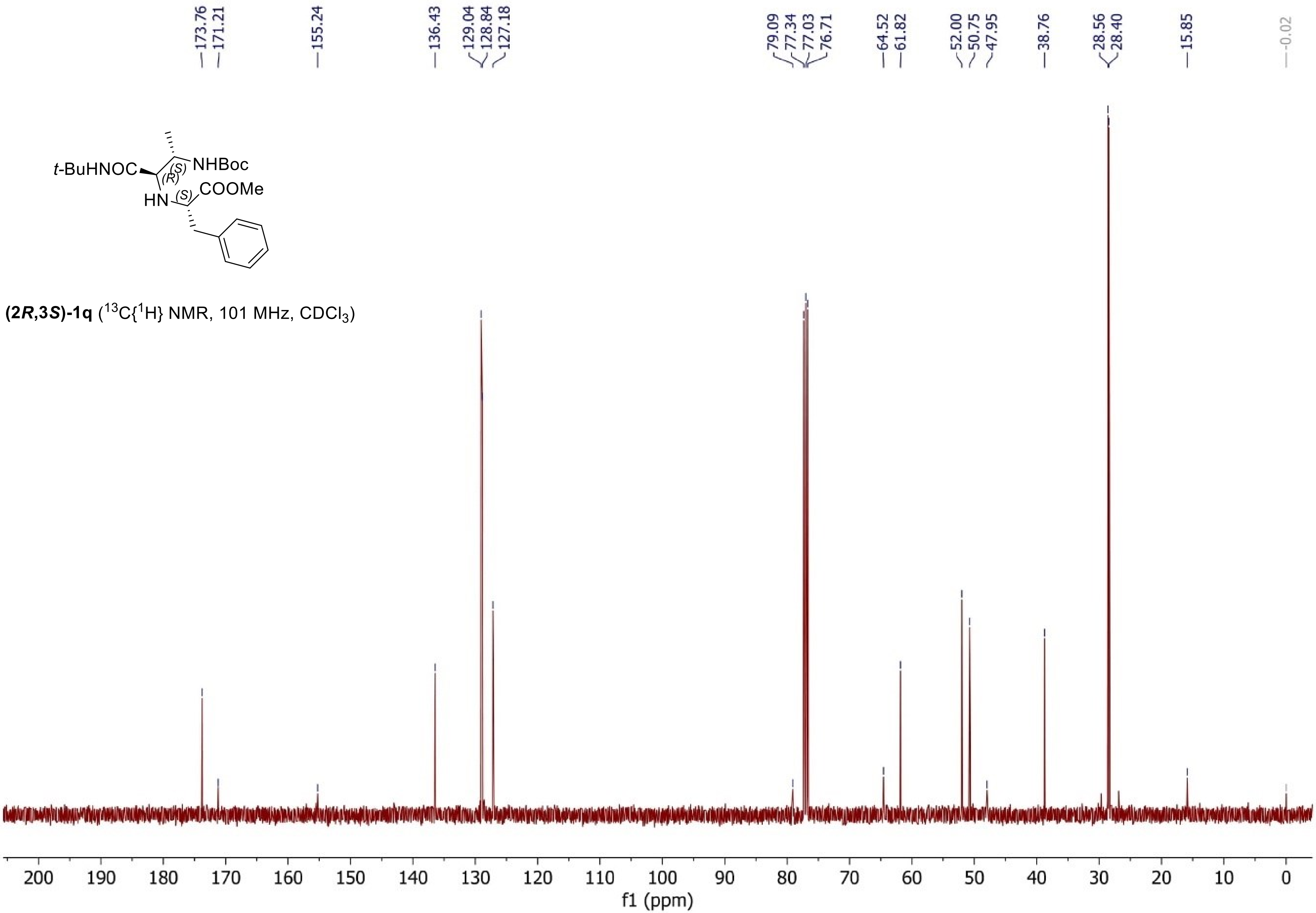

(2*R*,3*S*)-1q ($^{13}C\{^{1}H\}$ NMR, 101 MHz, $CDCl_3$)

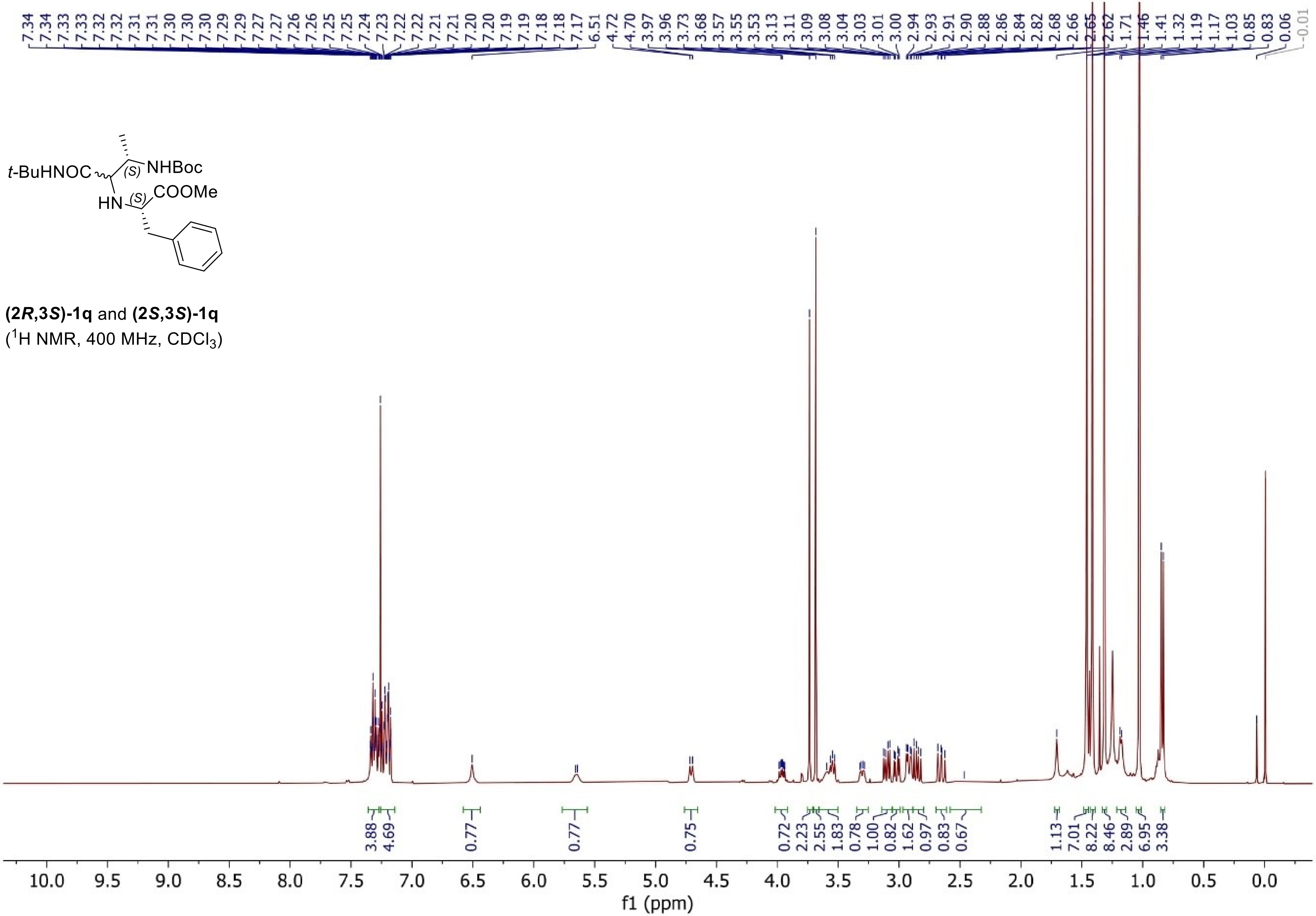

(2*R*,3*S*)-1q and **(2*S*,3*S*)-1q**
(^{1}H NMR, 400 MHz, $CDCl_3$)

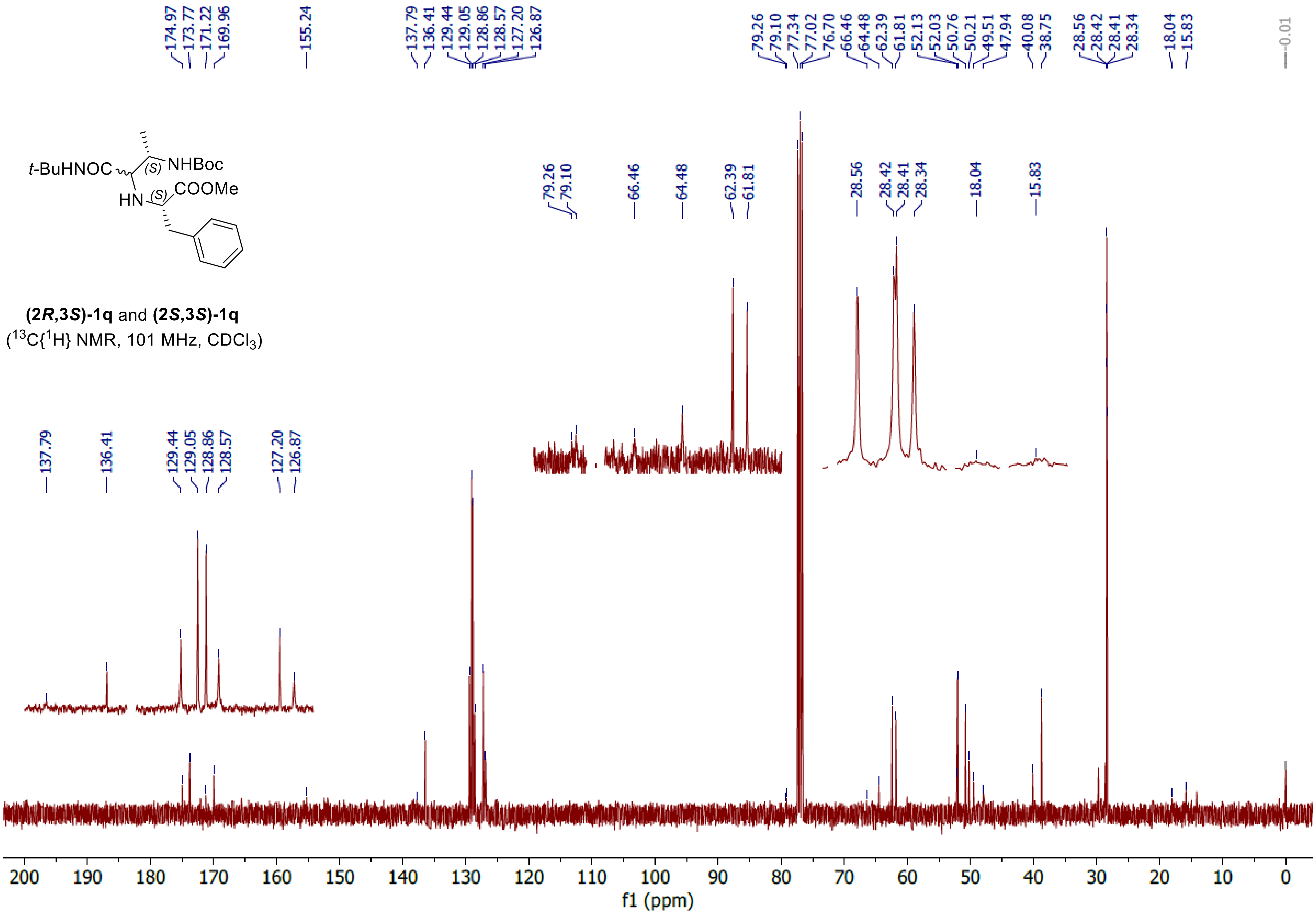

(2*R*,3*S*)-1q and **(2*S*,3*S*)-1q**
($^{13}C\{^{1}H\}$ NMR, 101 MHz, $CDCl_3$)

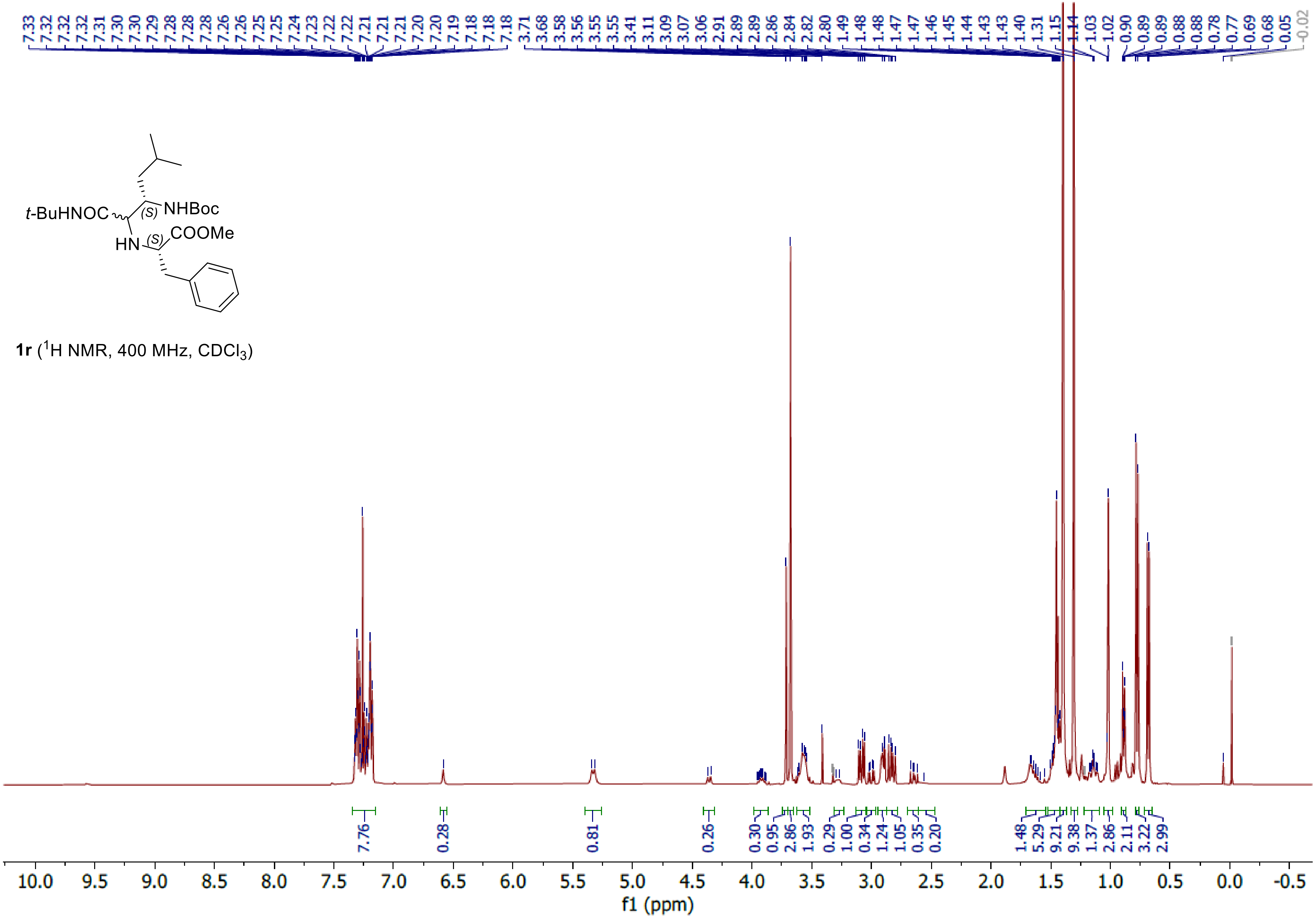

1r (^{1}H NMR, 400 MHz, $CDCl_3$)

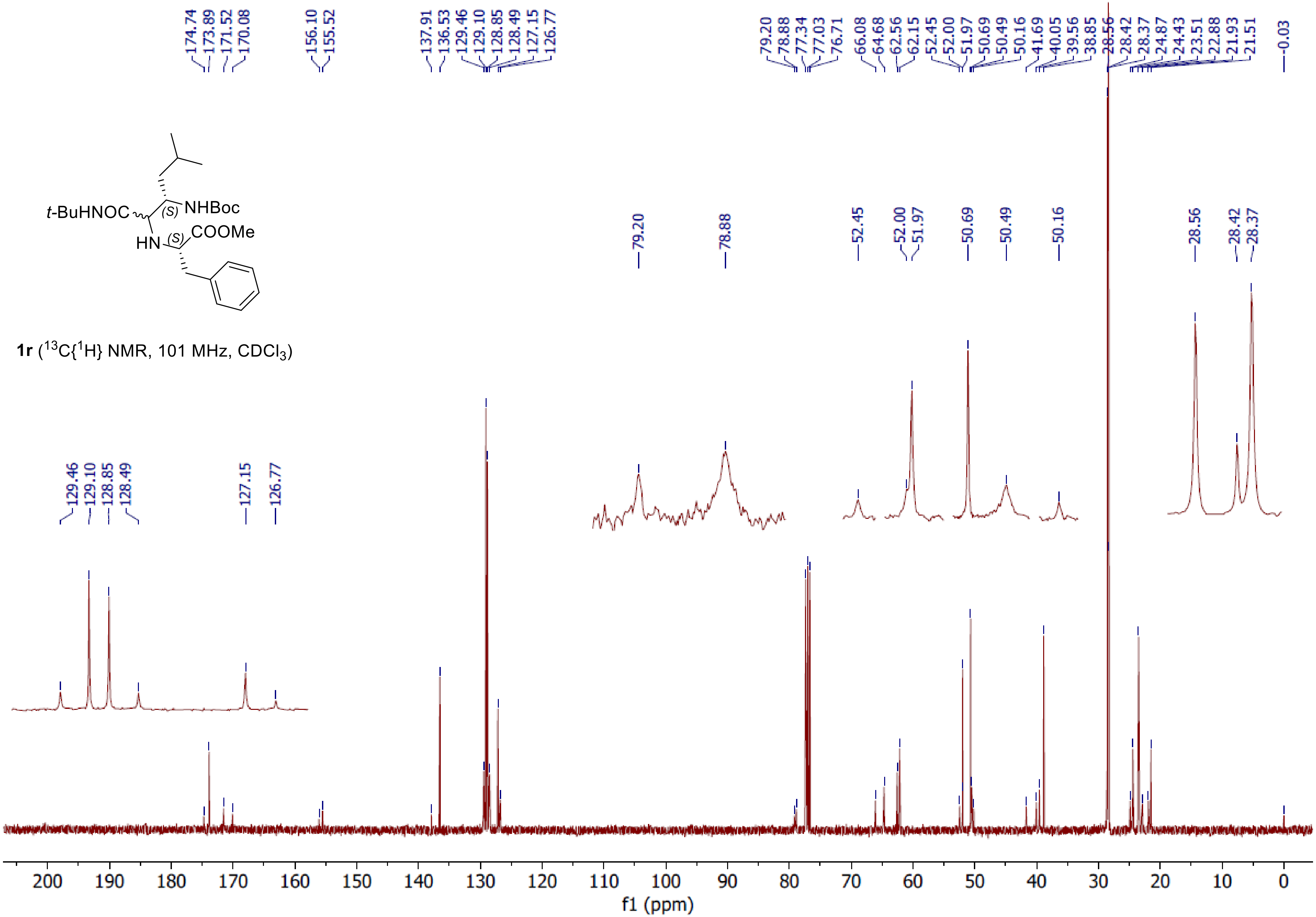

1r ($^{13}C\{^1H\}$ NMR, 101 MHz, $CDCl_3$)

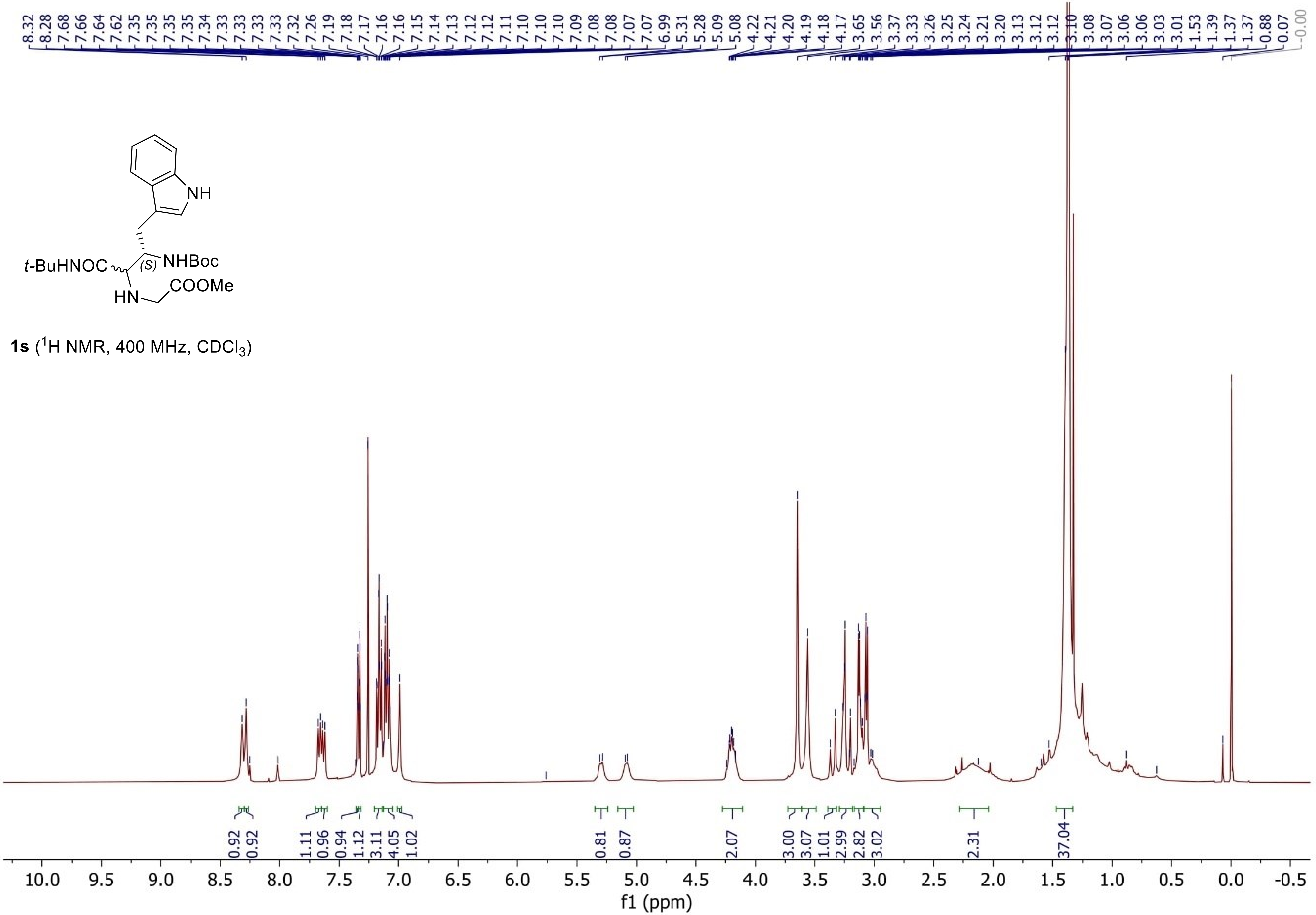

1s (^{1}H NMR, 400 MHz, $CDCl_3$)

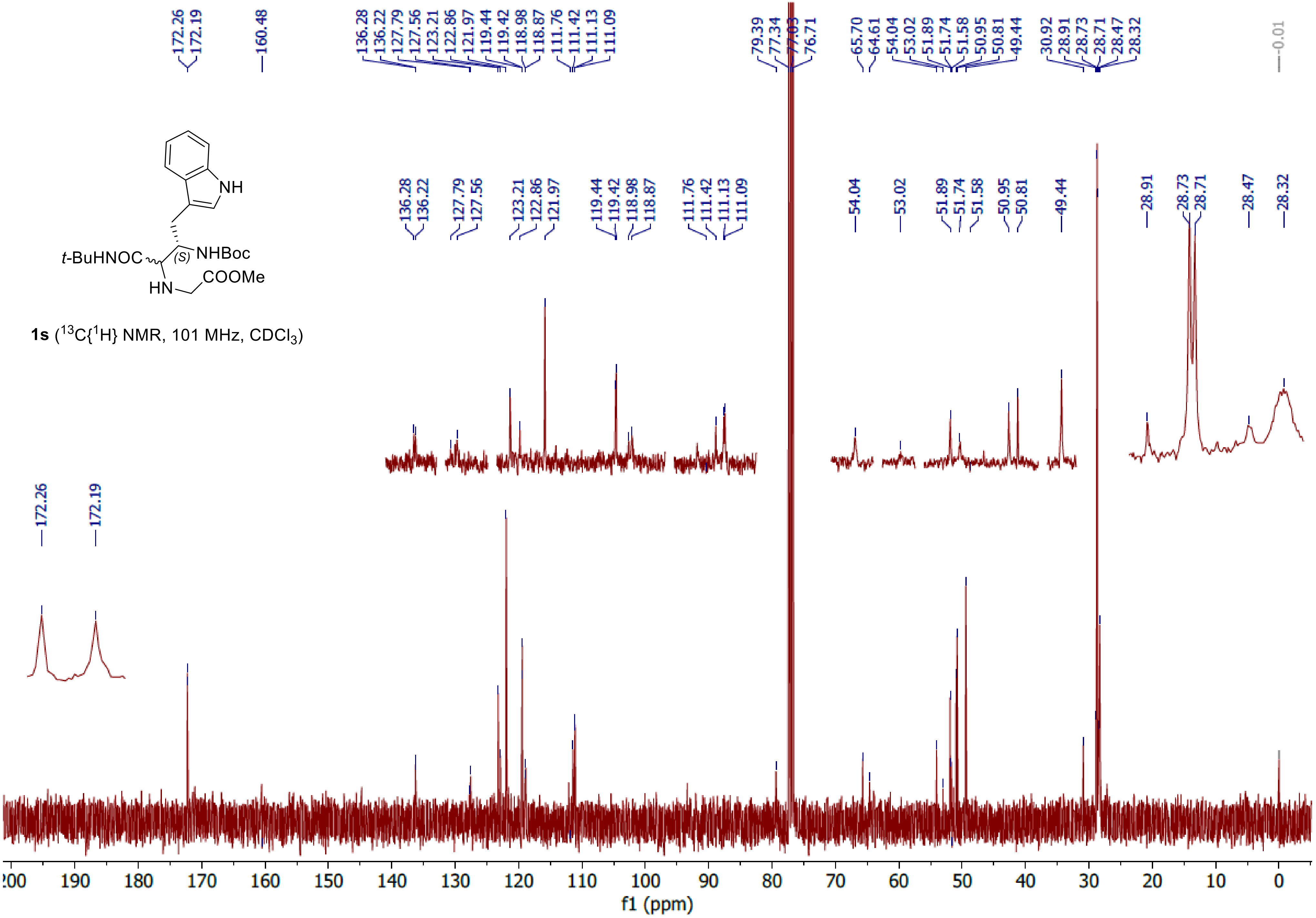

1s ($^{13}C\{^{1}H\}$ NMR, 101 MHz, $CDCl_3$)

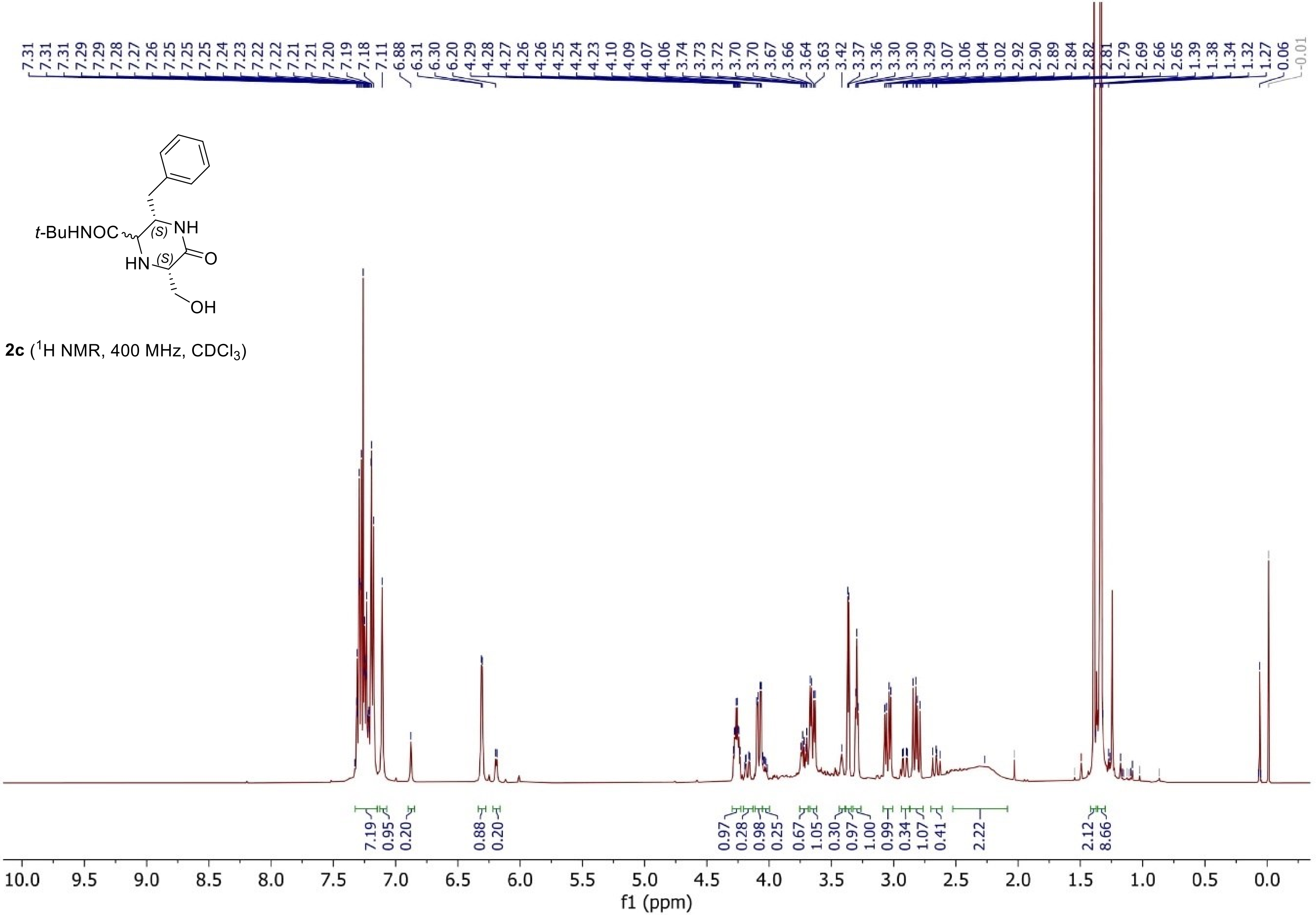

2c (^{1}H NMR, 400 MHz, $CDCl_3$)

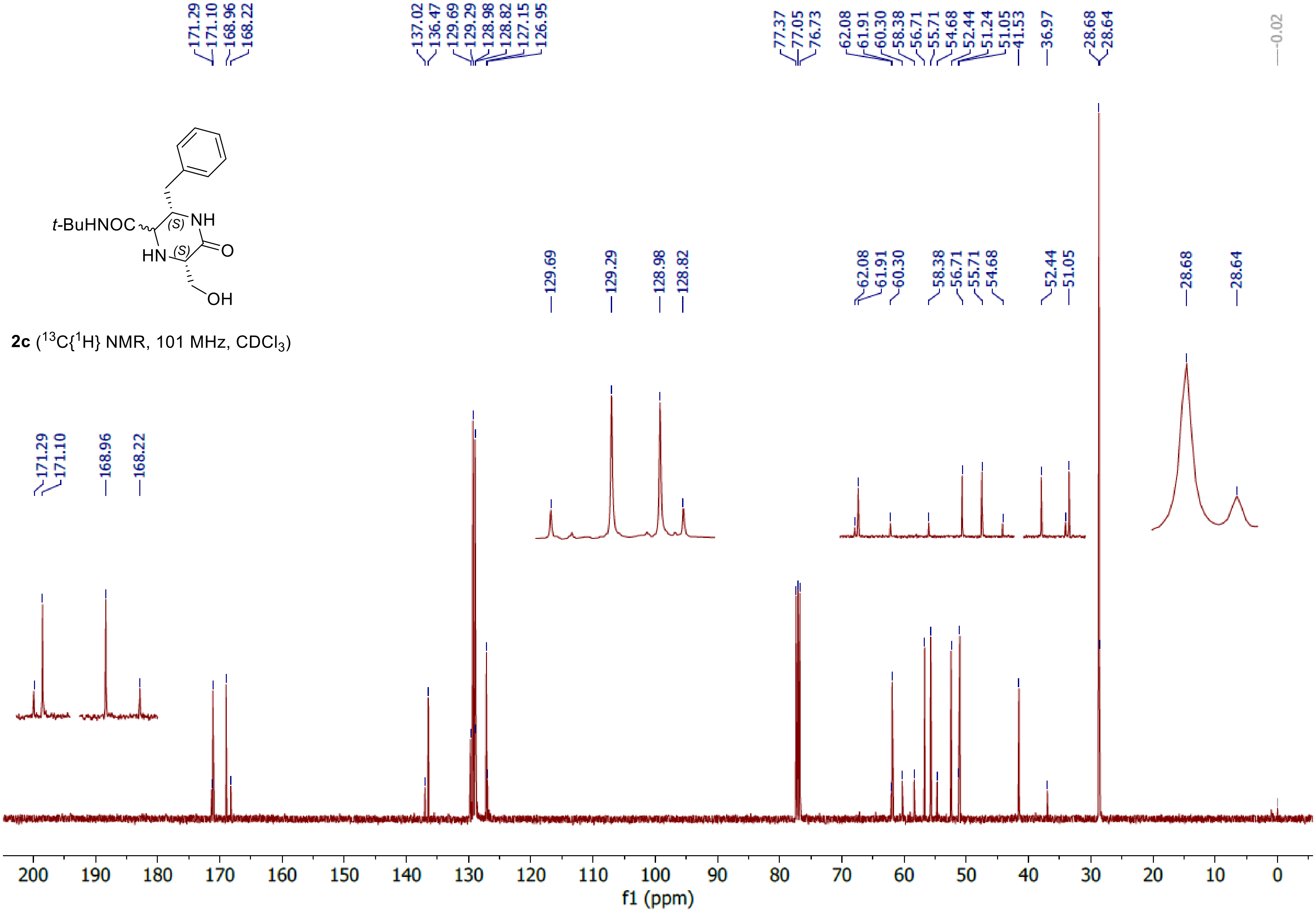
171.29
171.10
168.96
168.22
137.02
136.47
129.69
129.29
128.98
128.82
127.15
126.95
77.37
77.05
76.73
62.08
61.91
60.30
58.38
56.71
55.71
54.68
52.44
51.24
51.05
41.53
36.97
28.68
28.64
-0.02
t-BuHNOC
(S)
NH
HN
(S)
O
OH
2c (13C{1H} NMR, 101 MHz, CDCl3)
f1 (ppm)

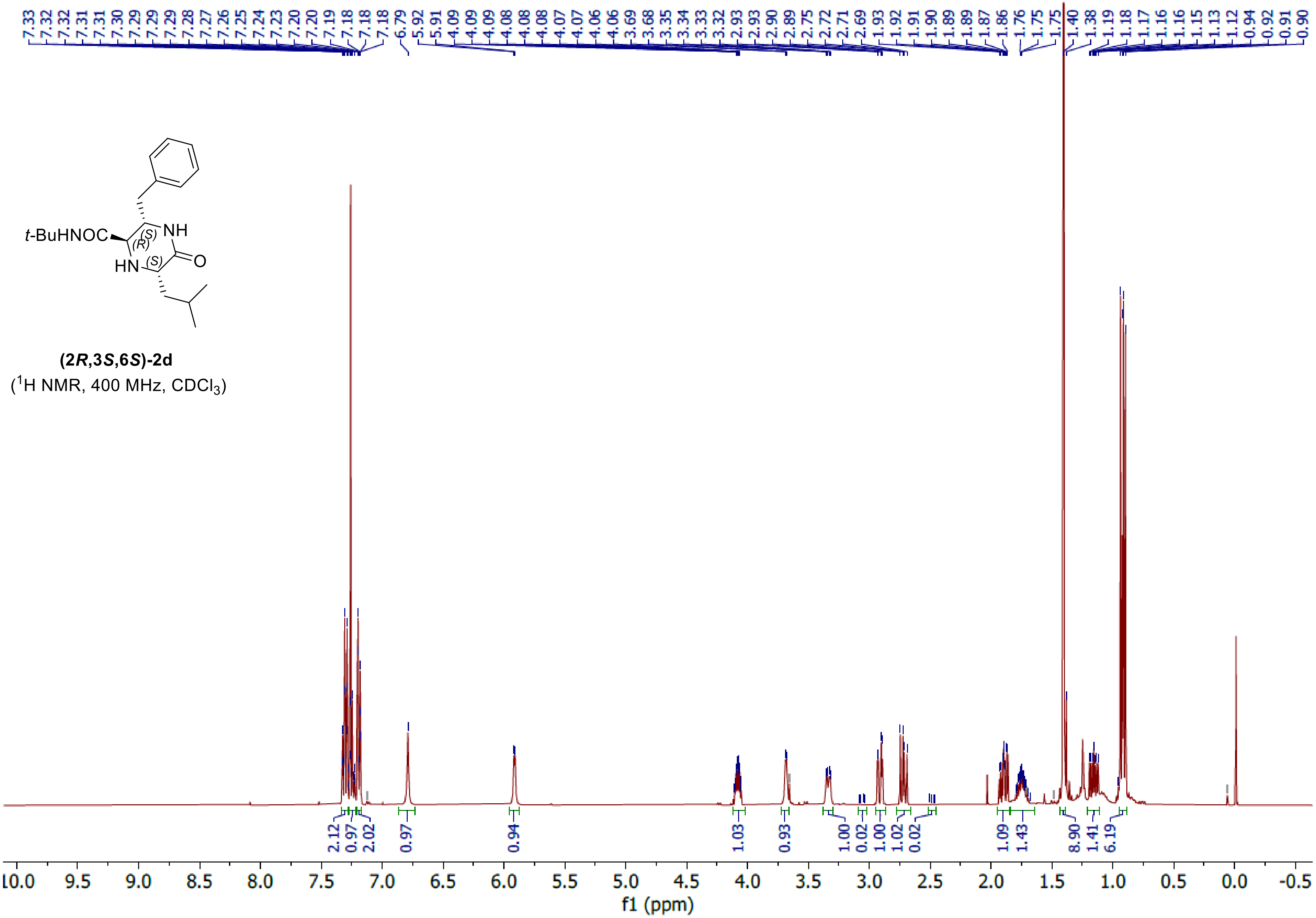

(2*R*,3*S*,6*S*)-2d

(^{1}H NMR, 400 MHz, $CDCl_3$)

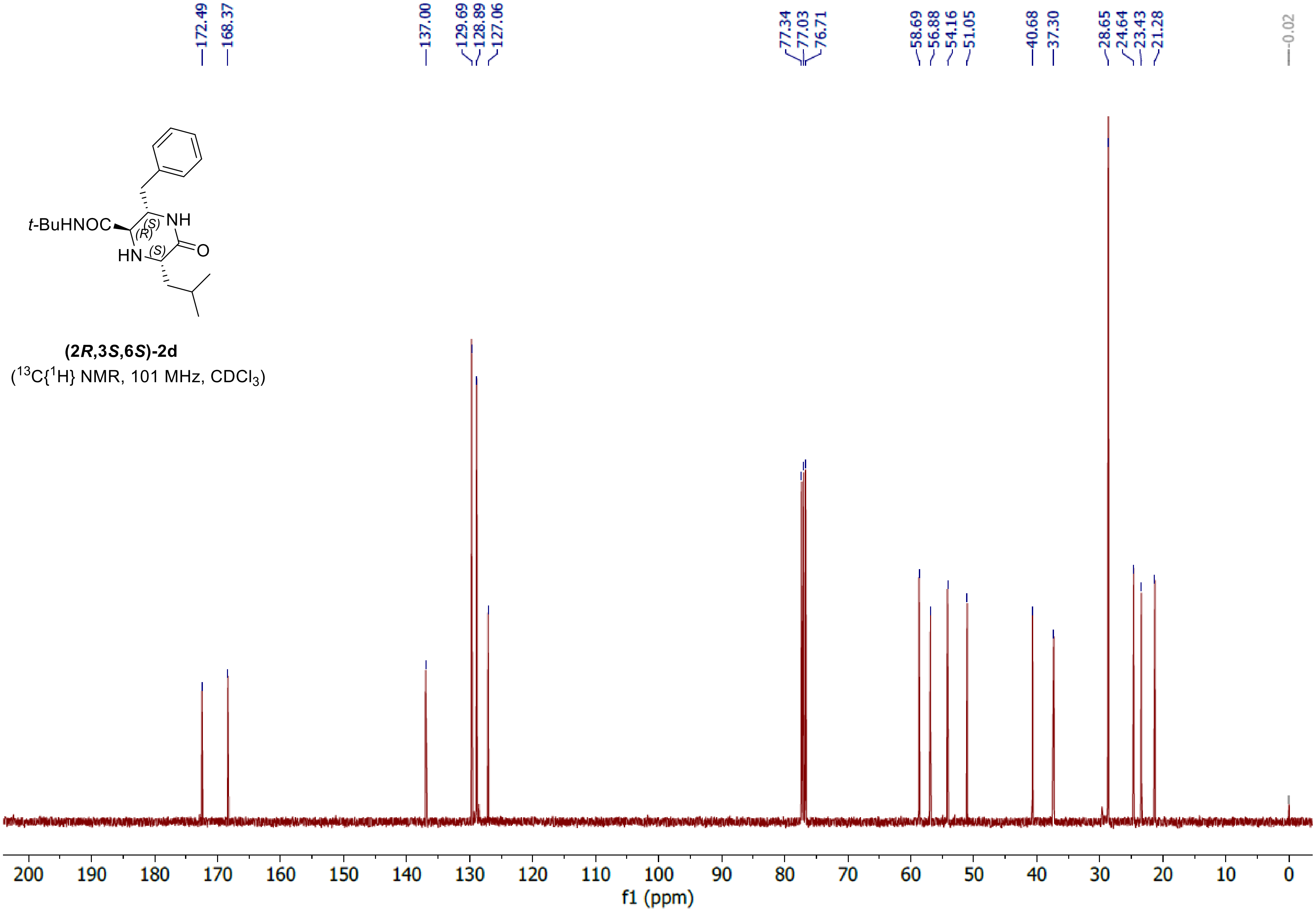
172.49
168.37
137.00
129.69
128.89
127.06
77.34
77.03
76.71
58.69
56.88
54.16
51.05
40.68
37.30
28.65
24.64
23.43
21.28
-0.02
t-BuHNOC
NH
HN
O
(S)
(R)
(S)
(2R,3S,6S)-2d
($^{13}C\{^{1}H\}$ NMR, 101 MHz, $CDCl_3$)
200 190 180 170 160 150 140 130 120 110 100 90 80 70 60 50 40 30 20 10 0
f1 (ppm)

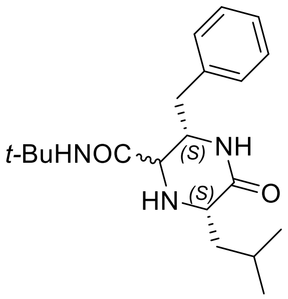

(2*R*,3*S*,6*S*)-2d and **(2*S*,3*S*,6*S*)-2d**
(^{1}H NMR, 400 MHz, $CDCl_3$)

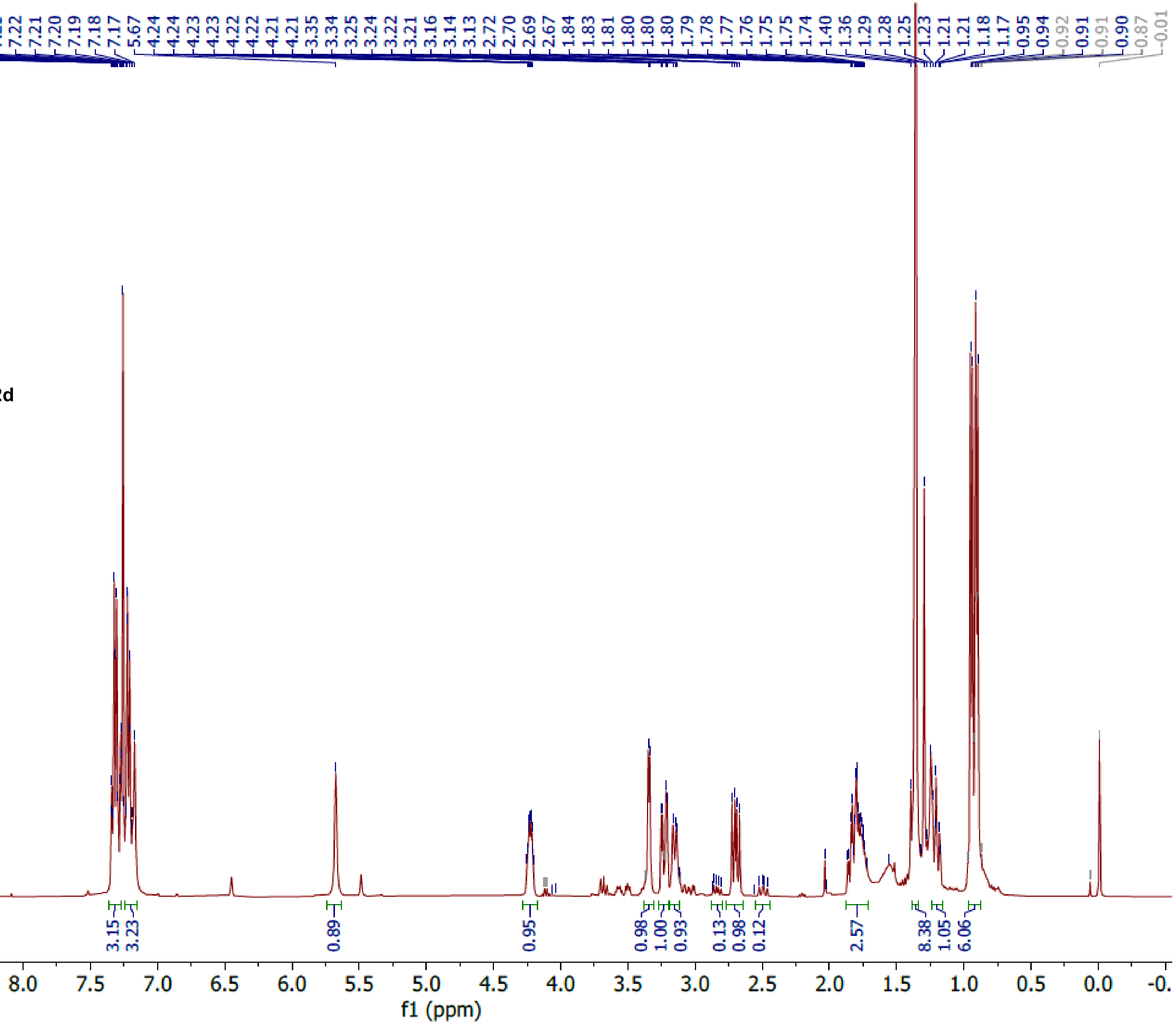

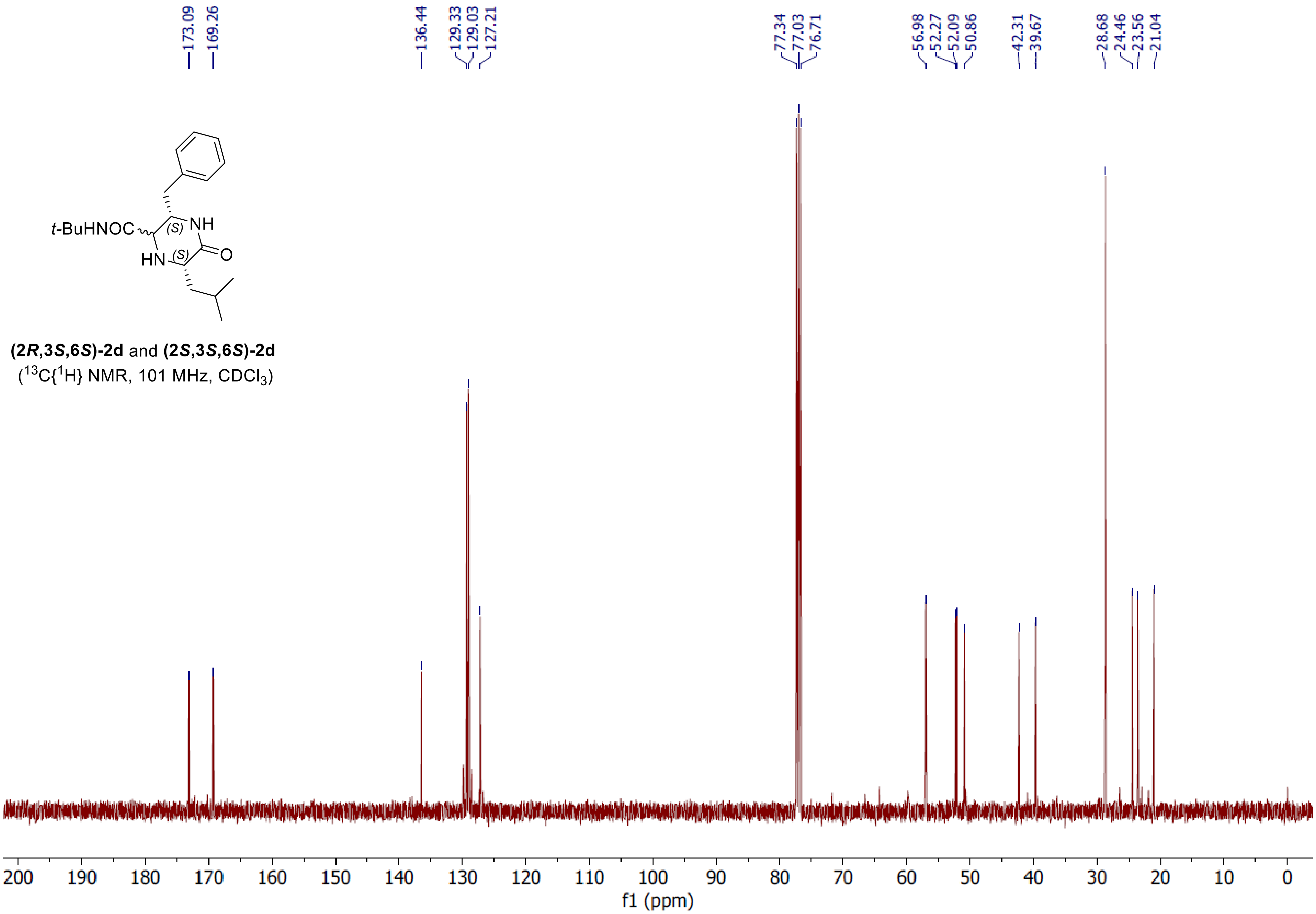
173.09
169.26
136.44
129.33
129.03
127.21
77.34
77.03
76.71
56.98
52.27
52.09
50.86
42.31
39.67
28.68
24.46
23.56
21.04
t-BuHNOC
(S)
NH
HN
(S)
O
(2R,3S,6S)-2d and (2S,3S,6S)-2d
(13C{1H} NMR, 101 MHz, CDCl3)
200
190
180
170
160
150
140
130
120
110
100
90
80
70
60
50
40
30
20
10
0
f1 (ppm)

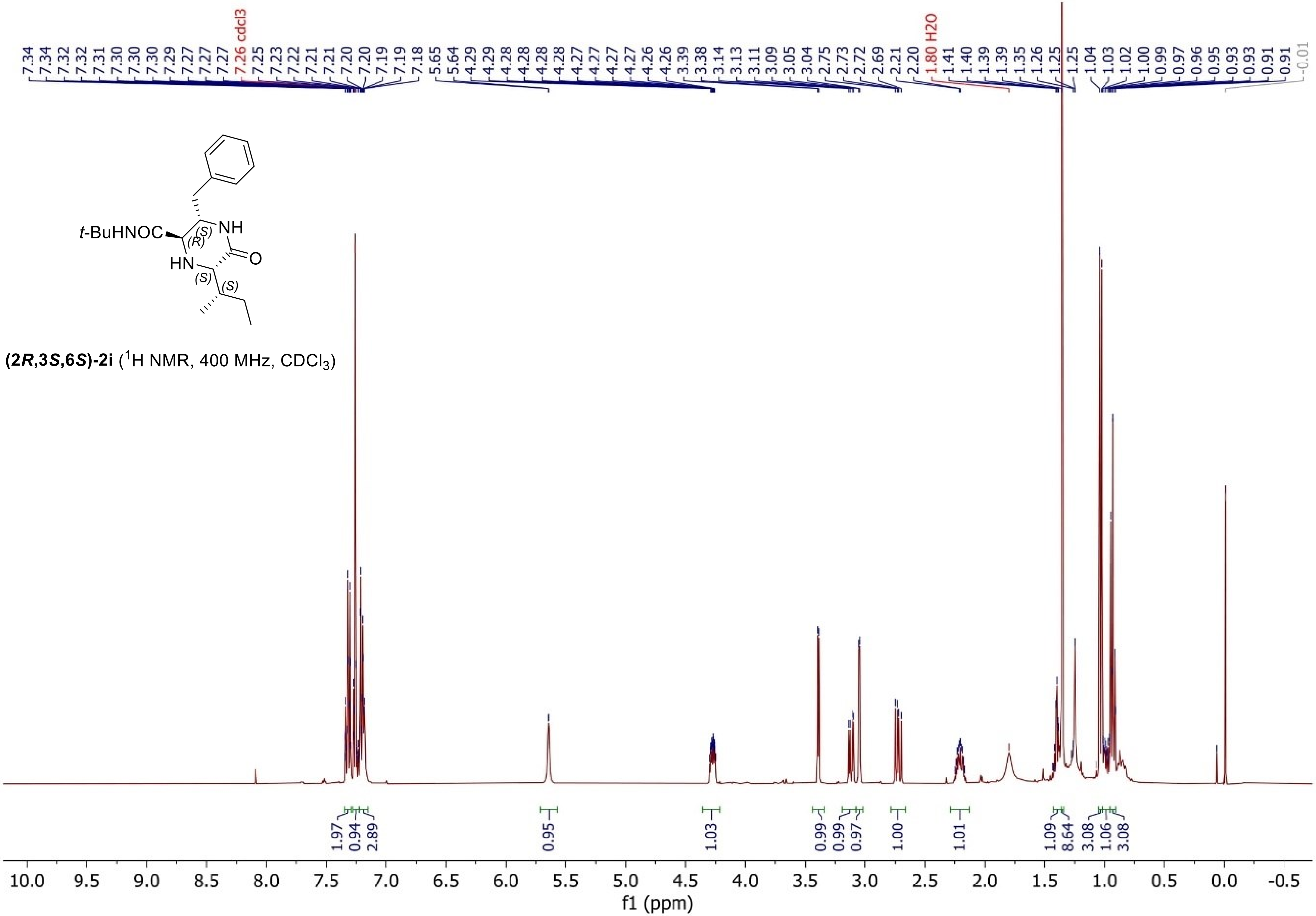

(2*R*,3*S*,6*S*)-2i (^1H NMR, 400 MHz, $CDCl_3$)

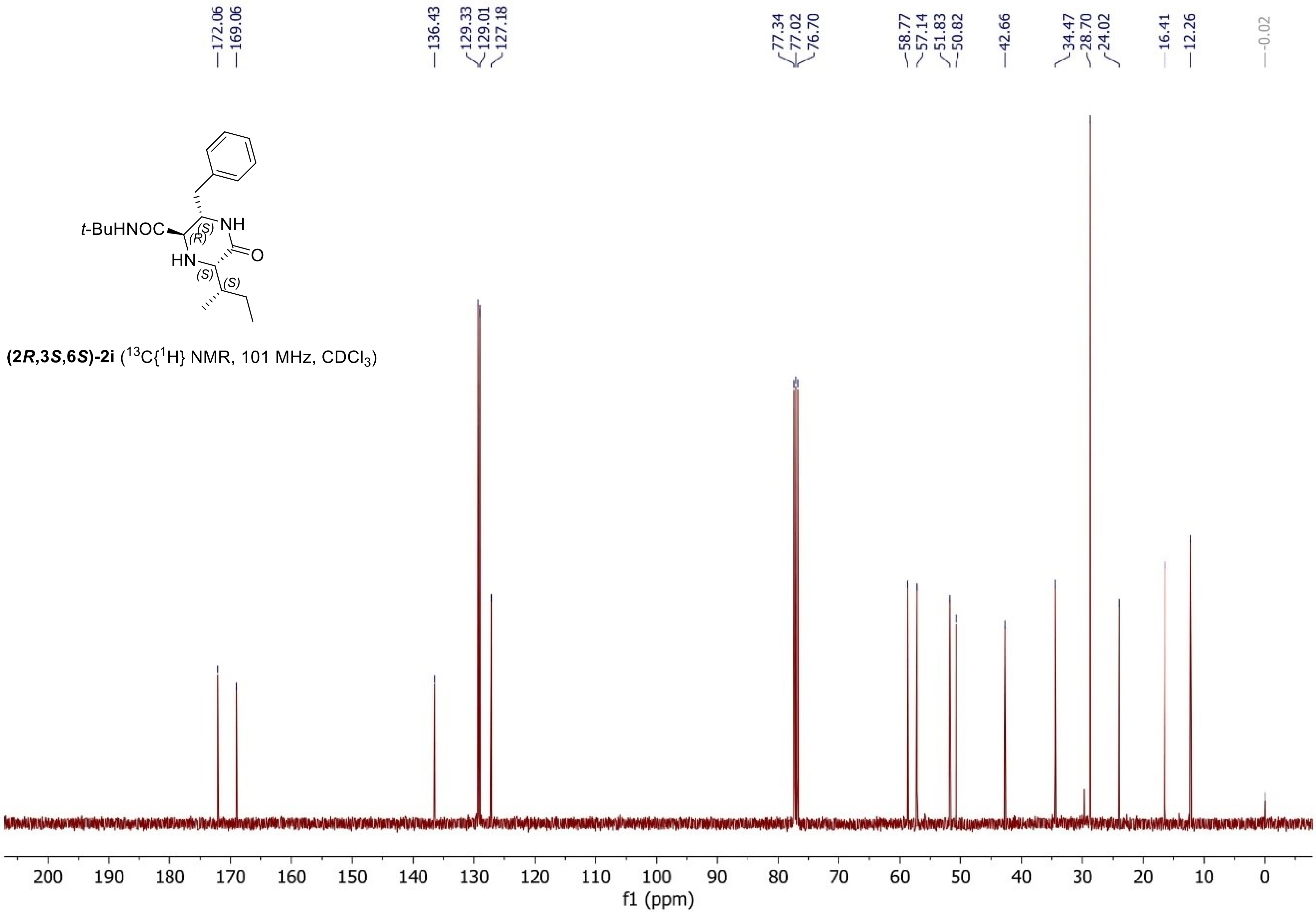

(2*R*,3*S*,6*S*)-2i ($^{13}C\{^{1}H\}$ NMR, 101 MHz, $CDCl_3$)

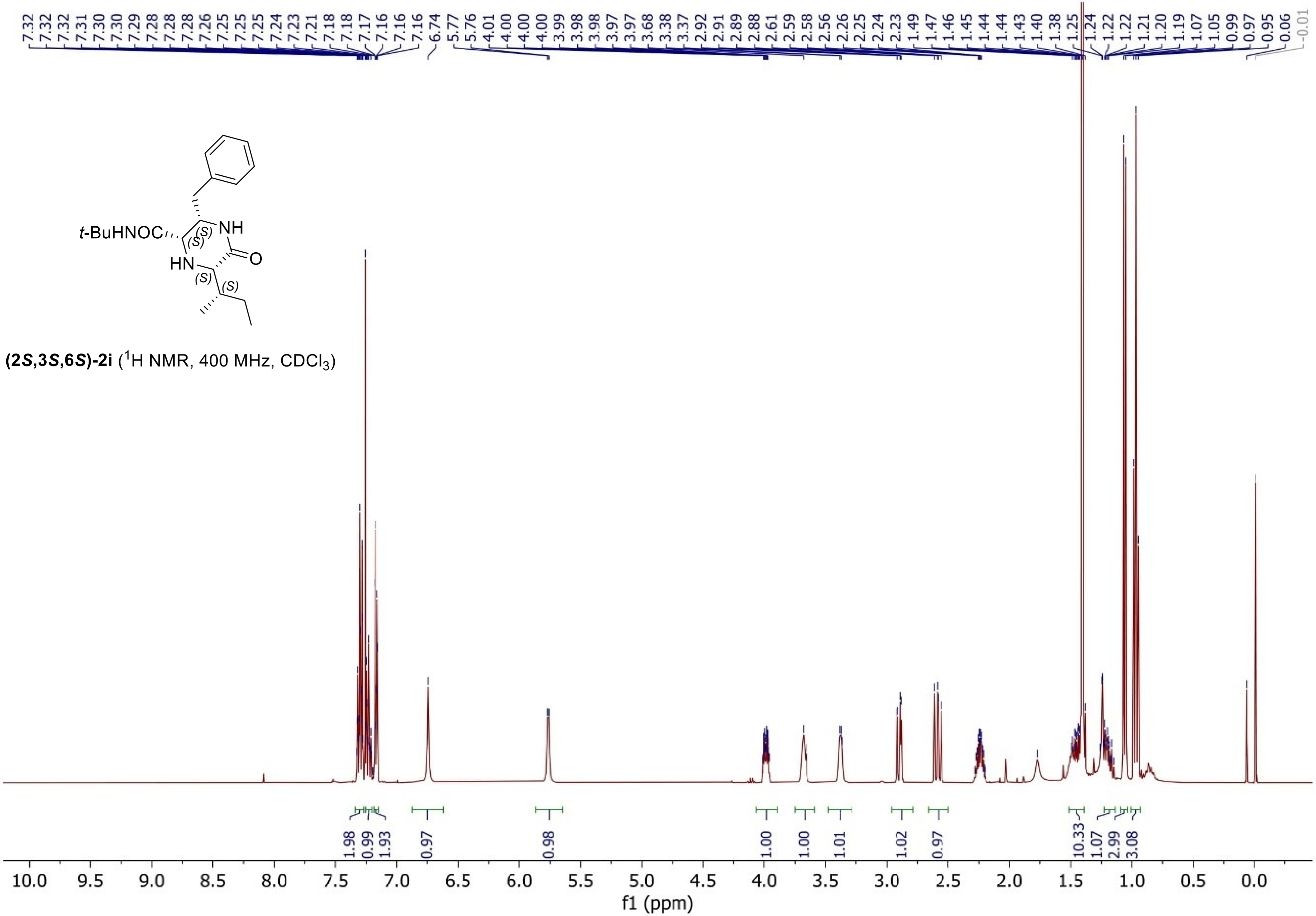

(2*S*,3*S*,6*S*)-2i (^{1}H NMR, 400 MHz, $CDCl_3$)

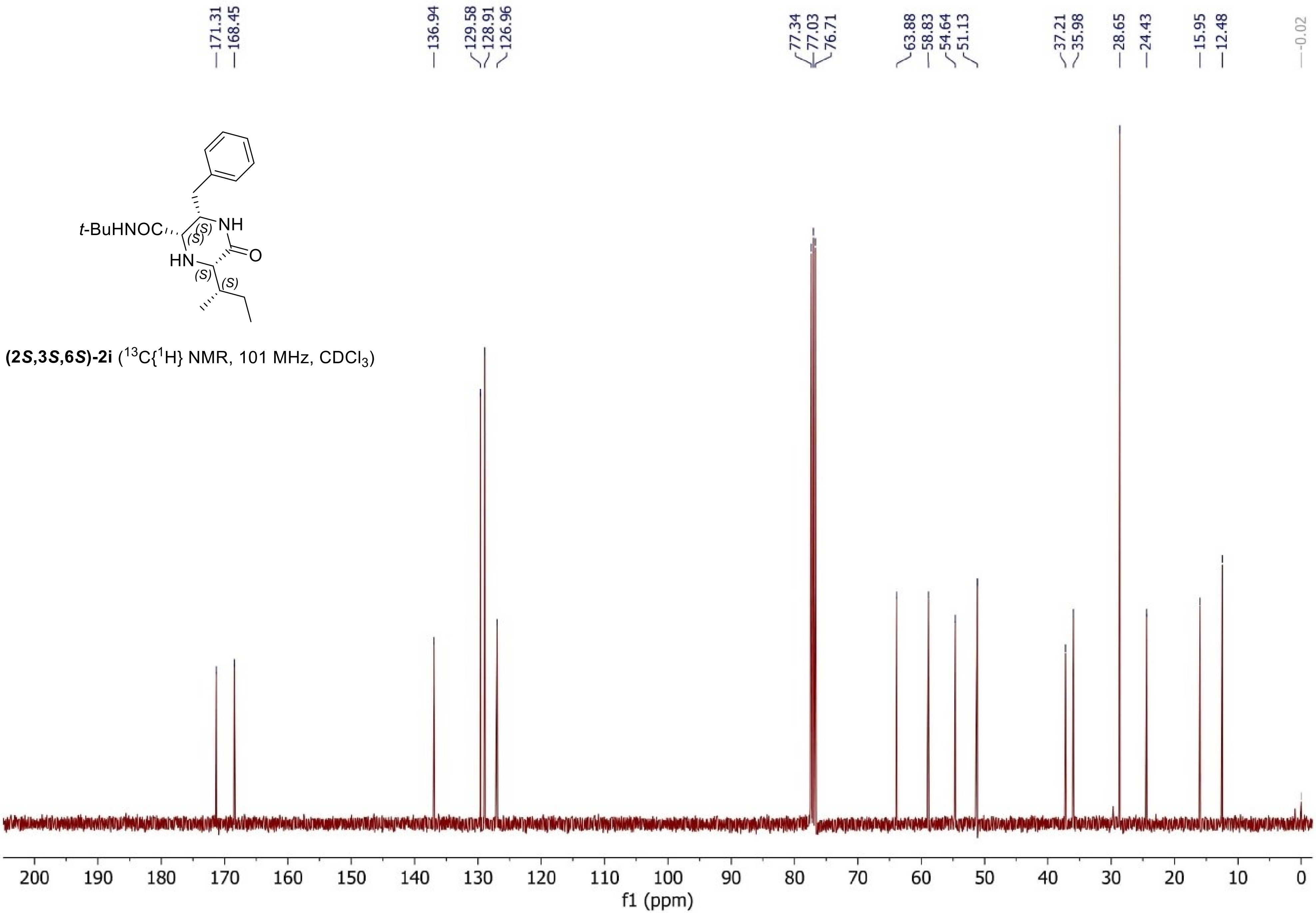

(2*S*,3*S*,6*S*)-2i ($^{13}C\{^{1}H\}$ NMR, 101 MHz, $CDCl_3$)

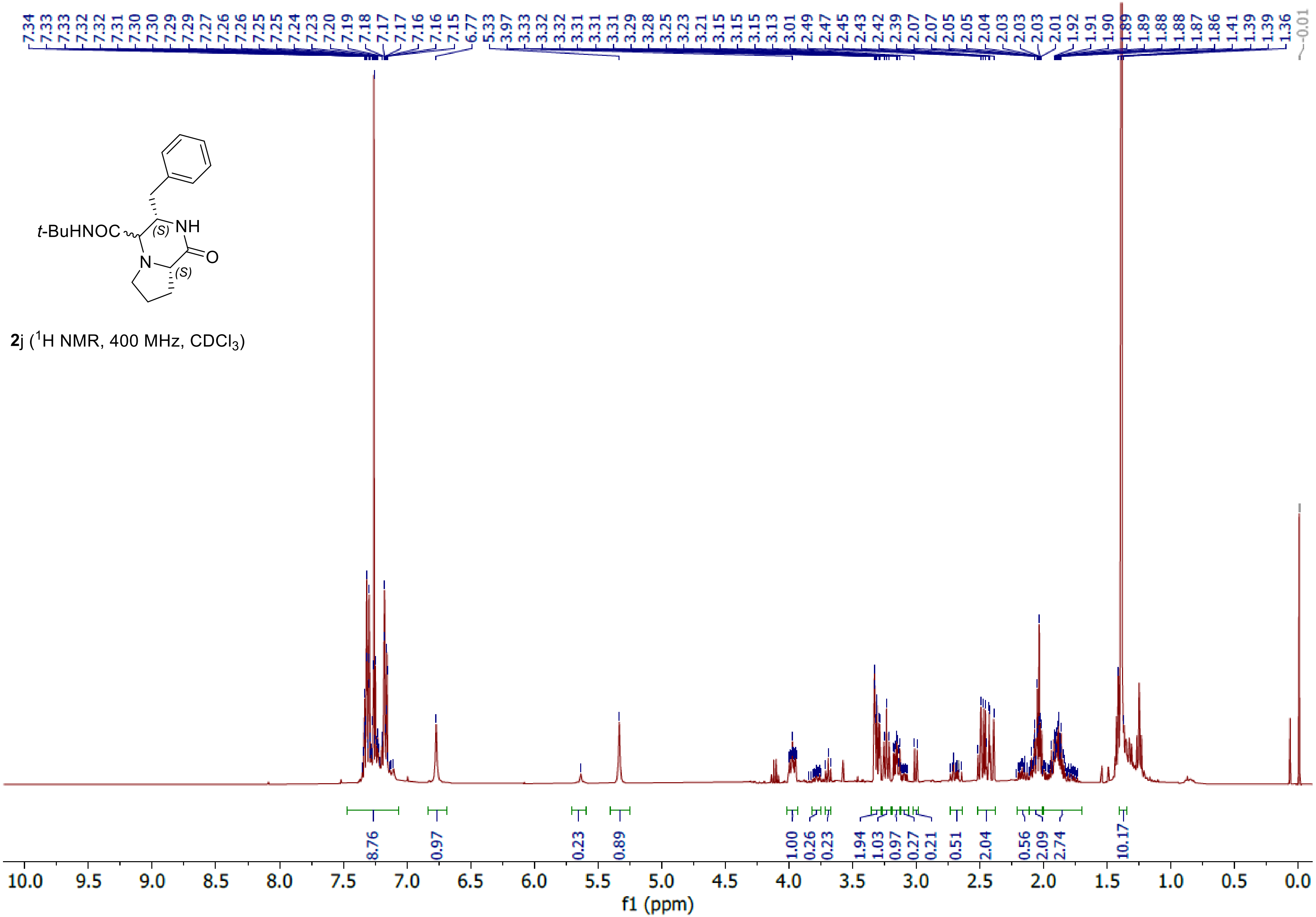

2j (^{1}H NMR, 400 MHz, $CDCl_3$)

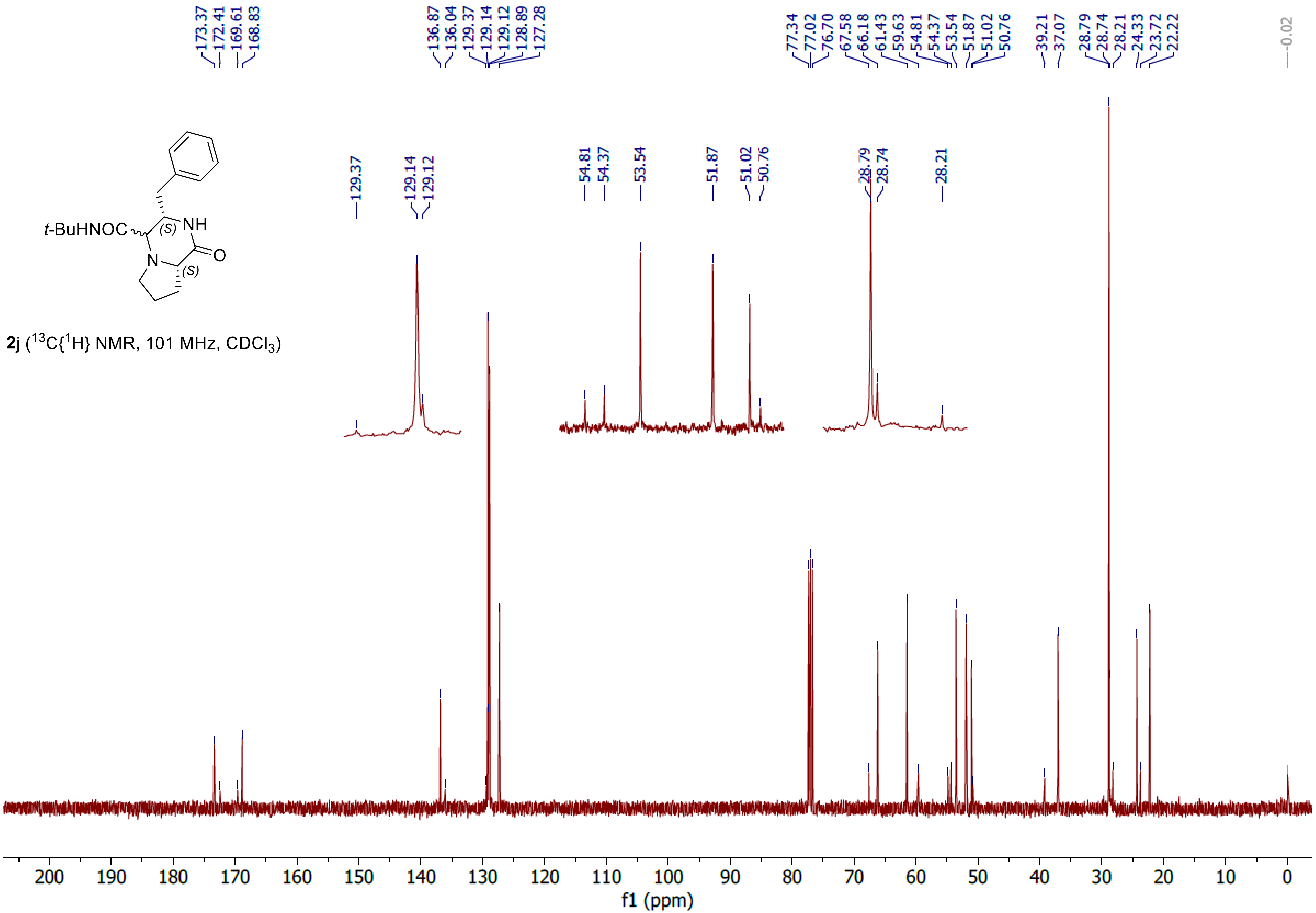

2j ($^{13}C\{^{1}H\}$ NMR, 101 MHz, $CDCl_3$)

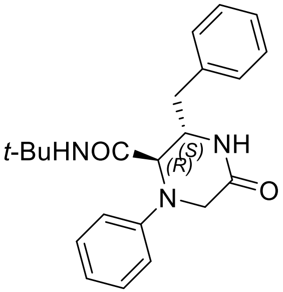

(2*R*,3*S*)-2k (^{1}H NMR, 400 MHz, $CDCl_3$)

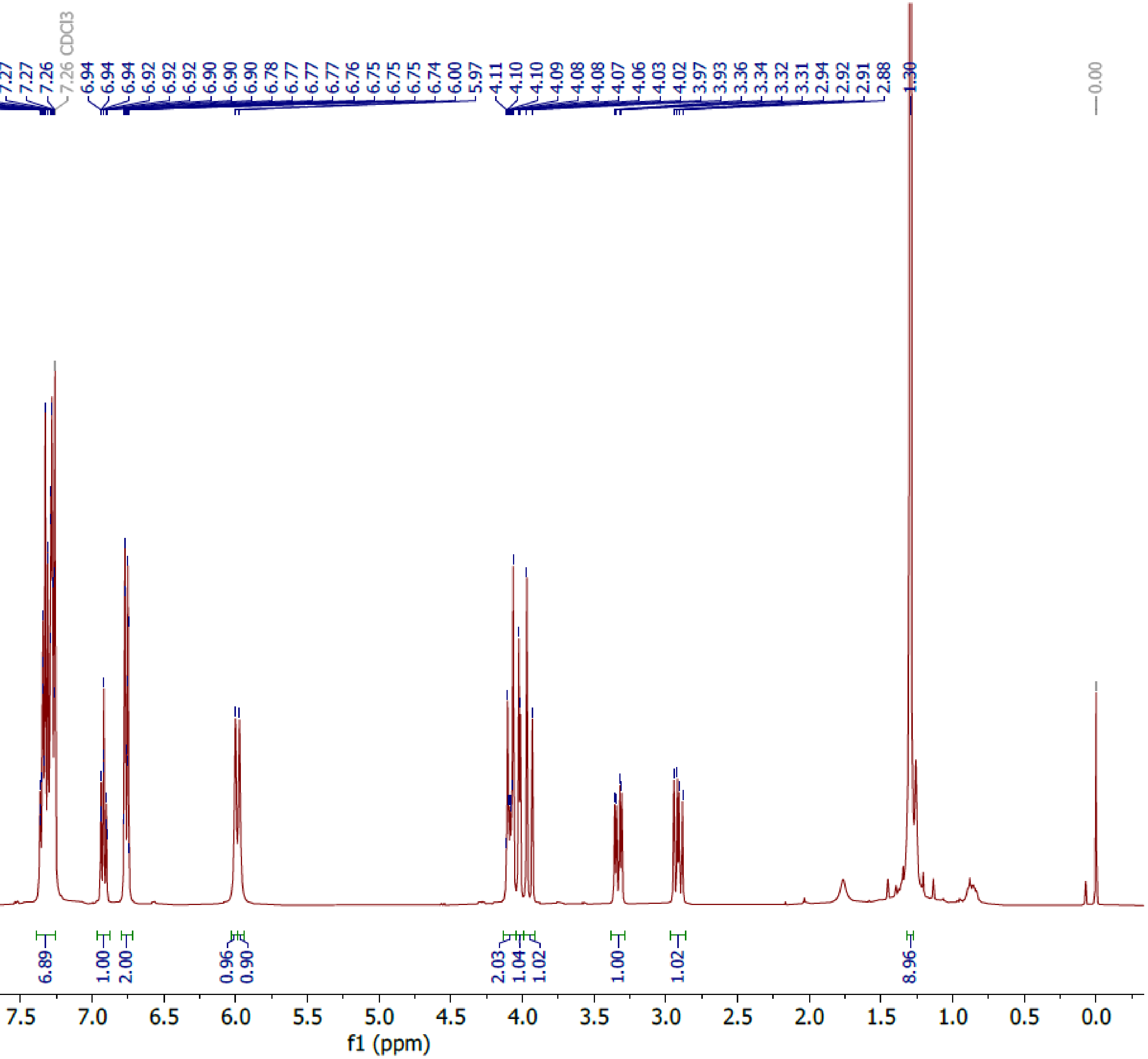

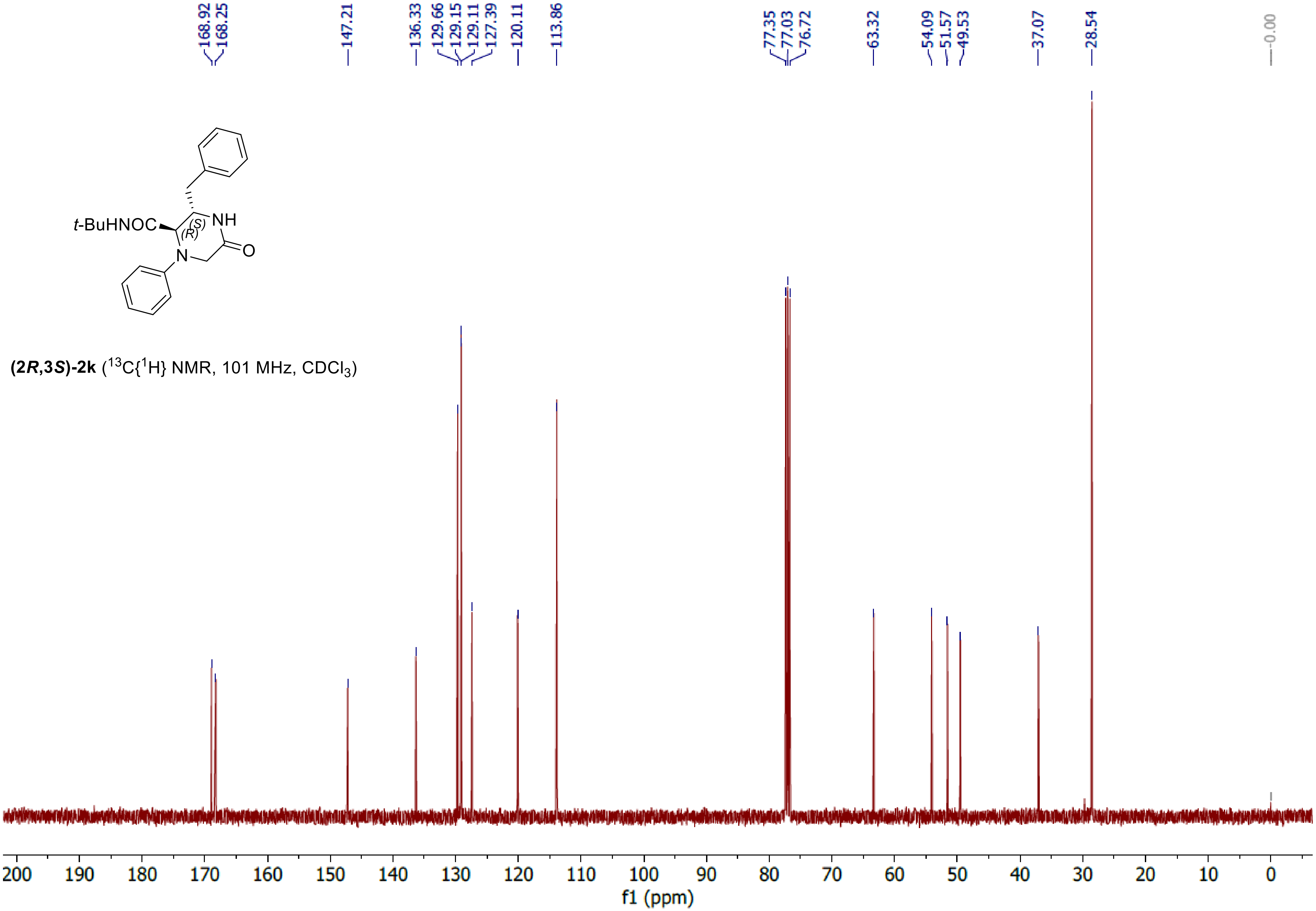
168.92
168.25
147.21
136.33
129.66
129.15
129.11
127.39
120.11
113.86
77.35
77.03
76.72
63.32
54.09
51.57
49.53
37.07
28.54
0.00
t-BuHNOC
(S)
(R)
NH
N
O
(2R,3S)-2k ($^{13}C\{^{1}H\}$ NMR, 101 MHz, $CDCl_3$)
200
190
180
170
160
150
140
130
120
110
100
90
80
70
60
50
40
30
20
10
0
f1 (ppm)

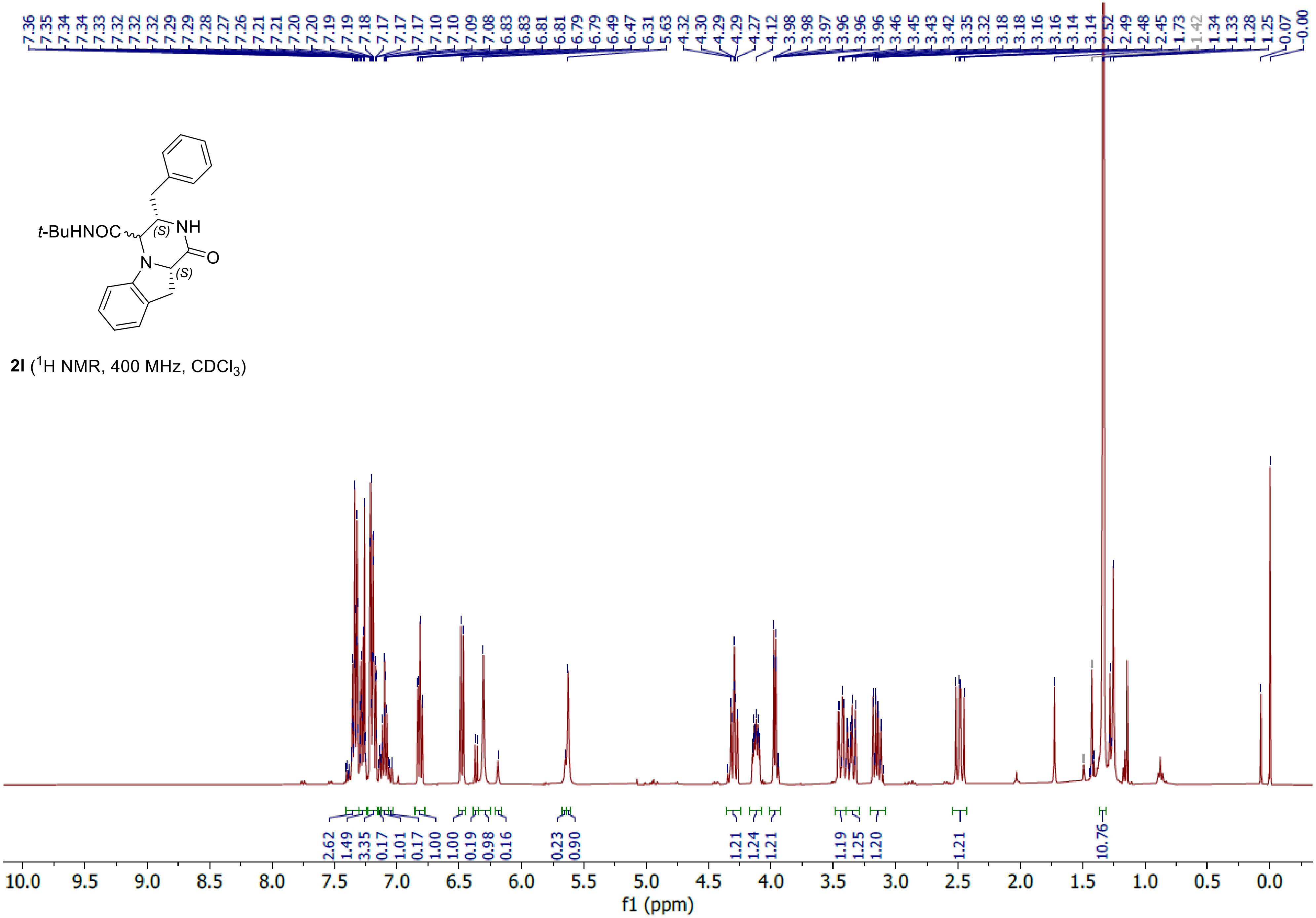

2l (^{1}H NMR, 400 MHz, $CDCl_3$)

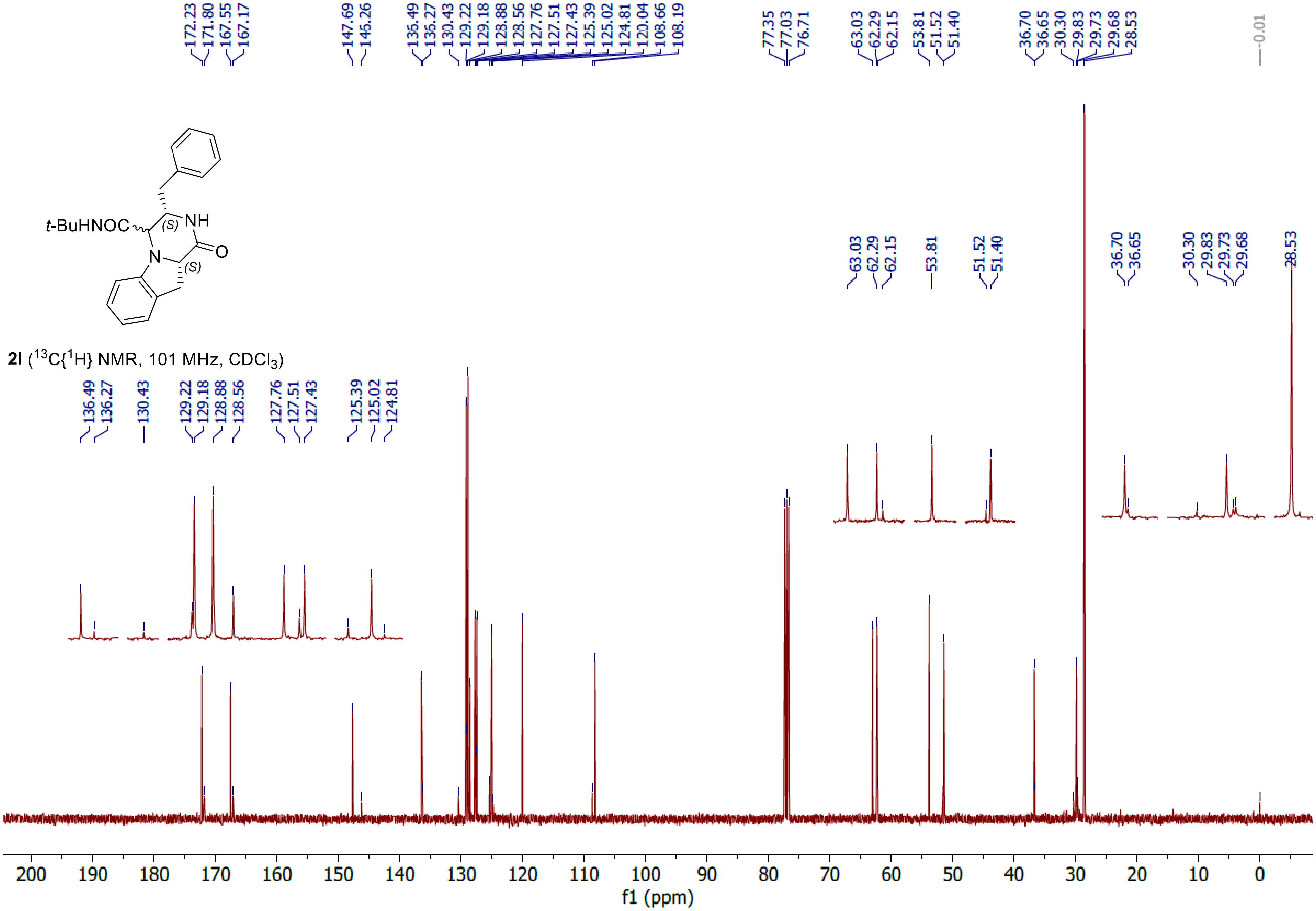

2l ($^{13}C\{^{1}H\}$ NMR, 101 MHz, $CDCl_3$)

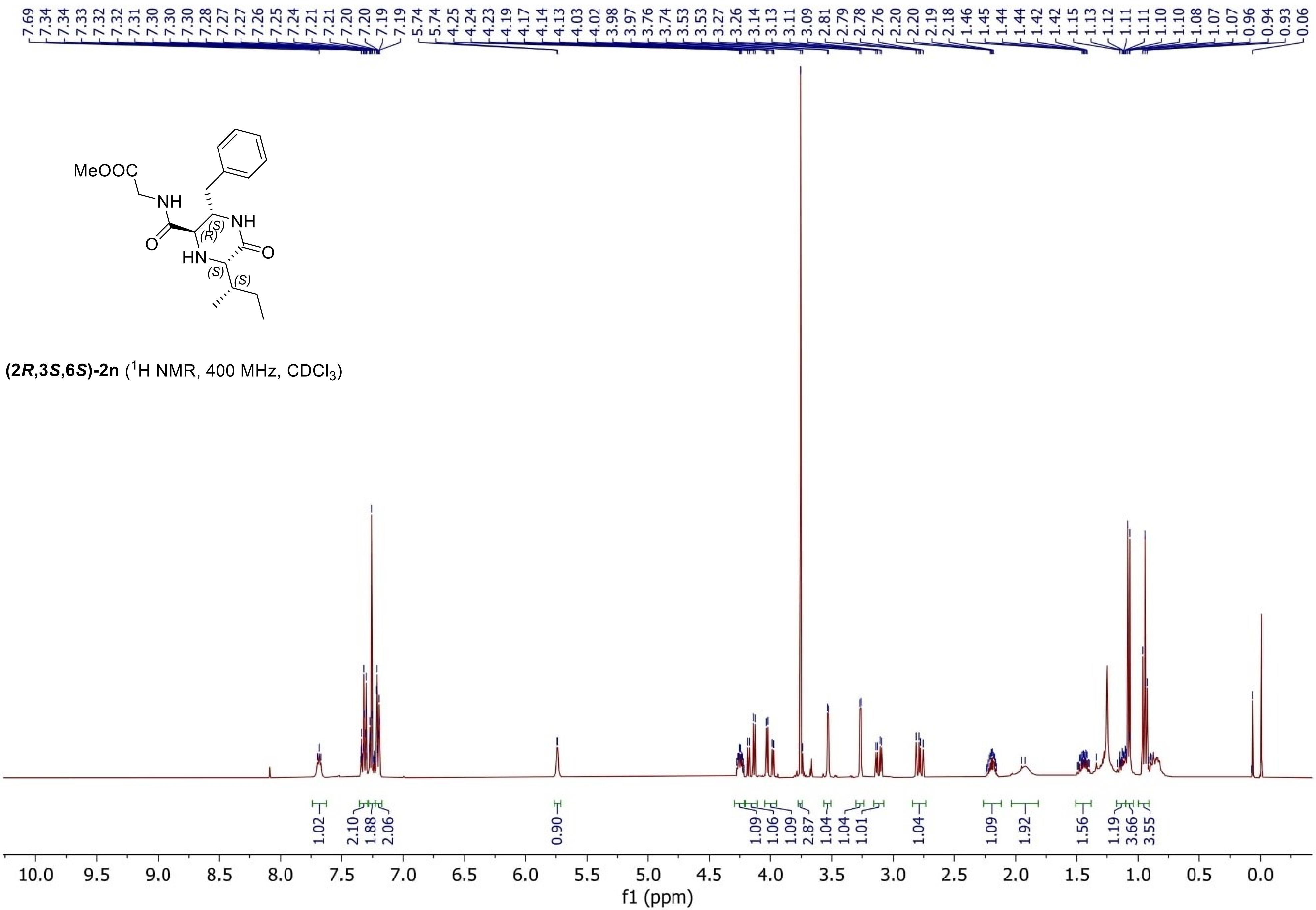

(2*R*,3*S*,6*S*)-2n (^{1}H NMR, 400 MHz, $CDCl_3$)

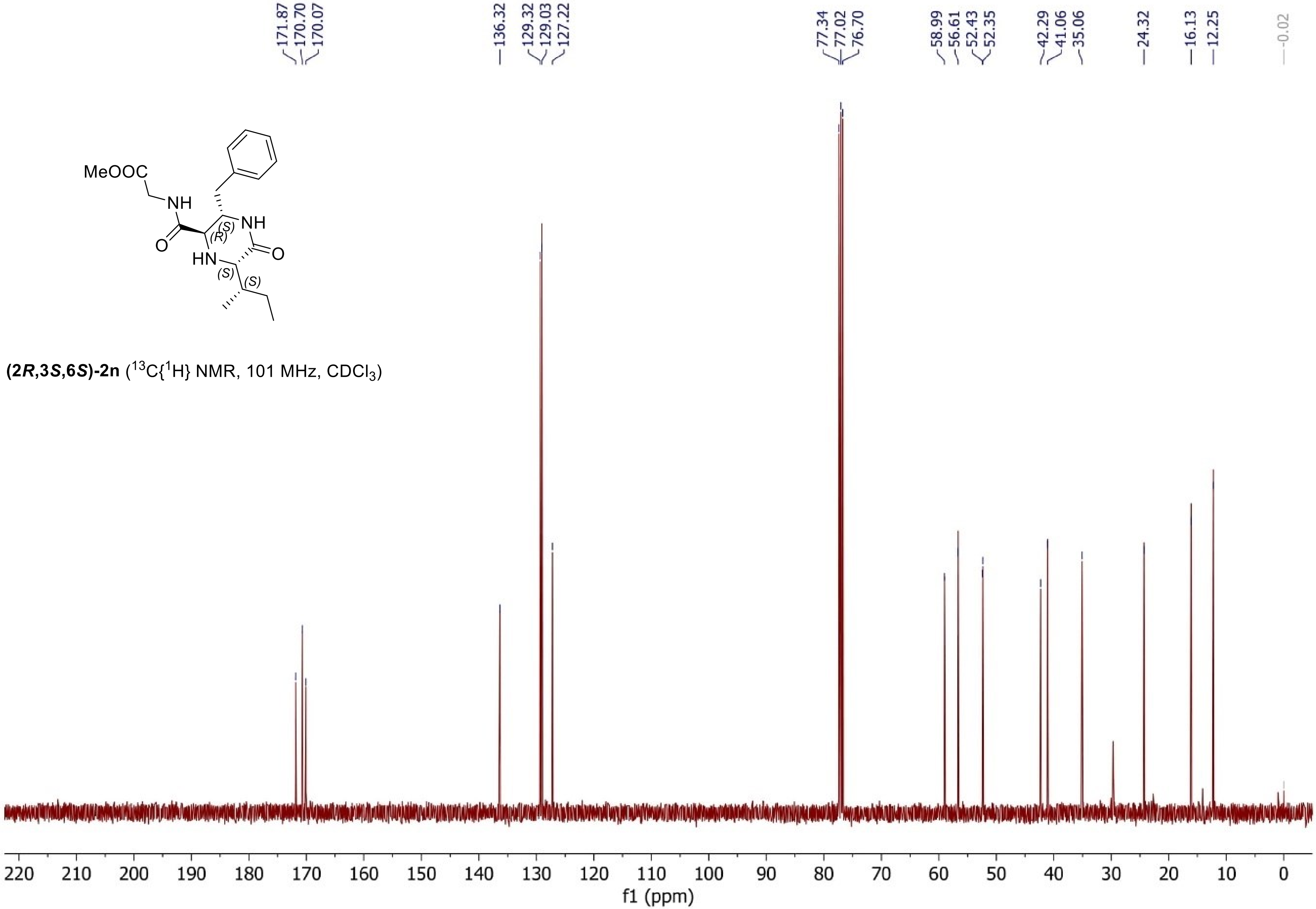

171.87
170.70
170.07
136.32
129.32
129.03
127.22
77.34
77.02
76.70
58.99
56.61
52.43
52.35
42.29
41.06
35.06
24.32
16.13
12.25
-0.02
MeOOC
NH
NH
O
HN
O
(S)
(R)
(S)
(S)
(2R,3S,6S)-2n (13C{1H} NMR, 101 MHz, CDCl3)
220 210 200 190 180 170 160 150 140 130 120 110 100 90 80 70 60 50 40 30 20 10 0
f1 (ppm)

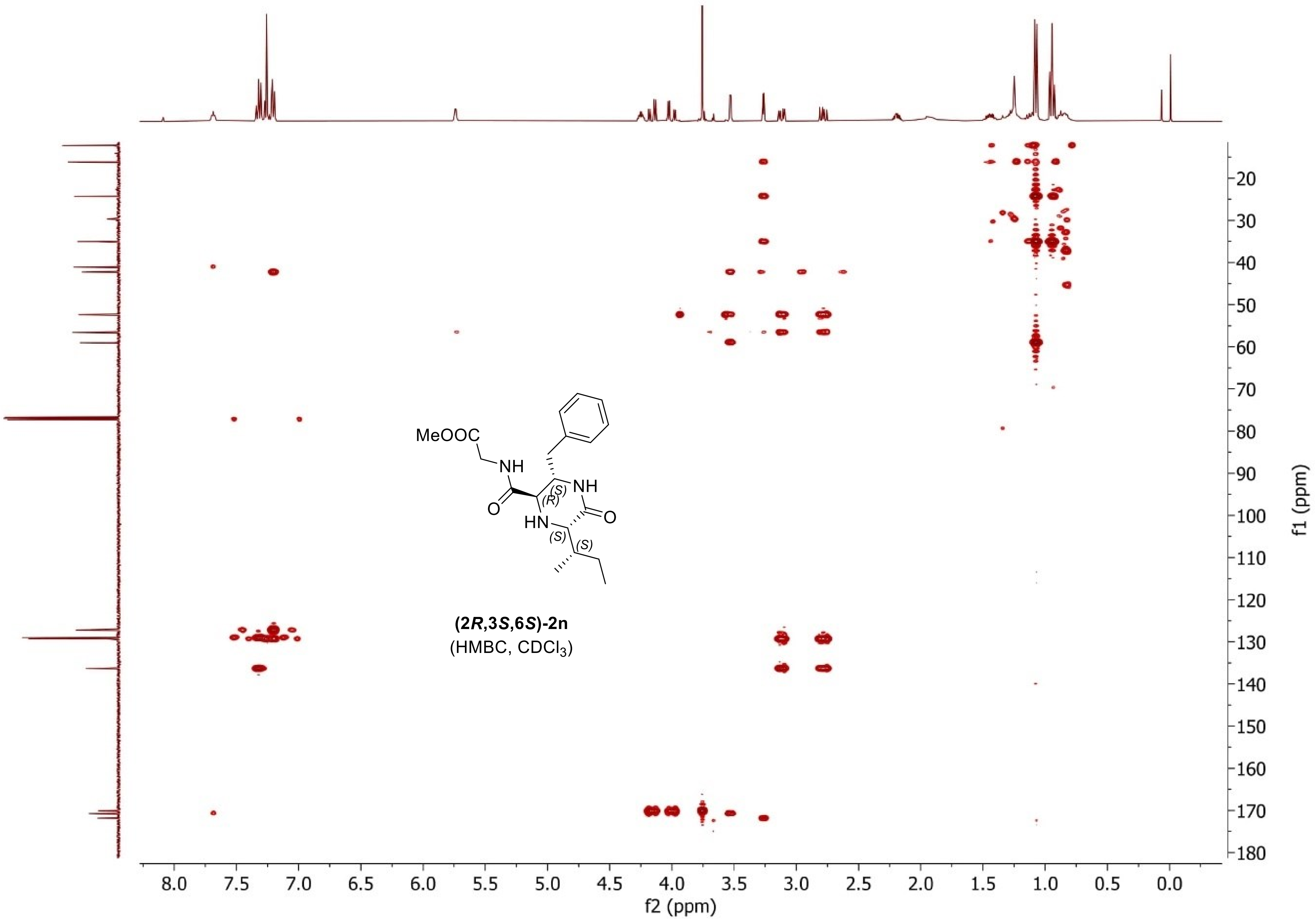

(2*R*,3*S*,6*S*)-2n
(HMBC, $CDCl_3$)

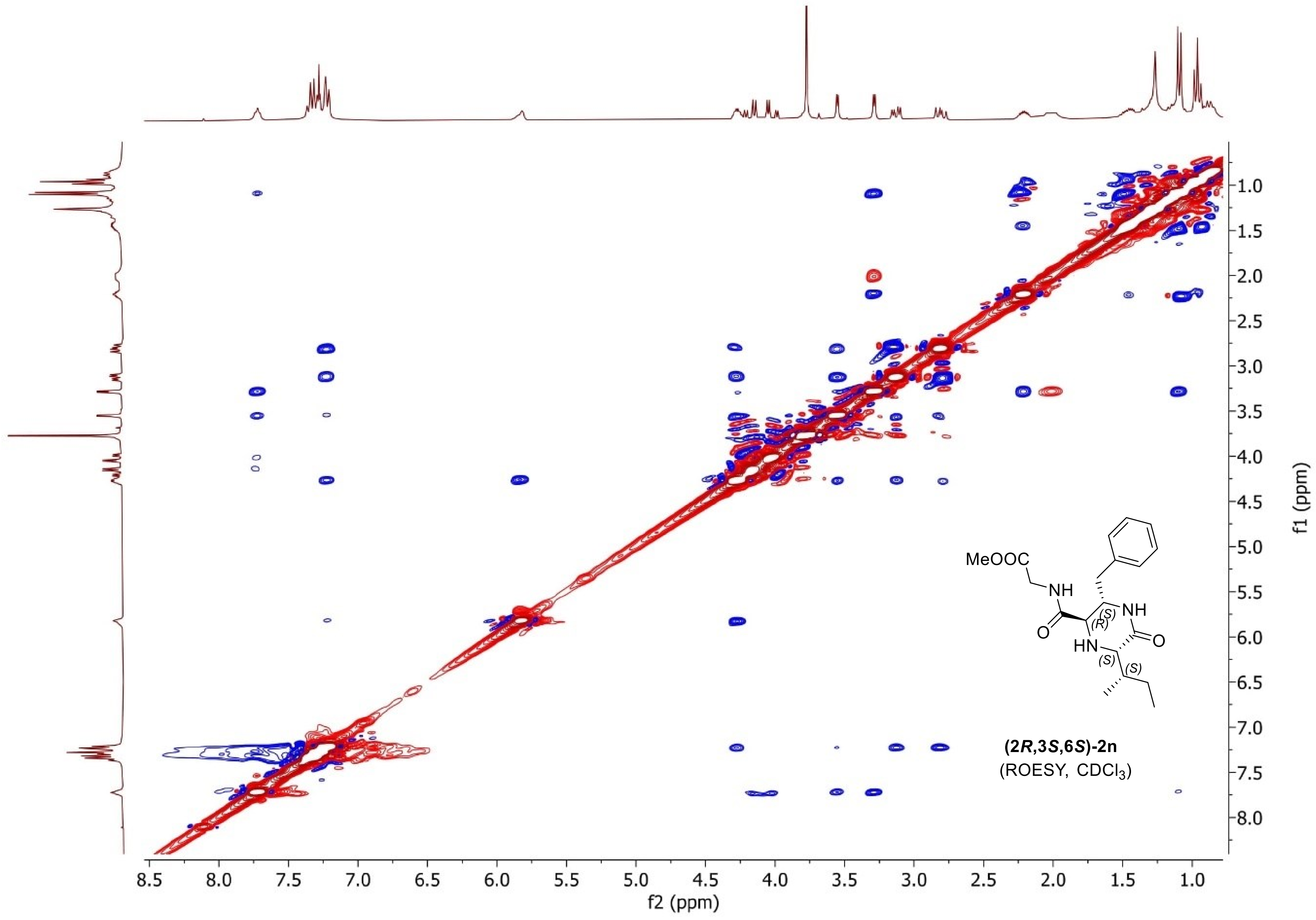

(2*R*,3*S*,6*S*)-2n
(ROESY, $CDCl_3$)

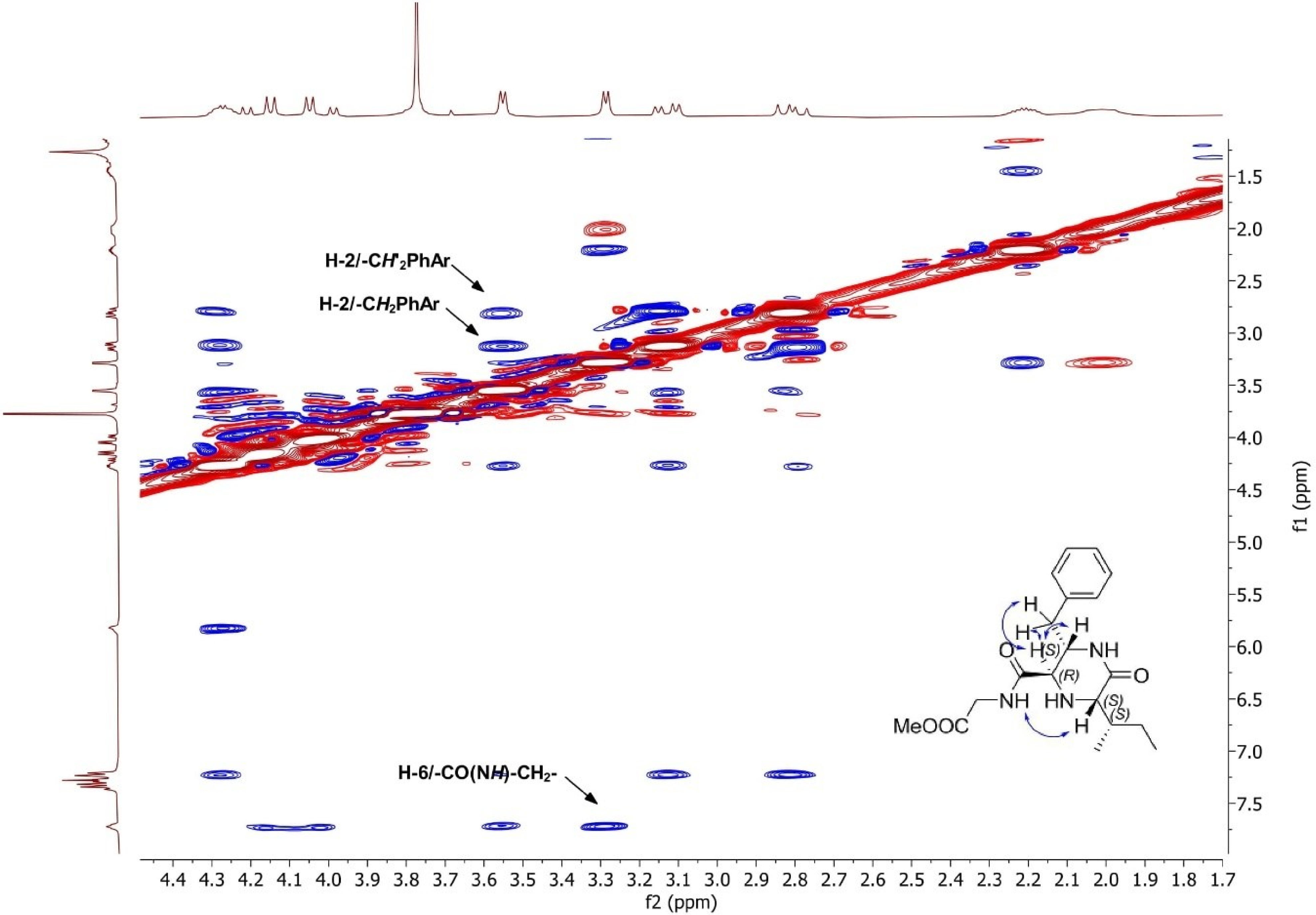

H-2/-CH'2PhAr
H-2/-CH2PhAr
H-6/-CO(NH)-CH2-
f2 (ppm)
f1 (ppm)
MeOOC
NH
HN
NH
O
H
(S)
(R)

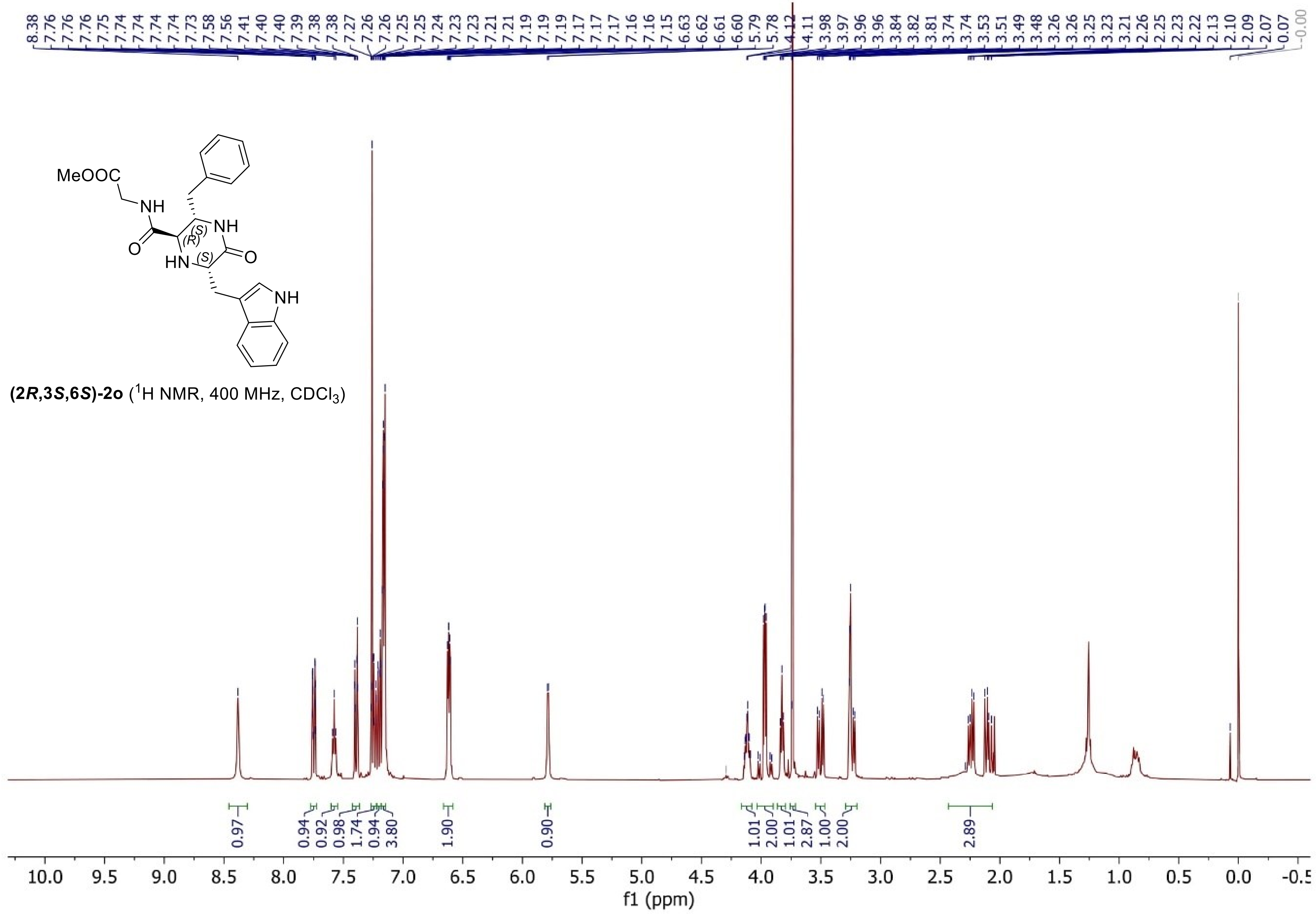

(2*R*,3*S*,6*S*)-2o (^{1}H NMR, 400 MHz, $CDCl_3$)

(2*R*,3*S*,6*S*)-2o

($^{13}C\{^{1}H\}$ NMR, 101 MHz, $CDCl_3$)

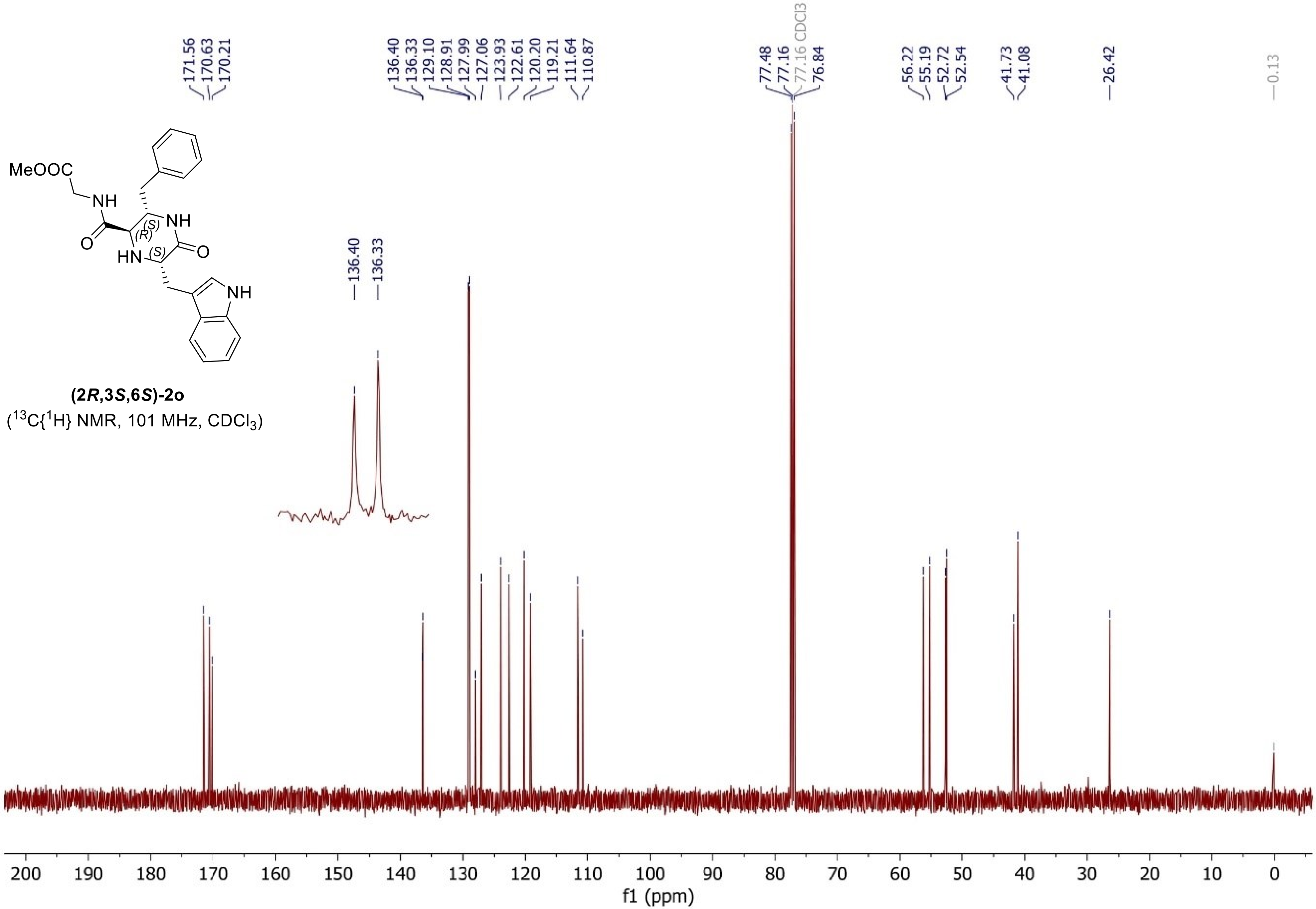

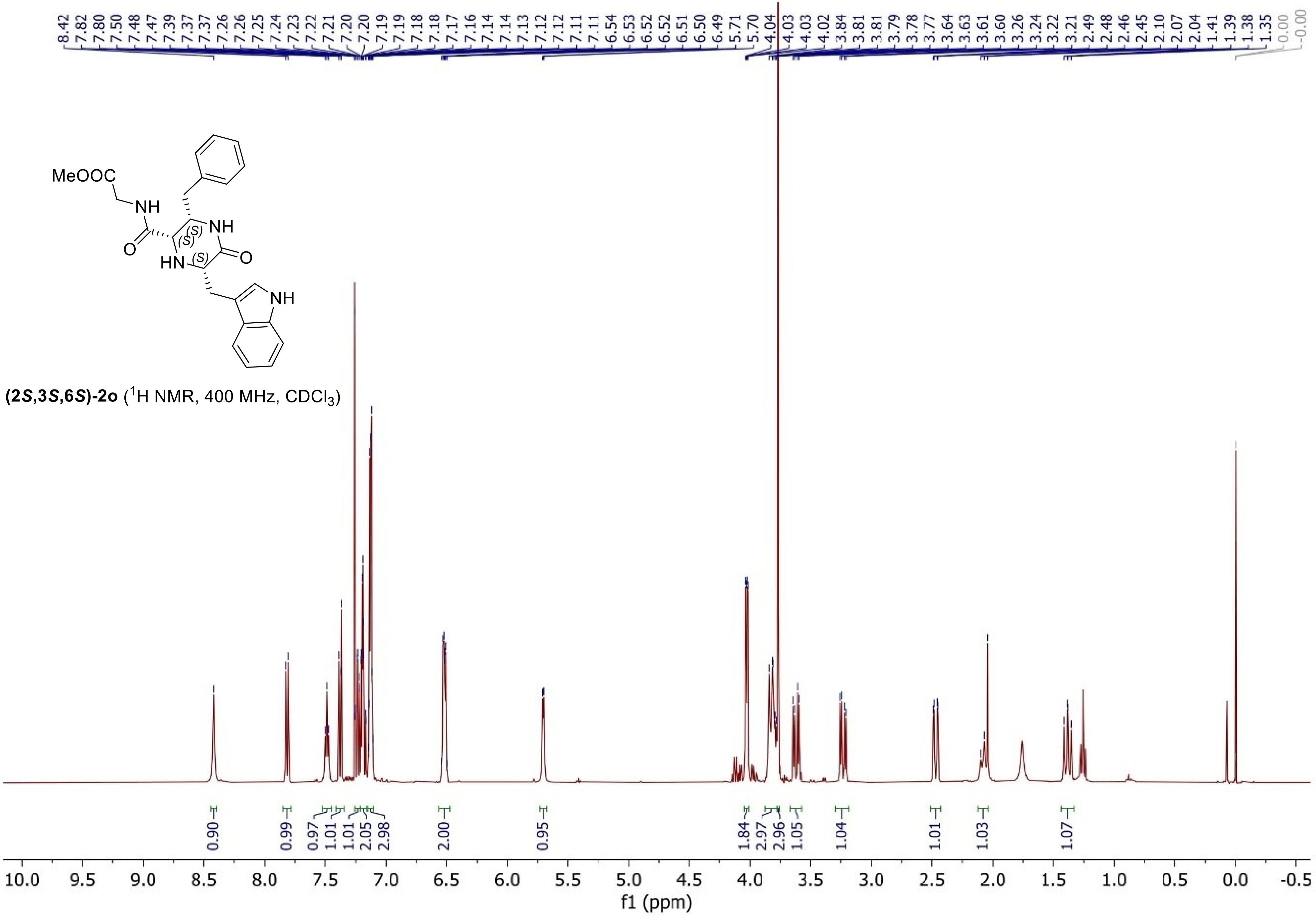

(2*S*,3*S*,6*S*)-2o (^1H NMR, 400 MHz, $CDCl_3$)

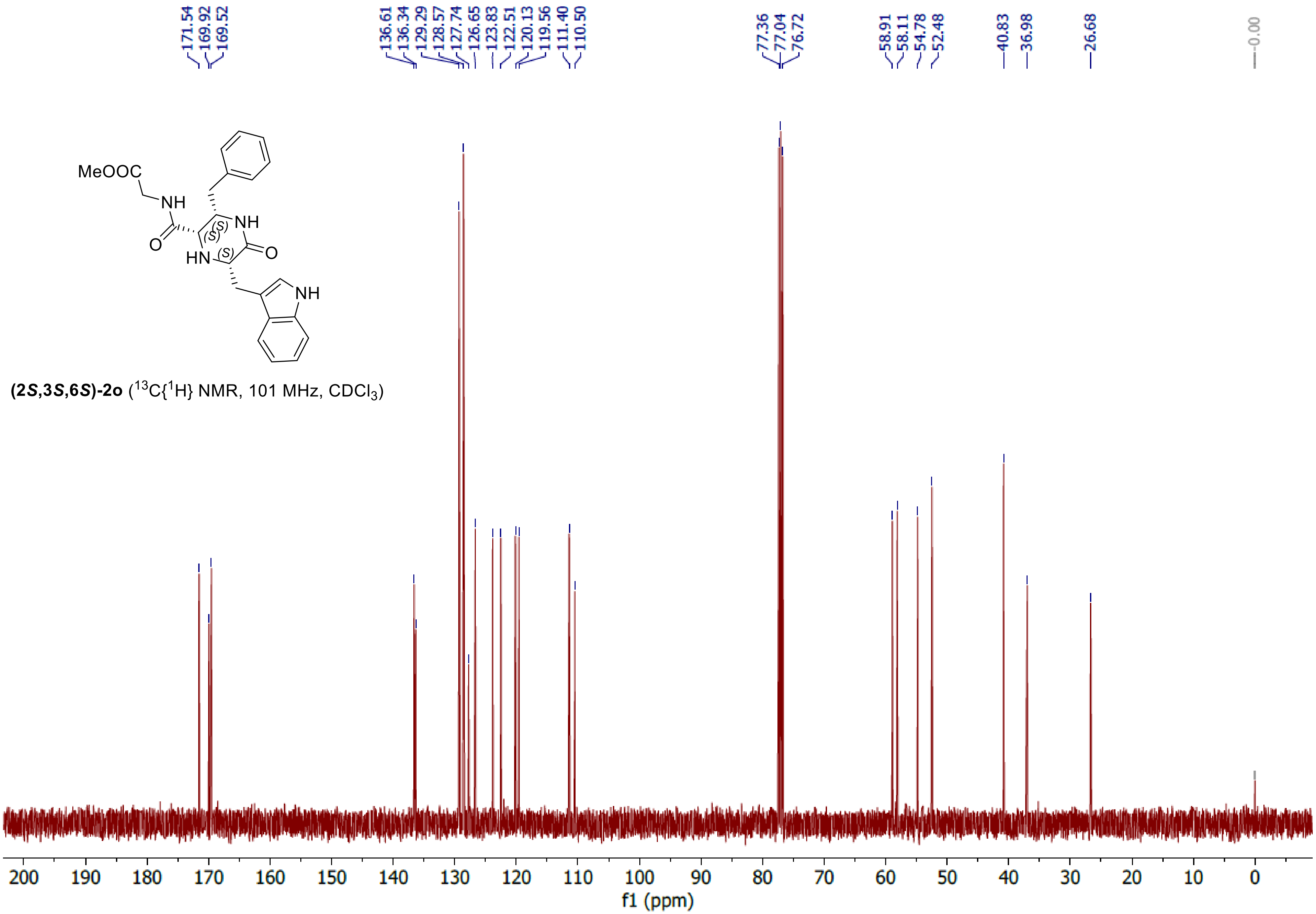

(2*S*,3*S*,6*S*)-2o ($^{13}C\{^1H\}$ NMR, 101 MHz, $CDCl_3$)

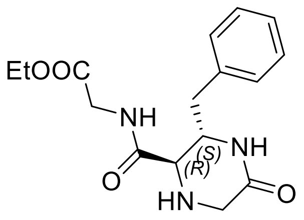

(2*R*,3*S*)-2p (^{1}H NMR, 400 MHz, $CDCl_3$)

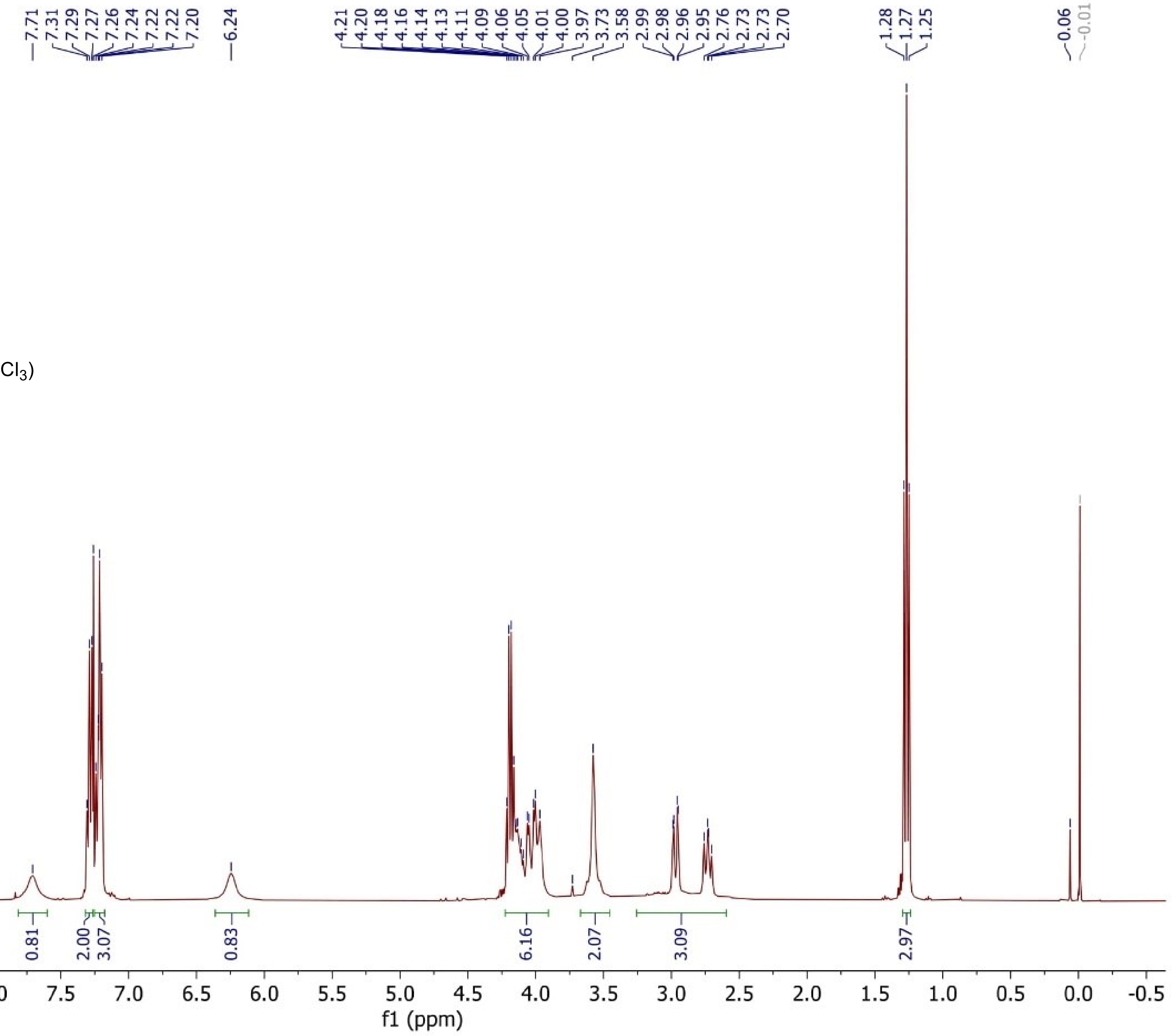

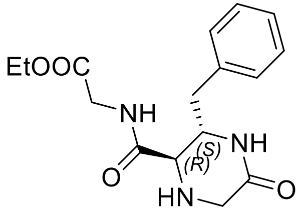

(2*R*,3*S*)-2p ($^{13}C\{^{1}H\}$ NMR, 101 MHz, $CDCl_3$)

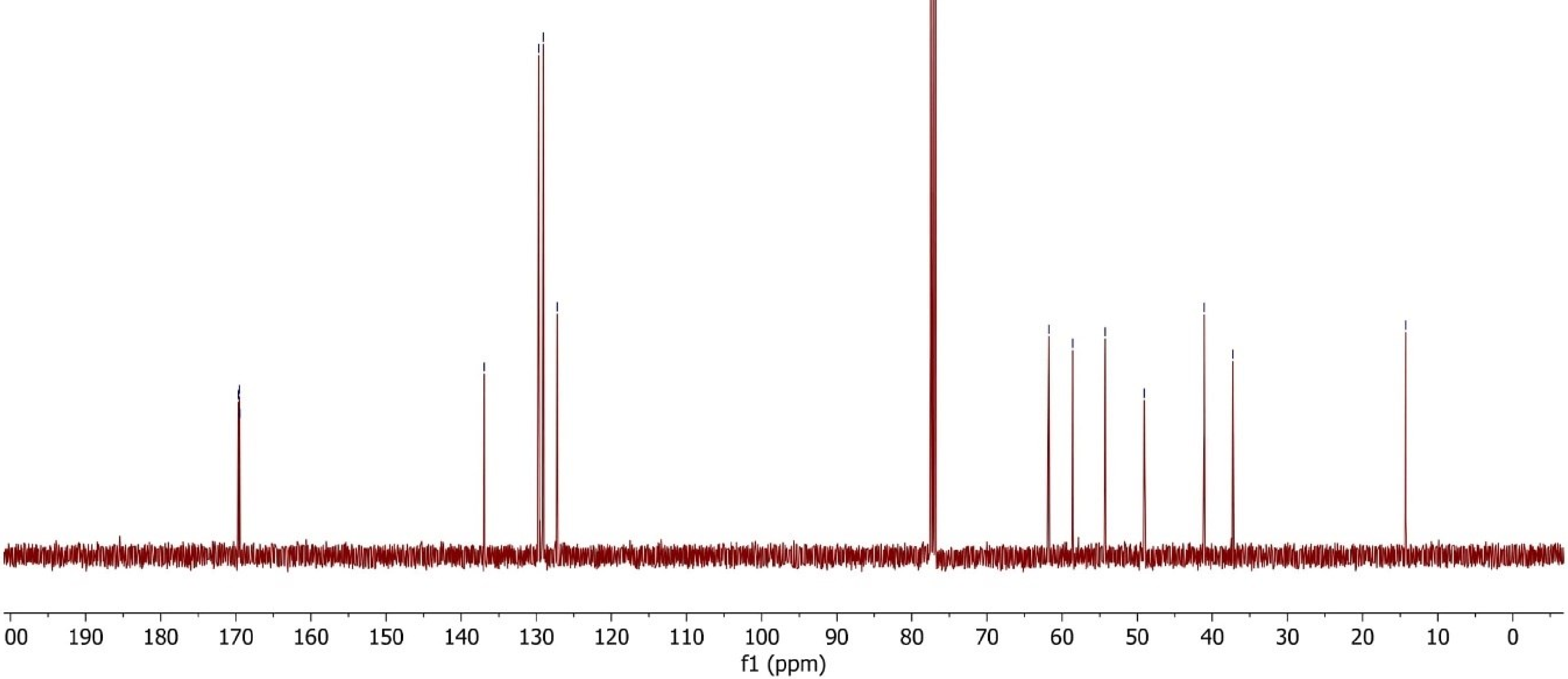

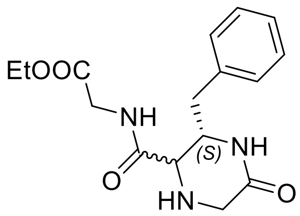

(2*R*,3*S*)-2p and **(2*S*,3*S*)-2p**
(^{1}H NMR, 400 MHz, $CDCl_3$)

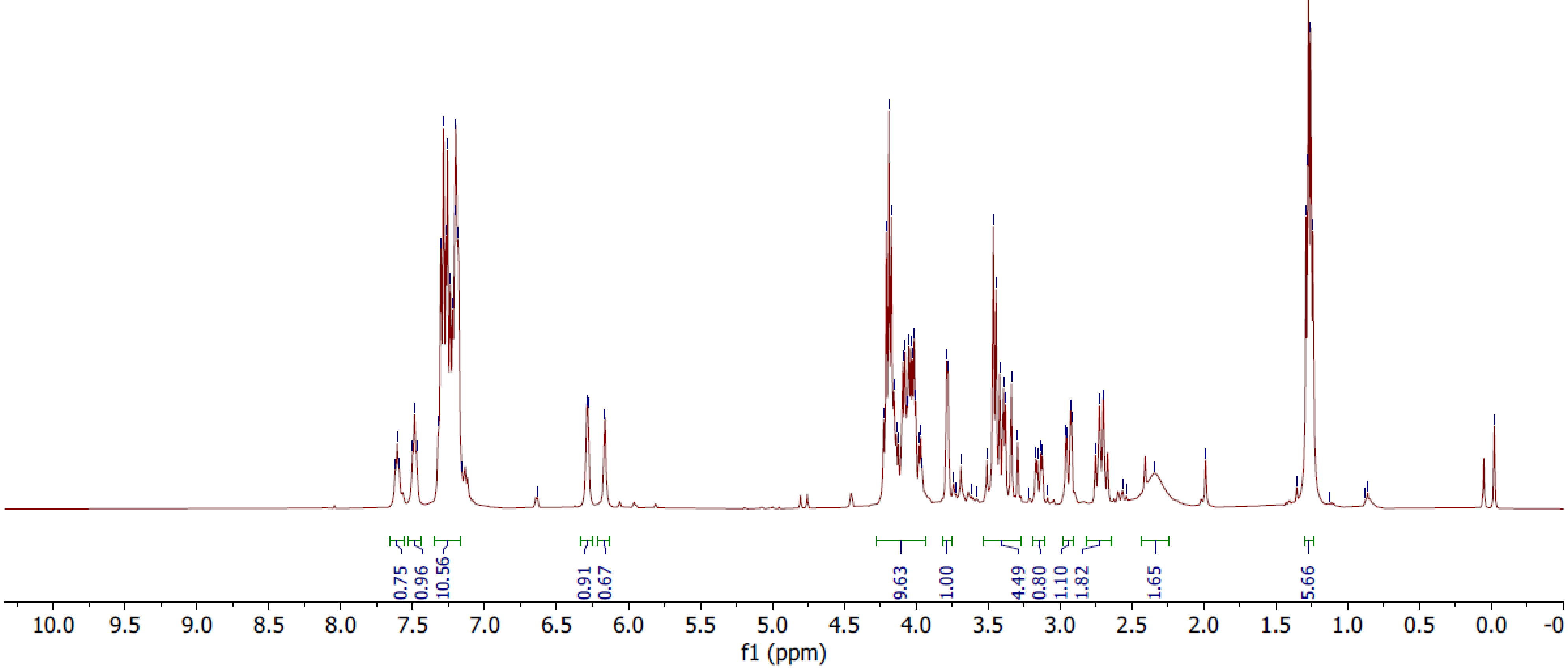

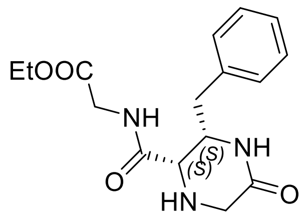

(2*R*,3*S*)-2p and **(2*S*,3*S*)-2p**
($^{13}C\{^1H\}$ NMR, 101 MHz, $CDCl_3$)

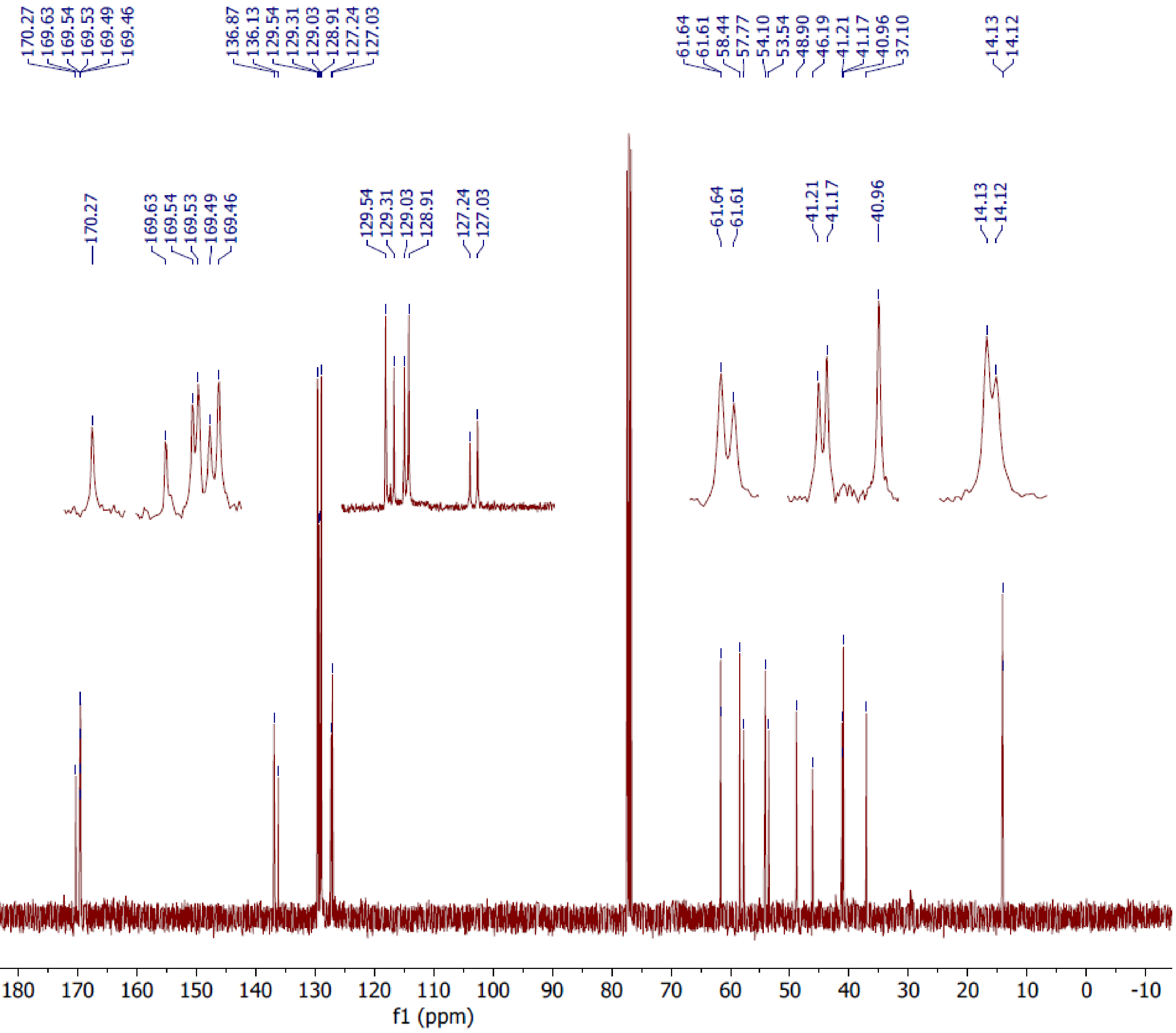

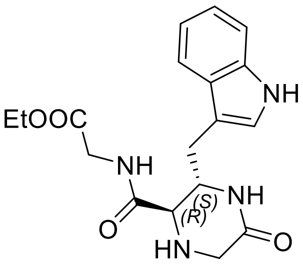

(***2R*,3*S*)-2t** (^{1}H NMR, 500 MHz, CD_3OD)

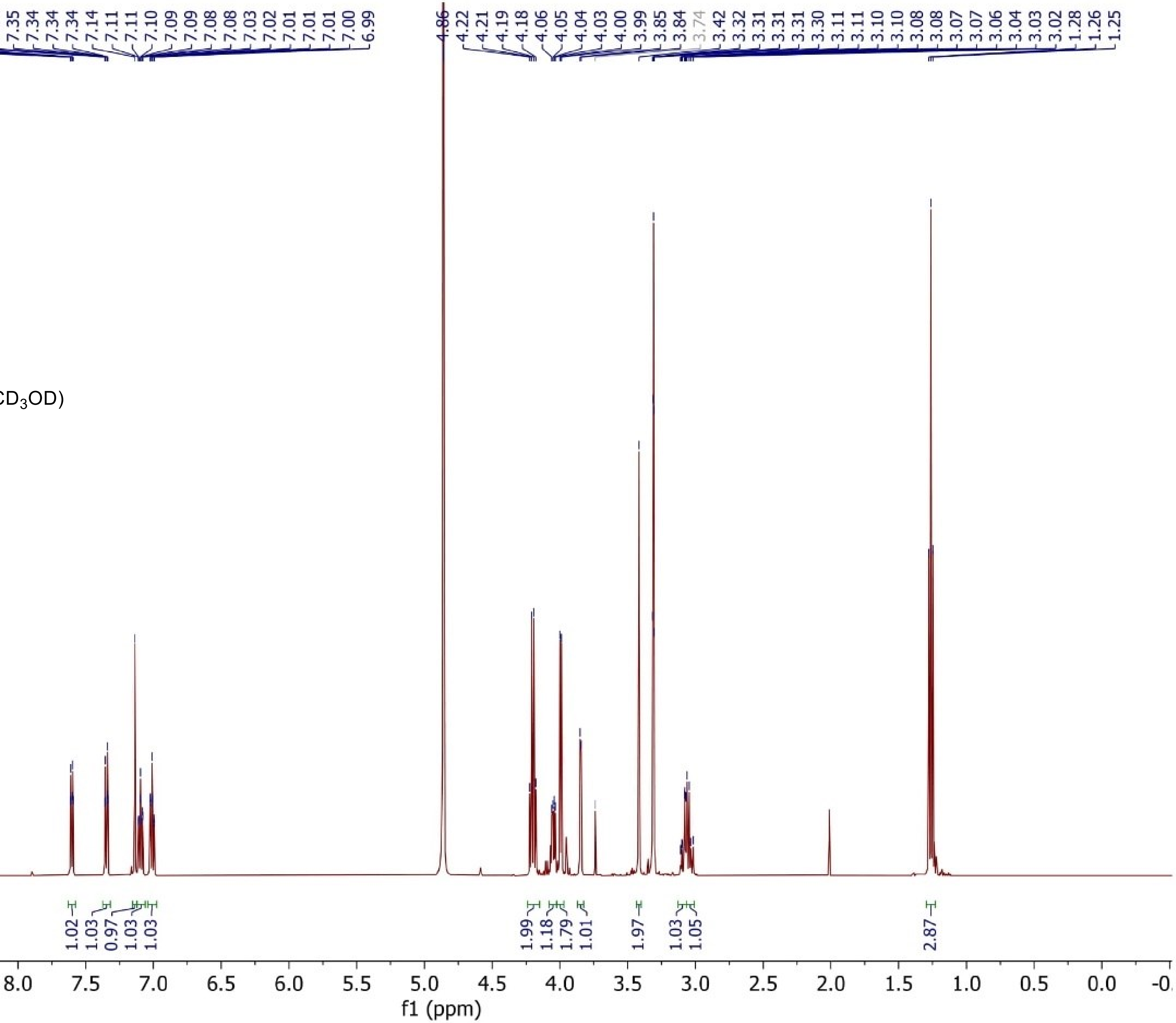

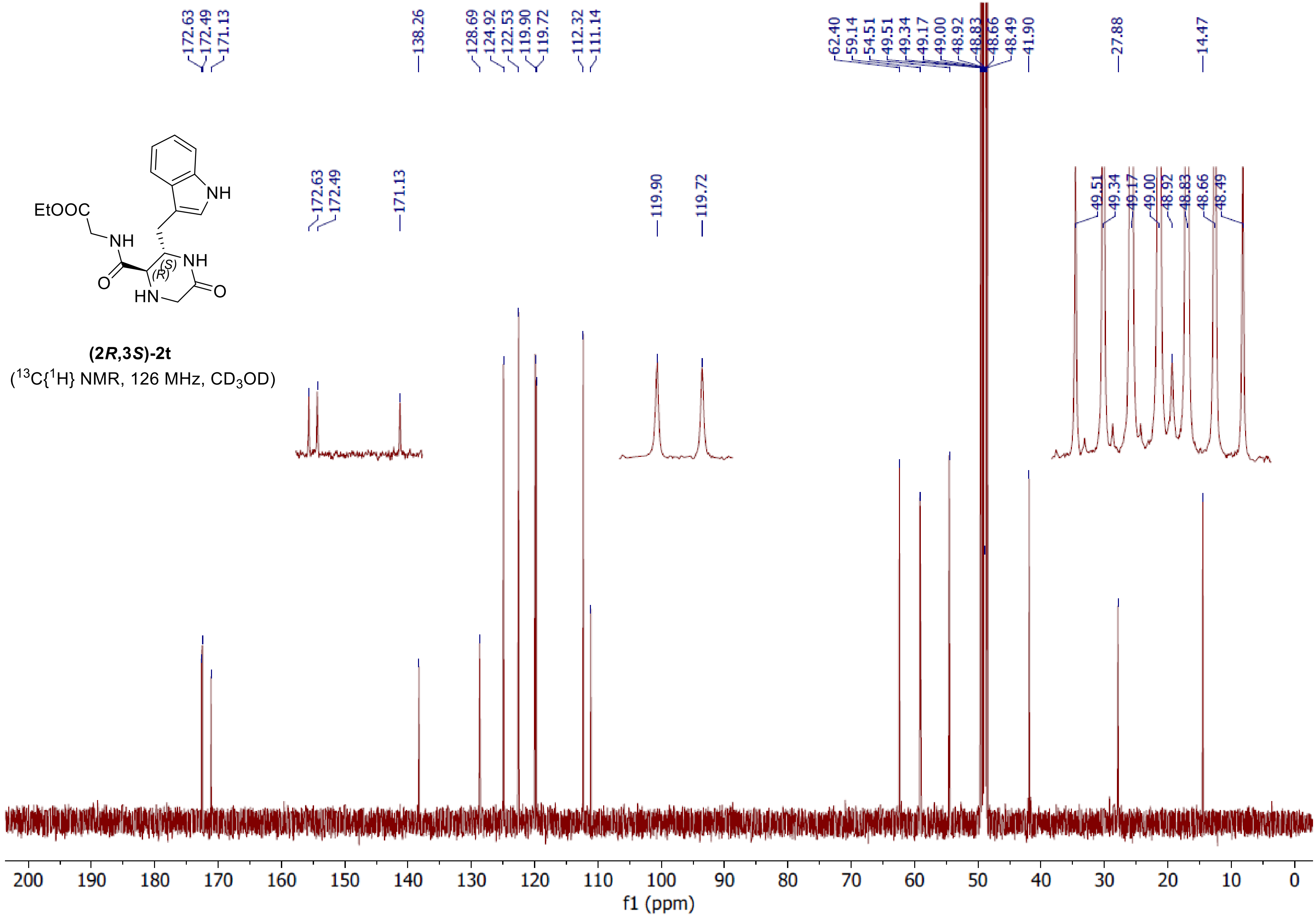

(2*R*,3*S*)-2t

($^{13}C\{^{1}H\}$ NMR, 126 MHz, CD_3OD)

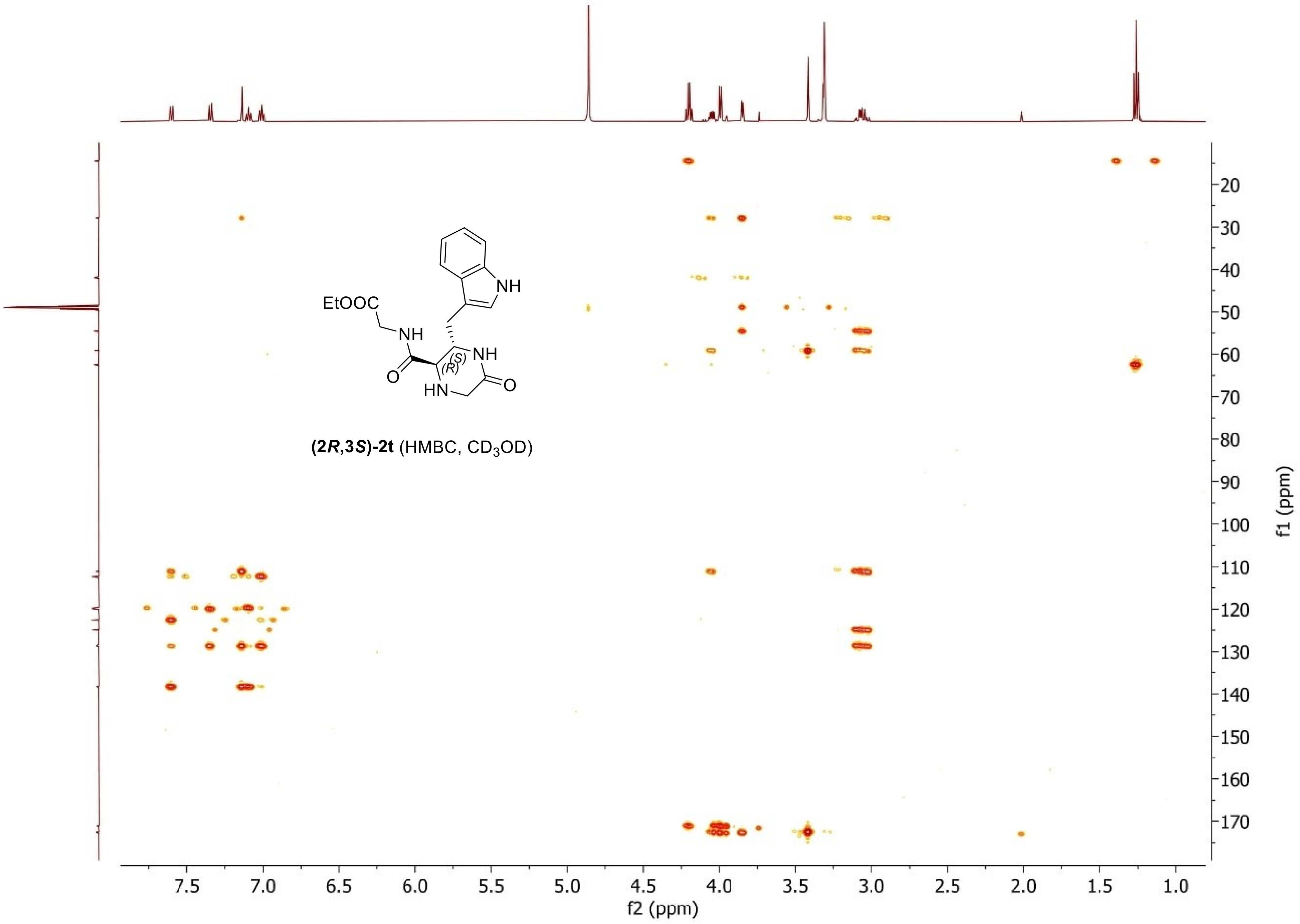
EtOOC
NH
NH
NH
HN
O
O
(S)
(R)
(2R,3S)-2t (HMBC, CD3OD)
f1 (ppm)
f2 (ppm)
20
30
40
50
60
70
80
90
100
110
120
130
140
150
160
170
7.5
7.0
6.5
6.0
5.5
5.0
4.5
4.0
3.5
3.0
2.5
2.0
1.5
1.0

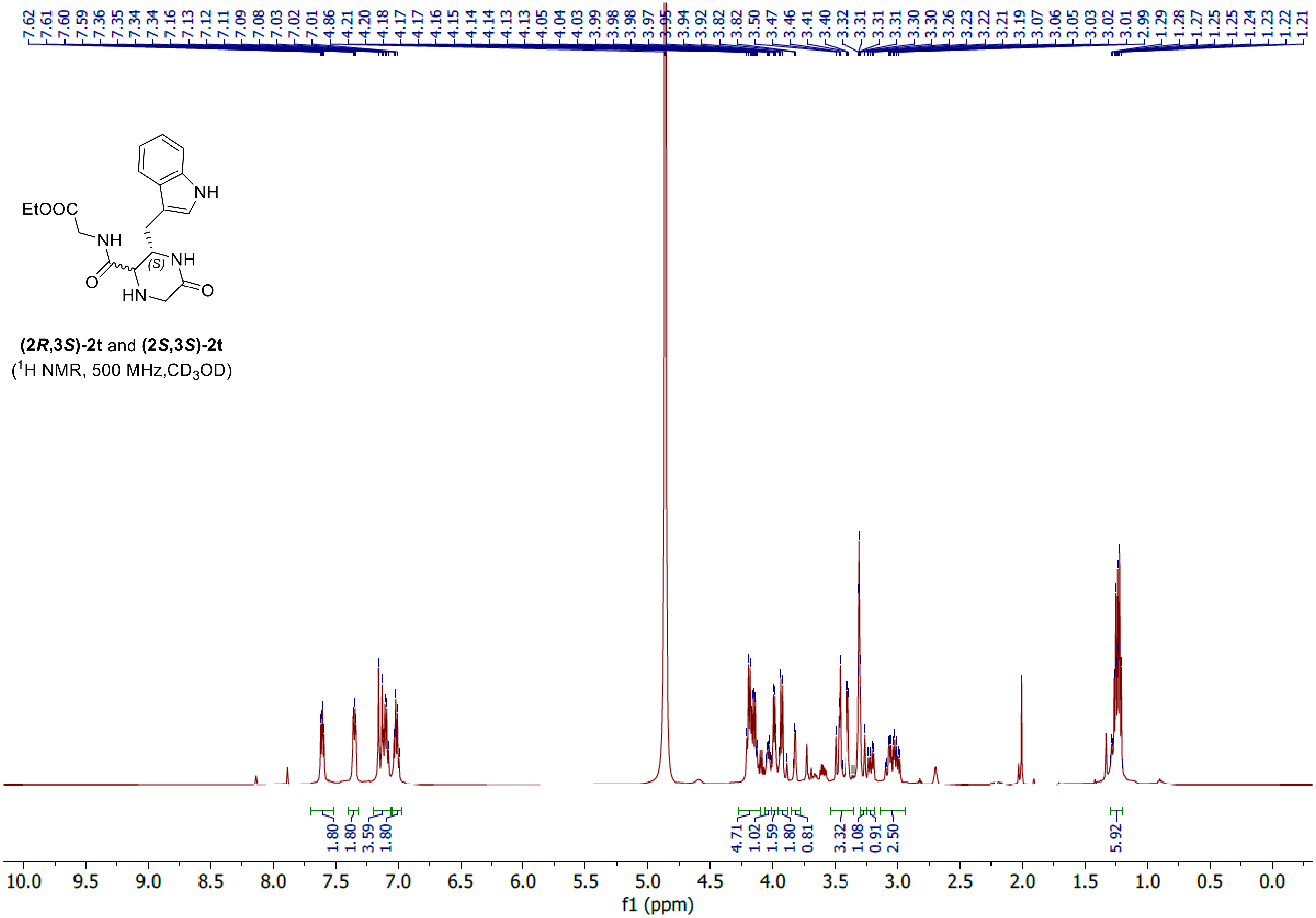

(2*R*,3*S*)-2t and **(2*S*,3*S*)-2t**
(^{1}H NMR, 500 MHz, CD_3OD)

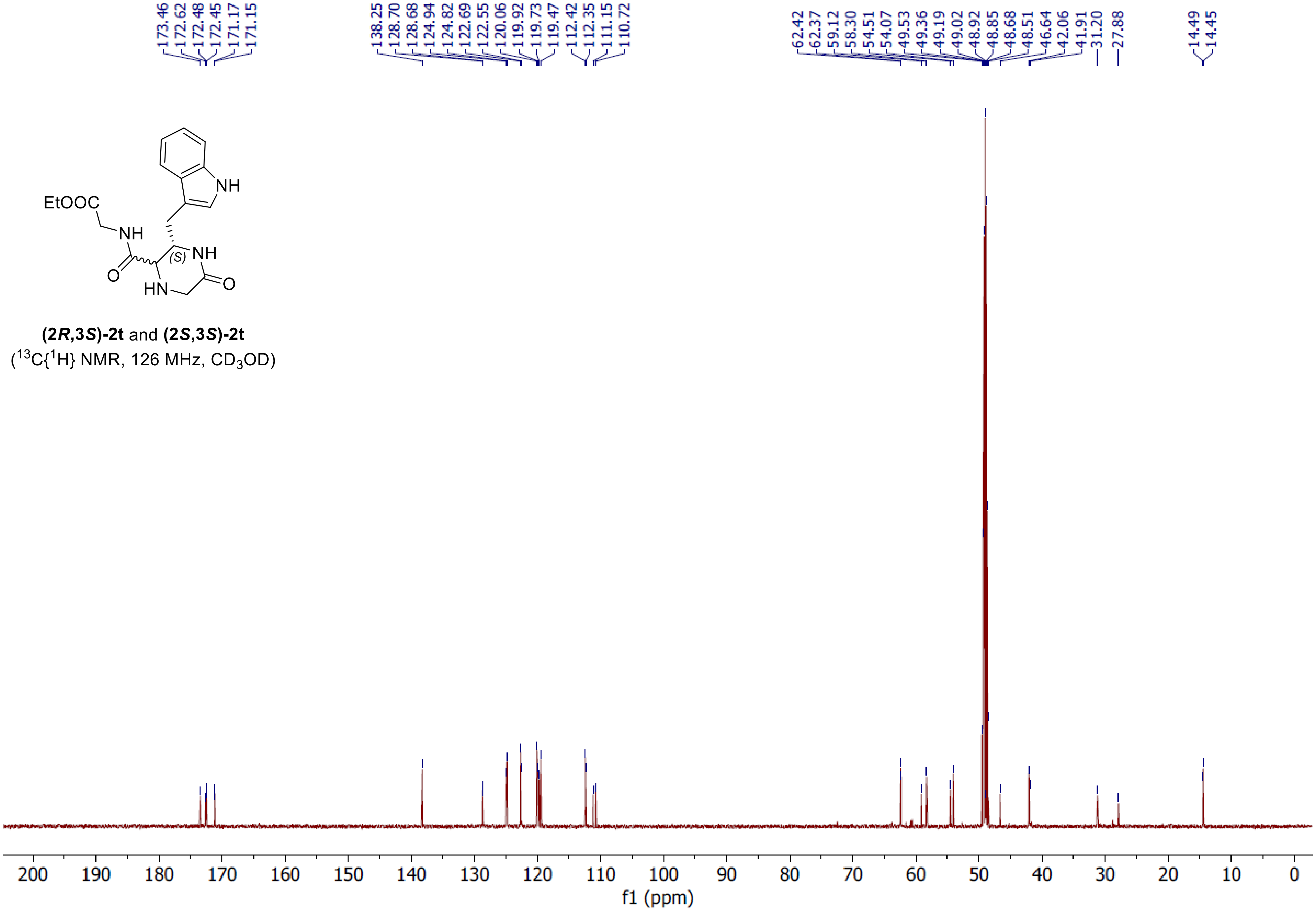

(2*R*,3*S*)-2t and **(2*S*,3*S*)-2t**
($^{13}C\{^1H\}$ NMR, 126 MHz, CD_3OD)

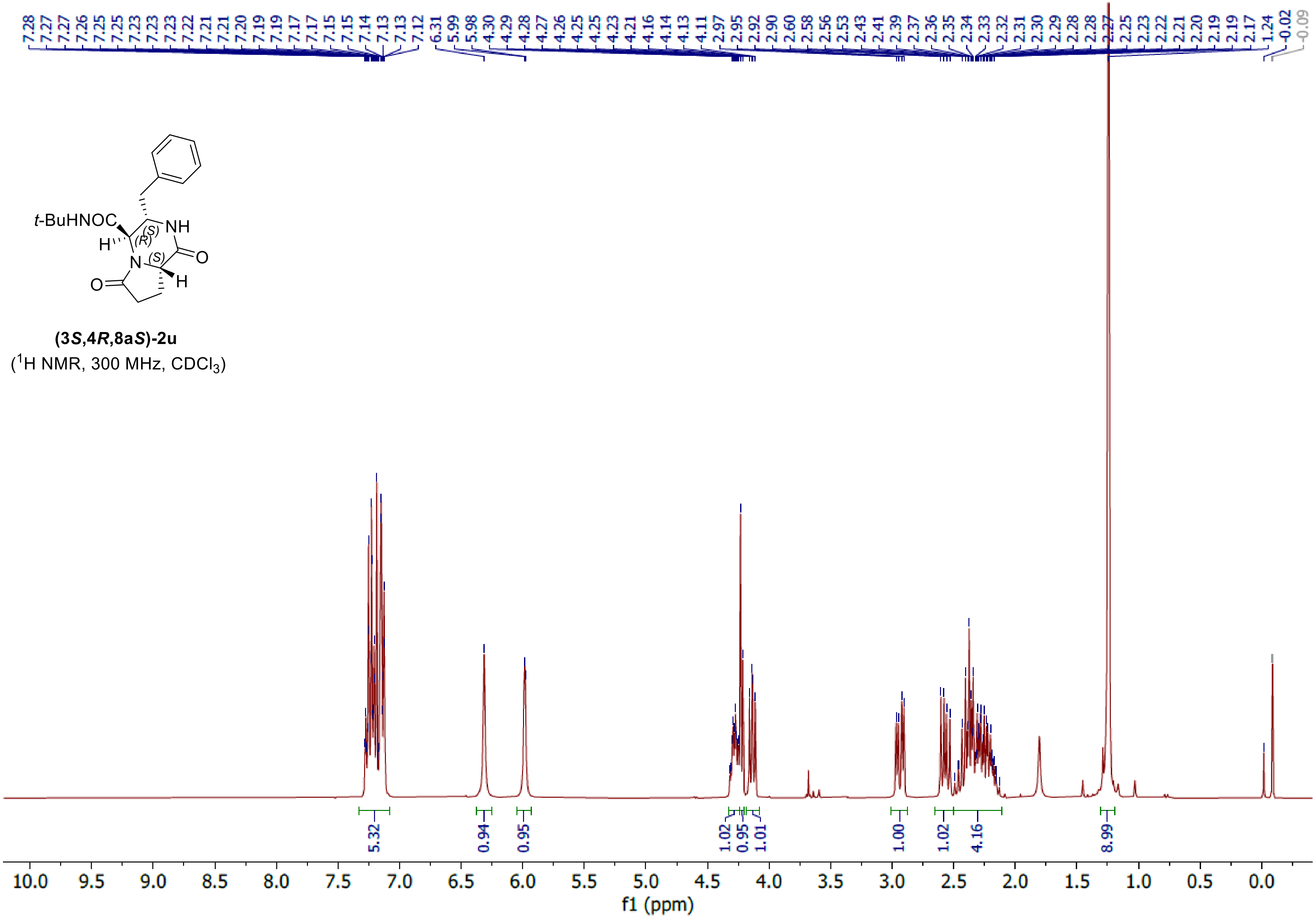
t-BuHNOC
NH
H
N
O
O
H
(S)
(R)
(S)
(3S,4R,8aS)-2u
(1H NMR, 300 MHz, CDCl3)
f1 (ppm)

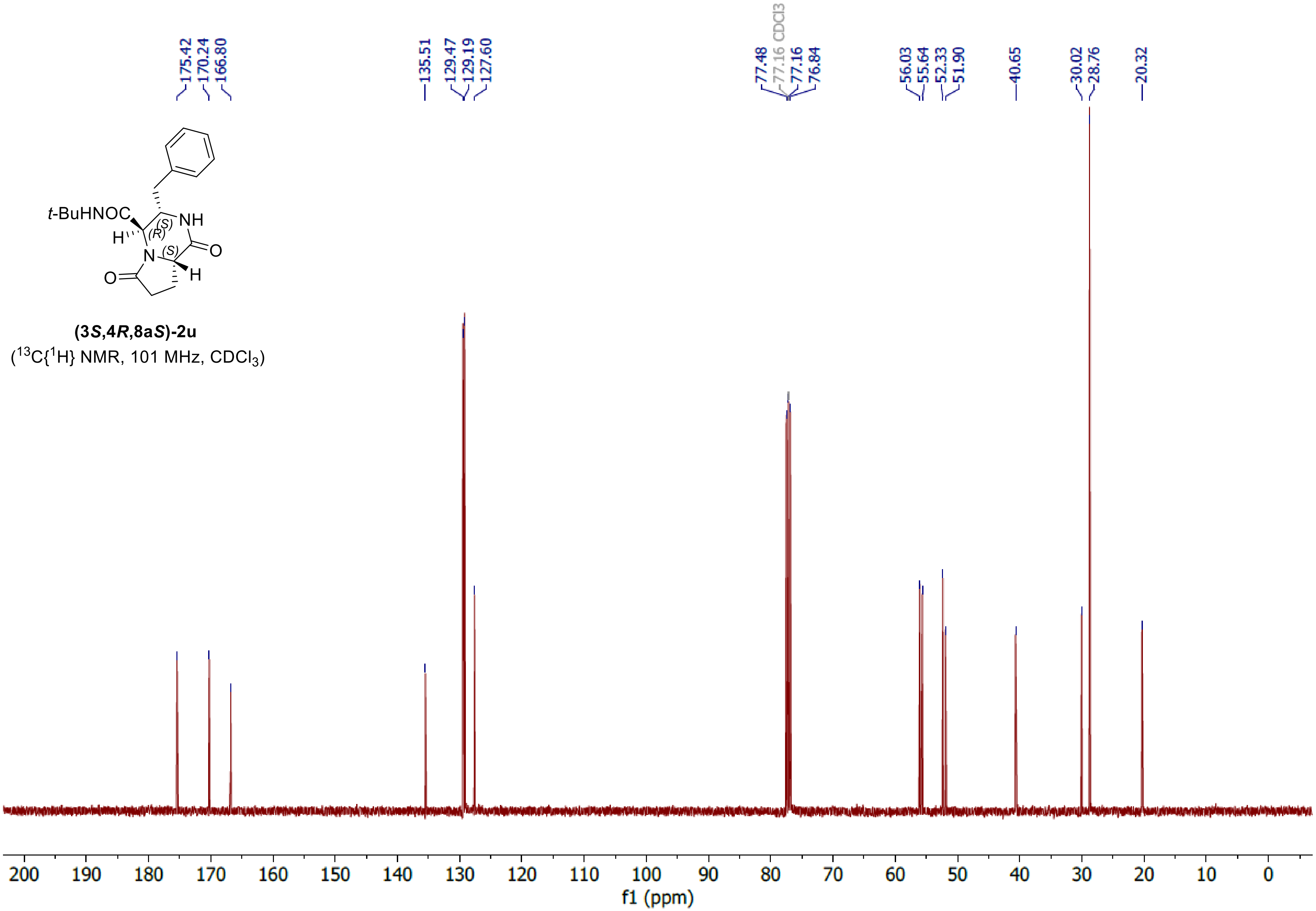

(3*S*,4*R*,8a*S*)-2u

($^{13}C\{^{1}H\}$ NMR, 101 MHz, $CDCl_3$)

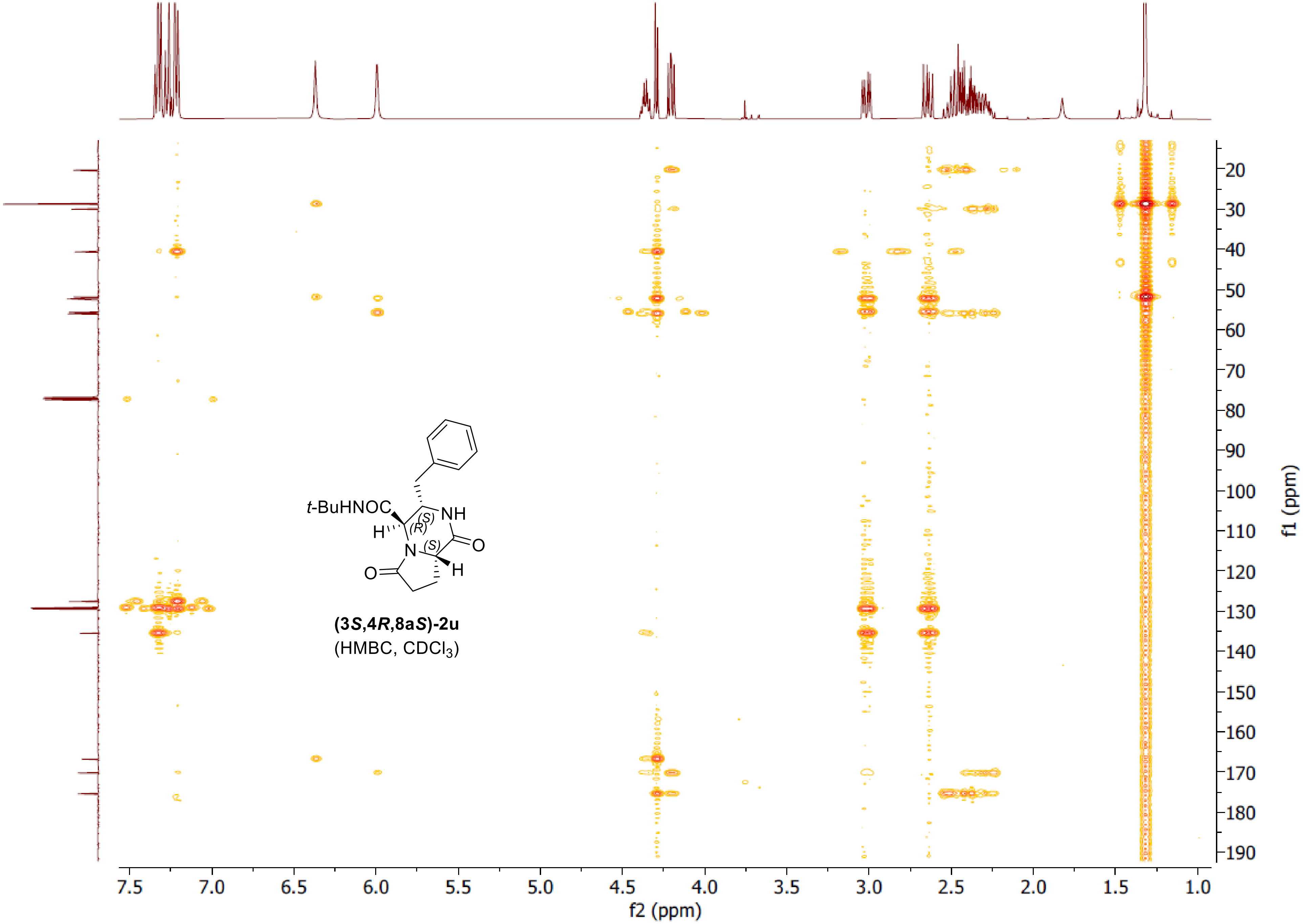
t-BuHNOC
NH
O
H
N
O
H
(S)
(R)
(S)
(3S,4R,8aS)-2u
(HMBC, CDCl3)
f2 (ppm)
f1 (ppm)

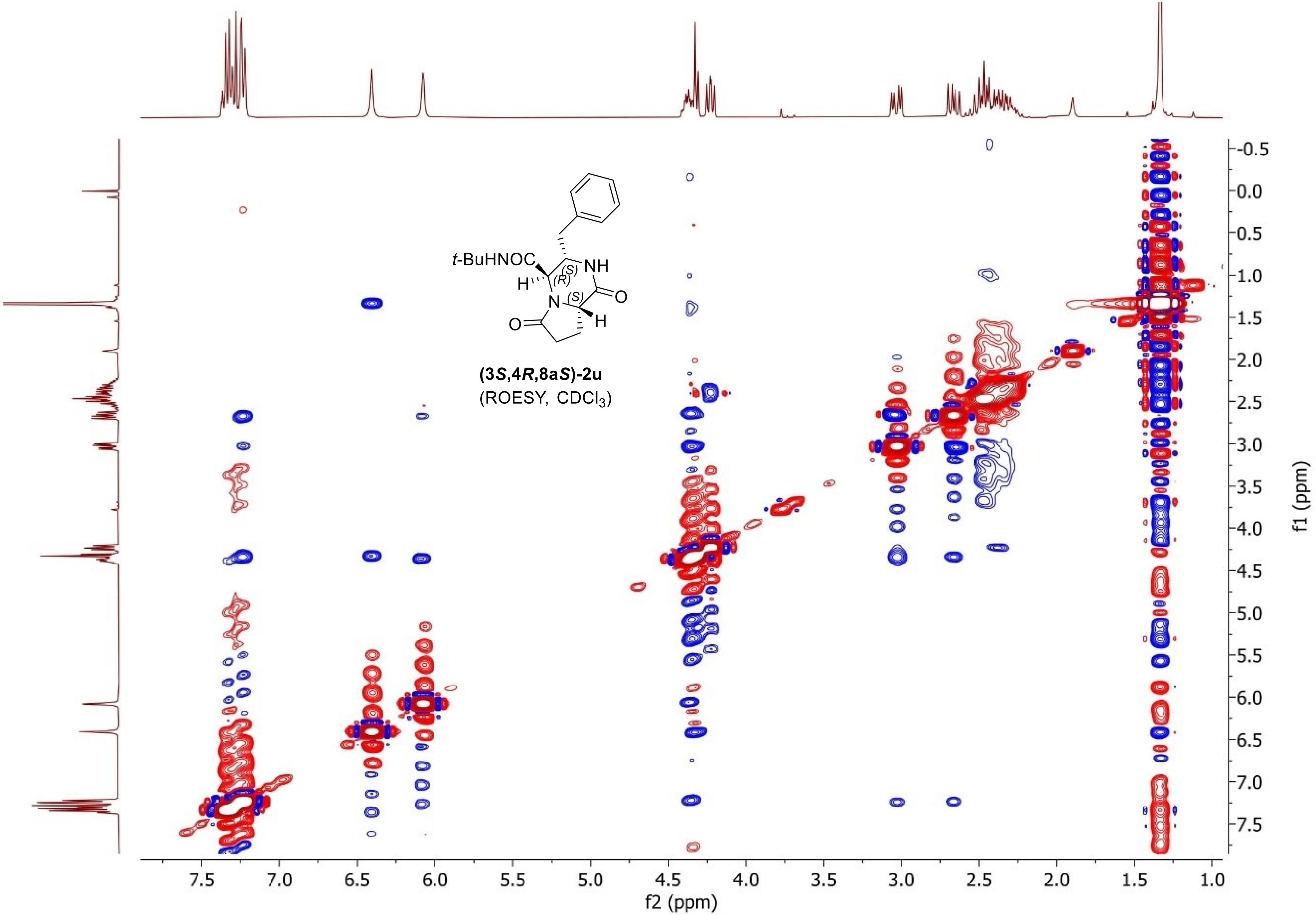
t-BuHNOC
NH
H
N
O
O
H
(S)
(R)
(S)
(3S,4R,8aS)-2u
(ROESY, CDCl3)
f2 (ppm)
f1 (ppm)

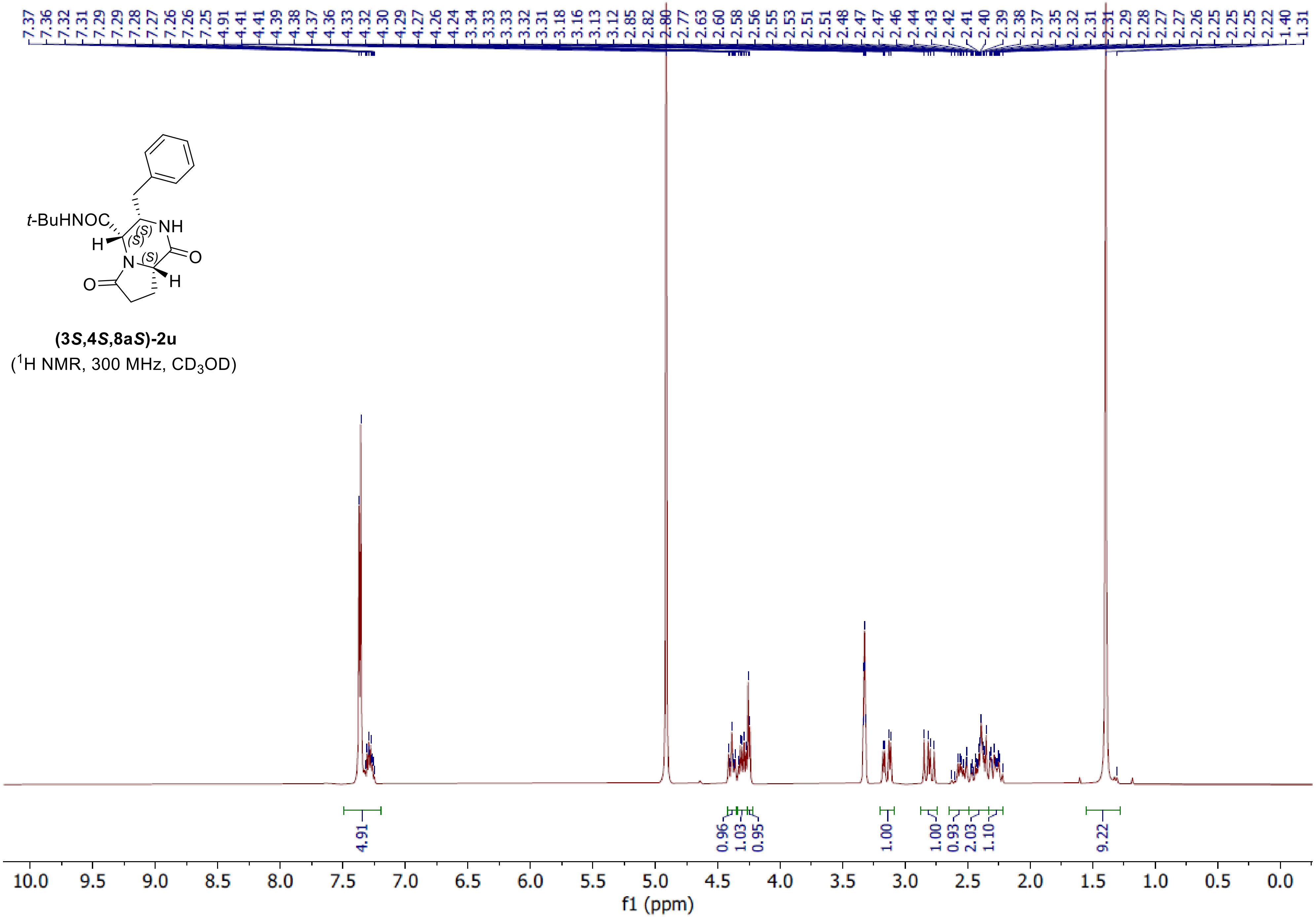

(3*S*,4*S*,8a*S*)-2u
(^{1}H NMR, 300 MHz, CD_3OD)

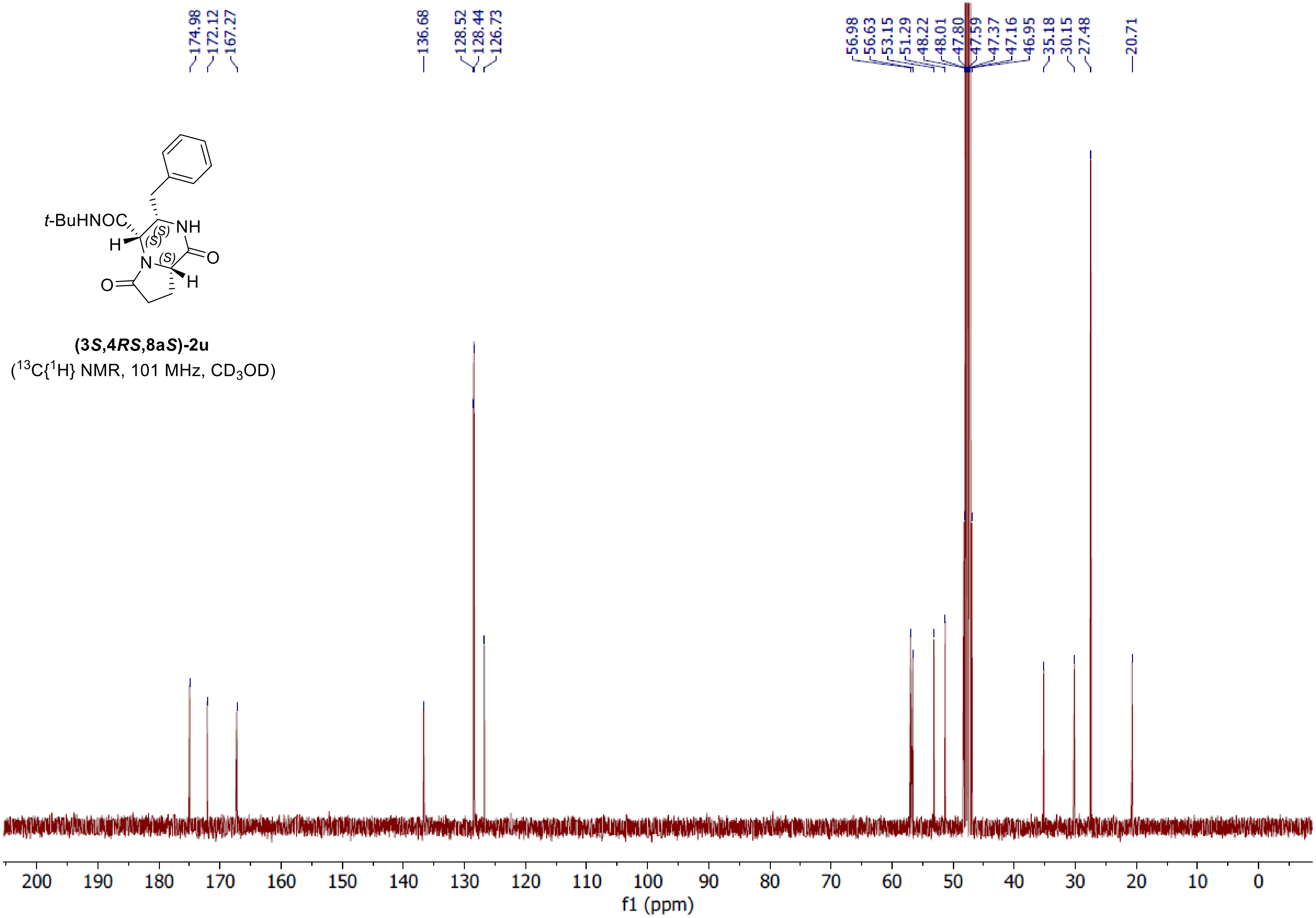

(3*S*,4*RS*,8a*S*)-2u

($^{13}C\{^{1}H\}$ NMR, 101 MHz, CD_3OD)

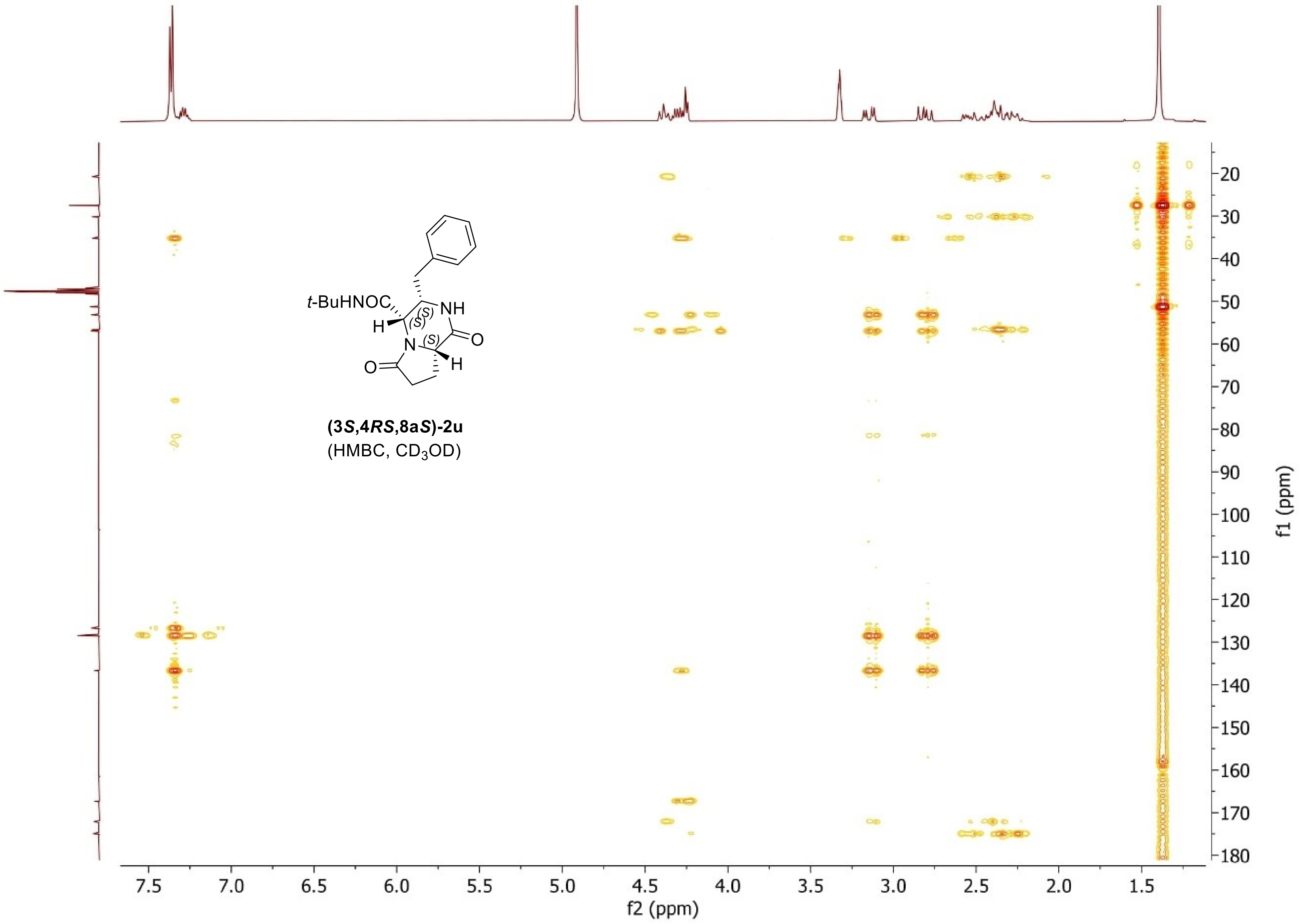
t-BuHNOC
NH
H
(S)
N
(S)
O
O
H
(3S,4RS,8aS)-2u
(HMBC, CD3OD)
f1 (ppm)
f2 (ppm)

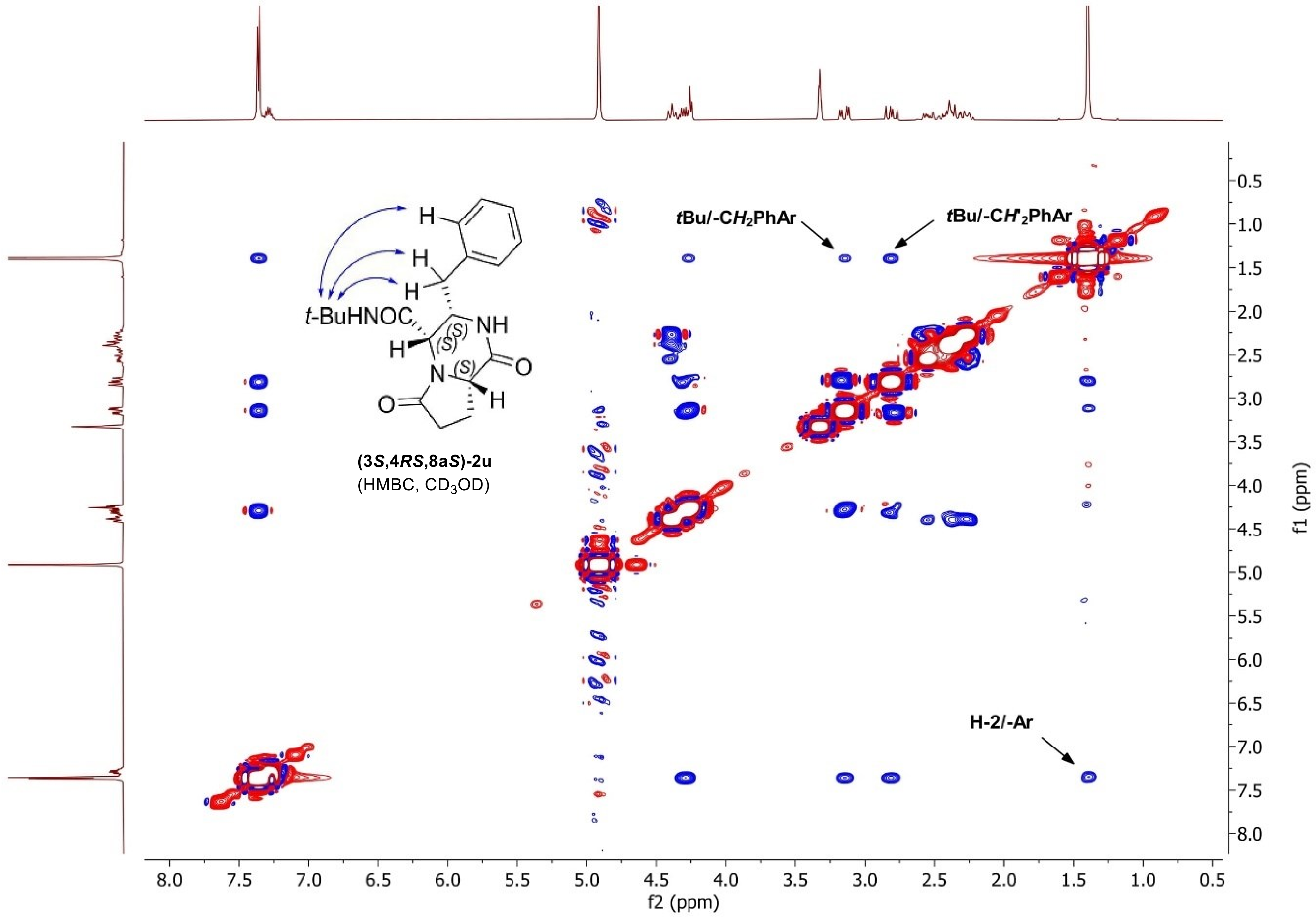

tBu/-CH2PhAr
tBu/-CH'2PhAr
H-2/-Ar
t-BuHNOC
NH
N
O
H
(S)
(3S,4RS,8aS)-2u
(HMBC, CD3OD)
f2 (ppm)
f1 (ppm)

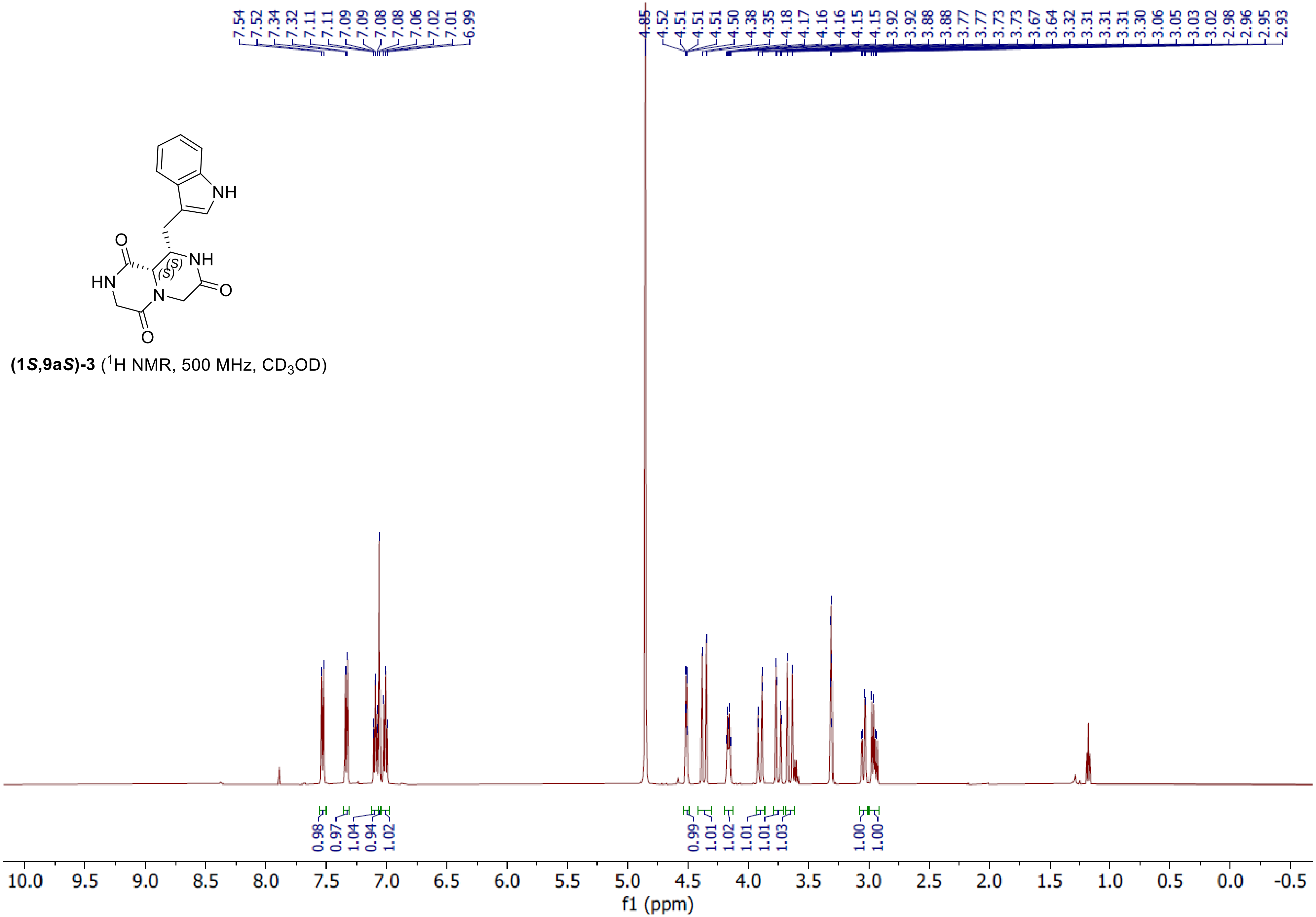

(1*S*,9a*S*)-3 (^{1}H NMR, 500 MHz, CD_3OD)

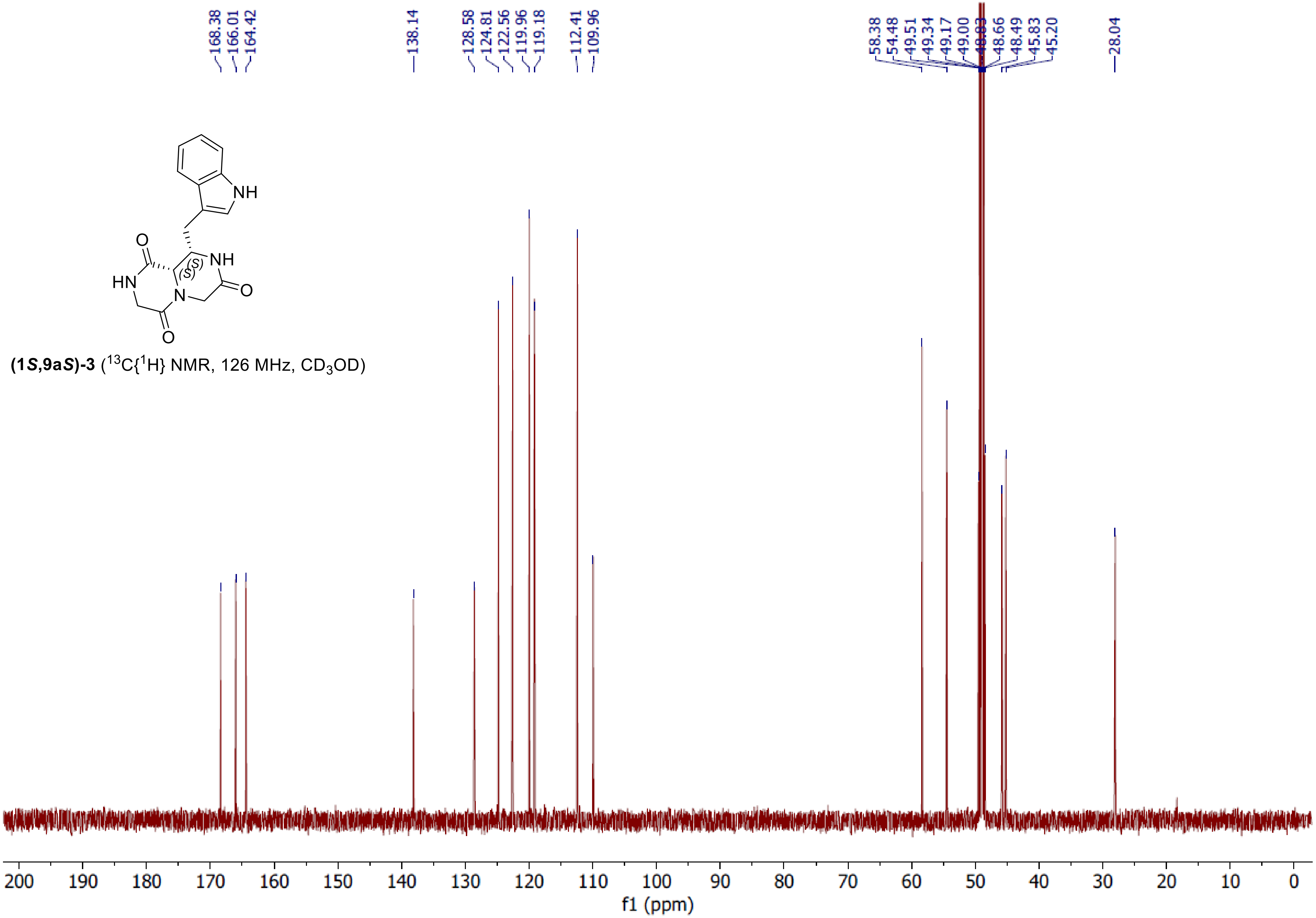

(1*S*,9a*S*)-3 ($^{13}C\{^1H\}$ NMR, 126 MHz, CD_3OD)

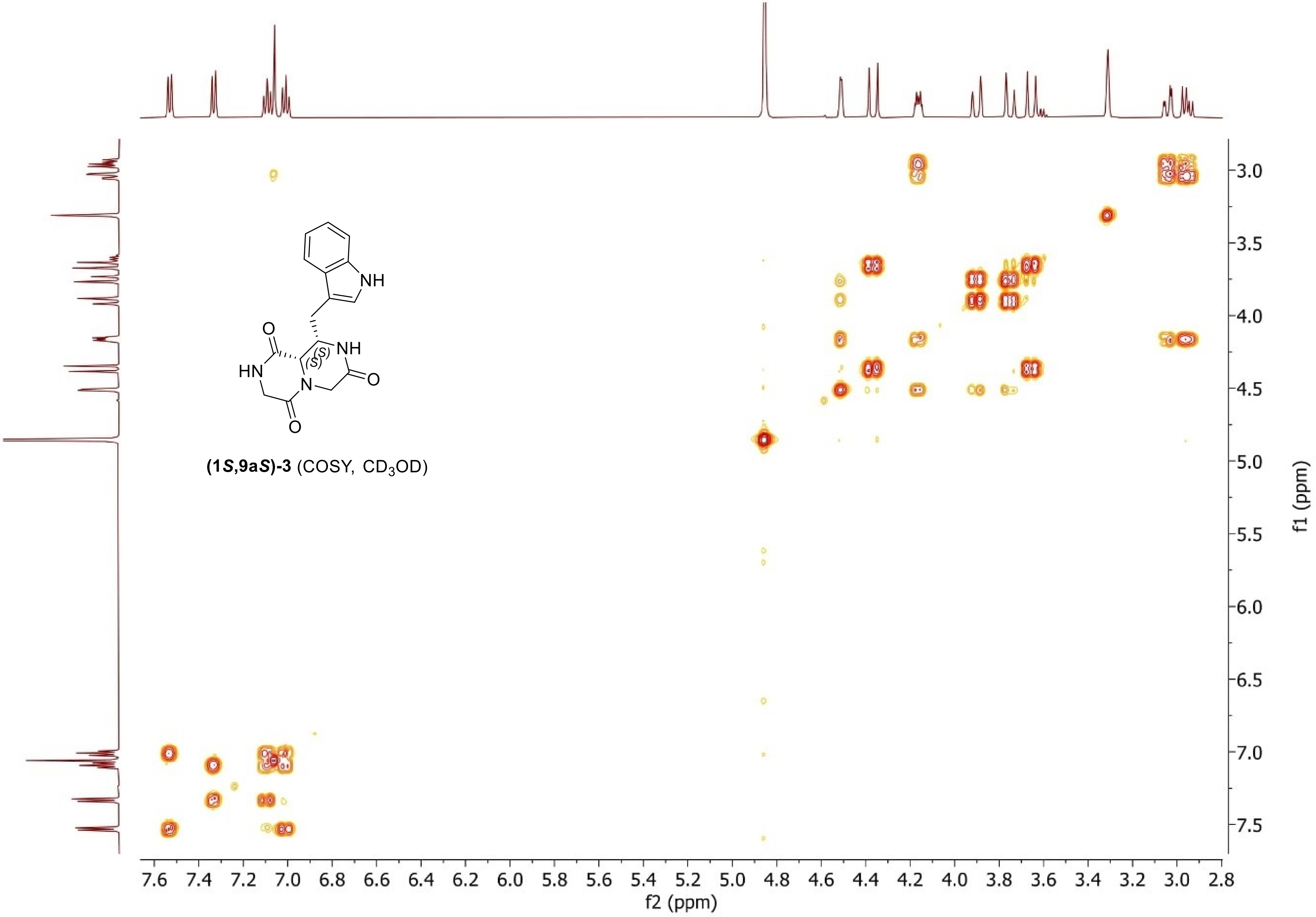

(1*S*,9a*S*)-3 (COSY, CD_3OD)

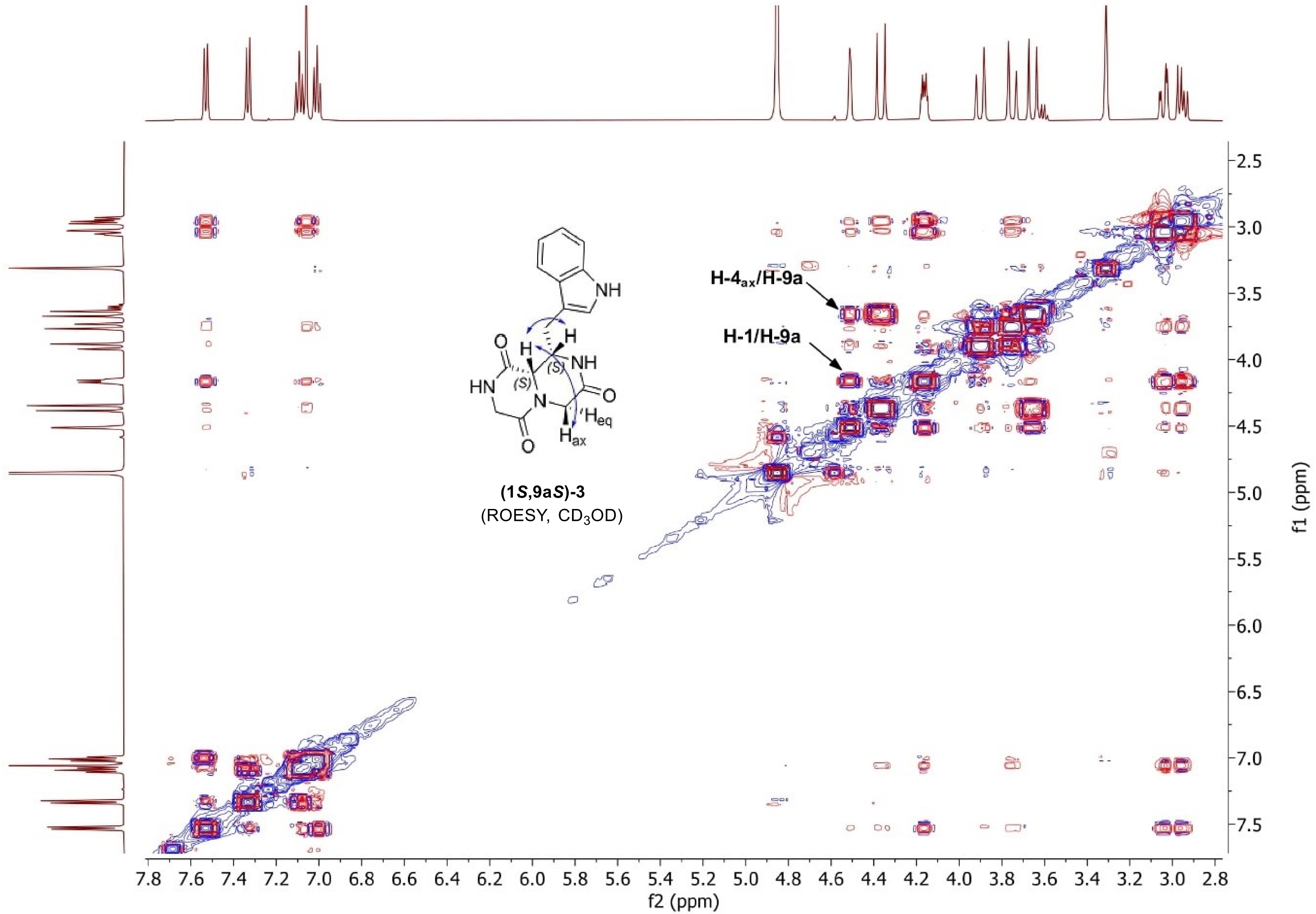

H-4ax/H-9a
H-1/H-9a
NH
O
H
NH
HN
(S)
(S)
N
O
Heq
Hax
O
(1S,9aS)-3
(ROESY, CD3OD)
f1 (ppm)
f2 (ppm)

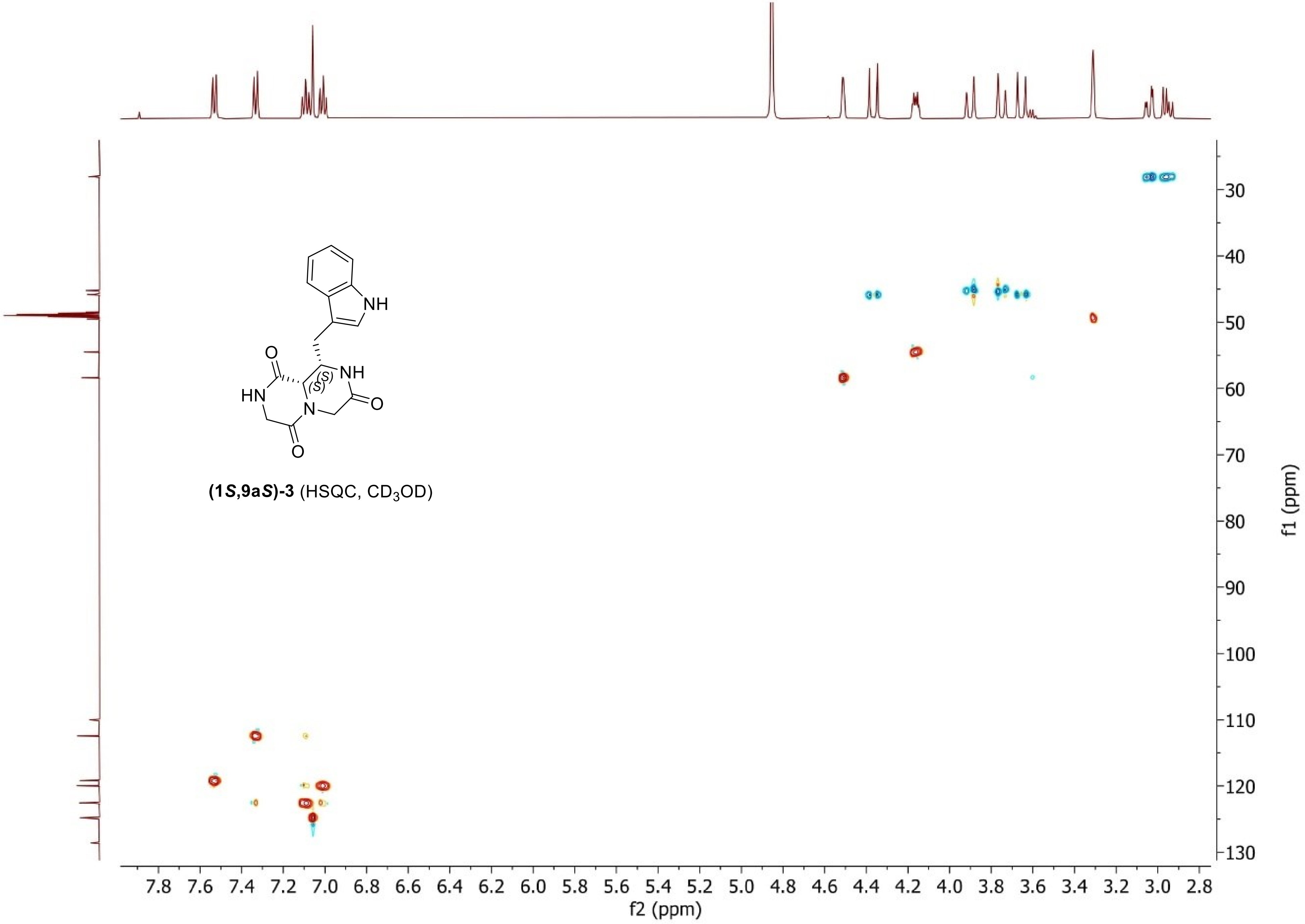

(1*S*,9a*S*)-3 (HSQC, CD_3OD)

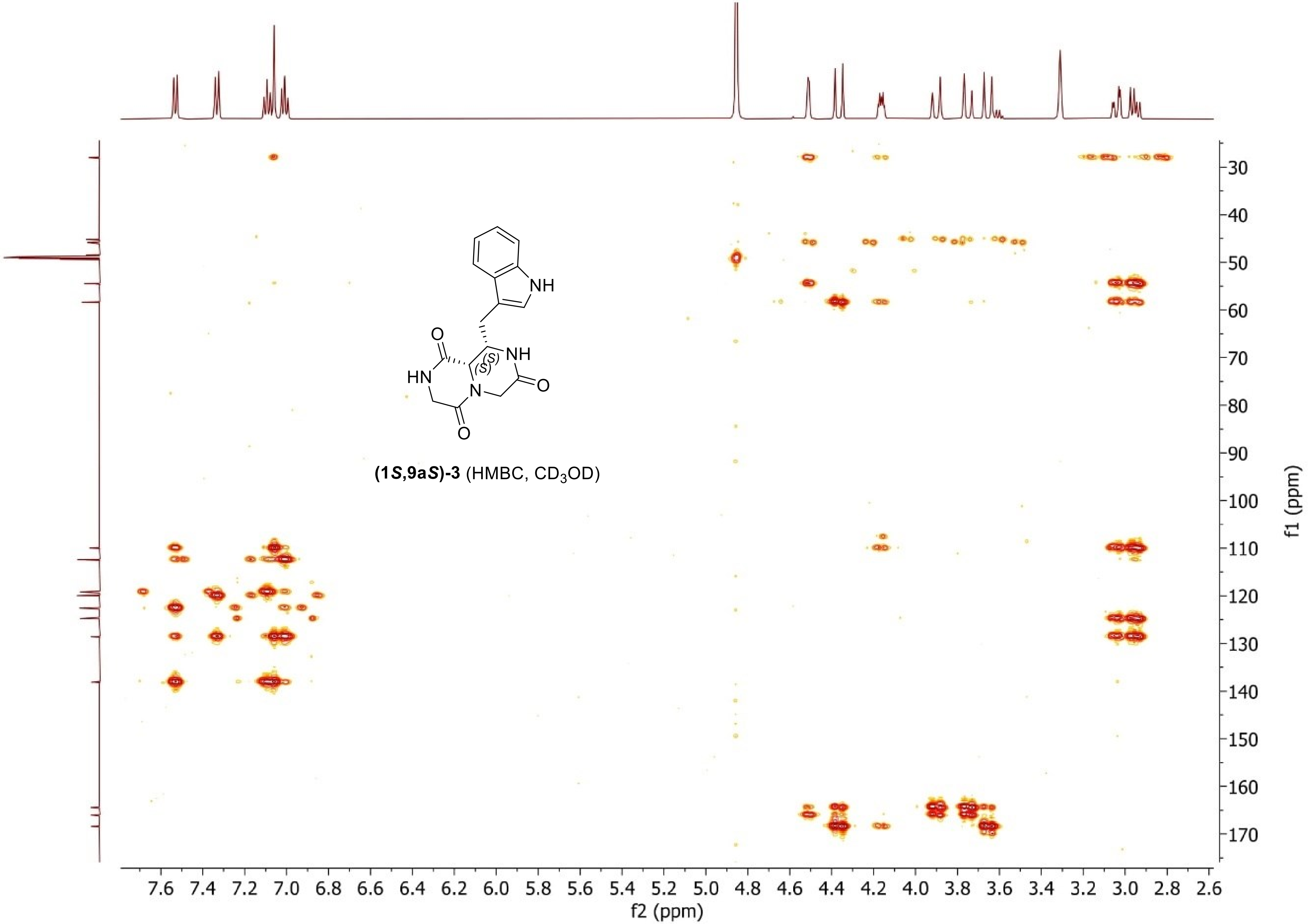
NH
NH
HN
N
O
O
O
(S)
(S)
(1S,9aS)-3 (HMBC, CD$_3$OD)
f1 (ppm)
f2 (ppm)
30
40
50
60
70
80
90
100
110
120
130
140
150
160
170
7.6 7.4 7.2 7.0 6.8 6.6 6.4 6.2 6.0 5.8 5.6 5.4 5.2 5.0 4.8 4.6 4.4 4.2 4.0 3.8 3.6 3.4 3.2 3.0 2.8 2.6

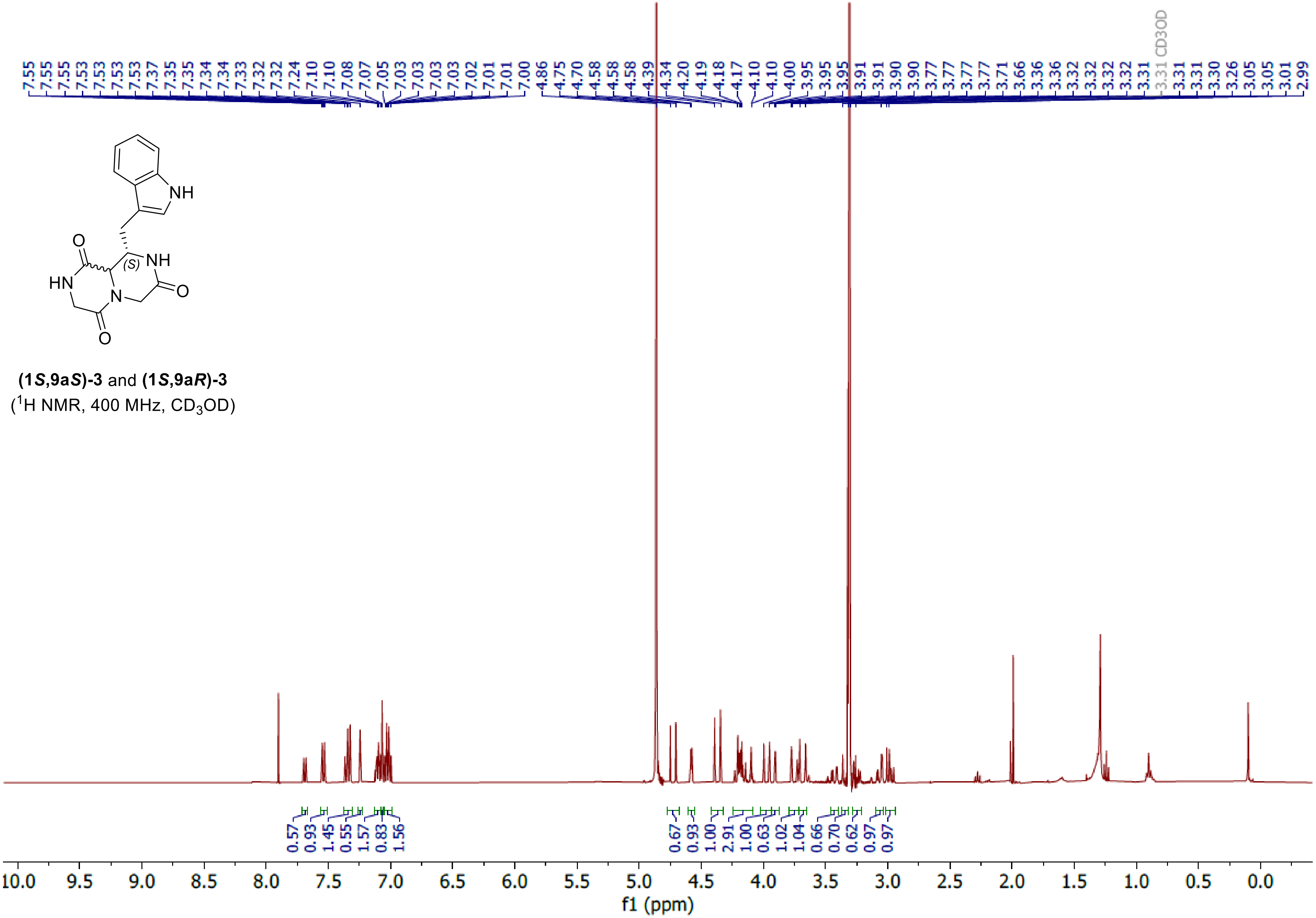

(1*S*,9a*S*)-3 and **(1*S*,9a*R*)-3**
(^{1}H NMR, 400 MHz, CD_3OD)

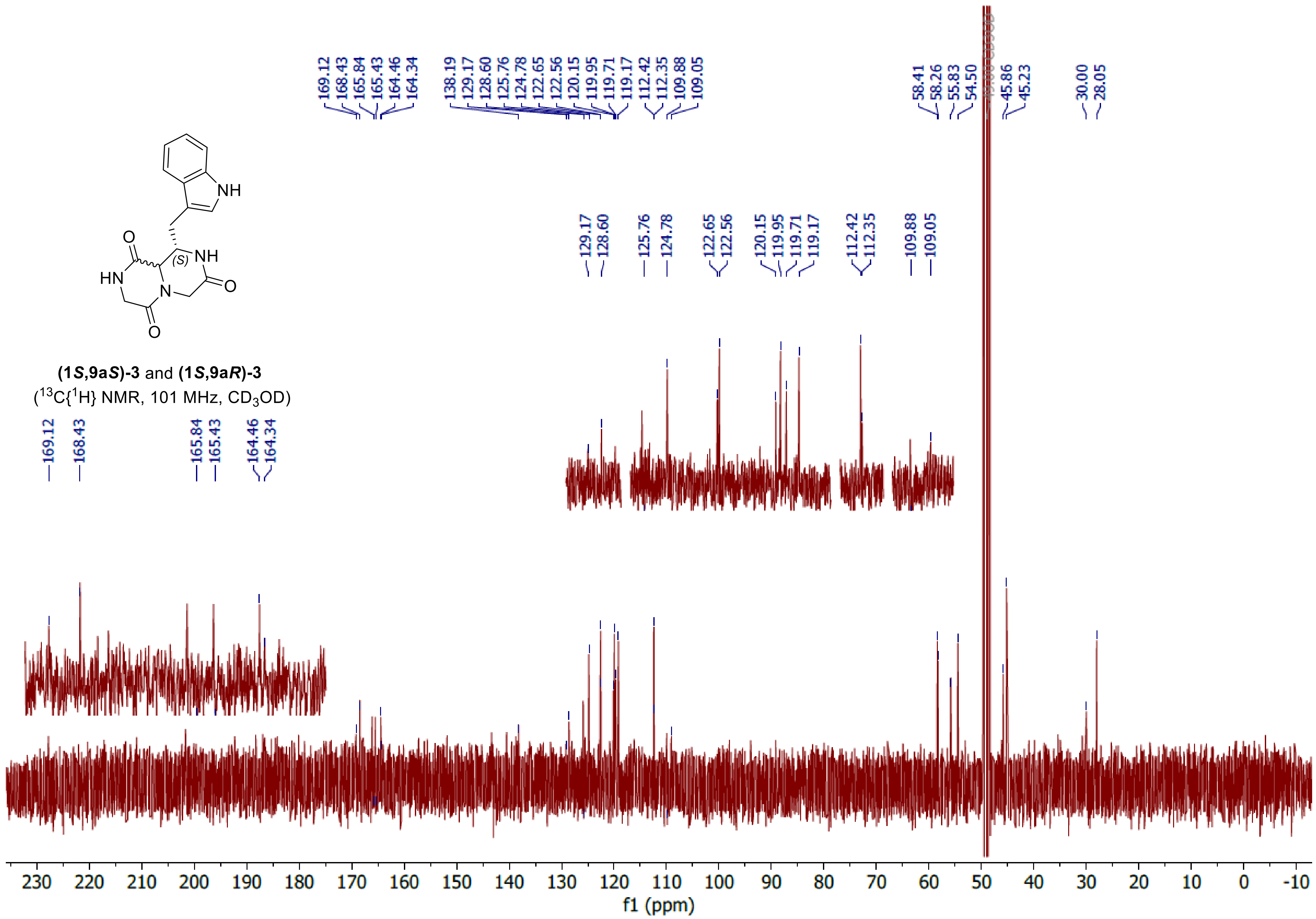

169.12
168.43
165.84
165.43
164.46
164.34
138.19
129.17
128.60
125.76
124.78
122.65
122.56
120.15
119.95
119.71
119.17
112.42
112.35
109.88
109.05
58.41
58.26
55.83
54.50
45.86
45.23
30.00
28.05
NH
O
(S)
NH
HN
N
O
O
(1*S*,9a*S*)-3 and **(1*S*,9a*R*)-3**
($^{13}C\{^1H\}$ NMR, 101 MHz, CD_3OD)
f1 (ppm)
230 220 210 200 190 180 170 160 150 140 130 120 110 100 90 80 70 60 50 40 30 20 10 0 -10

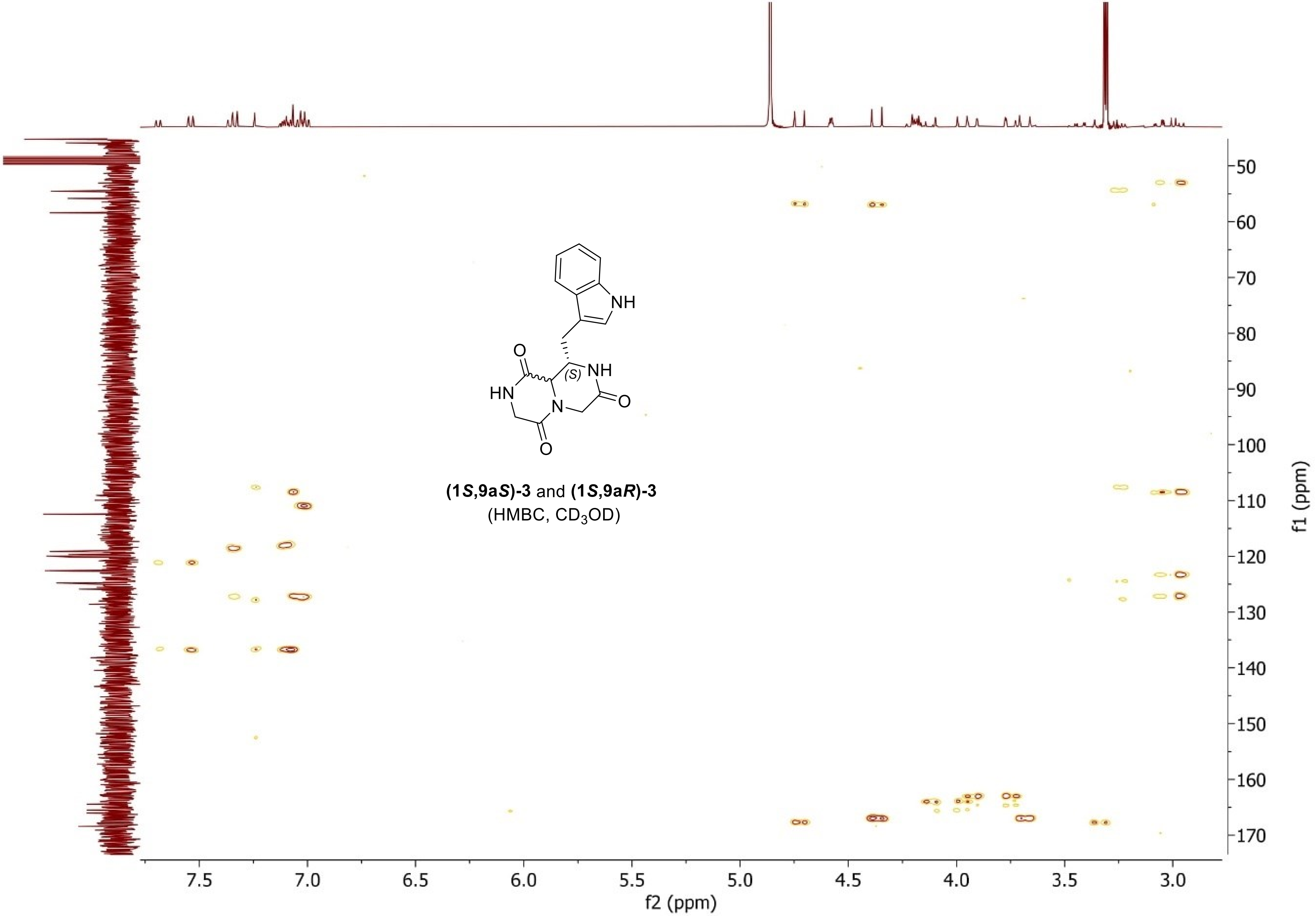
NH
O
(S)
NH
HN
N
O
O
(1S,9aS)-3 and (1S,9aR)-3
(HMBC, CD3OD)
50
60
70
80
90
100
110
120
130
140
150
160
170
f1 (ppm)
7.5
7.0
6.5
6.0
5.5
5.0
4.5
4.0
3.5
3.0
f2 (ppm)

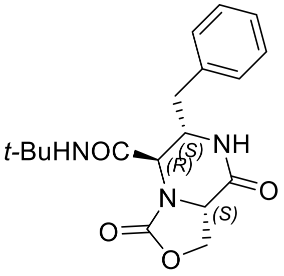

4 (^{1}H NMR, 400 MHz, $CDCl_3$)

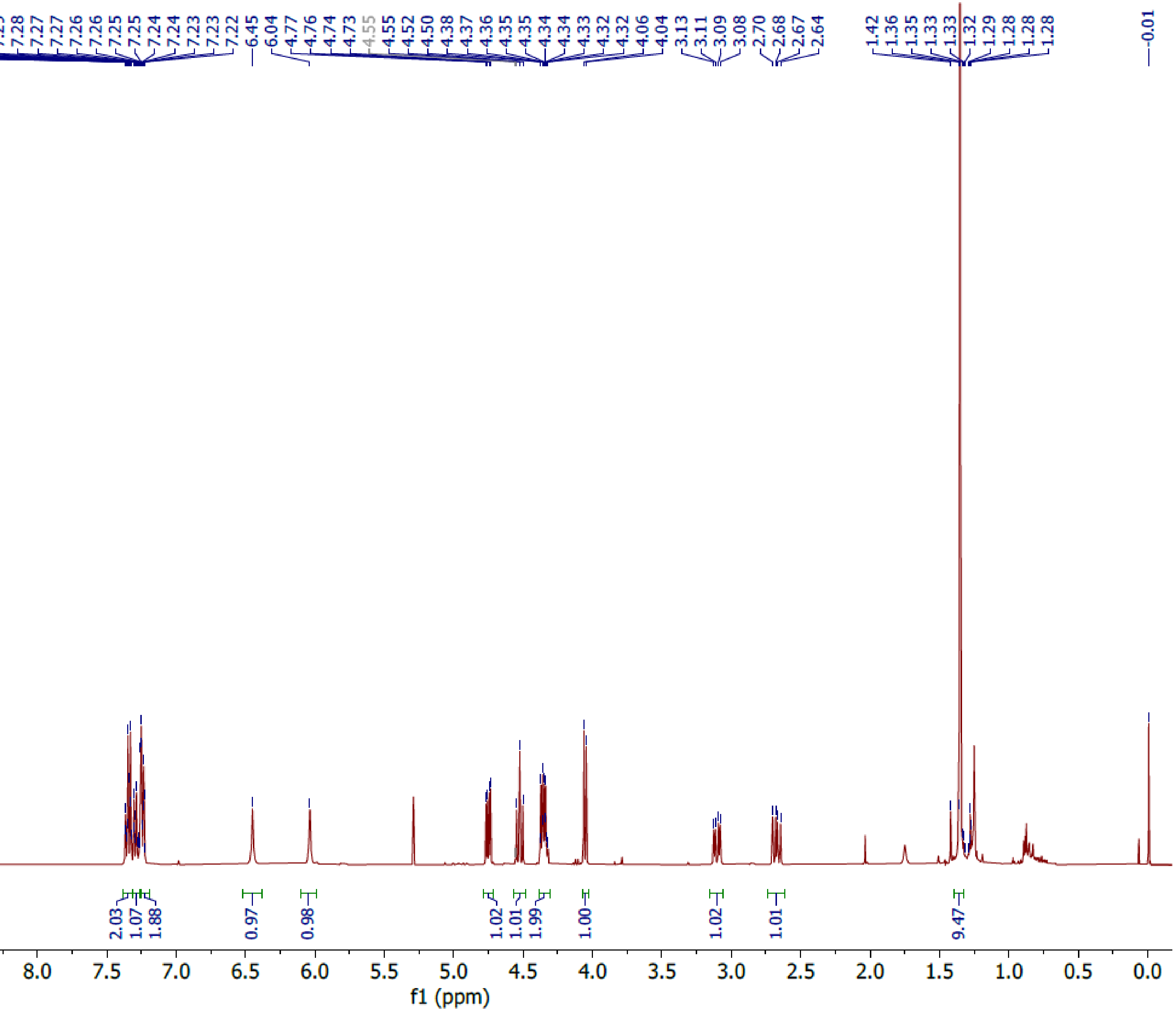

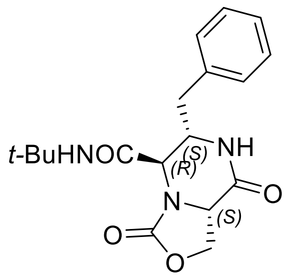

4 ($^{13}C\{^1H\}$ NMR, 101 MHz, $CDCl_3$)

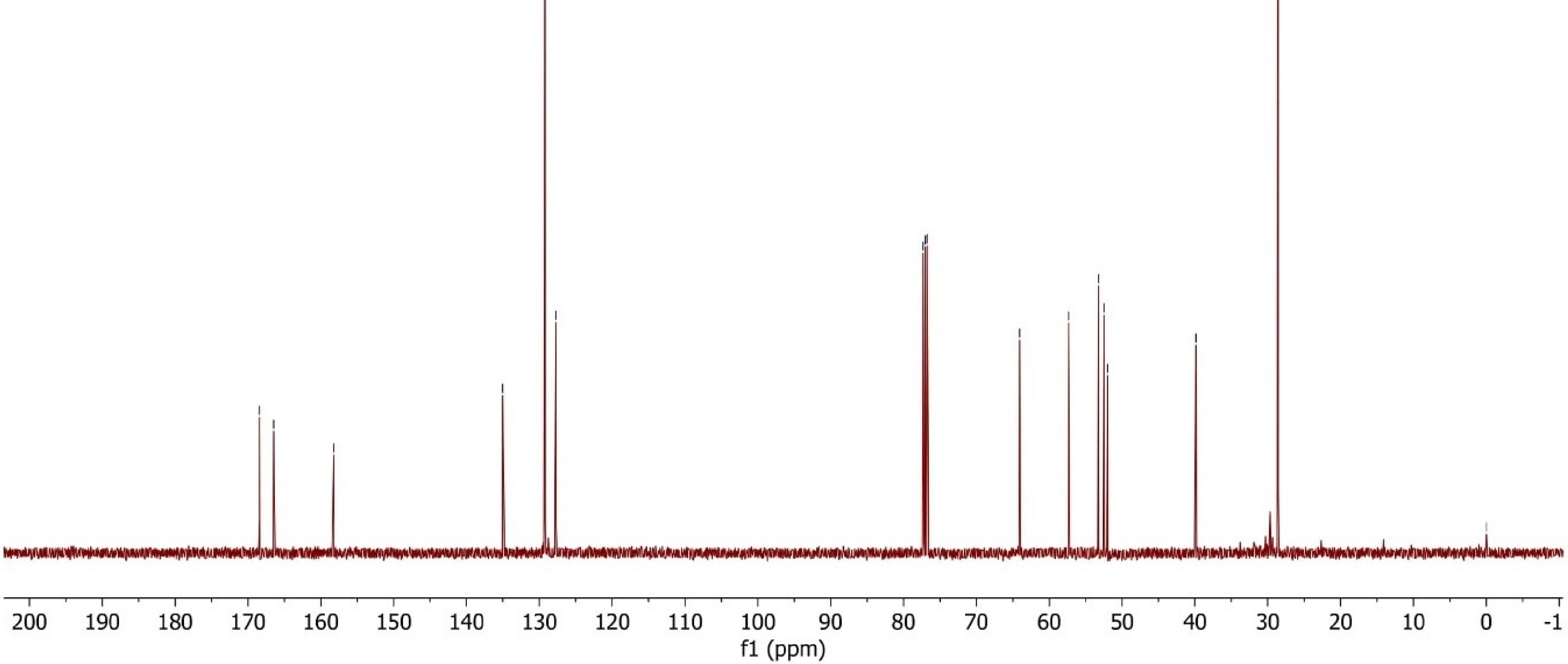

Supplement: Supplementary file 1 — Supplementary file1 (PDF 9417 kb) [file 11030_2023_10760_MOESM1_ESM.pdf]
